# Enzymatic Glycosylation of 4′-Hydroxychalcones: Expanding the Scope of Nature’s Catalytic Potential

**DOI:** 10.3390/ijms252111482

**Published:** 2024-10-25

**Authors:** Paweł Chlipała, Agata Matera, Sandra Sordon, Jarosław Popłoński, Marcelina Mazur, Tomasz Janeczko

**Affiliations:** Department of Food Chemistry and Biocatalysis, Faculty of Biotechnology and Food Science, Wrocław University of Environmental and Life Sciences, 50-375 Wrocław, Poland; agata.matera@upwr.edu.pl (A.M.); sandra.sordon@upwr.edu.pl (S.S.); jaroslaw.poplonski@upwr.edu.pl (J.P.); marcelina.mazur@upwr.edu.pl (M.M.)

**Keywords:** chalcone, 4′-hydroxychalcones, glucosyltransferase, *Glicyne max* sucrose synthase (GmSuSy), enzymatic biotransformation

## Abstract

Chalcones, including 4′-hydroxychalcones, have garnered significant attention in the area of drug discovery due to their diverse pharmacological properties, such as anti-inflammatory, antioxidative, and anticancer effects. However, their low water solubility and bioavailability limit their efficacy *in vivo*. Glycosylation presents a promising approach to enhance the water solubility, stability, and metabolic properties of chalcones. This study investigates the enzymatic glycosylation of eight chemically synthesized 4′-hydroxychalcones using a diverse set of sugar glucosyltransferases from bacterial, plant, and fungal sources, alongside *Glycine max* sucrose synthase (GmSuSy) in a cascade reaction. Among the tested enzymes, five exhibited a remarkable versatility for glycoside production, and for large-scale biotransformation, flavonoid 7-*O*-glucosyltransferase Sbaic7OGT from *Scutellaria baicalensis* was selected as the most effective. As a result of the experiments conducted, eight *trans*-chalcone glycosides were obtained. During the purification of the reaction products, we also observed the isomerization of the products by simple sunlight exposure, which resulted in eight additional *cis*-chalcone glycosides. This study highlights the novel use of a cascade reaction involving Glycine max sucrose synthase (GmSuSy) for the efficient glycosylation of *trans*-4′-hydroxychalcones, alongside the unexpected discovery of *cis*-chalcone glycosides during the purification process.

## 1. Introduction

In the pursuit of novel bioactive compounds with therapeutic potential, natural products have long stood as a reservoir of inspiration and innovation. Among these, chalcones, characterized by their versatile chemical structure and diverse pharmacological activities, have garnered considerable attention in the area of drug discovery and development [1,2]. Chalcones, bearing a hydroxyl group at the *para*-position on one of the aromatic rings, exhibit promising biological properties, ranging from antioxidant and anti-inflammatory to anticancer and antimicrobial activities [3,4,5]. 4′-Hydroxychalcones occur naturally in various plant species [6]. 4′-Hydroxychalcone and 4′-hydroxydihydrochalcones are a diagnostic chemical cluster in the *Dracaena* species. Dihydrochalcones, e.g., loureirin A and loureirin B, are often used as the chemical markers for the quality control of dragon’s blood [7]. Dragon’s blood (Chinese name: Xuejie), which comprises red resins obtained from several plants (27 species from four families), is drawing worldwide interests in medicinal applications owing to its broad pharmacological spectrum such as promoting blood circulation, regenerating muscle, relieving swelling and pain, maintaining hemostasis, etc. [8]. A total of 13 chalcones have been identified to date from the six *Dracaena* species [7,9].

The biological activities of 4′-hydroxychalcones stem from their ability to modulate various cellular pathways and molecular targets [8,10,11]. These compounds have demonstrated potent antioxidant effects, scavenging reactive oxygen species (ROS) and protecting cells from oxidative damage [12]. Additionally, 4′-hydroxychalcones exhibit anti-inflammatory properties by inhibiting pro-inflammatory mediators and cytokines, thereby attenuating inflammatory responses implicated in chronic diseases such as arthritis and inflammatory bowel disease.

In addition to their antioxidant, anti-inflammatory, and anticancer properties, 4′-hydroxychalcones possess antimicrobial activity against a broad spectrum of pathogens, including bacteria, fungi, and viruses. These compounds exhibit antibacterial effects by disrupting microbial cell membranes, inhibiting biofilm formation, and interfering with bacterial quorum sensing systems. Furthermore, 4′-hydroxychalcones demonstrate antifungal activity by inhibiting fungal growth and biofilm formation, offering potential applications in the treatment of fungal infections [13].

However, the clinical utility of these compounds is often hampered by their low water solubility and limited bioavailability [14,15,16]. To overcome these challenges, glycosylation has emerged as a viable strategy to enhance the solubility, stability, and pharmacokinetic properties of chalcones [17,18]. Glycosylation, the enzymatic process of attaching sugar moieties to organic molecules, is a pivotal biosynthetic pathway in nature, contributing to the structural diversity and biological functionality of numerous natural products [19,20,21]. While whole-cell biocatalysis has been explored for this purpose, it often leads to undesired reductions in the double C-C bond of chalcones, rendering it unsuitable for glycoside production [22,23,24,25]. In contrast, enzymatic biotransformations offer higher selectivity, making them an attractive alternative for glycosylation reactions [21,26].

In this study, we investigated the enzymatic glycosylation of eight 4′-hydroxychalcones synthesized chemically using a panel of eight glucosyltransferases from different origins (bacterial, plant, and fungal) in an enzymatic cascade coupled with *Glycine max* sucrose synthase (GmSuSy). We performed the reaction under the conditions optimized in our previous publication [27]. Our aim was to evaluate the versatility of these enzymes for glycoside production and identify optimal candidates for large-scale biotransformation.

Here, we present the results of our experiments, highlighting the successful synthesis of eight *trans*-chalcone glycosides. Additionally, we observed an intriguing phenomenon of isomerization during the purification process, leading to the formation of eight *cis*-chalcone glycosides. These findings underscore the untapped potential of enzymatic biotransformation as a selective and efficient method for the glycosylation of 4′-hydroxychalcones, offering new opportunities for the development of bioactive glycoconjugates with improved pharmaceutical properties.

## 2. Results and Discussion

The enzymatic glycosylation of the synthesized 4′-hydroxychalcones was carried out using a panel of sugar nucleotide-dependent glucosyltransferases from various sources, coupled with *Glycine max* sucrose synthase (GmSuSy) for uridine diphosphate glucose (UDP-glucose) regeneration. The enzymatic glycosylation reaction was performed based on the conditions developed and described in our earlier publication [27]. The optimized parameters included the following: The optimal induction temperature for the co-expression of glycosyltransferases (GTs) with GmSuSy was determined to be 25 °C. This temperature was selected to enhance enzyme expression while minimizing the formation of inactive aggregates. A concentration of 0.5 mM UDP was found to be optimal for the reaction, as it provided a sufficient supply of the glycosyl donor while minimizing the inhibitory effects of excess of UDP. The pH of the reaction mixture was crucial for the enzyme activity and the regioselectivity of the glycosyltransferases. The optimal pH range was identified as 7.5 to 8.0, using a HEPES buffer system. This pH range was selected based on its ability to maintain enzyme stability and activity, as well as to influence the distribution of glycosylated products. The substrate concentration was carefully optimized to ensure efficient glycosylation. The reactions were conducted with varying concentrations of the 4′-hydroxychalcones to determine the optimal substrate load that would maximize the glycoside yield without causing substrate inhibition or precipitation. The use of crude protein extracts as a catalyst source was evaluated. While cost-effective, these extracts contained native glycosidases from *E. coli* BL21 (DE3) host cells, which could hydrolyze the glycosylated products. This challenge was addressed by supplementing the reactions with additional UDP to counteract any loss of product due to glycosidase activity.

**Figure 1:**
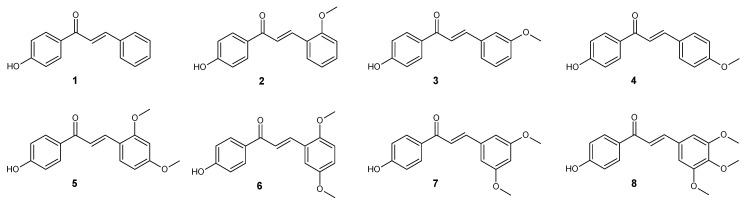
*Trans*-4′-hydroxychalcones obtained by chemical synthesis.

**Scheme 1:**
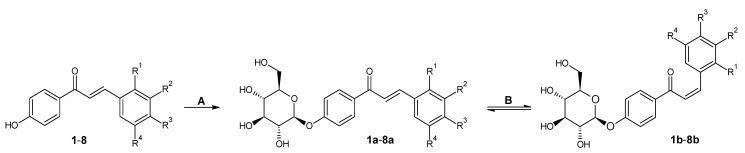
Enzymatic glycosylation of *trans*-4′-hydroxychalcones and observed *trans*–*cis* isomerization catalyzed by light exposure.

This biotransformation resulted in the successful synthesis of eight *trans*-chalcone glycosides. Remarkably, an isomerization phenomenon was observed during the purification process, leading to the formation of the corresponding *cis*-chalcone glycosides. The chemical synthesis of the 4′-hydroxychalcones was accomplished through a Claisen–Schmidt condensation reaction, yielding eight distinct derivatives (Figure 1). The biotransformation reactions were monitored using thin-layer chromatography (TLC) and ultra-high-performance liquid chromatography (UHPLC). The isolation and structural confirmation of the eight 4′-hydroxychalcones and their glycosides were achieved through nuclear magnetic resonance (NMR) spectroscopy, including ^1^H and ^13^C NMR, alongside advanced two-dimensional techniques such as COSY, HSQC, and HMBC. The *trans*–*cis* isomerization (Scheme 1 and Table 1) was particularly noted under UV irradiation conditions during product purification, as evidenced by the distinct chemical shifts in the NMR spectra.

The glycosylation of the 4′-hydroxychalcones was carried out using appropriate buffers and substrates under the conditions described in the Materials and Methods section (Section 3). The reactions involved a variety of enzymes from bacterial, plant, and fungal origins. The amount of enzyme was carefully selected to prevent side reactions, such as the formation of diglycosides [21]. We tested eight glycosyltransferases: two bacterial–macrolide glycosyltransferase OleD from *Streptomyces antibioticus* [28] and glucosyltransferase YjiC from *Bacillus licheniformis* [29], four plant–flavonoid 7-*O*-glucosyltransferase *Sbaic*7OGT from *Scutellaria baicalensis* [30], betanidin 5-*O*-glucosyltransferase Bet5OGT from *Cleretum bellidiforme* [31], phloretin 2′-*O*-glycosyltransferase UGT88F1 from apple (*Malus x domestica*) [32], and UDP-glucosyl transferase UGT85A2 from *Arabidopsis thaliana* [33]; for comparison, we also tested di-C-glycosyltransferase GgCGT identified in *Glycyrrhiza glabra* [34,35] and one novel fungal glucosyltransferase *Bb*GT278 from *Beauveria bassiana* [27]. The reactions were conducted under optimal conditions, which allowed for efficient glycosylation with the minimal formation of side products such as diglycosides. The progress of the reactions was monitored using UHPLC, allowing for the precise control and evaluation of the glycosylation efficiency.

By conducting this experiment, we gained insights into the catalytic capabilities of the tested enzymes with respect to the 4′-hydroxychalcones we used (Table 2). We demonstrated that five out of the studied enzymes effectively glycosylated most of the tested compounds. We observed that the position and number of the methoxy groups in the substrate molecule influenced the glycosylation efficiency. We confirmed that the enzyme YjiC is an effective tool for the glycosylation of phenolic compounds containing hydroxyl groups in their structure [36,37,38]. The 4′-hydroxychalcones (**1**–**7**) we studied underwent glycosylation with good conversion rates of 54–68% under the experimental conditions used with this enzyme. Only for compound **8** was a lower conversion rate of 27% observed.

However, we recorded even higher conversion rates for the tested substrates in reactions with two plant-derived enzymes (Sbaic7OGT and Bet5OGT). Betanidin 5-*O*-glucosyltransferase (Bet5OGT) from *Cleretum bellidiforme* is an enzyme that plays a crucial role in the biosynthesis of betalains, pigments found in various plants, particularly in the *Caryophyllales* order [39]. This enzyme catalyzes the transfer of glucose from UDP-glucose to the 5-hydroxyl group of betanidin, resulting in the formation of glucosylated betanidin derivatives, which contribute to the pigmentation of fruits and flowers in these plants [31]. However, betanidin 5-*O*-glucosyltransferase (Bet5OGT) is capable of converting certain flavonoids, specifically *ortho*-dihydroxylated flavonols and flavones, preferentially at the B-ring 4′-hydroxyl group [27,31]. In this study, we demonstrated that 4′-hydroxychalcones are also effectively converted by this enzyme, with the conversion of the substrate to 4′-glycoside presented in Table 2. A comparable glycoside formation efficiency was noted for the enzyme *Sbaic*7OGT.

Flavonoid 7-*O*-glucosyltransferase, referred to as *Sbaic*7OGT, is an enzyme derived from *Scutellaria baicalensis*, a plant known for its medicinal properties [27,40]. This enzyme plays a crucial role in the biosynthesis of flavonoid glycosides, particularly in the conversion of baicalein to baicalein 7-*O*-glucoside by utilizing UDP-glucose as a sugar donor [30,41]. We have shown that this enzyme, known for catalyzing the transfer of glucose to the 7-hydroxyl group of flavonoids, is also able to efficiently convert 4′-hydroxychalcones. Compounds **1**–**7** underwent glycosylation under the reaction conditions we used with this enzyme, achieving conversion rates of 75–85%. In contrast, compound **8** underwent glycosylation with a conversion rate of 35%.

We observed significantly clearer substrate specificity (**1**–**8**) for two additional glucosyltransferases: OleD and BbGT278 (Table 2). The macrolide glycosyltransferase OleD, encoded by the oleD gene in *Streptomyces antibioticus*, plays a crucial role in the biosynthesis and modification of the macrolide antibiotic oleandomycin [42]. This enzyme is responsible for the glycosylation of various macrolides, facilitating the transfer of a glucose moiety from UDP-glucose (UDP-Glc) to the antibiotic substrate [43]. Choi et al. demonstrated that OleD effectively glycosylates several flavonoids, including apigenin, chrysin, daidzein, genistein, kaempferol, and luteolin [44]. However, it has been noted that the degree of glycosylation and the specific positions on the flavonoid where glycosylation occurs can vary. As a result of using this enzyme, we obtained glycosides from compounds **1** and **3**–**7** with conversion rates above 50%. However, compounds **2** and **8** underwent glycosylation with the conversion rates not exceeding 10%.

BbGT278 is a versatile glucosyltransferase enzyme derived from the fungus *Beauveria bassiana* AM278 *(*GenBank: OR545370.1) This enzyme has been characterized by its ability to glycosylate various substrates, particularly flavonoids, enhancing their solubility and stability [27]. However, in this study, we demonstrated that the enzyme catalyzes the glycosylation of 4′-hydroxychalcones with varying conversion rates depending on the substitution position and the number of methoxy groups in the substrate molecule. The highest conversion rate (above 75%) was observed for *trans*-4′-hydroxy-4-methoxychalcone (**4**). A nearly 70% conversion was recorded for the glycosylation of *trans*-4′-hydroxy-chalcone (**1**). The compounds *trans*-4′-*O*-β-D-(glucopyranosyl)-3-methoxychalcone (**3a**) and *tran*s-4′-*O*-β-D-(glucopyranosyl)-2,4-dimethoxychalcone (**5a**) were formed with conversion rates over 50%. In contrast, *trans*-4′-*O*-β-D-(glucopyranosyl)-2-methoxychalcone (**2a**) and *trans*-4′-*O*-β-D-(glucopyranosyl)-2,5-dimethoxychalcone (**6a**) were formed with conversion rates of 33.1% and 38.1%, respectively. The conversion rate for *trans*-4′-hydroxy-3,5-dimethoxychalcone (**7**) slightly exceeded 10%, while for compound **8**, the conversion rate was below 1%.

The di-C-glycosyltransferase, identified as GgCGT from *Glycyrrhiza glabra*, is an enzyme that catalyzes the addition of two glucose units to flavonoid substrates, specifically through C-glycosylation [35,45]. The specificity and efficiency of GgCGT can vary depending on the structure of the flavonoid substrate. GgCGT catalyzes the two-step di-C-glycosylation of flopropione-containing substrates (for example phloretin) with conversion rates of >98%. However, quercetin and genitein are converted by this enzyme to both *C*- and *O*-glycosides with a significantly lower conversion [35,45]. On the other hand, chalcones containing a hydroxyl group at C-4 and C-4′ in their structure only undergo *O*-glycosylation [35]. Our research demonstrated that this enzyme is also capable of *O*-glycosylating 4′-hydroxychalcones (**1** and **3**–**7**). The corresponding glycosides were not observed in reaction mixtures with *trans*-4′-hydroxy-2-methoxychalcone (**2**) and *trans*-4′-hydroxy-3,4,5-trimethoxychalcone (**8**) as substrates.

The enzyme *Sbaic*7OGT was selected for subsequent experiments due to its versatility with the 4′-hydroxychalcones being studied. To determine the most suitable *Sbaic*7OGT extract concentration to perform the enzymatic glycosylation, we prepared four solutions, as described in Table 3. The solutions were prepared in total volume of 800 μL. Additionally, each solution consisted of 500 mM of sucrose. The reactions were performed in triplicates. 

Substrate **1** was chosen as model compound, and it was added into each sample in four portions of 20 μL each: in the beginning of the reaction, after 30 min, after 60 min, and after 90 min from the start. This procedure was used based on our previous studies [27]. The convertion rate was monitored with the use of UHPLC. Samples were collected after 1 h, 2 h, 3 h, 4 h, 6 h, 18 h, and 24 h of the reaction. The samples were prepared as follows: With 200 μL of ethyl acetate, 20 μL of the reaction mixture was extracted and centrifuged. Next, 50 μL of the top layer was sampled into a glass vial and 450 μL of HPLC grade methanol was added. The prepared samples were run on the UHPLC. The convertion rates are presented in Table 4.

As the **b** variant gave satisfactory convertion rates and allowed materials to be saved, we decided to perform all the preparative scales with the use of this variant. At the same time, this variant ensured obtaining glycosides in quantities that allowed for determining their structure using spectroscopic methods. The reactions were performed in 50 mL falcons, each containing 20 mL of protein extract, 16 mL of HEPES buffer, 4 mL of 100 mM substrate solution in DMSO, 6.85 g of sucrose, and 9 mg of UDP. The substrate was added in four portions of 1 mL each: in the beginning of the reaction, after 30 min, after 60 min, and after 90 min of the reaction. The reactions were monitored with the use of UHPLC, exactly the same as described previously. After the overnight incubation of the reaction at 30 °C and agitation 120 rpm, the reaction mixture was extracted three times with ethyl acetate. The extracts were collected, dried over anhydrous MgSO_4_, and evaporated in vacuo. The obtained debris was purified by preparative TLC with the use of a chloroform:methanol 9:1 (*v/v*) mixture as an eluent. According to the procedure described earlier, the glycosides and their aglycones were separated [46]. The separated product was scraped off the TLC plate, gel was extracted three times with ethyl acetate, and the filtered extracts were collected and evaporated in vacuo. This procedure was performed for each of the substrates **1**–**8**. The purified products were analyzed involving ^1^H NMR, ^13^C NMR, HSQC, HSBC, COSY, and UHPLC. The quantity of the products of the enzymatic reactions in the reaction mixture over time are presented in Table 5.

During the enzymatic experiments, we observed only the corresponding *trans*-chalcones and their *trans*-glycosides. However, after purifying and isolating the glycosides and performing NMR analysis, we discovered that the *cis*-isomers of the glycosides were also present in the samples. This *trans–cis* isomerization was most likely caused by the method used to isolate the products of the enzymatic reaction. The enzymatic reactions were conducted in Falcon tubes without exposure to light. However, the purification process was carried out on a laboratory bench under daylight. This phenomenon has been thoroughly described in other publications, where authors also report the occurrence of similar *trans–cis* photochemical isomerization in related chalcone structures [6,47,48]. The observed *trans–cis* isomerization highlights the need for the careful control of purification conditions to prevent unwanted light-induced structural changes. To minimize this effect, future studies should consider conducting purification steps in light-controlled environments, such as in the dark or under UV-filtered conditions, to ensure the stability of the *trans*-isomers. Furthermore, we plan to investigate the potential impact of this isomerization on the biological activity of the isolated *cis*- and *trans*-chalcone glycosides, as it is well known that different isomers may exhibit distinct pharmacological properties. These planned studies will help to elucidate whether *cis*-chalcones offer differing therapeutic benefits compared to their *trans* counterparts

The NMR data for the *trans*-4′-hydroxychalcones obtained from the chemical synthesis and the products of the enzymatic glycosylation are presented in Table 6 and Table 7, as well as in the Materials and Methods section. Based on analyses including ^1^H-NMR, ^13^C-NMR, and two-dimensional techniques such as HMBC (heteronuclear multiple bond correlation), HMQC (heteronuclear multiple quantum coherence), and COSY (correlation spectroscopy), the structures of the respective *trans*-4′-hydroxychalcones and their glycosides were determined. The coupling constants, multiplicity, and chemical shifts observed for the relevant signals in the NMR spectra clearly indicated that the isolated products have a chalcone structure identical to that of the starting substrate. In the HMBC spectrum, there is a visible correlation between the signal from the C-4′ carbon of the chalcone backbone and H-1″ of the sugar unit, precisely indicating the attachment site of the sugar unit to the chalcone core. Additionally, in the product analyses, another compound was observed, which was also a 4′-glycoside. This additional product was characterized by signals from protons on the aromatic rings, with an identical multiplicity to the *trans*-glycoside from which it originated. However, the coupling constants for the α and β protons of this compound were significantly smaller (around 12 Hz). Furthermore, the signals from these protons were found in a lower field (lower δ value), due to the different electronic environment and spatial proximity of the substituents (double bond), which unequivocally indicates that this additional product in each of the analyzed glycoside samples is the corresponding *cis* isomer. For example, in *trans*-4′-*O*-β-D-(glucopyranosyl)-chalcone (**1a**), the H-α signal is observed at δ 7.90 (d, *J* = 15.6 Hz), and the H-β at δ 7.68 (d, *J* = 15.6 Hz). In contrast, the coupling constants for the α and β protons in the *cis* isomers are smaller; for *cis*-4′-*O*-β-D-(glucopyranosyl)-chalcone (**1b**), H-α is observed at δ 6.70 (d, *J* = 12.9 Hz), and H-β at δ 7.00 (d, *J* = 12.9 Hz). Samples containing both *trans*- and *cis*-glycosides dissolved in DMSO-*d_6_* were exposed to sunlight, and the NMR analysis was repeated. This experiment showed a clear increase in the percentage of the *cis* isomer in most of the compounds, allowing us to obtain spectral data for the *cis* isomers of the 4′-hydroxychalcone glycosides under investigation. During the isomerization of *trans*-4′-*O*-β-D-(glucopyranosyl)-3,4,5-trimethoxychalcone (**8a**), in addition to the expected *cis*-4′-*O*-β-D-(glucopyranosyl)-3,4,5-trimethoxychalcone, two other compounds were formed. However, their quantities were too low to allow for the determination of their structures or the *cis*-glycoside structure. Furthermore, during the purification of the reaction products of compound **3**, an additional product was detected alongside the expected *trans*-4′-*O*-β-D-(glucopyranosyl)-3-methoxychalcone (**3a**) and *cis*-4′-*O*-β-D-(glucopyranosyl)-3-methoxychalcone (**3b**). This product emerged during the glycoside purification process. NMR analysis revealed that this compound does not possess a double bond between the C-α and C-β carbons. In the HMBC spectrum, a coupling between the C-β carbon signal and a singlet corresponding to three protons, observed in the ^1^H-NMR spectrum at δ 3.15 ppm, was noted. This coupling, along with the positioning of the C-β carbon signal (δ 80.56), clearly indicates the presence of a methoxy substituent in this carbon. The structure of the aromatic rings, including the sugar substituent, is identical to that of compound 3a. The structure of this product has been identified as 1-(4′-O-β-D-(4″-O-methylglucopyranosyl)-phenyl)-β-methoxy-3-(3-methoxyphenyl)-propan-1-one (Table 6 and Table 7).

The enzymatic glycosylation of the 4′-hydroxychalcones explored in this study aligns with the broader research trend focusing on enhancing the bioavailability and pharmacological properties of chalcones through glycosylation. The current study demonstrates the use of eight different glucosyltransferases from diverse origins, identifying flavonoid 7-*O*-glucosyltransferase (Sbaic7OGT) from *Scutellaria baicalensis* as particularly effective for large-scale biotransformation. A key finding in this study is the observed isomerization of *trans*-chalcone glycosides to their *cis* forms during the purification process. This phenomenon has been reported in other studies, such as in the work of Simmler et al., where the isomerization of similar compounds under UV light was noted [6], suggesting that light exposure during the purification of glycosides could induce such transformations. This adds a layer of complexity to the production of chalcone glycosides, which must be managed to ensure consistency in the pharmacological properties of the end products. 

Moreover, the study’s use of *Glycine max* sucrose synthase (GmSuSy) in combination with the glucosyltransferases to enhance the UDP-glucose regeneration process reflects an advanced biotechnological approach to increase the reaction efficiency. This method has been previously validated by the work of Matera et al. [27], who also utilized a similar coupling strategy to improve the glycosylation yield of flavonoids, thereby confirming the robustness of this approach.

The potential of the newly synthesized chalcone glycosides to exhibit enhanced bioactivity is a promising avenue for drug development. Other studies have shown that the glycosylation of flavonoids can significantly enhance their solubility and stability, leading to improved pharmacokinetic properties and bioavailability [17,20,49]. Given the observed *trans–cis* isomerization, future work could focus on optimizing purification methods to minimize this conversion, ensuring that the pharmacological efficacy of the glycosides is not compromised. In line with these goals, we plan to investigate the potential impact of *trans–cis* isomerization on the biological activity of the isolated *cis*- and *trans*-chalcone glycosides. Understanding whether *cis*-glycosides offer differing therapeutic benefits compared to their *trans* counterparts will be crucial for the development of effective drugs. Moreover, steps such as conducting purification in dark or UV-filtered environments will be explored to minimize unwanted isomerization and preserve the pharmacological efficacy of the *trans*-glycosides. Additionally, the detailed bioactivity profiling of the synthesized glycosides will be crucial to fully understand their therapeutic potential. This study lays a solid foundation for further exploration into the application of glycosylated chalcones in medicinal chemistry, with the potential for developing new, more effective drugs. It is also worth highlighting the potential of utilizing photoactivation for the selective isomerization of the *trans*-isomer into its *cis* form upon its administration into the body. Such a strategy could enable the localized activation of compounds with desired biological properties, using light as a trigger for inducing isomerization. The phenomenon of photoinduced isomerization could represent an innovative approach to enhancing the selectivity and pharmacological efficacy of these compounds, particularly in light-targeted therapies.

## 3. Material and Methods

### 3.1. Chemicals and Reagents

All the chemicals and reagents used in the synthesis of the 4′-hydroxychalcones were of analytical grade and purchased from Sigma-Aldrich (St. Louis, MO, USA). All the chemicals, antibiotics, and components of the microbiological media were purchased from Sigma-Aldrich (St. Louis, MO, USA), Carbosynth (Compton, Berkshire, UK), SERVA Electrophoresis Gmb (Heidelberg, Germany), or Cayman Chemical Company (Ellsworth, ME, USA). The molecular biology reagents, including T4 DNA ligase, restriction enzymes (BsaI-HFv2, BbsIHF), Taq PCR Kit, and Plasmid Miniprep Kit were acquired from New England Biolabs Inc. (NEB, Ipswich, MA, USA). All the genes were codon-optimized and synthesized using Invitrogen GeneArt Gene Synthesis (Thermo Fisher Scientific, Waltham, MA, USA). The primers were purchased from Sigma-Aldrich (St. Louis, MO, USA). The UPLC-grade solvents were bought from Merck KGaA (Darmstadt, Germany). The purity grades of the utilized reagents and solvents were as follows: UDP-glucose (≥98%), UDP (95%), sucrose (≥99.5%), acetonitrile (≥99.9%), ethyl acetate (99%), and methanol (99%).

### 3.2. Synthesis of Trans-4′-hydroxychalcones (***1***–***8***)

**Figure 2:**
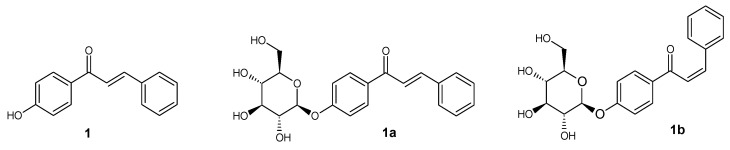
Structure of compounds **1**, **1a**, and **1b**.

**Figure 3:**
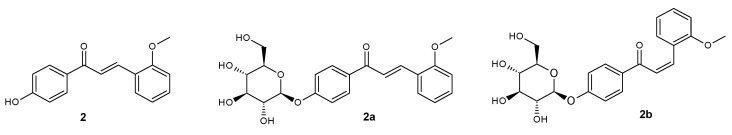
Structure of compounds **2**, **2a**, and **2b**.

**Figure 4:**
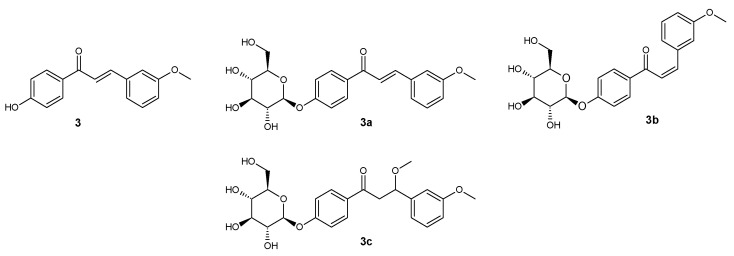
Structure of compounds **3**, **3a**, **3b**, and **3c**.

**Figure 5:**
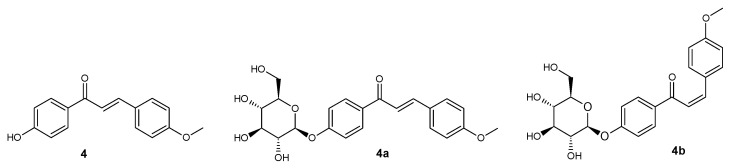
Structure of compounds **4**, **4a**, and **4b**.

**Figure 6:**
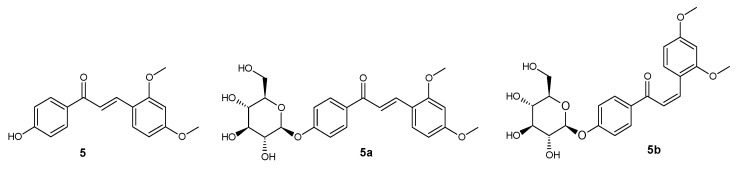
Structure of compounds **5**, **5a**, and **5b**.

**Figure 7:**
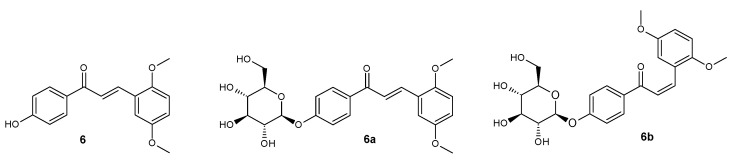
Structure of compounds **6**, **6a**, and **6b**.

**Figure 8:**
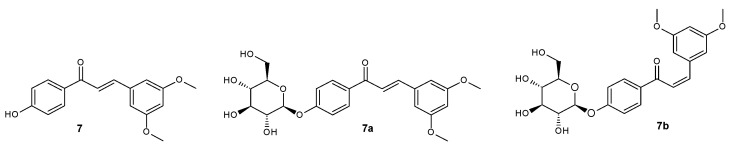
Structure of compounds **7**, **7a**, and **7b**.

**Figure 9:**
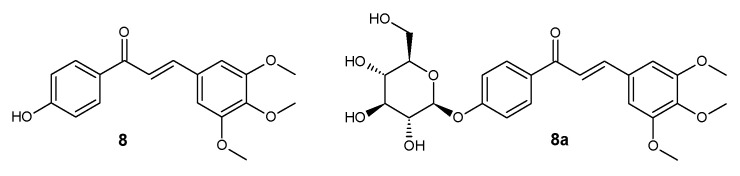
Structure of compounds **8** and **8a**.

Eight 4′-hydroxychalcones (**1**–**8**) were synthesized via Claisen–Schmidt condensation. An equimolar mixture of methoxy-substituted benzaldehyde (40 mmol) and 4′-hydroxyacetophenone (40 mmol) was dissolved in ethanol (200 mL) and NaOH (0.5 mol) was then added to the reaction mixture. The reaction was stirred under reflux conditions for 6–48 h. The progress of the reaction was monitored by thin-layer chromatography (TLC). Upon completion, the reaction mixture was poured into 500 mL of ice-cold acid (1M HCl in water), and the precipitate was filtered, washed with water, and recrystallized from ethanol to obtain pure 4′-hydroxychalcones, according to the procedure described previously [48,50]. The structure of the obtained methoxychalcone (Figure 2, Figure 3, Figure 4, Figure 5, Figure 6, Figure 7, Figure 8 and Figure 9) was confirmed by NMR analysis. The resulting compounds (**1**–**8**) were used as substrates for the biotransformation.

### 3.3. Preparation of Glycosyltransferases

Eight different glucosyltransferases from various origins (bacterial, plant, and fungal) were used. The enzymes were expressed and purified according to standard protocols [21,51]. Specifically*, E. coli* BL21 (DE3) cells were transformed with plasmids containing the genes encoding the glucosyltransferases. The protein expression was induced 25 mM of L-rhamnose at 25 °C for 16 h. The cells were harvested by centrifugation, resuspended in 50 mM Tris-HCl buffer (pH 7.5, 150 mM NaCl 10 mM imidazole), and lysed by sonication. The lysate was clarified by centrifugation, and the resulting crude lysates were used in the screening and preparative reactions; the positive reactions were verified with purified enzymes, as all the tested GTs were fused with N-termina His_6x_-tag and thus purified via Ni-NTA affinity chromatography. In case of UGT88F2, only crude lysates were used; the activity was confirmed with phloretin as a substrate.

### 3.4. Coupling with Soy Sucrose Synthase (GmSuSy)

To enhance the glycosylation efficiency, the glucosyltransferases were coupled with *Glycine max* sucrose synthase (GmSuSy). The GmSuSy was expressed and co-expressed with the GTs and purified along with or independently to the glucosyltransferases. The coupled enzyme reactions were performed in the presence of 500 mM sucrose to continuously regenerate UDP-glucose in situ. The reactions were conducted under the same conditions as the individual enzyme reactions.

### 3.5. Preparation of Protein Extract

*E. coli* BL21 (DE3) with the transformed plasmid of *Sbaic*7OGT-GmSuSy was grown overnight in 100 mL Erlenmayer flask containing a 30 mL LB medium supplemented with 20 mg/L gentamicin at 37 °C in an incubator shaker with 120 rpm agitation. After that time, 5 mL of the culture was transferred into a 2 L Erlenmayer flask containing 500 mL of dynamite medium (tryptone—12.0 g, yeast extract—24.0 g, glycerol—7.9 g, glucose—5.0 g, MgSO_4_—0.2 g, K_2_HPO_4_—6.25 g, KH_2_PO_4_—1.9 g, and NaCl—10.0 g; dispersed in 1 L of water) supplemented with 20 mg/L of gentamicin, and the culture was incubated in a baffled flask at 37 °C with 120 rpm agitation until the time that OD_600_ reached 0.6–0.8. The protein expression was then induced by adding 25 mM of L-rhamnose, and the cultivation was continued overnight at 25 °C. For the preparation of the crude extract of the enzymes, the culture was centrifuged (4000× *g*, 30 min, 4 °C; Centrifuge 5810R; Eppendorf, Hamburg, Germany), and the cell pellets were suspended in HEPES buffer (50 mM, 50 mM KCl, 10 mM MgCl_2_, pH 7.5), digested with the use of lysosym for 1 h at 4 °C, and disturbed by ice bathing with a Vibra-Cell Ultrasonic Liquid Processor. Afterwards, the cell debris was removed by centrifugation (14,000× *g*, 30 min, 4 °C; Centrifuge 5430R, Hamburg, Germany), and the resulting cell extracts containing the enzymes of interest were stored at 4 °C for the short term or at −20 °C for the long term.

### 3.6. Screening

The screening procedure was carried out in total volume of 800 μL in 2 mL Eppendorf tubes, each containing 300 μL HEPES buffer (50 mM, 50 mM KCl, 300 mM NaCl, pH 7.5), 78 μL crude extract, 20 μL UDP-Glucose (20 mM), and a 2 μL substrate (10 mM). In the case of the UGT88F2, a GgCGT-purified fraction of GmSuSy (10 μL) was added. Reactions were carried out with a thermomixer at 30 °C and 800 rpm. The reactions were carried out under the conditions and according to the procedure described earlier [27]. Samples were collected after 24 h of the biotransformation process. All the products were extracted using ethyl acetate, and the extracts were dried using anhydrous MgSO_4_, concentrated in vacuo, dissolved in methanol, filtrated, and analyzed using the TLC and UHPLC methods.

### 3.7. Preparative Scale

Preparative biotransformations were performed in 50 mL falcons, each containing 20 mL of protein extract, 16 mL of HEPES buffer, and 4 mL of substrate (~100 mmol) solution in DMSO added in four portions of 1 mL at the beginning of the reaction, after 1 h, after 2 h, and after 3 h; 0.5 mM of UDP and 500 mM of sucrose were also added. This was conducted in accordance with the optimal conditions established by Matera et al., 2024 [27] (Table 8). The products were extracted three times using ethyl acetate and then analyzed using TLC, UHPLC, and NMR spectroscopy (^1^H NMR, ^13^C NMR, COSY, HMBC, and HSQC) analysis.

### 3.8. TLC and NMR Analysis

The course of the biotransformation was monitored using TLC plates (SiO_2_, DC Alufolien Kieselgel 60 F_254_ (0.2 mm thick), Merck, Darmstadt, Germany) and UPLC. The biotransformation was evaluated by the UPLC-DAD assay performed on the Dionex Ultimate 3000 UHPLC + instrument (Thermo Fisher Scientific, Waltham, MA, USA). The UPLC system was equipped with a DGP-3600A dual pump liquid control compartment, a TCC-3200 thermostated column module, a WPS-3000 autosampler, a diode array detector (DAD), and an analytical C-18 Acclaim RSLC PolarAdvantage II (2.2 μm, 2.1 × 100 mm, Thermo Fisher Scientific, Waltham, MA, USA) column thermostated at 40 °C with two mobile phases—(A) 0.1% formic acid solution in water and (B) 0.1% formic acid solution in acetonitrile—and the following gradient elution program: 0–3 min, 15–98% B; 3–4.2 min, 98% B; 4.2–4.4 min, 98–15% B; 4.4–6 min, 15% B at 0.7 mL/min flow rate. The system control and data acquisition were managed in Chromeleon 6.80 software (Dionex, Sunnyvale, CA, USA). The identification of the substrates and products was based on authentic standard compounds (detection wavelength 280 nm), determined by their retention times and UV spectra.

The products were separated using preparative TLC plates (Silica Gel GF, 20 × 20 cm, 500 μm, Analtech) and a chloroform:methanol mixture (9:1, *v/v*) as an eluent. The product was observed (without additional visualization) under the UV lamp for the wavelength of 254 nm. During the purification process, the isomerization of the *trans*-chalcone glycosides to *cis*-chalcone glycosides was observed (the structures are presented in Figure 2, Figure 3, Figure 4, Figure 5, Figure 6, Figure 7, Figure 8 and Figure 9). This isomerization was monitored by HPLC and confirmed by NMR spectroscopy.

The NMR analysis was performed using a DRX 600 MHz Bruker spectrometer (Bruker, Billerica, MA, USA). The prepared samples were dissolved in Acetone-*d_6_* or DMSO-*d*_6_. The performed analyses included ^1^H-NMR, ^13^C-NMR, HMBC (two-dimensional analysis), HSQC (heteronuclear correlation), and COSY (correlation spectroscopy). The NMR analyses are presented in the Supplementary Materials section (Figures S1–S128, Table S1) (compounds **5**–**8, 1a**-**8a**, and **1b**-**7b**) and, for compounds **1**–**4**, in our previous publication [48].

*trans*-4′-hydroxy-chalcone (**1**) (Figure 2)

^1^H NMR (600 MHz; DMSO-*d*_6_) δ (ppm): 10.44 (s, 1H, C-4′-O*H*), 8.05–8.10 (m, 2H, H-2′ and H-6′), 7.92 (d, 1H, *J* = 15.6 Hz, H-α), 7.85–7.89 (m, 2H, H-2 and H-6), 7.68 (d, 1H, *J* = 15.6 Hz, H-β), 7.42–7.47 (m, 3H, H-3, H-4 and H-5), 6.88–6.92 (m, 2H, H-3′ and H-5′).

*trans*-4′-O-β-D-(glucopyranosyl)-chalcone (**1a**) (Figure 2)

^1^H NMR (600 MHz; Acetone-*d*_6_) δ (ppm): 8.13–8.17 (m, 2H, H-2′ and H-6′), 7.90 (d, 1H, *J* = 15.6 Hz, H-α), 7.81–7.86 (m, 2H, H-2 and H-6), 7.77 (d, 1H, *J* = 15.6Hz, H-β), 7.41–7.50 (m, 3H, H-3, H-4 and H-5), 7.17–7.22 (m, 2H, H-3′ and H-5′), 5.14 (d, 1H, *J* = 7.3 Hz, H-1″), 3.86–3.93 (m, 1H, one of H-6″), 3.68–3.75 (m, 1H, one of H-6″), 3.43–3.64 (m, 4H, H-2″, H-3″, H-4″ and H-5″).

*cis*-4′-O-β-D-(glucopyranosyl)-chalcone (**1b**) (Figure 2)

^1^H NMR (600 MHz; Acetone-*d*_6_) δ (ppm): 7.93–7.97 (m, 2H, H-2′ and H-6′), 7.42–7.47 (m, 2H, H-2 and H-6), 7.22–7.27 (m, 3H, H-3, H-4 and H-5), 7.10–7.13 (m, 2H, H-3′ and H-5′), 7.00 (d, 1H, *J* = 12.9 Hz, H-β), 6.70 (d, 1H, *J* = 12.9 Hz, H-α), 5.08 (d, 1H, *J* = 7.5 Hz, H-1″), 3.85–3.92 (m, 1H, one of H-6″), 3.66–3.74 (m, 1H, one of H-6″), 3.44–3.64 (m, 4H, H-2″, H-3″, H-4″ and H-5″).

*trans*-4′-hydroxy-2-methoxychalcone (**2**) (Figure 3)

^1^H NMR (600 MHz; DMSO-*d*_6_) δ (ppm): 10.42 (s, 1H, C-4′-O*H*), 8.02–8.06 (m, 2H, H-2′ and H-6′), 7.99 (d, 1H, *J* = 15.6 Hz, H-β), 7.94 (dd, 1H, *J* = 7.7, 1.5 Hz, H-6), 7.85 (d, 1H, *J* = 15.6 Hz, H-α), 7.43 (ddd, 1H, *J* = 8.2, 7.5, 1.6 Hz, H-4), 7.10 (d, 1H, *J* = 8.2 Hz, H-3), 7.02 (t, 1H, *J* = 7.5 Hz, H-5), 6.87–6.92 (m, 2H, H-3′ and H-5′), 3.89 (s, 3H, C-2-OC*H*_3_).

*trans*-4′-O-β-D-(glucopyranosyl)-2-methoxychalcone (**2a**) (Figure 3)

^1^H NMR (600 MHz; Acetone-*d*_6_) δ (ppm): 8.14 (d, 1H, *J* = 15.8 Hz, H-β), 8.10–8.14 (m, 2H, H-2′ and H-6′), 7.88 (dd, 1H, *J* = 7.7, 1.6 Hz, H-6), 7.86 (d, 1H, *J* = 15.8 Hz, H-α), 7.43 (ddd, 1H, *J* = 8.8, 7.5, 1.6 Hz, H-4), 7.17–7.21 (m, 2H, H-3′ and H-5′), 7.10 (d, 1H, *J* = 8.2 Hz, H-3), 7.02 (t, 1H, *J* = 7.5 Hz, H-5), 5.13 (d, 1H, *J* = 7.3 Hz, H-1″), 3.95 (s, 3H, C-2-OC*H*_3_), 3.87–3.94 (m, 1H, one of H-6″), 3.68–3.75 (m, 1H, one of H-6″), 3.44–3.63 (m, 4H, H-2”, H-3″, H-4” and H-5″).

*cis-*4′-O-β-D-(glucopyranosyl)-2-methoxychalcone (**2b**) (Figure 3)

^1^H NMR (600 MHz; Acetone-*d*_6_) δ (ppm): 7.89–7.92 (m, 2H, H-2′ and H-6′), 7.28 (dd, 1H, *J* = 7.6, 1.6 Hz, H-6), 7.21 (ddd, 1H, *J* = 8.3, 7.5, 1.6 Hz, H-4), 7.15 (d, 1H, *J* = 12.8 Hz, H-β), 7.07–7.10 (m, 2H, H-3′ and H-5′), 6.92 (dd, 1H, *J* = 8.3, 0.6 Hz, H-3), 6.76 (td, 1H, *J* = 7.5, 0.6 Hz, H-5), 6.66 (d, 1H, *J* = 12.8 Hz, H-α), 5.07 (d, 1H, *J* = 7.6 Hz, H-1″), 3.86–3.92 (m, 1H, one of H-6″), 3.77 (s, 3H, C-2-OC*H*_3_), 3.67–3.74 (m, 1H, one of H-6″), 3.43–3.62 (m, 4H, H-2″, H-3″, H-4″ and H-5″).

*trans*-4′-hydroxy-3-methoxychalcone (**3**) (Figure 4)

^1^H NMR (600 MHz; DMSO-*d*_6_) δ (ppm): 10.43 (s, 1H, C-4′-O*H*), 8.04–8.12 (m, 2H, H-2′ and H-6′), 7.92 (d, 1H, *J* = 15.6 Hz, H-α), 7.65 (d, 1H, *J* = 15.6 Hz, H-β), 7.46 (t, 1H, *J* = 1.9 Hz, H-2), 7.41 (d, 1H, *J* = 7.7 Hz, H-6), 7.46 (t, 1H, *J* = 7.8 Hz, H-5), 7.86 (ddd, 1H, *J* = 8.0, 2.4, 0.8 Hz, H-4), 6.88–6.92 (m, 2H, H-3′ and H-5′), 3.83 (s, 3H, C-3-OC*H*_3_).

*trans*-4′-O-β-D-(glucopyranosyl)-3-methoxychalcone (**3a**) (Figure 4)

^1^H NMR (600 MHz; Acetone-*d*_6_) δ (ppm): 8.12–8.17 (m, 2H, H-2′ and H-6′), 7.89 (d, 1H, *J* = 15.6 Hz, H-α), 7.74 (d, 1H, *J* = 15.6 Hz, H-β), 7.41 (t, 1H, *J* = 1.9 Hz, H-2), 7.39 (dt, 1H, *J* = 7.6, 1.4 Hz, H-6), 7.36 (t, 1H, *J* = 7.8 Hz, H-5), 7.01 (ddd, 1H, *J* = 7.7, 2.5, 1.5 Hz, H-4), 7.17–7.22 (m, 2H, H-3′ and H-5′), 5.13 (d, 1H, *J* = 7.3 Hz, H-1″), 3.88–3.94 (m, 1H, one of H-6″), 3.86 (s, 3H, C-3-OC*H*_3_), 3.68–3.75 (m, 1H, one of H-6″), 3.44–3.63 (m, 4H, H-2″, H-3″, H-4″ and H-5″).

*cis*-4′-O-β-D-(glucopyranosyl)-3-methoxychalcone (**3b**) (Figure 4)

^1^H NMR (600 MHz; Acetone-*d*_6_) δ (ppm): 7.94–7.97 (m, 2H, H- and H-6′), 7.16 (t, 1H, *J* = 7.9 Hz, H-5), 7.13–7.10 (m, 2H, H-3′ and H-5′), 7.03 (dd, 1H, *J* = 2.2, 1.8 Hz, H-2), 6.97 (dm, 1H, *J* = 7.9 Hz, H-6), 6.96 (d, 1H, *J* = 13.0 Hz, H-β), 6.81 (ddd, 1H, *J* = 8.1, 2.6, 0.6 Hz, H-4), 6.66 (d, 1H, *J* = 13.0 Hz, H-α), 5.08 (d, 1H, *J* = 7.5 Hz, H-1″), 3.86–3.91 (m, 1H, one of H-6″), 3.67–3.73 (m, 1H, one of H-6″), 3.68 (s, 3H, C-3-OC*H*_3_), 3.44–3.61 (m, 4H, H-2″, H-3″, H-4″ and H-5″).

1-(4ʹ-O-β-D-(4″-O-methylglucopiranosyl)-phenyl)-β-metoxy-3-(-3-metoxyphenyl)-propan-1-on (**3c**) (Figure 4)

^1^H NMR (600 MHz; Acetone-*d*_6_) δ (ppm): 7.96–7.99 (m, 2H, H-2′ and H-6′), 7.28 (t, 1H, *J* = 7.8 Hz, H-5), 7.12–7.15 (m, 2H, H-3′ and H-5′), 7.01 (ddd, 1H, *J* = 7.7, 2.5, 1.5 Hz, H-6), 7.00 (dd, 1H, *J* = 2.2, 1.8 Hz, H-2), 6.81 (ddd, 1H, *J* = 8.2, 2.5, 1.0 Hz, H-4), 5.10 (d, 1H, *J* = 7.5 Hz, H-1″), 4.80 (d, 1H, *J* = 8.6, 4.3 Hz, H-β), 3.86–3.91 (m, 1H, one of H-6″), 3.80 (s, 3H, C-3-OC*H*_3_), 3.67–3.73 (m, 1H, one of H-6″), 3.44–3.61 (m, 4H, H-2″, H-3″, H-4″ and H-5″), 3.58–3.62 (m, 1H, one of H-α), 3.15 (s, 3H, C-β-OC*H*_3_), 3.03 (dd, 1H, *J* = 16.3, 4.3 Hz, one of H-α).

*trans*-4′-hydroxy-4-methoxychalcone (**4**) (Figure 5)

^1^H NMR (600 MHz; DMSO-*d*_6_) δ (ppm): 10.41 (s, 1H, C-4′-O*H*), 8.03–8.06 (m, 2H, H-2′ and H-6′), 7.79–7.83 (m, 2H, H-2 and H-6), 7.76 (d, 1H, *J* = 15.5 Hz, H-α), 7.65 (d, 1H, *J* = 15.5 Hz, H-β), 6.98–7.02 (m, 2H, H-3 and H-5), 6.87–6.91 (m, 2H, H-3′ and H-5′), 3.81 (s, 3H, C-4-OC*H*_3_).

*trans*-4′-O-β-D-(glucopyranosyl)-4-methoxychalcone (**4a**) (Figure 5)

^1^H NMR (600 MHz; Acetone-*d*_6_) δ (ppm): 8.11–8.14 (m, 2H, H-2′ and H-6′), 7.78–7.81 (m, 2H, H-2 and H-6), 7.76 (d, 1H, *J* = 15.5 Hz, H-β), 7.73 (d, 1H, *J* = 15.5 Hz, H-α), 7.17–7.20 (m, 2H, H-3 and H-5), 7.00–7.03 (m, 2H, H-3ʹ and H-5ʹ), 5.12 (d, 1H, *J* = 7.4 Hz, H-1″), 3.88–3.92 (m, 1H, one of H-6″), 3.87 (s, 3H, C-4-OC*H*_3_) 3.69–3.76 (m, 1H, one of H-6″), 3.44–3.61 (m, 4H, H-2″, H-3″, H-4″ and H-5″).

*cis-*4′-O-β-D-(glucopyranosyl)-4-methoxychalcone (**4b**) (Figure 5)

^1^H NMR (600 MHz; Acetone-*d*_6_) δ (ppm): 77.95–7.99 (m, 2H, H-2′ and H-6′), 7.52–7.57 (m, 2H, H-2 and H-6), 7.10–7.14 (m, 2H, H-3′ and H-5′), 6.93 (d, 1H, *J* = 12.9 Hz, H-β), 6.82–6.85 (m, 2H, H-3 and H-5), 6.63 (d, 1H, *J* = 12.9 Hz, H-α), 5.08 (d, 1H, *J* = 7.5 Hz, H-1″), 3.86–3.92 (m, 1H, one of H-6″), 3.78 (s, 3H, C-4-OC*H*_3_), 3.67–3.74 (m, 1H, one of H-6″), 3.44–3.61 (m, 4H, H-2″, H-3″, H-4″ and H-5″).

*trans*-4′-hydroxy-2,4-dimethoxychalcone (**5**) (Figure 6)

^1^H NMR (600 MHz; DMSO-*d*_6_) δ (ppm): 10.36 (s, 1H, C-4′-O*H*), 7.98–8.04 (m, 2H, H-2′ and H-6′), 7.93 (d, 1H, *J* = 15.7 Hz, H-β), 7.89 (d, 1H, *J* = 8.5 Hz, H-6), 7.73 (d, 1H, *J* = 15.7 Hz, H-α), 6.86–6.91 (m, 2H, H-3′ and H-5′), 6.63 (d, 1H, *J* = 2.3 Hz, H-3), 6.61 (dd, 1H, *J* = 8.6, 2.3 Hz, H-5), 3.89 (s, 3H, C-2-OC*H*_3_), 3.83 (s, 3H, C-4-OC*H*_3_).

*trans*-4′-O-β-D-(glucopyranosyl)-2,4-dimethoxychalcone (**5a**) (Figure 6)

^1^H NMR (600 MHz; Acetone-*d*_6_) δ (ppm): 8.07–8.11 (m, 2H, H-2′ and H-6′), 8.07 (d, 1H, *J* = 15.7 Hz, H-β), 7.81 (d, 1H, *J* = 8.6 Hz, H-6), 7.74 (d, 1H, *J* = 15.7 Hz, H-α), 7.16–7.20 (m, 2H, H-3′ and H-5′), 6.64 (d, 1H, *J* = 2.3 Hz, H-3), 6.61 (dd, 1H, *J* = 8.5, 2.3 Hz, H-5), 5.12 (d, 1H, *J* = 7.3 Hz, H-1″), 3.95 (s, 3H, C-2-OC*H*_3_), 3.91 (dd, 1H, *J* = 11.8, 2.8 Hz, one of H-6″), 3.87 (s, 3H, C-4-OC*H*_3_), 3.72 (m, 1H, *J* = 11.8, 5.5 Hz, one of H-6″), 3.45–3.62 (m, 4H, H-2″, H-3″, H-4″ and H-5″).

*cis*-4′-O-β-D-(glucopyranosyl)-2,4-dimethoxychalcone (**5b**) (Figure 6)

^1^H NMR (600 MHz; Acetone-*d*_6_) δ (ppm): 7.89–7.94 (m, 2H, H-2′ and H-6′), 7.44 (d, 1H, *J* = 8.6 Hz, H-6), 7.12 (d, 1H, *J* = 12.9 Hz, H-β), 7.07–7.11 (m, 2H, H-3′ and H-5′), 6.57 (d, 1H, *J* = 12.9 Hz, H-α), 6.48 (d, 1H, *J* = 2.4 Hz, H-3), 6.36 (dd, 1H, *J* = 8.6, 2.4 Hz, H-5), 5.07 (d, 1H, *J* = 7.3 Hz, H-1″), 3.88–3.94 (m, 1H, one of H-6″), 3.78 (s, 3H, C-4-OC*H*_3_), 3.76 (s, 3H, C-2-OC*H*_3_), 3.68–3.75 (m, 1H, one of H-6″), 3.43–3.62 (m, 4H, H-2″, H-3″, H-4″ and H-5″). 

*trans*-4′-hydroxy-2,5-dimethoxychalcone (**6**) (Figure 7)

^1^H NMR (600 MHz; DMSO-*d*_6_) δ (ppm): 10.42 (s, 1H, C-4′-O*H*), 8.04–8.09 (m, 2H, H-2′ and H-6′), 7.98 (d, 1H, *J* = 15.7 Hz, H-β), 7.87 (d, 1H, *J* = 15.7 Hz, H-α), 7.53 (d, 1H, *J* = 2.5 Hz, H-6), 7.04 (d, 1H, *J* = 9.0 Hz, H-3), 7.04 (dd, 1H, *J* = 9.0, 2.5 Hz, H-4), 6.87–6.92 (m, 2H, H-3′ and H-5′), 3.83 (s, 3H, C-2-OC*H*_3_), 3.79 (s, 3H, C-5-OC*H*_3_). 

*trans*-4′-O-β-D-(glucopyranosyl)-2,5-dimethoxychalcone (**6a**) (Figure 7)

^1^H NMR (600 MHz; Acetone-*d*_6_) δ (ppm): 8.11–8.15 (m, 2H, H-2′ and H-6′), 8.12 (d, 1H, *J* = 15.7 Hz, H-β), 7.87 (d, 1H, *J* = 15.7 Hz, H-α), 7.46 (d, 1H, *J* = 2.8 Hz, H-6), 7.17–7.21 (m, 2H, H-3′ and H-5′), 7.04 (d, 1H, *J* = 8.9 Hz, H-3), 7.01 (dd, 1H, *J* = 8.9, 2.8 Hz, H-4), 5.12 (d, 1H, *J* = 7.3 Hz, H-1″), 3.86–3.94 (m, 1H, one of H-6″), 3.90 (s, 3H, C-2-OC*H*_3_), 3.82 (s, 3H, C-5-OC*H*_3_), 3.67–3.75 (m, 1H, one of H-6″), 3.44–3.62 (m, 4H, H-2″, H-3″, H-4″ and H-5″).

*cis*-4′-*O*-β-D-(glucopyranosyl)-2,5-dimethoxychalcone (**6b**) (Figure 7)

^1^H NMR (600 MHz; Acetone-*d*_6_) δ (ppm): 7.90–7.94 (m, 2H, H-2′ and H-6′), 7.14 (d, 1H, *J* = 12.9 Hz, H-β), 7.07–7.11 (m, 2H, H-3′ and H-5′), 6.93 (dd, 1H, *J* = 3.0 Hz, H-6), 6.86 (d, 1H, *J* = 8.9 Hz, H-3), 6.78 (dd, 1H, *J* = 8.9, 3.0 Hz, H-4), 6.66 (d, 1H, *J* = 12.9 Hz, H-α), 5.07 (d, 1H, *J* = 7.3 Hz, H-1″), 3.86–3.94 (m, 1H, one of H-6″), 3.73 (s, 3H, C-2-OC*H*_3_), 3.67–3.74 (m, 1H, one of H-6″), 3.59 (s, 3H, C-5-OC*H*_3_), 3.43–3.62 (m, 4H, H-2″, H-3″, H-4″ and H-5″). 

*trans*-4′-hydroxy-3,5-dimethoxychalcone (**7**) (Figure 8)

^1^H NMR (600 MHz; DMSO-*d*_6_) δ (ppm): 10.45 (s, 1H, C-4′-O*H*), 8.07–8.11 (m, 2H, H-2′ and H-6′), 7.91 (d, 1H, *J* = 15.6 Hz, H-α), 7.60 (d, 1H, *J* = 15.6 Hz, H-β), 7.05 (d, 2H, *J* = 2.2 Hz, H-2 and H-6), 6.87–6.92 (m, 2H, H-3′ and H-5′), 6.57 (d, 1H, *J* = 2.2 Hz, H-4), 3.80 (s, 6H, C-3-OC*H*_3_ and C-5-OC*H*_3_). 

*trans*-4′-*O*-β-D-(glucopyranosyl)-3,5-dimethoxychalcone (**7a**) (Figure 8)

^1^H NMR (600 MHz; Acetone-*d*_6_) δ (ppm): 8.12–8.15 (m, 2H, H-2′ and H-6′), 7.88 (d, 1H, *J* = 15.6 Hz, H-α), 7.69 (d, 1H, *J* = 15.6 Hz, H-β), 7.17–7.21 (m, 2H, H-3′ and H-5′), 7.01 (d, 2H, *J* = 2.2 Hz, H-2 and H-6), 6.57 (d, 1H, *J* = 2.2 Hz, H-4), 5.13 (d, 1H, *J* = 7.4 Hz, H-1″), 3.85–3.93 (m, 1H, one of H-6″), 3.85 (s, 6H, C-3-OC*H*_3_ and C-5-OC*H*_3_), 3.67–3.76 (m, 1H, one of H-6″), 3.43–3.61 (m, 4H, H-2″, H-3″, H-4″ and H-5″).

*cis*-4′-*O*-β-D-(glucopyranosyl)-3,5-dimethoxychalcone (**7b**) (Figure 8)

^1^H NMR (600 MHz; Acetone-*d*_6_) δ (ppm): 7.94–7.97 (m, 2H, H-2′ and H-6′), 7.10–7.13 (m, 2H, H-3′ and H-5′), 6.92 (d, 1H, *J* = 13.0 Hz, H-β), 6.62 (d, 1H, *J* = 13.0 Hz, H-α), 6.58 (d, 2H, *J* = 2.2 Hz, H-2 and H-6), 6.36 (d, 1H, *J* = 2.2 Hz, H-4), 5.08 (d, 1H, *J* = 7.5 Hz, H-1″), 3.86–3.93 (m, 1H, one of H-6″), 3.67–3.75 (m, 1H, one of H-6″), 3.67 (s, 6H, C-3-OC*H*_3_ and C-5-OC*H*_3_), 3.43–3.61 (m, 4H, H-2″, H-3″, H-4″ and H-5″).

*trans*-4′-hydroxy-3,4,5-trimethoxychalcone (**8**) (Figure 9)

^1^H NMR (600 MHz; DMSO-*d*_6_) δ (ppm): 10.42 (s, 1H, C-4′-O*H*), 8.07–8.11 (m, 2H, H-2′ and H-6′), 7.86 (d, 1H, *J* = 15.5 Hz, H-α), 7.64 (d, 1H, *J* = 15.5 Hz, H-β), 7.21 (s, 2H, H-2 and H-6), 6.88–6.93 (m, 2H, H-3′ and H-5′), 3.86 (s, 6H, C-3-OC*H*_3_ and C-5-OC*H*_3_), 3.71 (s, 3H, C-4-OC*H*_3_).

*trans*-4′-*O*-β-D-(glucopyranosyl)- 3,4,5-trimethoxychalcone (**8a**) (Figure 9)

^1^H NMR (600 MHz; Acetone-*d*_6_) δ (ppm): 8.09–8.13 (m, 2H, H-2′ and H-6′), 7.83 (d, 1H, *J* = 15.5 Hz, H-α), 7.71 (d, 1H, *J* = 15.5 Hz, H-β), 7.17–7.21 (m, 2H, H-3′ and H-5′), 7.18 (s, 2H, H-2 and H-6), 5.12 (d, 1H, *J* = 7.4 Hz, H-1″), 3.86–3.94 (m, 1H, one of H-6″), 3.91 (s, 6H, C-3-OC*H*_3_ and C-5-OC*H*_3_), 3.78 (s, 3H, C-4-OC*H*_3_), 3.67–3.73 (m, 1H, one of H-6″), 3.57–3.61 (m, 1H, H-5″), 3.44–3.56 (m, 3H, H-2″, H-3″ and H-4″).

## 4. Conclusions

This study successfully explored the enzymatic glycosylation of 4′-hydroxychalcones using a diverse set of glucosyltransferases from bacterial, plant, and fungal origins. The research demonstrated that enzymatic biotransformation is a viable method for producing glycosylated 4′-hydroxychalcones. Among the tested enzymes, flavonoid 7-*O*-glucosyltransferase from *Scutellaria baicalensis* (*Sbaic*7OGT) was selected for large-scale biotransformation.

The enzymatic glycosylation approach demonstrated here highlights the potential for generating bioactive glycosides with improved pharmaceutical properties. The observed *trans–cis* isomerization introduces an additional layer of complexity and potential variability in biological activity, which warrants further investigation. Future work will focus on the detailed bioactivity profiling of these glycosides, including their antioxidant, anti-inflammatory, and antimicrobial properties. In conclusion, the successful glycosylation of 4′-hydroxychalcones via enzymatic methods opens new avenues for the development of novel glycoconjugates with potential therapeutic applications. The ability to selectively modify chalcones through glycosylation enhances their utility in drug development and warrants the further exploration of this biotransformation strategy. Future research could also focus on exploring the potential for the controlled photoactivation of the pre-administration drug *trans*-isomer to the drug’s *cis* form post-administration, which could open new avenues in therapeutic applications. This technique may increase pharmacological selectivity by using light activation within target tissues. 

## Figures and Tables

**Table 1:** Glucosides of *trans*-chalcones obtained via biotransformations and glucosides of *cis-*chalcones identified as a result of UV-isomerization.

*trans*-chalcone	*trans*-4′-glycoside	*cis*-4′-glycoside	R^1^	R^2^	R^3^	R^3^
**1**	**1a**	**1b**	-H	-H	-H	-H
**2**	**2a**	**2b**	-OCH_3_	-H	-H	-H
**3**	**3a**	**3b**	-H	-OCH_3_	-H	-H
**4**	**4a**	**4b**	-H	-H	-H	-OCH_3_
**5**	**5a**	**5b**	-OCH_3_	-H	-OCH_3_	-H
**6**	**6a**	**6b**	-OCH_3_	-H	-H	-OCH_3_
**7**	**7a**	**7b**	-H	-OCH_3_	-H	-OCH_3_
**8**	**8a**	**8b**	-H	-OCH_3_	-OCH_3_	-OCH_3_

**Table 2:** Enzymatic relative activity (%) of GTs on 4′-hydroxychalcones.

Substrate	Enzyme							
	YjiC	*Sbaic*7OGT	Bet5OGT	BbGT278	OleD	UGT88F2	UGT85A2	GgCGT
**1**	63.8	80.7	86.8	69.7	53.5	0.0	0.0	3.3
**2**	48.8	73.7	77.9	33.1	9.1	0.0	0.0	0.0
**3**	61.4	78.4	87.1	50.8	56.0	0.0	0.0	3.2
**4**	59.2	73.4	85.3	75.2	61.7	0.0	0.0	3.4
**5**	55.0	70.0	82.7	54.6	55.1	0.0	0.0	1.1
**6**	60.1	85.2	76.8	38.1	62.8	0.0	0.0	2.7
**7**	55.1	75.0	87.9	12.2	54.6	0.5	0.0	2.1
**8**	27.3	35.5	26.5	0.9	6.9	0.0	0.0	0.0

**Table 3:** Composition of reaction mixture based on protein extract dilution.

Composition	Variant			
	a	b	c	d
Crude Enzyme Extract (μL)	712	400	200	100
UDP (50 mM) (μL)	8	8	8	8
Substrate (200 mM) (μL)	80	80	80	80
Buffer (μL)	0	312	512	612

**Table 4:** Biotransformation rate of compound **1** into **1a** in accordance with chosen reaction variant.

Time	Variant			
	a	b	c	d
1 h	87.3 ± 0.4	93.1 ± 3.3	76.6 ± 9.8	45.9 ± 7.4
2 h	68.6 ± 0.4	64.2 ± 1.9	43.6 ± 6.3	30.4 ± 3.2
3 h	58.5 ± 0.2	46.1 ± 1.2	32.5 ± 3.0	21.0 ± 5.0
4 h	53.8 ± 1.2	43.9 ± 0.7	31.1 ± 5.2	24.5 ± 6.0
6 h	56.0 ± 3.8	42.2 ± 2.0	31.1 ± 2.4	22.5 ± 2.9
18 h	73.5 ± 2.0	45.1 ± 2.4	38.4 ± 2.3	26.7 ± 5.9
24 h	70.5 ± 4.5	47.1 ± 1.2	39.9 ± 3.5	29.6 ± 5.4

**Table 5:** Percentage of products **1a**–**8a** formed in reaction in the variant **b** mixture over time.

Compound	1 h	2 h	4 h	24 h
**1a**	27.6	27.0	24.6	30.0
**2a**	32.5	35.2	25.7	30.2
**3a**	27.1	27.0	30.7	29.9
**4a**	25.4	22.3	30.9	36.0
**5a**	38.1	30.0	25.5	30.0
**6a**	23.0	23.1	22.9	30.2
**7a**	26.5	25.3	25.9	36.2
**8a**	39.7	24.0	17.9	36.4

**Table 6:** Summary of signals visible in the ^13^C NMR spectrum of the substrates (**1**–**4**) and the biotransformation (**1a**–**4a**) and photoisomerization (**1b**–**4b**) products.

carbon	1	1a	1b	2	2a	2b	3	3a	3b	3c	4	4a	4b
C=O	187.14	188.23	193.66	187.31	188.54	193.27	187.15	188.25	193.96	196.29	187.18	188.15	193.03
α	122.11	122.68	128.28	121.91	122.70	127.86	122.37	122.88	128.60	47.11	119.64	120.27	125.12
β	142.76	144.16	138.16	137.29	139.19	134.21	142.75	144.19	137.65	80.56	142.80	144.10	139.10
1	134.91	136.08	136.54	123.16	124.55	125.57	136.32	137.45	137.79	144.40	127.58	128.73	129.12
2	128.92	129.40	130.10	158.14	159.54	157.98	113.20	113.91	115.17	112.95	130.64	131.21	132.37
3	128.74	129.74	129.00	111.78	112.29	111.45	159.66	161.03	160.31	160.81	115.41	115.20	114.35
4	130.35	131.12	129.35	132.01	132.66	130.86	116.45	117.17	115.11	113.82	161.20	162.60	161.20
5	128.74	129.74	129.00	120.71	121.50	120.70	129.92	130.71	130.03	130.24	115.41	115.20	114.35
6	128.92	129.40	130.10	129.26	129.35	131.01	121.55	122.10	122.58	119.69	130.64	131.21	132.37
1′	129.12	132.98	132.07	128.37	133.20	132.33	129.10	132..96	132.70	132.40	129.41	133.33	132.62
2′.6′	131.24	131.38	131.70	131.01	131.28	131.50	131.27	131.39	131.70	131.04	131.12	131.23	131.58
3′.5′	115.41	116.94	116.94	115.42	116.92	116.73	115.40	116.93	116.92	116.75	114.46	116.92	116.92
4′	162.25	162.33	162.56	162.14	162.20	162.25	162.25	162.32	162.56	162.27	162.07	162.21	162.44
1″	-	101.22	101.17	-	101.21	101.13	-	101.20	101.12	101.18	-	101.31	101.24
2″	-	74.54	74.50	-	74.53	74.50	-	74.52	74.51	74.48	-	74.61	74.55
3″	-	77.85	77.88	-	77.82	77.81	-	77.82	77.78	77.80	-	77.92	77.86
4″	-	71.15	71.08	-	71.12	71.10	-	71.13	71.05	71.10	-	71.24	71.16
5″	-	77.94	77.94	-	77.90	77.87	-	77.92	77.92	77.85	-	77.99	77.92
6″	-	62.50	62.43	-	62.49	62.45	-	62.49	62.41	62.46	-	62.57	62.49
C-2-O*C*H_3_	-	-	-	55.72	56.01	55.62	-	-	-	-	-	-	-
C-3-O*C*H_3_	-	-	-	-	-	-	55.31	55.63	55.33	55.39	-	-	-
C-4-O*C*H_3_	-	-	-	-	-	-	-	-	-	-	55.43	55.76	55.54
β-O*C*H_3_	-	-	-	-	-	-	-	-	-	56.81	-	-	-

**Table 7:** Summary of signals visible in the ^13^C NMR spectrum of the substrates (**5**–**8**) and the biotransformation (**5a**–**8a**) and photoisomerization (**5b**–**7b**) products.

carbon	5	5a	5b	6	6a	6b	7	7a	7b	8	8a
=O	187.25	188.49	192.96	187.26	188.46	193.46	187.16	188.29	194.28	187.08	188.21
α	119.14	120.02	125.05	122.09	122.85	128.16	122.58	123.13	128.96	121.27	121.81
β	137.52	139.40	134.37	137.03	138.97	133.69	142.90	144.35	137.34	143.25	144.64
1	116.13	117.51	118.18	123.72	125.13	122.85	136.86	138.02	138.32	130.48	133.13
2	159.80	161.16	159.55	152.59	153.98	152.33	106.58	107.18	108.04	106.34	107.06
3	98.32	98.93	98.52	113.01	113.54	112.57	160.73	162.16	161.52	153.12	154.60
4	162.85	164.16	162.76	117.82	118.37	115.85	102.64	103.39	101.41	139.47	141.36
5	106.28	106.85	105.36	153.28	154.64	153.81	160.73	162.16	161.52	153.12	154.60
6	129.93	130.92	132.28	112.47	113.51	116.40	106.64	107.18	108.04	106.34	107.06
1′	129.54	133.52	132.74	129.23	133.19	132.32	129.08	133.00	132.03	129.22	131.54
2′.6′	130.93	131.11	131.43	131.20	131.32	131.55	131.31	131.41	131.72	131.22	131.31
3′.5′	115.34	116.86	116.73	115.38	116.91	116.80	115.39	116.97	116.97	115.36	116.93
4′	161.90	162.01	162.14	162.16	162.25	162.34	162.27	162.37	162.62	162.17	162.29
1″	-	101.23	101.16	-	101.25	101.16	-	101.27	101.19	-	101.28
2″	-	74.54	74.50	-	74.55	74.52	-	74.56	74.53	-	74.56
3″	-	77.82	77.79	-	77.81	77.87	-	77.90	77.84	-	77.90
4″	-	71.13	71.08	-	71.16	71.09	-	71.20	71.12	-	71.22
5″	-	77.89	77.89	-	77.87	77.95	-	77.97	77.90	-	77.99
6″	-	62.49	62.44	-	62.51	62.45	-	62.54	62.46	-	62.56
C-2-O*C*H_3_	55.83	56.07	55.73	56.15	56.48	56.01	-	-	-	-	
C-3-O*C*H_3_	-	-	-	-	-	-	55.46	55.79	55.48	56.13	56.53
C-4-O*C*H_3_	55.54	55.84	55.60	-	-	-	-	-	-	60.15	60.62
C-5-O*C*H_3_	-	-	-	55.70	56.01	55.67	55.46	55.79	55.48	56.13	56.53

**Table 8:** Retention times based on UPLC and isolated yield of obtained glycosides.

Retention Time [min]						Isolated Yield [%] of Glycoside
*trans*-chalcone		*trans*-4′-glycoside		*cis*-4′-glycoside		
**1**	3.534	**1a**	2.714	**1b**	2.551	12.6%
**2**	3.574	**2a**	2.779	**2b**	2.575	11.6%
**3**	3.555	**3a**	2.775	**3b**	2.583	21.0%
**4**	3.572	**4a**	2.731	**4b**	2.619	19.7%
**5**	3.367	**5a**	2.803	**5a**	2.657	20.0%
**6**	3.563	**6a**	2.811	**6a**	2.588	15.3%
**7**	3.591	**7a**	2.843	**7a**	2.639	9.3%
**8**	3.371	**8a**	2.664	**8a**	2.473	2.7%

## Data Availability

Data are contained within the article and Supplementary Materials.

## Supplementary Materials

The following supporting information can be downloaded at: https://www.mdpi.com/article/10.3390/ijms252111482/s1.
