# Supplementary material for: Enzymatic Glycosylation of 4′-Hydroxychalcones: Expanding the Scope of Nature’s Catalytic Potential
Source: Int J Mol Sci. 2024 Oct 25;25(21):11482. doi: 10.3390/ijms252111482 (PMC11546794; doi:10.3390/ijms252111482)
Supplement: Supplementary file 1 [file ijms-25-11482-s001.zip › ijms-3223190-supplementary.pdf]

**Supplementary Data**

# Enzymatic Glycosylation of 4′-Hydroxychalcones: Expanding the Scope of Nature's Catalytic Potential

**Paweł Chlipała *, Agata Matera, Sandra Sordon, Jarosław Popłoński, Marcelina Mazur, Tomasz Janeczko***

Department of Food Chemistry and Biocatalysis, Faculty of Biotechnology and Food Science, Wrocław University of Environmental and Life Sciences, 50-375 Wrocław, Poland. 125375@student.upwr.edu.pl (J.B.); agata.matera@upwr.edu.pl (A.M.); sandra.sordon@upwr.edu.pl (S.S.); jaroslaw.poplonski@upwr.edu.pl (J.P.); marcelina.mazur@upwr.edu.pl (M.M.)
Correspondence: pawel.chlipala@upwr.edu.pl (P.C.); tomasz.janeczko@upwr.edu.pl (T.J.)

**Contents:**

**Figure S1** Chromatogram of reaction mixture of trans-4′-hydroxychalcone (**1**) and trans-4′-O-β-D-(glucopyranosyl)-chalcone (**1a**) before product purification. 5
**Figure S2** Chromatogram of mixture of trans-4′-O-β-D-(glucopyranosyl)-chalcone (**1a**) and cis-4′-O-β-D-(glucopyranosyl)-chalcone (**1b**) after NMR analysis. 5
**Figure S3** UV-VIS spectra of *trans*-4′-hydroxychalcone (**1**) *trans*-4′-*O*-β-D-(glucopyranosyl)-chalcone (**1a**) and *cis*-4′-*O*-β-D-(glucopyranosyl)-chalcone (**1b**) 5
**Figure S4** Chromatogram of reaction mixture of trans-4′-hydroxy-2-methoxychalcone (**2**) and trans-4′-O-β-D-(glucopyranosyl)-2-methoxychalcone (**2a**) before product purification. 6
**Figure S5** Chromatogram of mixture of trans-4′-O-β-D-(glucopyranosyl)-2-methoxychalcone (**2a**) and cis-4′-O-β-D-(glucopyranosyl)-2-methoxychalcone (**2b**) after NMR analysis. 6
**Figure S6** UV-VIS spectra of *trans*-4′-hydroxy-2-methoxychalcone (**2**) *trans*-4′-*O*-β-D-(glucopyranosyl)-2-methoxychalcone (**2a**) and *cis*-4′-*O*-β-D-(glucopyranosyl)-2-methoxychalcone (**2b**). 6
**Figure S7** Chromatogram of reaction mixture of trans-4′-hydroxy-3-methoxychalcone (**3**) and trans-4′-O-β-D-(glucopyranosyl)-3-methoxychalcone (**3a**) before product purification. 7
**Figure S8** Chromatogram of mixture of trans-4′-O-β-D-(glucopyranosyl)-3-methoxychalcone (**3a**) and cis-4′-O-β-D-(glucopyranosyl)-3-methoxychalcone (**3b**) after NMR analysis. 7
**Figure S9** UV-VIS spectra of *trans*-4′-hydroxy-3-methoxychalcone (**3**) *trans*-4′-*O*-β-D-(glucopyranosyl)-3-methoxychalcone (**3a**) and *cis*-4′-*O*-β-D-(glucopyranosyl)-3-methoxychalcone (**3b**). 7
**Figure S10** Chromatogram of reaction mixture of trans-4′-hydroxy-4-methoxychalcone (**4**) and trans-4′-O-β-D-(glucopyranosyl)-4-methoxychalcone (**4a**) before product purification. 8
**Figure S11** Chromatogram of mixture of trans-4′-O-β-D-(glucopyranosyl)-4-methoxychalcone (**4a**) and cis-4′-O-β-D-(glucopyranosyl)-4-methoxychalcone (**4b**) after NMR analysis. 8
**Figure S12** UV-VIS spectra of *trans*-4′-hydroxy-4-methoxychalcone (**4**) *trans*-4′-*O*-β-D-(glucopyranosyl)-4-methoxychalcone (**4a**) and *cis*-4′-*O*-β-D-(glucopyranosyl)-4-methoxychalcone (**4b**). 8
**Figure S13** Chromatogram of reaction mixture of trans-4′-hydroxy-2,4-dimethoxychalcone (**5**) and trans-4′-O-β-D-(glucopyranosyl)-2,4-dimethoxychalcone (**5a**) before product purification. 9
**Figure S14** Chromatogram of mixture of trans-4′-O-β-D-(glucopyranosyl)-2,4-dimethoxychalcone (**5a**) and cis-4′-O-β-D-(glucopyranosyl)-2,4-dimethoxychalcone (**5b**) after NMR analysis. 9
**Figure S15** UV-VIS spectra of *trans*-4′-hydroxy-2,4-dimethoxychalcone (**5**) *trans*-4′-*O*-β-D-(glucopyranosyl)-2,4-dimethoxychalcone (**5a**) and *cis*-4′-*O*-β-D-(glucopyranosyl)-2,4-dimethoxychalcone (**5b**). 9
**Figure S16** Chromatogram of reaction mixture of trans-4′-hydroxy-2,5-dimethoxychalcone (**6**) and trans-4′-O-β-D-(glucopyranosyl)-2,5-dimethoxychalcone (**6a**) before product purification. 10
**Figure S17** Chromatogram of mixture of trans-4′-O-β-D-(glucopyranosyl)-2,5-dimethoxychalcone (**6a**) and cis-4′-O-β-D-(glucopyranosyl)-2,5-dimethoxychalcone (**6b**) after NMR analysis. 10
**Figure S18** UV-VIS spectra of *trans*-4′-hydroxy-2,5-dimethoxychalcone (**6**) *trans*-4′-*O*-β-D-(glucopyranosyl)-2,5-dimethoxychalcone (**6a**) and *cis*-4′-*O*-β-D-(glucopyranosyl)-2,5-dimethoxychalcone (**6b**). 10
**Figure S19** Chromatogram of reaction mixture of trans-4′-hydroxy-3,5-dimethoxychalcone (**7**) and trans-4′-O-β-D-(glucopyranosyl)-3,5-dimethoxychalcone (**7a**) before product purification. 11

**Figure S20** Chromatogram of mixture of trans-4′-O-β-D-(glucopyranosyl)-3,5-dimethoxychalcone (**7a**) and cis-4′-O-β-D-(glucopyranosyl)-3,5-dimethoxychalcone (**7b**) after NMR analysis. 11
**Figure S21** UV-VIS spectra of *trans*-4′-hydroxy-3,5-dimethoxychalcone (**7**) *trans*-4′-*O*-β-D-(glucopyranosyl)-3,5-dimethoxychalcone (**7a**) and *cis*-4′-*O*-β-D-(glucopyranosyl)-3,5-dimethoxychalcone (**7b**). 11
**Figure S22** Chromatogram of reaction mixture of trans-4′-hydroxy-3,4,5-trimethoxychalcone (**8**) and trans-4′-O-β-D-(glucopyranosyl)-3,4,5-trimethoxychalcone (**8a**) before product purification. 12
**Figure S23** Chromatogram of mixture of trans-4′-O-β-D-(glucopyranosyl)-3,4,5-trimethoxychalcone (**8a**) and cis-4′-O-β-D-(glucopyranosyl)-3,4,5-trimethoxychalcone (**8b**) after NMR analysis. 12
**Figure S24** UV-VIS spectra of *trans*-4′-hydroxy-3,4,5-trimethoxychalcone (**8**) *trans*-4′-*O*-β-D-(glucopyranosyl)-3,4,5-trimethoxychalcone (**8a**) and *cis*-4′-*O*-β-D-(glucopyranosyl)-3,4,5-trimethoxychalcone (**8b**). 12
**Figure S25** $^1$H NMR spectrum of trans-4′-O-β-D-(glucopyranosyl)-chalcone (600MHz; Acetone-$d_6$) 13
**Figure S26** Flavonoid part of $^1$H NMR spectrum of trans-4′-O-β-D-(glucopyranosyl)-chalcone (600MHz; Acetone-$d_6$) 13
**Figure S27** $^{13}$C NMR spectrum of trans-4′-O-β-D-(glucopyranosyl)-chalcone (151MHz; Acetone-$d_6$) 14
**Figure S28** COSY NMR spectrum of trans-4′-O-β-D-(glucopyranosyl)-chalcone (600MHz; Acetone-$d_6$) 14
**Figure S29** HMQC NMR spectrum of trans-4′-O-β-D-(glucopyranosyl)-chalcone (600MHz; Acetone-$d_6$) 15
**Figure S30** HMBC NMR spectrum of trans-4′-O-β-D-(glucopyranosyl)-chalcone (600MHz; Acetone-$d_6$) 15
**Figure S31** $^1$H NMR spectrum of cis-4′-O-β-D-(glucopyranosyl)-chalcone (600MHz; Acetone-$d_6$) 16
**Figure S32** Flavonoid part of $^1$H NMR spectra of cis-4′-O-β-D-(glucopyranosyl)-chalcone (600MHz; Acetone-$d_6$) 16
**Figure S33** $^{13}$C NMR spectra of cis-4′-O-β-D-(glucopyranosyl)-chalcone (151MHz; Acetone-$d_6$) 17
**Figure S34** COSY NMR spectra of cis-4′-O-β-D-(glucopyranosyl)-chalcone (600MHz; Acetone-$d_6$) 17
**Figure S35** HMQC NMR spectra of cis-4′-O-β-D-(glucopyranosyl)-chalcone (600MHz; Acetone-$d_6$) 18
**Figure S36** HMBC NMR spectra of cis-4′-O-β-D-(glucopyranosyl)-chalcone (600MHz; Acetone-$d_6$) 18
**Figure S37** $^1$H NMR spectrum of trans-4′-O-β-D-(glucopyranosyl)-2-methoxychalcone (600MHz; Acetone-$d_6$) 19
**Figure S38** Flavonoid part of $^1$H NMR spectrum of trans-4′-O-β-D-(glucopyranosyl)-2-methoxychalcone (600MHz; Acetone-$d_6$) 19
**Figure S39** $^{13}$C NMR spectrum of trans-4′-O-β-D-(glucopyranosyl)-2-methoxychalcone (151MHz; Acetone-$d_6$) 20
**Figure S40** COSY NMR spectrum of trans-4′-O-β-D-(glucopyranosyl)-chalcone (600MHz; Acetone-$d_6$) 20
**Figure S41** HMQC NMR spectrum of trans-4′-O-β-D-(glucopyranosyl)-2-methoxychalcone (600MHz; Acetone-$d_6$) 21
**Figure S42** HMBC NMR spectrum of trans-4′-O-β-D-(glucopyranosyl)-2-methoxychalcone (600MHz; Acetone-$d_6$) 21
**Figure S43** $^1$H NMR spectrum of cis-4′-O-β-D-(glucopyranosyl)-2-methoxychalcone (600MHz; Acetone-$d_6$) 22
**Figure S44** Flavonoid part of $^1$H NMR spectrum of cis-4′-O-β-D-(glucopyranosyl)-2-methoxychalcone (600MHz; Acetone-$d_6$) 22
**Figure S45** $^{13}$C NMR spectrum of cis-4′-O-β-D-(glucopyranosyl)-2-methoxychalcone (151MHz; Acetone-$d_6$) 23
**Figure S46** COSY NMR spectrum of cis-4′-O-β-D-(glucopyranosyl)-2-methoxychalcone (600MHz; Acetone-$d_6$) 23
**Figure S47** HMQC NMR spectrum of cis-4′-O-β-D-(glucopyranosyl)-2-methoxychalcone (600MHz; Acetone-$d_6$) 24
**Figure S48** HMBC NMR spectrum of cis-4′-O-β-D-(glucopyranosyl)-2-methoxychalcone (600MHz; Acetone-$d_6$) 24
**Figure S49** $^1$H NMR spectrum of trans-4′-O-β-D-(glucopyranosyl)-3-methoxychalcone (600MHz; Acetone-$d_6$) 25
**Figure S50** Flavonoid part of $^1$H NMR spectrum of trans-4′-O-β-D-(glucopyranosyl)-3-methoxychalcone (600MHz; Acetone-$d_6$) 25
**Figure S51** $^{13}$C NMR spectrum of trans-4′-O-β-D-(glucopyranosyl)-3-methoxychalcone (151MHz; Acetone-$d_6$) 26
**Figure S52** COSY NMR spectrum of trans-4′-O-β-D-(glucopyranosyl)-3-methoxychalcone (600MHz; Acetone-$d_6$) 26
**Figure S53** HMQC NMR spectrum of trans-4′-O-β-D-(glucopyranosyl)-3-methoxychalcone (600MHz; Acetone-$d_6$) 27
**Figure S54** HMBC NMR spectrum of *trans*-4′-*O*-β-D-(glucopyranosyl)-3-methoxychalcone (600MHz; Acetone-$d_6$) 27
**Figure S55** $^1$H NMR spectrum of cis-4′-O-β-D-(glucopyranosyl)-3-methoxychalcone (600MHz; Acetone-$d_6$) 28
**Figure S56** Flavonoid part of $^1$H NMR spectrum of cis-4′-O-β-D-(glucopyranosyl)-3-methoxychalcone (600MHz; Acetone-$d_6$) 28
**Figure S57** COSY NMR spectrum of cis-4′-O-β-D-(glucopyranosyl)-3-methoxychalcone (600MHz; Acetone-$d_6$) 29
**Figure S58** HMQC NMR spectrum of cis-4′-O-β-D-(glucopyranosyl)-3-methoxychalcone (600MHz; Acetone-$d_6$) 29
**Figure S59** HMBC NMR spectrum of cis-4′-O-β-D-(glucopyranosyl)-3-methoxychalcone (600MHz; Acetone-$d_6$) 30
**Figure S60** $^1$H NMR spectrum of trans-4′-O-β-D-(glucopyranosyl)-4-methoxychalcone (600MHz; Acetone-$d_6$) 30
**Figure S61** Flavonoid part of $^1$H NMR spectrum of trans-4′-O-β-D-(glucopyranosyl)-4-methoxychalcone (600MHz; Acetone-$d_6$) 31
**Figure S62** $^{13}$C NMR spectrum of trans-4′-O-β-D-(glucopyranosyl)-4-methoxychalcone (600MHz; Acetone-$d_6$) 31

**Figure S63** COSY NMR spectrum of trans-4′-O-β-D-(glucopyranosyl)-4-methoxychalcone (600MHz; Acetone-$d_6$) 32
**Figure S64** HMQC NMR spectrum of trans-4′-O-β-D-(glucopyranosyl)-4-methoxychalcone (600MHz; Acetone-$d_6$) 32
**Figure S65** HMBC NMR spectrum of trans-4′-O-β-D-(glucopyranosyl)-4-methoxychalcone (600MHz; Acetone-$d_6$) 33
**Figure S66** $^1$H NMR spectrum of cis-4′-O-β-D-(glucopyranosyl)-4-methoxychalcone (600MHz; Acetone-$d_6$) 33
**Figure S67** Flafonoid part of $^1$H NMR spectrum of cis-4′-O-β-D-(glucopyranosyl)-4-methoxychalcone (600MHz; Acetone-$d_6$) 34
**Figure S68** $^{13}$C NMR spectrum of cis-4′-O-β-D-(glucopyranosyl)-4-methoxychalcone (151MHz; Acetone-$d_6$) 34
**Figure S69** COSY NMR spectrum of cis-4′-O-β-D-(glucopyranosyl)-4-methoxychalcone (600MHz; Acetone-$d_6$) 35
**Figure S70** HMQC NMR spectrum of cis-4′-O-β-D-(glucopyranosyl)-4-methoxychalcone (600MHz; Acetone-$d_6$) 35
**Figure S71** HMBC NMR spectrum of cis-4′-O-β-D-(glucopyranosyl)-4-methoxychalcone (600MHz; Acetone-$d_6$) 36
**Figure S72** $^1$H NMR spectrum of trans-4′-hydroxy-2,4-dimethoxychalcone (600MHz; DMSO-$d_6$) 36
**Figure S73** Flavonoid part of spectrum of trans-4′-hydroxy-2,4-dimethoxychalcone (600MHz; DMSO-$d_6$) 37
**Figure S74** $^{13}$C NMR spectrum of trans-4′-hydroxy-2,4-dimethoxychalcone (151MHz; DMSO-$d_6$) 37
**Figure S75** COSY NMR spectrum of trans-4′-hydroxy-2,4-dimethoxychalcone (600MHz; DMSO-$d_6$) 38
**Figure S76** HMQC NMR spectrum of trans-4′-hydroxy-2,4-dimethoxychalcone (600MHz; DMSO-$d_6$) 38
**Figure S77** HMBC NMR spectrum of trans-4′-hydroxy-2,4-dimethoxychalcone (600MHz; DMSO-$d_6$) 39
**Figure S78** $^1$H NMR spectrum of *trans*-4′-*O*-β-D-(glucopyranosyl)-2,4-dimethoxychalcone (600MHz, Acetone-$d_6$) 39
Figure S79 Flavonoid fragment of $^1$H NMR spectrum of *trans*-4′-*O*-β-D-(glucopyranosyl)-2,4-dimethoxychalcone (600MHz, Acetone-$d_6$) 40
Figure S80 $^{13}$C NMR spectrum of *trans*-4′-*O*-β-D-(glucopyranosyl)-2,4-dimethoxychalcone (151MHz, Acetone-$d_6$) 40
**Figure S81** COSY NMR spectrum of trans-4′-O-β-D-(glucopyranosyl)-2,4-dimethoxychalcone (600MHz, Acetone-$d_6$) 41
**Figure S82** HMQC NMR spectrum of trans-4′-O-β-D-(glucopyranosyl)-2,4-dimethoxychalcone (600MHz, Acetone-$d_6$) 41
**Figure S83** HMBC NMR spectrum of trans-4′-O-β-D-(glucopyranosyl)-2,4-dimethoxychalcone (600MHz, Acetone-$d_6$) 42
**Figure S84** $^1$H NMR spectrum of trans-4′-hydroxy-2,5-dimethoxychalcone (600MHz; DMSO-$d_6$) 42
**Figure S85** Flavonoid part of $^1$H NMR spectrum of trans-4′-hydroxy-2,5-dimethoxychalcone (600MHz; DMSO-$d_6$) 43
**Figure S86** $^{13}$C NMR spectrum of 4′-hydroxy-2,5-dimethoxychalcone (151MHz; DMSO-$d_6$) 43
**Figure S87** COSY NMR spectrum of trans-4′-hydroxy-2,5-dimethoxychalcone (600MHz; DMSO-$d_6$) 44
**Figure S88** HMQC NMR spectrum of trans-4′-hydroxy-2,5-dimethoxychalcone (600MHz; DMSO-$d_6$) 44
**Figure S89** HMBC NMR spectrum of trans-4′-hydroxy-2,5-dimethoxychalcone (600MHz; DMSO-$d_6$) 45
**Figure S90** $^1$H NMR spectrum of cis-4′-O-β-D-(glucopyranosyl)-2,5-dimethoxychalcone (600MHz; Acetone-$d_6$) 45
**Figure S91** Flavonoid fragment of $^1$H NMR spectrum of cis-4′-O-β-D-(glucopyranosyl)-2,5-dimethoxychalcone (600MHz; Acetone-$d_6$) 46
**Figure S92** $^{13}$C NMR spectrum of cis-4′-O-β-D-(glucopyranosyl)-2,5-dimethoxychalcone (151MHz; Acetone-$d_6$) 46
**Figure S93** COSY NMR spectrum of cis-4′-O-β-D-(glucopyranosyl)-2,5-dimethoxychalcone (600MHz; Acetone-$d_6$) 47
**Figure S94** HMQC NMR spectrum of cis-4′-O-β-D-(glucopyranosyl)-2,5-dimethoxychalcone (600MHz; Acetone-$d_6$) 47
**Figure S95** HMBC NMR spectrum of cis-4′-O-β-D-(glucopyranosyl)-2,5-dimethoxychalcone (600MHz; Acetone-$d_6$) 48
**Figure S96** $^1$H NMR spectrum of trans-4′-hydroxy-3,5-dimethoxychalcone (600MHz, DMSO-$d_6$) 48
**Figure S97** Flavonoid part of spectrum of $^1$H NMR of trans-4′-hydroxy-3,5-dimethoxychalcone (600MHz, DMSO-$d_6$) 49
**Figure S98** $^{13}$C NMR spectrum of trans-4′-hydroxy-3,5-dimethoxychalcone (151MHz, DMSO-$d_6$) 49
**Figure S99** COSY NMR spectrum of trans-4′-hydroxy-3,5-dimethoxychalcone (600MHz, DMSO-$d_6$) 50
**Figure S100** HMQC NMR spectrum of trans-4′-hydroxy-3,5-dimethoxychalcone (600MHz, DMSO-$d_6$) 50
**Figure S101** HMBC NMR spectrum of trans-4′-hydroxy-3,5-dimethoxychalcone (600MHz, DMSO-$d_6$) 51
**Figure S102** $^1$H NMR spectrum of trans-4′-O-β-D-(glucopyranosyl)-3,5-dimethoxychalcone (600MHz, DMSO-$d_6$) 51
**Figure S103** Flavonoid part of $^1$H NMR spectrum of trans-4′-O-β-D-(glucopyranosyl)-3,5-dimethoxychalcone (600MHz, Acetone-$d_6$) 52
**Figure S104** $^1$H NMR spectrum of mixture of trans- and cis-4′-O-β-D-(glucopyranosyl)-3,5-dimethoxychalcone (same probe as **Figure S102** measured second time after few days) (600MHz, Acetone-$d_6$) 52
**Figure S105** Flavonoid part of $^1$H NMR spectrum of trans- and cis-4′-O-β-D-(glucopyranosyl)-3,5-dimethoxychalcone (same probe as **Figure S103** measured second time after few days) (600MHz, Acetone-$d_6$) 53
**Figure S106** COSY NMR spectrum of trans- and cis-4′-O-β-D-(glucopyranosyl)-3,5-dimethoxychalcone (600MHz, Acetone-$d_6$) 53
**Figure S107** HMQC NMR spectrum of trans- and cis-4′-O-β-D-(glucopyranosyl)-3,5-dimethoxychalcone (600MHz, Acetone-$d_6$) 54

**Figure S108** HMBC NMR spectrum of trans- and cis-4′-O-β-D-(glucopyranosyl)-3,5-dimethoxychalcone (600MHz, Acetone-$d_6$) 54
**Figure S109** $^1$H NMR spectrum of cis-4′-O-β-D-(glucopyranosyl)-3,5-dimethoxychalcone (600MHz, Acetone-$d_6$) 55
**Figure S110** Flavonoid part of spectrum of $^1$H NMR spectrum of cis-4′-O-β-D-(glucopyranosyl)-3,5-dimethoxychalcone (600MHz, Acetone-$d_6$) 55
**Figure S111** COSY NMR spectrum of $^1$H NMR spectrum of cis-4′-O-β-D-(glucopyranosyl)-3,5-dimethoxychalcone (600MHz, Acetone-$d_6$) 56
**Figure S112** HMQC NMR spectrum of cis-4′-O-β-D-(glucopyranosyl)-3,5-dimethoxychalcone (600MHz, Acetone-$d_6$) 56
**Figure S113** HMBC NMR spectrum of cis-4′-O-β-D-(glucopyranosyl)-3,5-dimethoxychalcone (600MHz, Acetone-$d_6$) 57
**Figure S114** $^1$H NMR of trans-4′-hydroxy-3,4,5-trimethoxychalcone (600MHz, DMSO-$d_6$) 57
**Figure S115** Flavonoid framgment of $^1$H NMR spectrum of trans-4′-hydroxy-3,4,5-trimethoxychalcone (600MHz, DMSO-$d_6$) 58
**Figure S116** $^{13}$C NMR of trans-4′-hydroxy-3,4,5-trimethoxychalcone (151MHz, DMSO-$d_6$) 58
**Figure S117** COSY NMR spectrum of trans-4′-hydroxy-3,4,5-trimethoxychalcone (600MHz, DMSO-$d_6$) 59
**Figure S118** HMQC NMR spectrum of trans-4′-hydroxy-3,4,5-trimethoxychalcone (600MHz, DMSO-$d_6$) 59
**Figure S119** HMBC NMR spectrum of trans-4′-hydroxy-3,4,5-trimethoxychalcone (600MHz, DMSO-$d_6$) 60
**Figure S120** $^1$H NMR spectrum of trans-4′-O-β-D-(glucopyranosyl)-3,4,5-trimethoxychalcone (600MHz, Acetone-$d_6$) 60
**Figure S121** Flavonoid fragment of $^1$H NMR spectrum of trans-4′-O-β-D-(glucopyranosyl)-3,4,5-trimethoxychalcone (600MHz, Acetone-$d_6$) 61
**Figure S122** $^{13}$C NMR spectrum of trans-4′-O-β-D-(glucopyranosyl)-3,4,5-trimethoxychalcone (151MHz, Acetone-$d_6$) 61
**Figure S123** COSY NMR spectrum of trans-4′-O-β-D-(glucopyranosyl)-3,4,5-trimethoxychalcone (600MHz, Acetone-$d_6$) 62
**Figure S124** HMQC NMR spectrum of trans-4′-O-β-D-(glucopyranosyl)-3,4,5-trimethoxychalcone (600MHz, Acetone-$d_6$) 62
**Figure S125** HMBC NMR spectrum of trans-4′-O-β-D-(glucopyranosyl)-3,4,5-trimethoxychalcone (600MHz, Acetone-$d_6$) 63
**Table S1** List of recombinant enzymes used with related accession. 63
**Figure S126** SDS-PAGE analysis of crude protein lysates and combined fractions after Ni-NTA purification 64
**Figure S127** SDS-PAGE analysis of repeated purification of UGT88F2. 64
**Figure S128** UPLC chromatograms of A – phloretin standard, B – control cascade reaction of UGT88F2 crude fraction with GmSuSy performed to verify its activity. 65

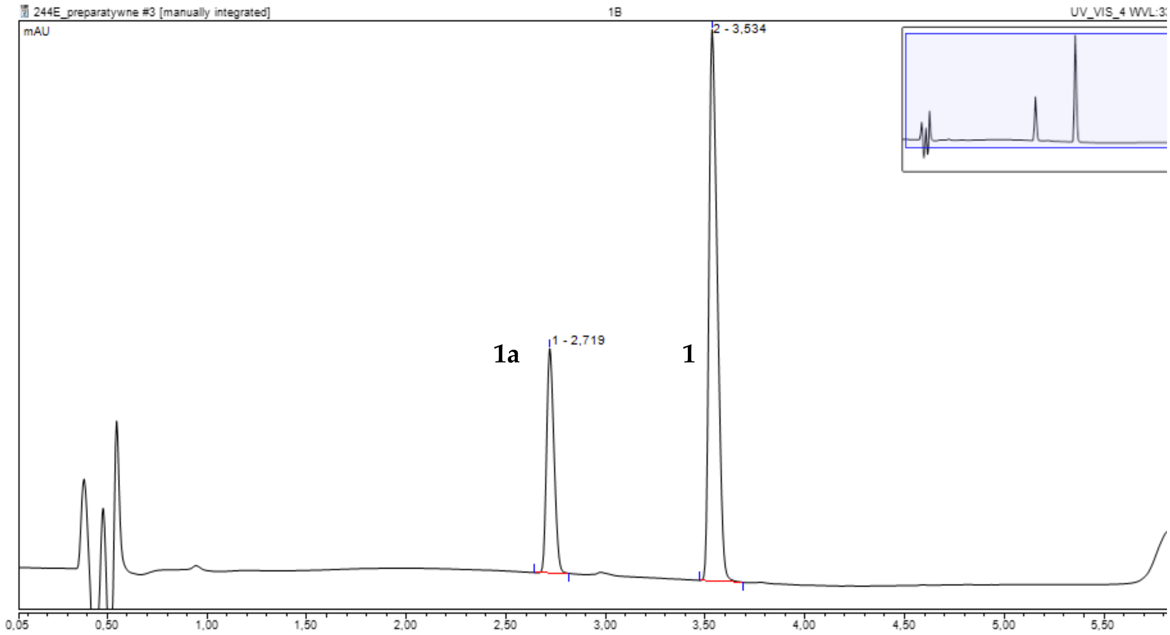


**Figure S1** Chromatogram of reaction mixture of *trans*-4′-hydroxychalcone (**1**) and *trans*-4′-*O*-β-D-(glucopyranosyl)-chalcone (**1a**) before product purification.

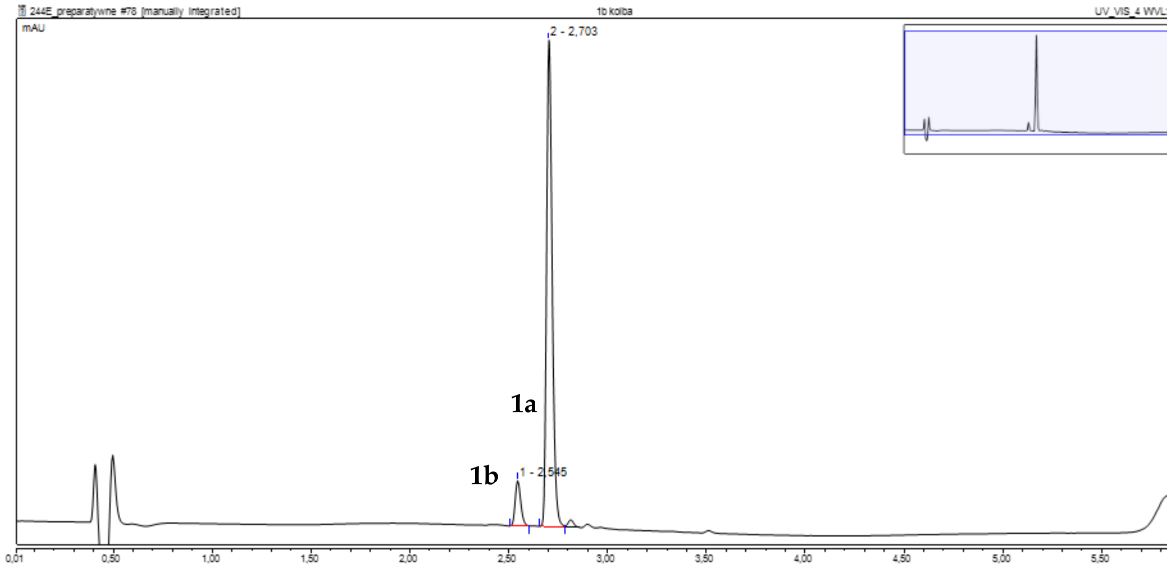


**Figure S2** Chromatogram of mixture of *trans*-4′-*O*-β-D-(glucopyranosyl)-chalcone (**1a**) and *cis*-4′-*O*-β-D-(glucopyranosyl)-chalcone (**1b**) after NMR analysis.

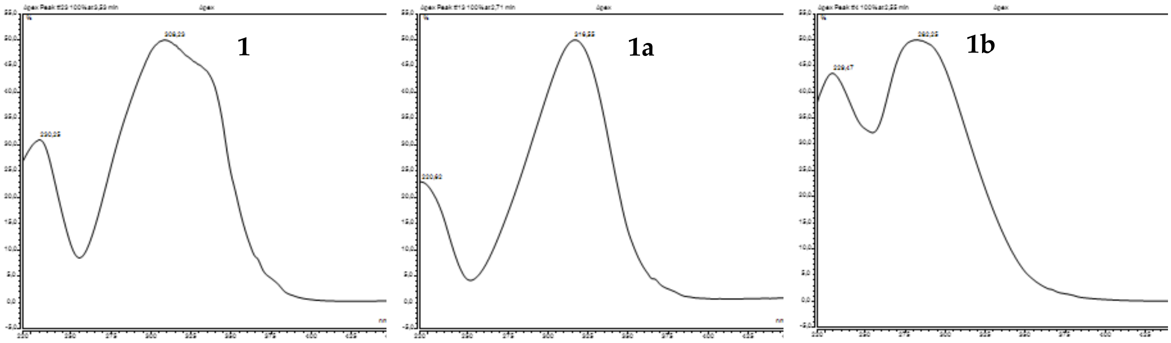


**Figure S3** UV-VIS spectra of *trans*-4′-hydroxychalcone (**1**) *trans*-4′-*O*-β-D-(glucopyranosyl)-chalcone (**1a**) and *cis*-4′-*O*-β-D-(glucopyranosyl)-chalcone (**1b**)

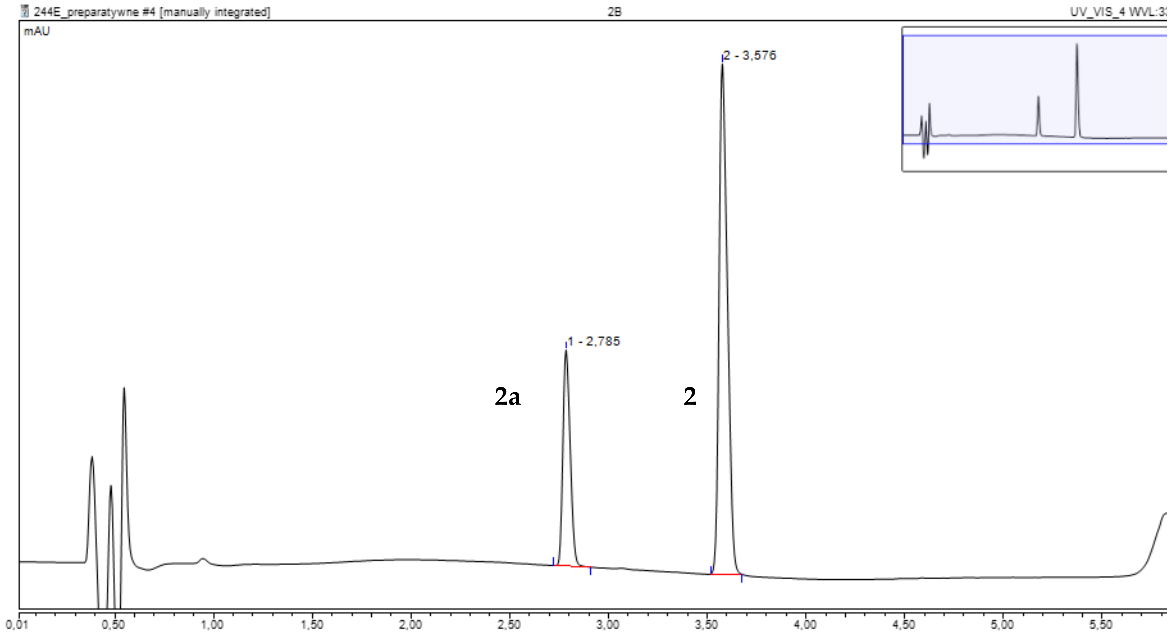


**Figure S4** Chromatogram of reaction mixture of *trans*-4′-hydroxy-2-methoxychalcone (**2**) and *trans*-4′-*O*-β-D-(glucopyranosyl)-2-methoxychalcone (**2a**) before product purification.

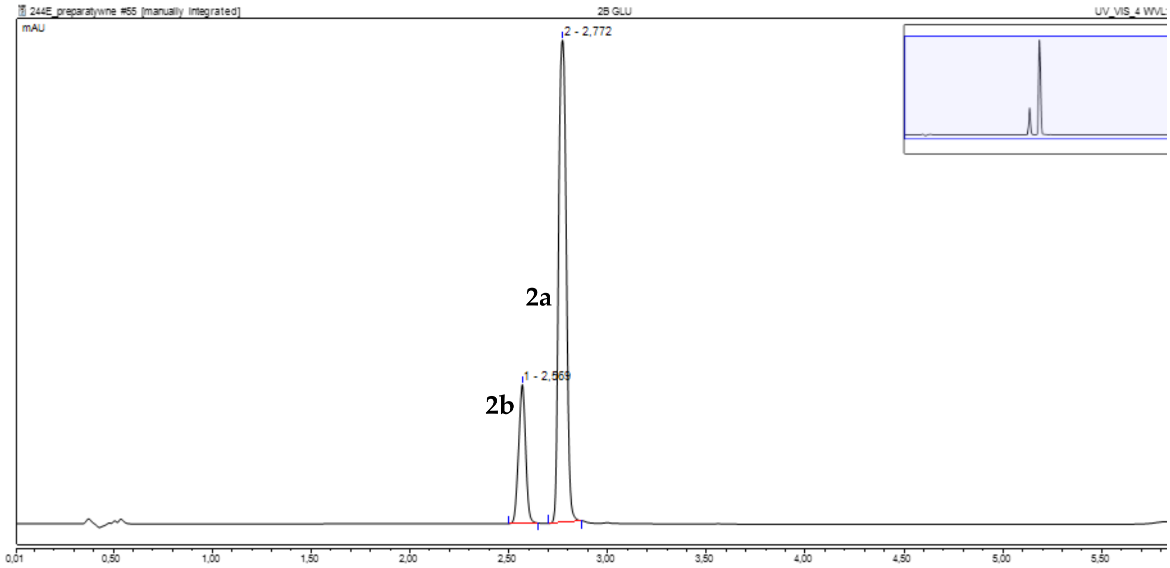


**Figure S5** Chromatogram of mixture of *trans*-4′-*O*-β-D-(glucopyranosyl)-2-methoxychalcone (**2a**) and *cis*-4′-*O*-β-D-(glucopyranosyl)-2-methoxychalcone (**2b**) after NMR analysis.

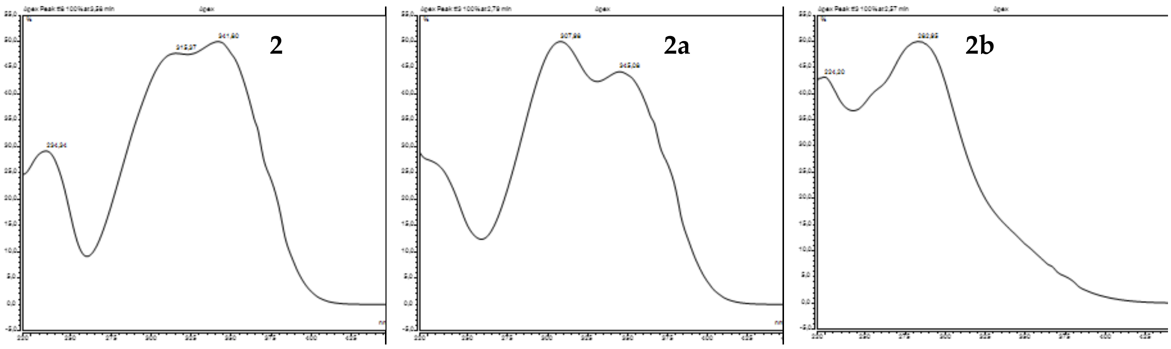


**Figure S6** UV-VIS spectra of *trans*-4′-hydroxy-2-methoxychalcone (**2**) *trans*-4′-*O*-β-D-(glucopyranosyl)-2-methoxychalcone (**2a**) and *cis*-4′-*O*-β-D-(glucopyranosyl)-2-methoxychalcone (**2b**).

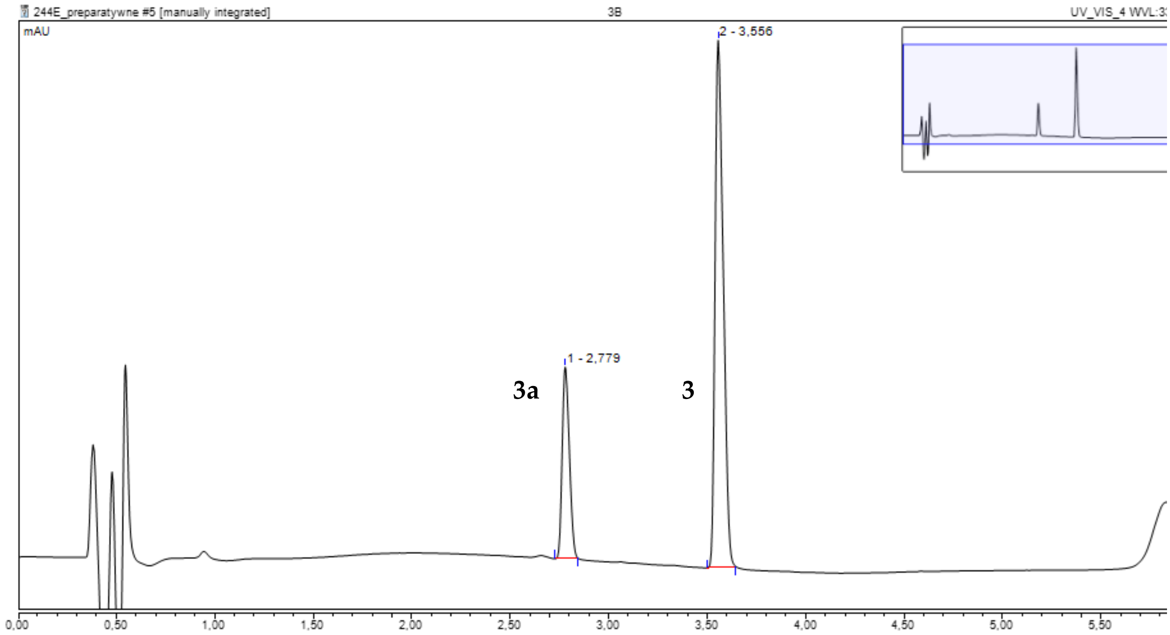


**Figure S7** Chromatogram of reaction mixture of *trans*-4′-hydroxy-3-methoxychalcone (**3**) and *trans*-4′-*O*-β-D-(glucopyranosyl)-3-methoxychalcone (**3a**) before product purification.

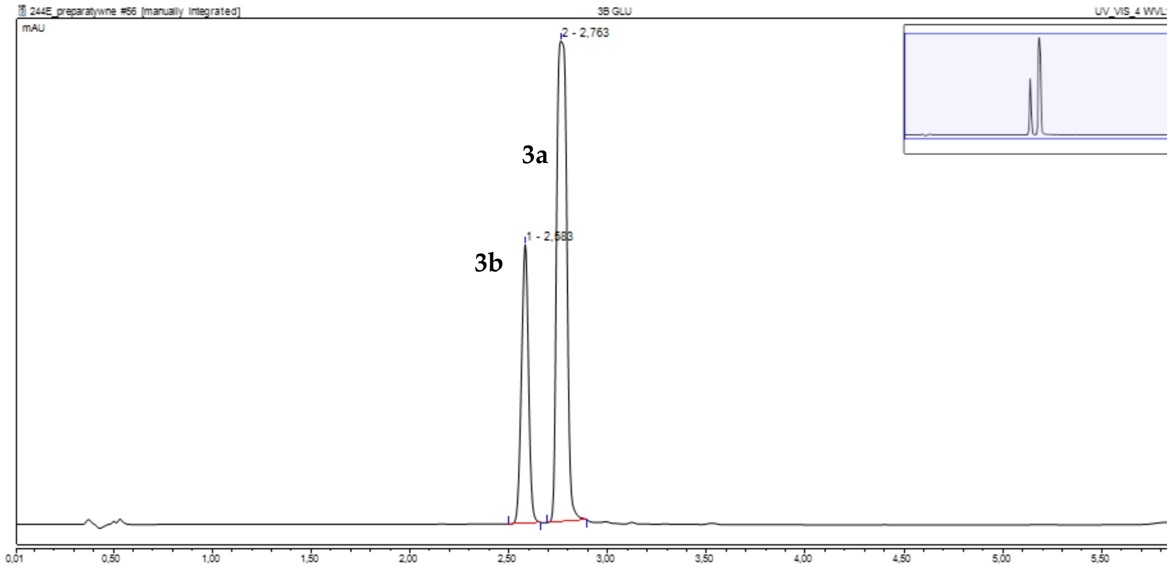


**Figure S8** Chromatogram of mixture of *trans*-4′-*O*-β-D-(glucopyranosyl)-3-methoxychalcone (**3a**) and *cis*-4′-*O*-β-D-(glucopyranosyl)-3-methoxychalcone (**3b**) after NMR analysis.

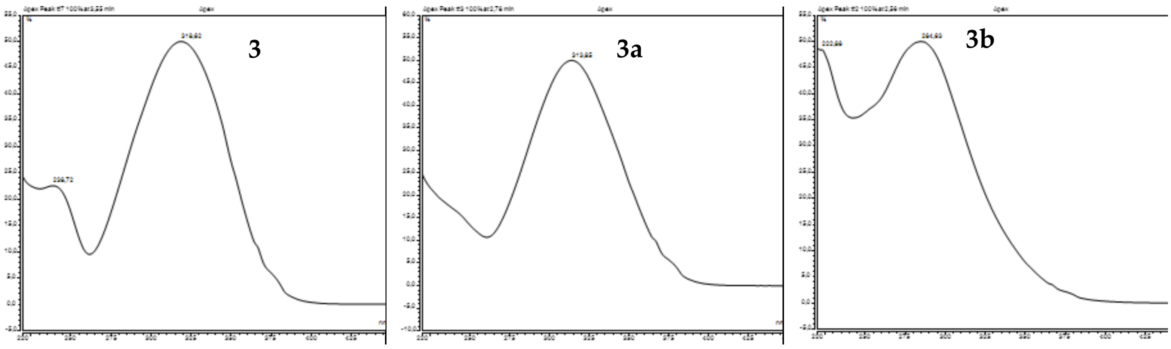


**Figure S9** UV-VIS spectra of *trans*-4′-hydroxy-3-methoxychalcone (**3**) *trans*-4′-*O*-β-D-(glucopyranosyl)-3-methoxychalcone (**3a**) and *cis*-4′-*O*-β-D-(glucopyranosyl)-3-methoxychalcone (**3b**).

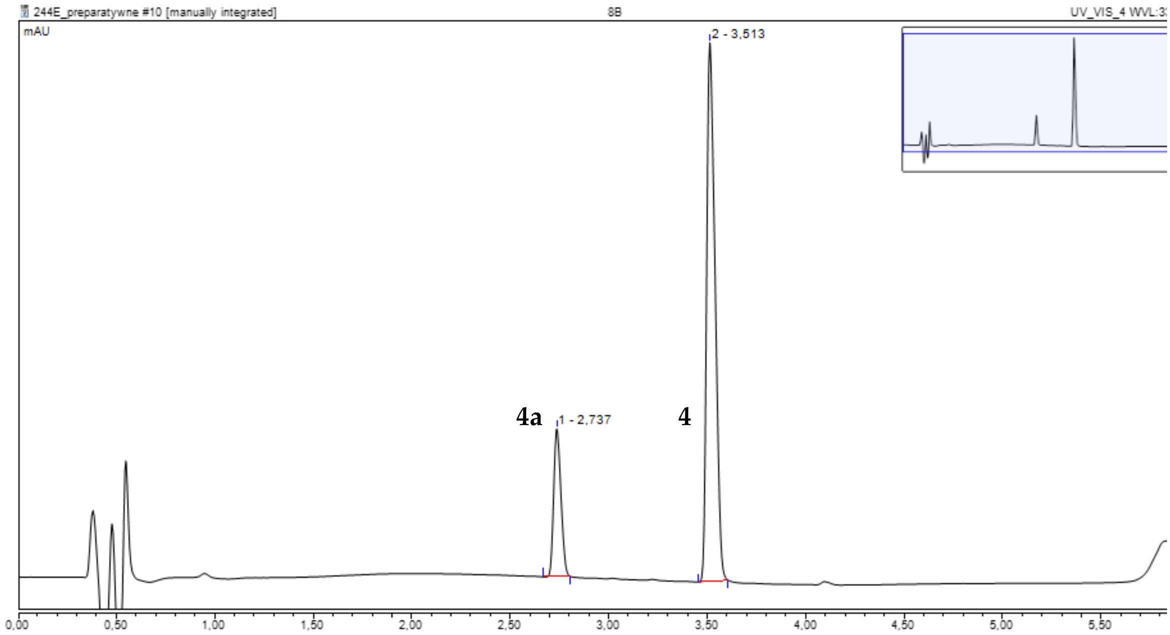


**Figure S10** Chromatogram of reaction mixture of *trans*-4′-hydroxy-4-methoxychalcone (**4**) and *trans*-4′-*O*-β-D-(glucopyranosyl)-4-methoxychalcone (**4a**) before product purification.

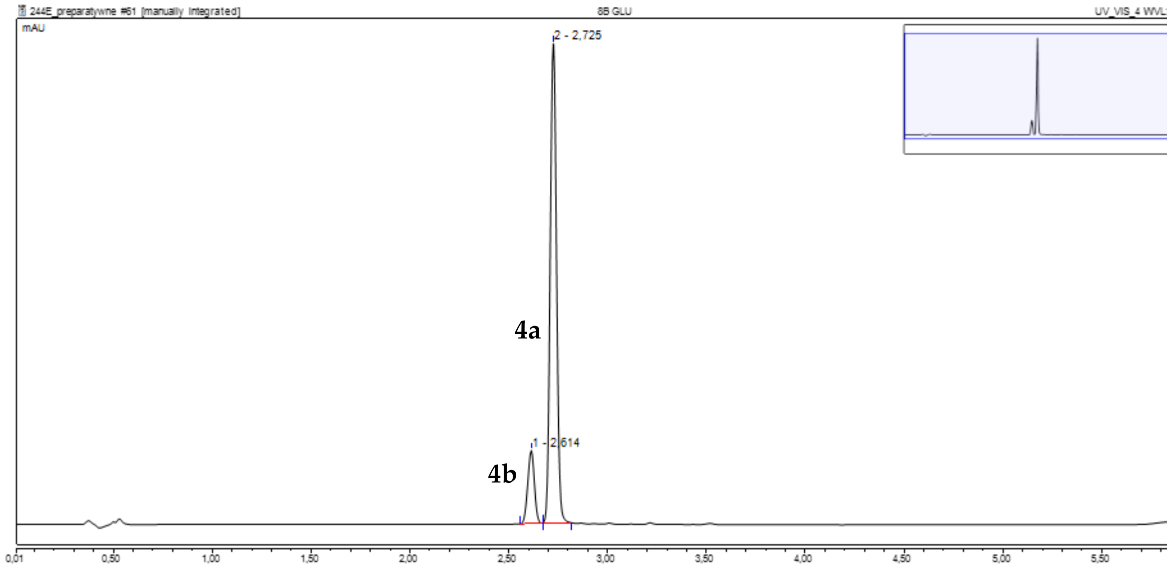


**Figure S11** Chromatogram of mixture of *trans*-4′-*O*-β-D-(glucopyranosyl)-4-methoxychalcone (**4a**) and *cis*-4′-*O*-β-D-(glucopyranosyl)-4-methoxychalcone (**4b**) after NMR analysis.

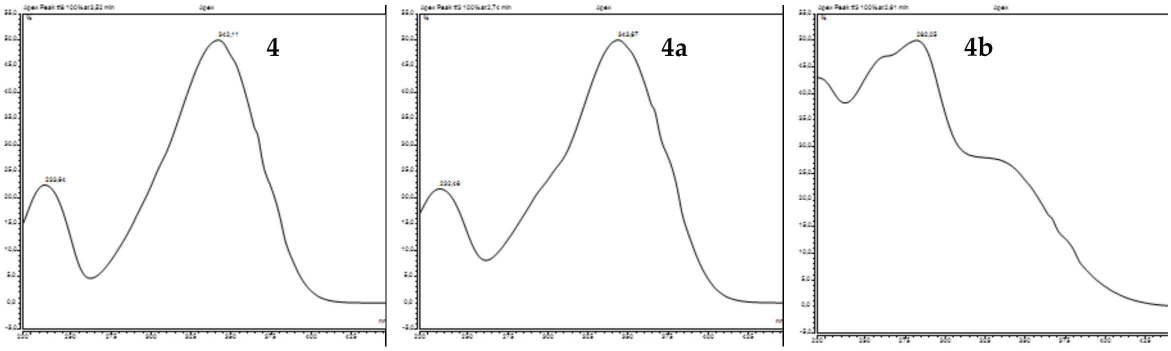


**Figure S12** UV-VIS spectra of *trans*-4′-hydroxy-4-methoxychalcone (**4**) *trans*-4′-*O*-β-D-(glucopyranosyl)-4-methoxychalcone (**4a**) and *cis*-4′-*O*-β-D-(glucopyranosyl)-4-methoxychalcone (**4b**).

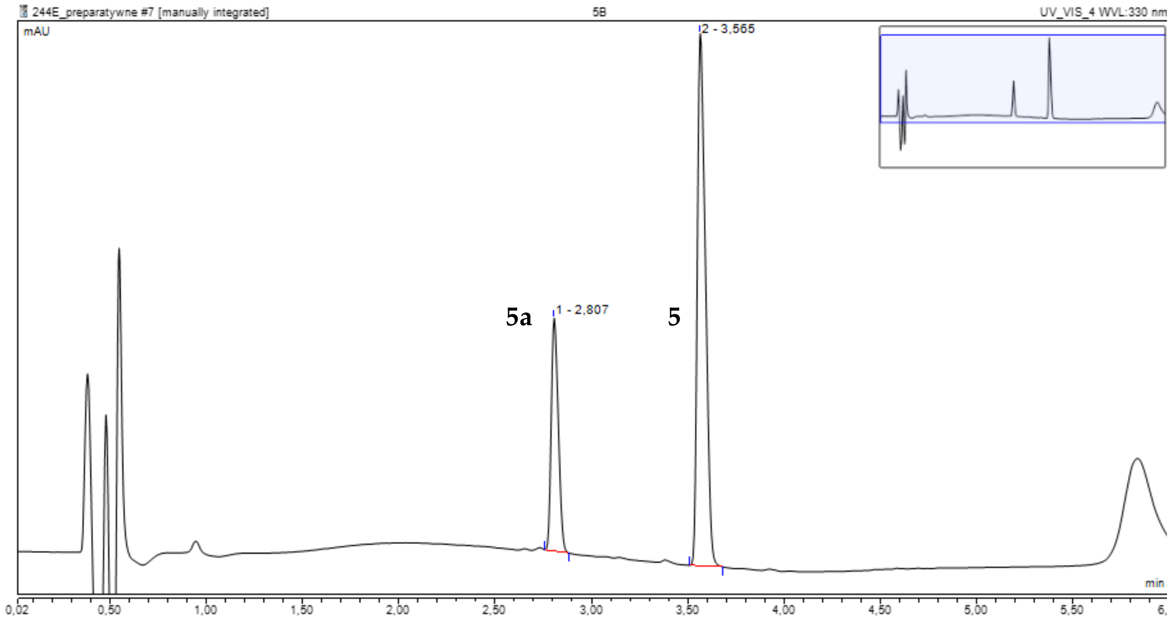


**Figure S13** Chromatogram of reaction mixture of *trans*-4′-hydroxy-2,4-dimethoxychalcone (**5**) and *trans*-4′-*O*-β-D-(glucopyranosyl)-2,4-dimethoxychalcone (**5a**) before product purification.

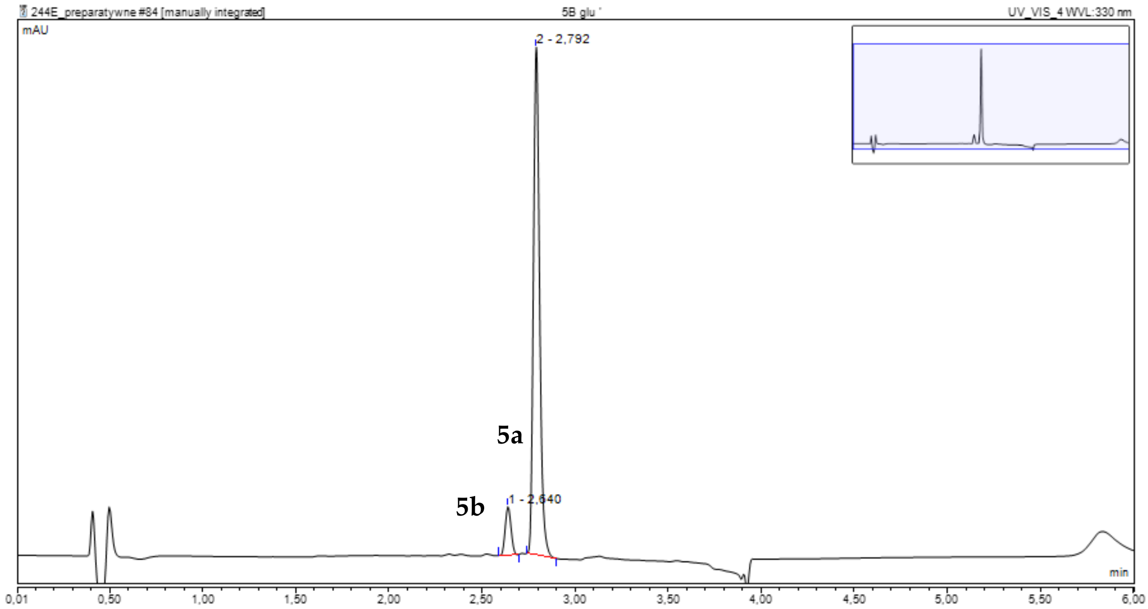


**Figure S14** Chromatogram of mixture of *trans*-4′-*O*-β-D-(glucopyranosyl)-2,4-dimethoxychalcone (**5a**) and *cis*-4′-*O*-β-D-(glucopyranosyl)-2,4-dimethoxychalcone (**5b**) after NMR analysis.

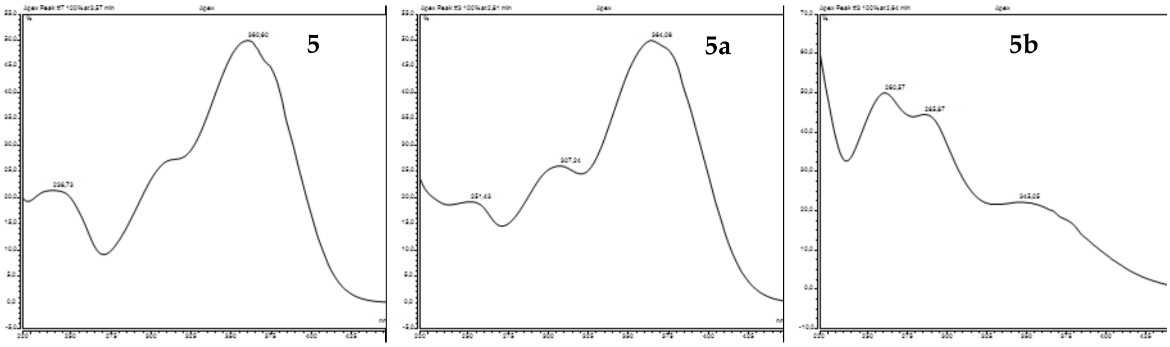


**Figure S15** UV-VIS spectra of *trans*-4′-hydroxy-2,4-dimethoxychalcone (**5**) *trans*-4′-*O*-β-D-(glucopyranosyl)-2,4-dimethoxychalcone (**5a**) and *cis*-4′-*O*-β-D-(glucopyranosyl)-2,4-dimethoxychalcone (**5b**).

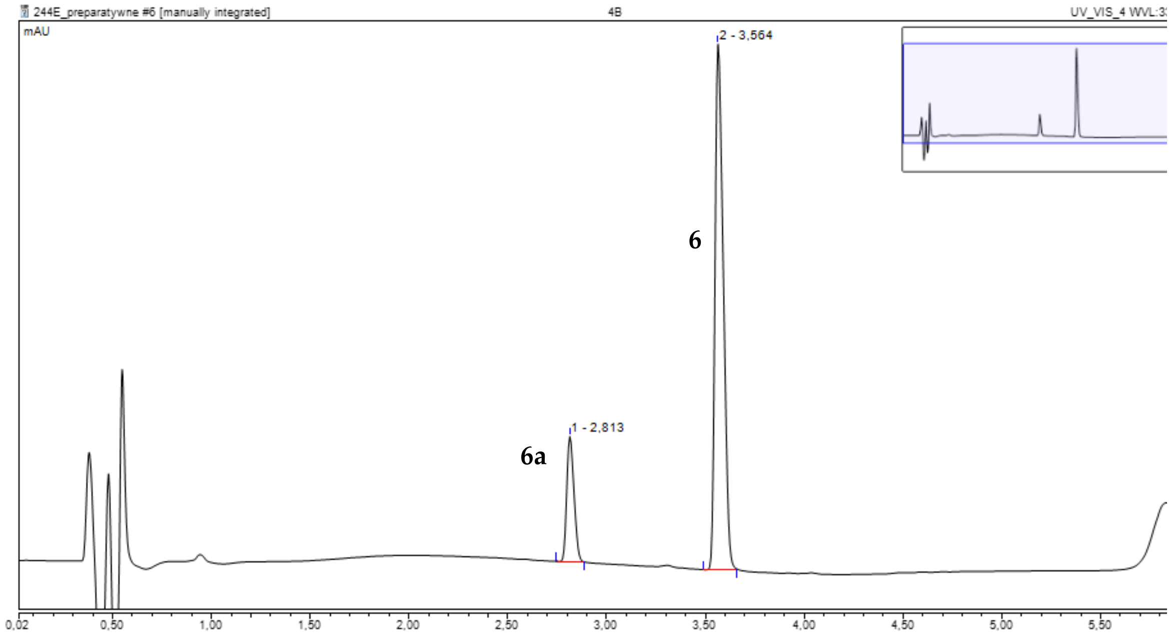


**Figure S16** Chromatogram of reaction mixture of *trans*-4′-hydroxy-2,5-dimethoxychalcone (**6**) and *trans*-4′-*O*-β-D-(glucopyranosyl)-2,5-dimethoxychalcone (**6a**) before product purification.

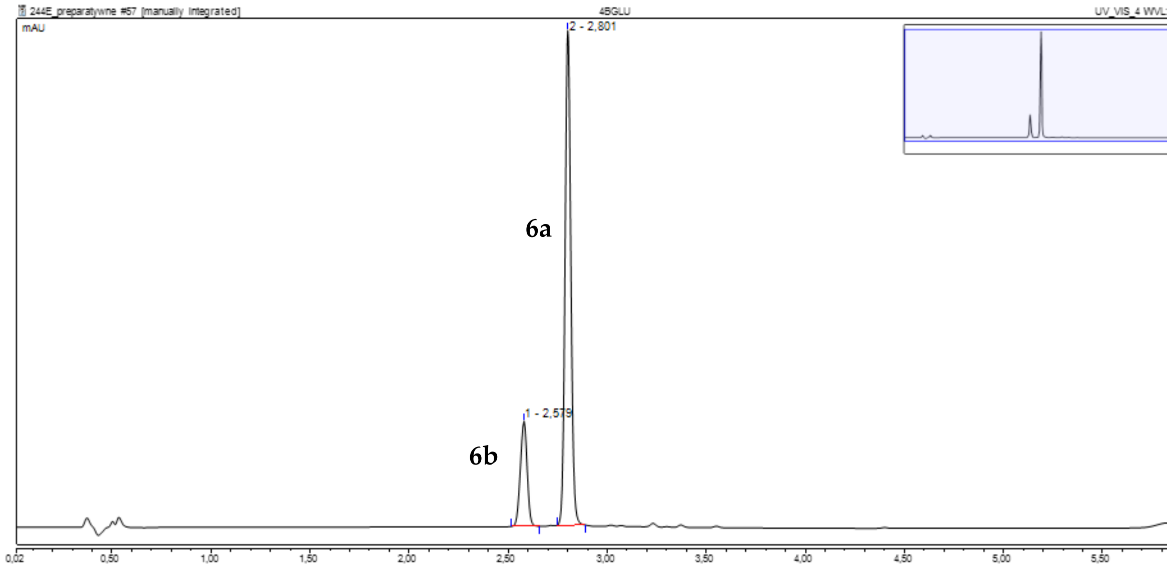


**Figure S17** Chromatogram of mixture of *trans*-4′-*O*-β-D-(glucopyranosyl)-2,5-dimethoxychalcone (**6a**) and *cis*-4′-*O*-β-D-(glucopyranosyl)-2,5-dimethoxychalcone (**6b**) after NMR analysis.

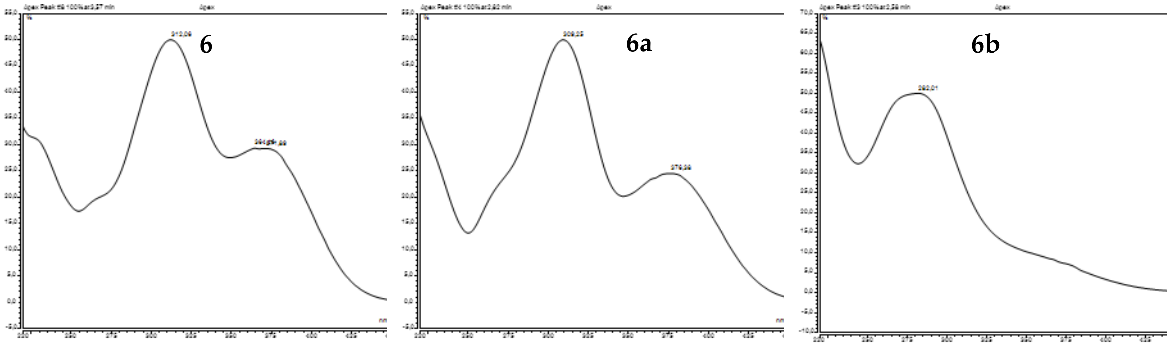


**Figure S18** UV-VIS spectra of *trans*-4′-hydroxy-2,5-dimethoxychalcone (**6**) *trans*-4′-*O*-β-D-(glucopyranosyl)-2,5-dimethoxychalcone (**6a**) and *cis*-4′-*O*-β-D-(glucopyranosyl)-2,5-dimethoxychalcone (**6b**).

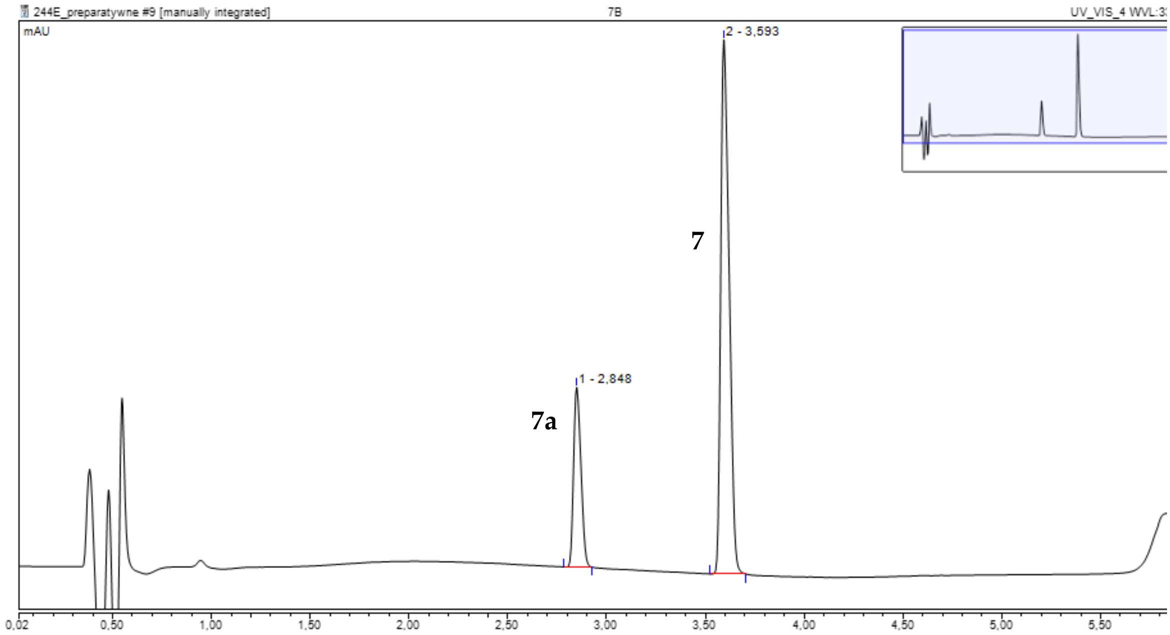


**Figure S19** Chromatogram of reaction mixture of *trans*-4′-hydroxy-3,5-dimethoxychalcone (**7**) and *trans*-4′-*O*-β-D-(glucopyranosyl)-3,5-dimethoxychalcone (**7a**) before product purification.

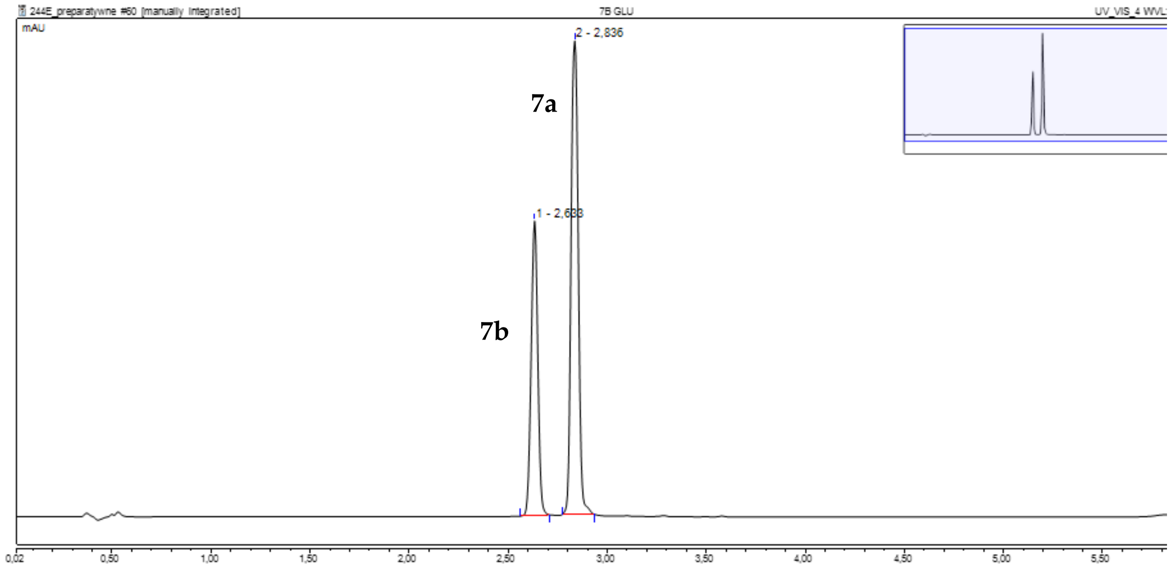


**Figure S20** Chromatogram of mixture of *trans*-4′-*O*-β-D-(glucopyranosyl)-3,5-dimethoxychalcone (**7a**) and *cis*-4′-*O*-β-D-(glucopyranosyl)-3,5-dimethoxychalcone (**7b**) after NMR analysis.

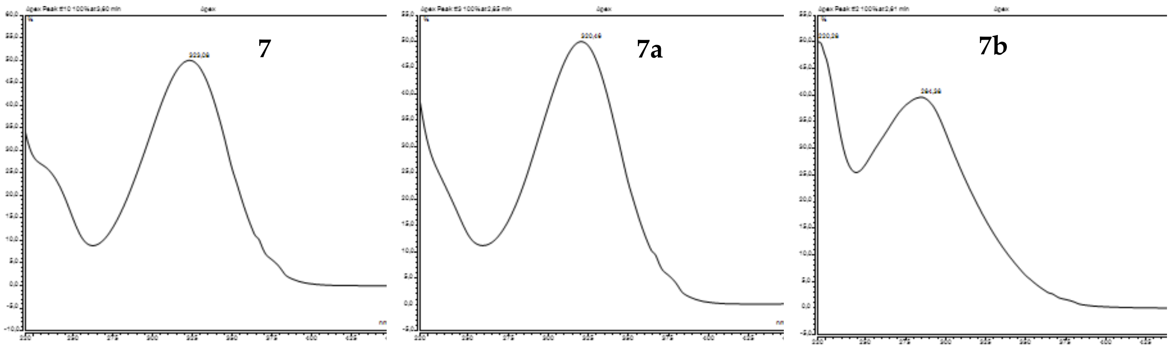


**Figure S21** UV-VIS spectra of *trans*-4′-hydroxy-3,5-dimethoxychalcone (**7**) *trans*-4′-*O*-β-D-(glucopyranosyl)-3,5-dimethoxychalcone (**7a**) and *cis*-4′-*O*-β-D-(glucopyranosyl)-3,5-dimethoxychalcone (**7b**).

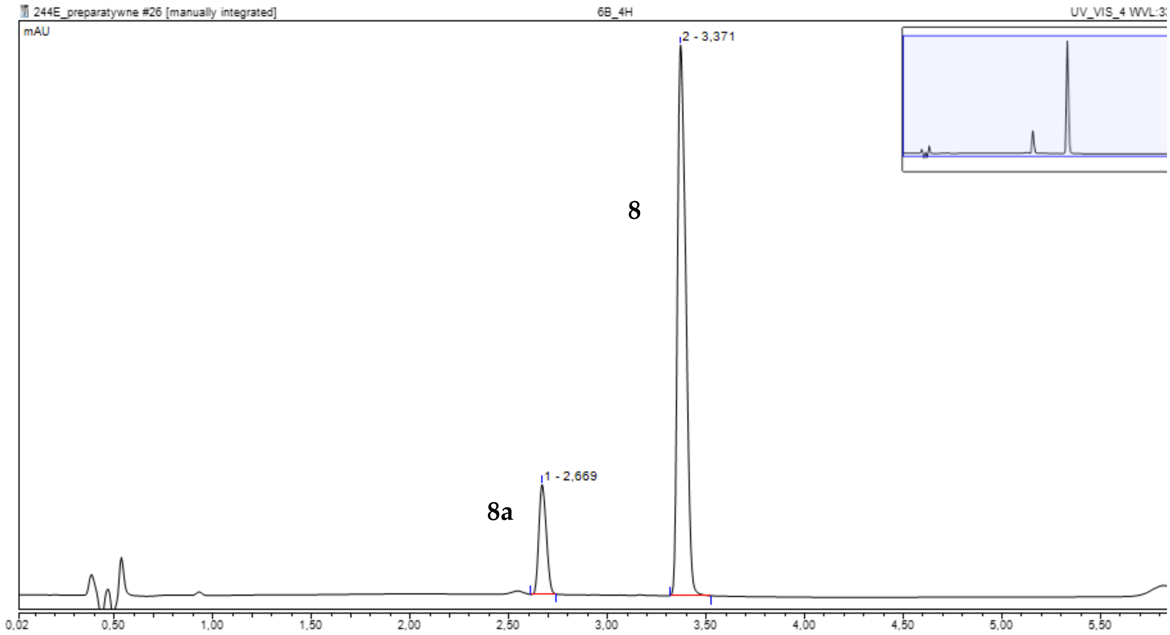


**Figure S22** Chromatogram of reaction mixture of *trans*-4′-hydroxy-3,4,5-trimethoxychalcone (**8**) and *trans*-4′-*O*-β-D-(glucopyranosyl)-3,4,5-trimethoxychalcone (**8a**) before product purification.

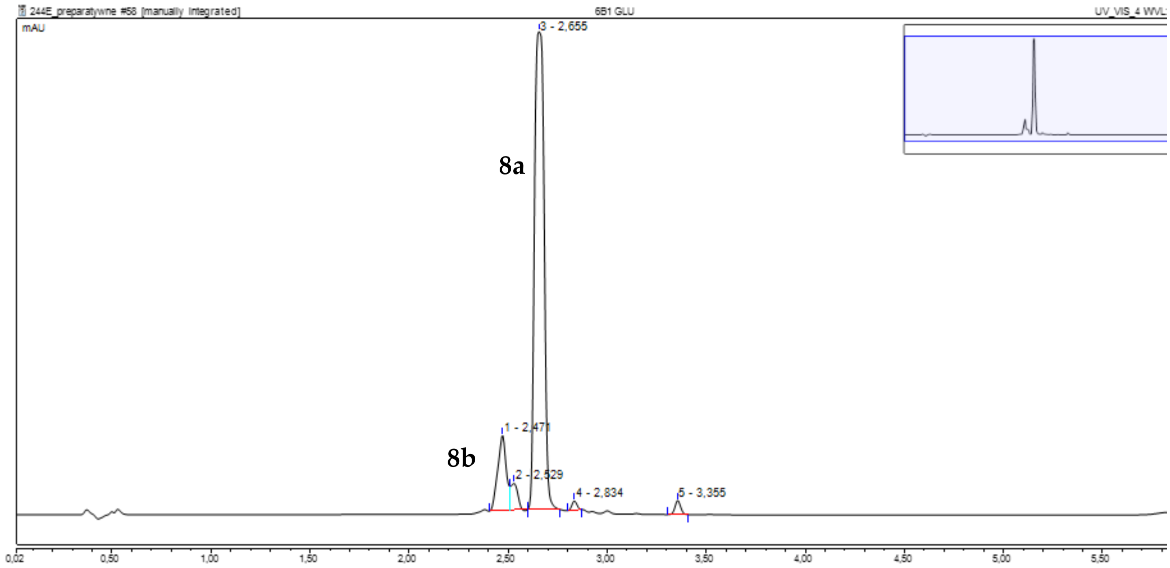


**Figure S23** Chromatogram of mixture of *trans*-4′-*O*-β-D-(glucopyranosyl)-3,4,5-trimethoxychalcone (**8a**) and *cis*-4′-*O*-β-D-(glucopyranosyl)-3,4,5-trimethoxychalcone (**8b**) after NMR analysis.

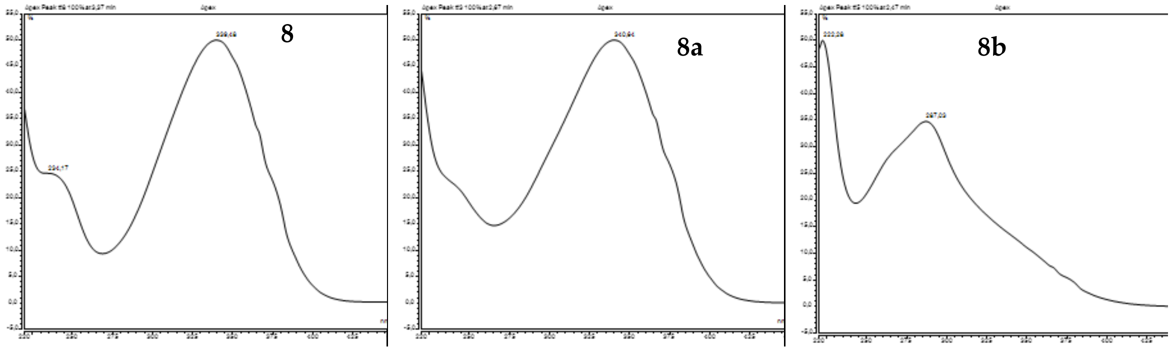


**Figure S24** UV-VIS spectra of *trans*-4′-hydroxy-3,4,5-trimethoxychalcone (**8**) *trans*-4′-*O*-β-D-(glucopyranosyl)-3,4,5-trimethoxychalcone (**8a**) and *cis*-4′-*O*-β-D-(glucopyranosyl)-3,4,5-trimethoxychalcone (**8b**).

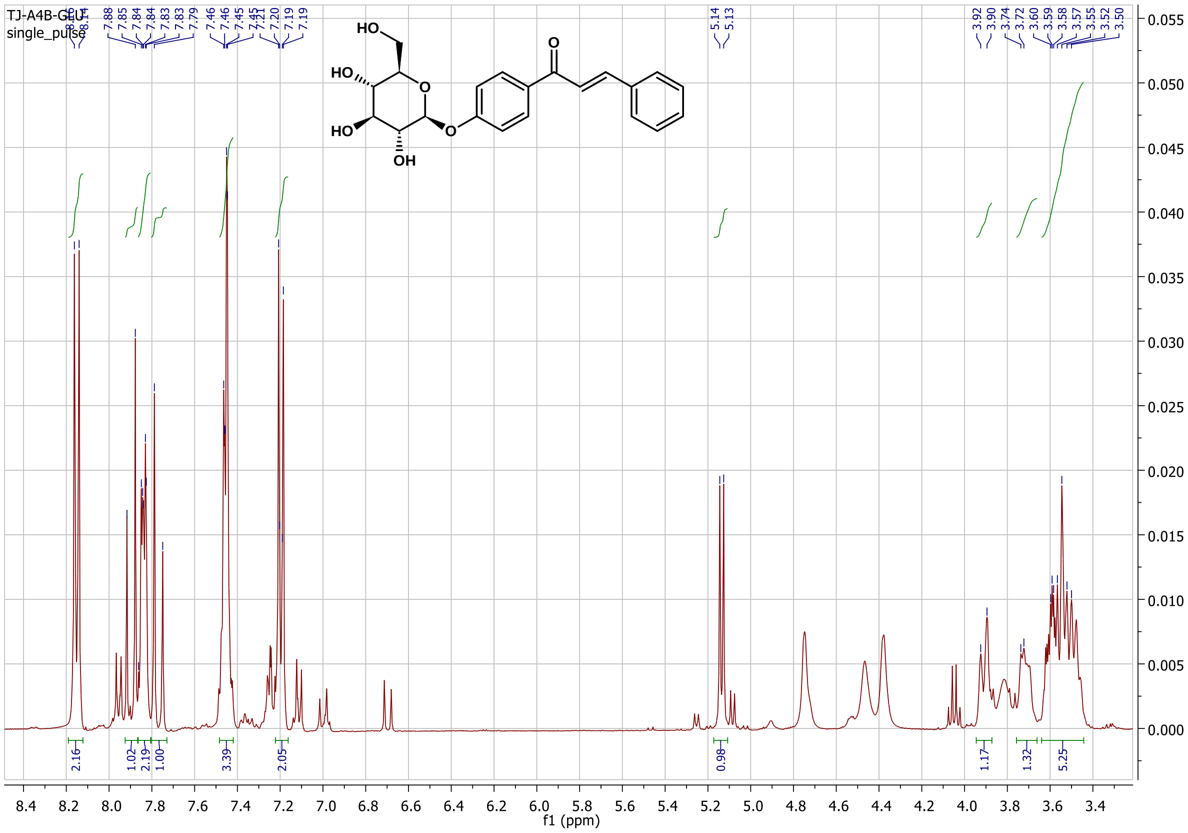


**Figure S25** $^1$H NMR spectrum of *trans*-4′-*O*-β-D-(glucopyranosyl)-chalcone (600MHz; Acetone-d$_6$)

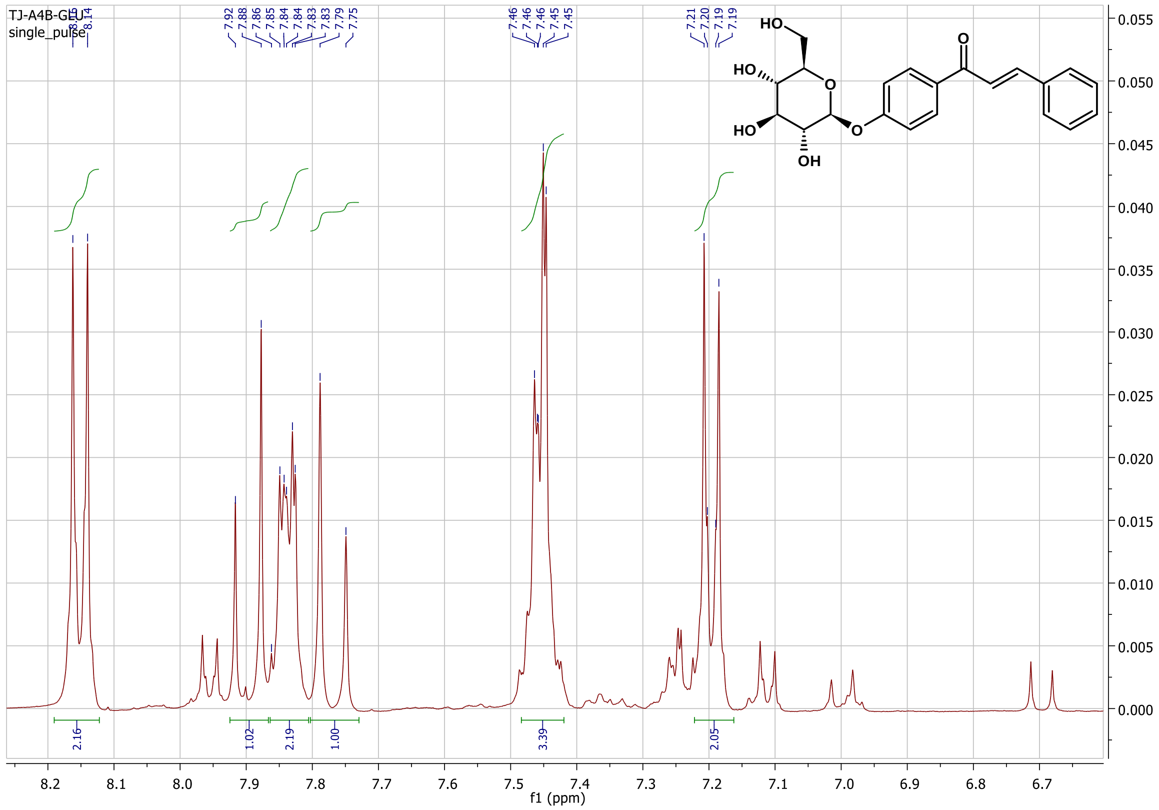


**Figure S26** Flavonoid part of $^1$H NMR spectrum of *trans*-4′-*O*-β-D-(glucopyranosyl)-chalcone (600MHz; Acetone-d$_6$)

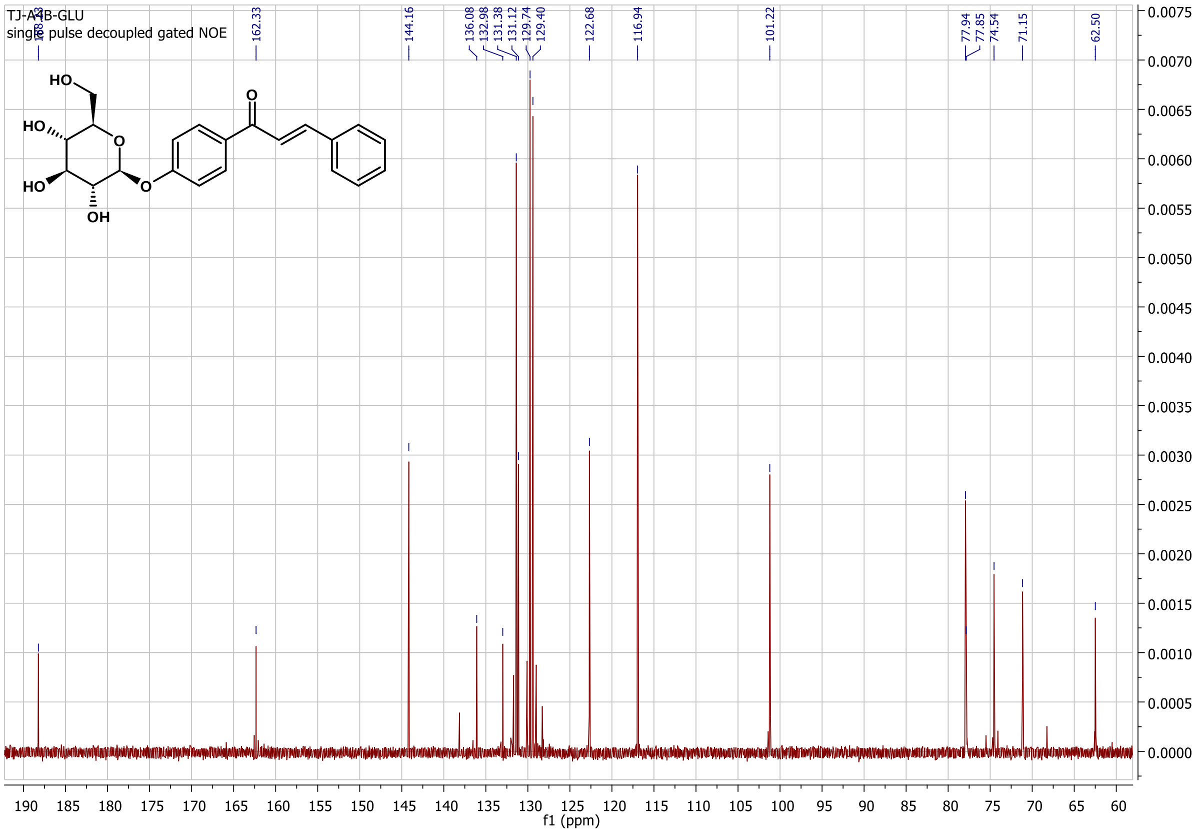


**Figure S27** $^{13}$C NMR spectrum of *trans*-4′-*O*-β-D-(glucopyranosyl)-chalcone (151MHz; Acetone-d$_6$)

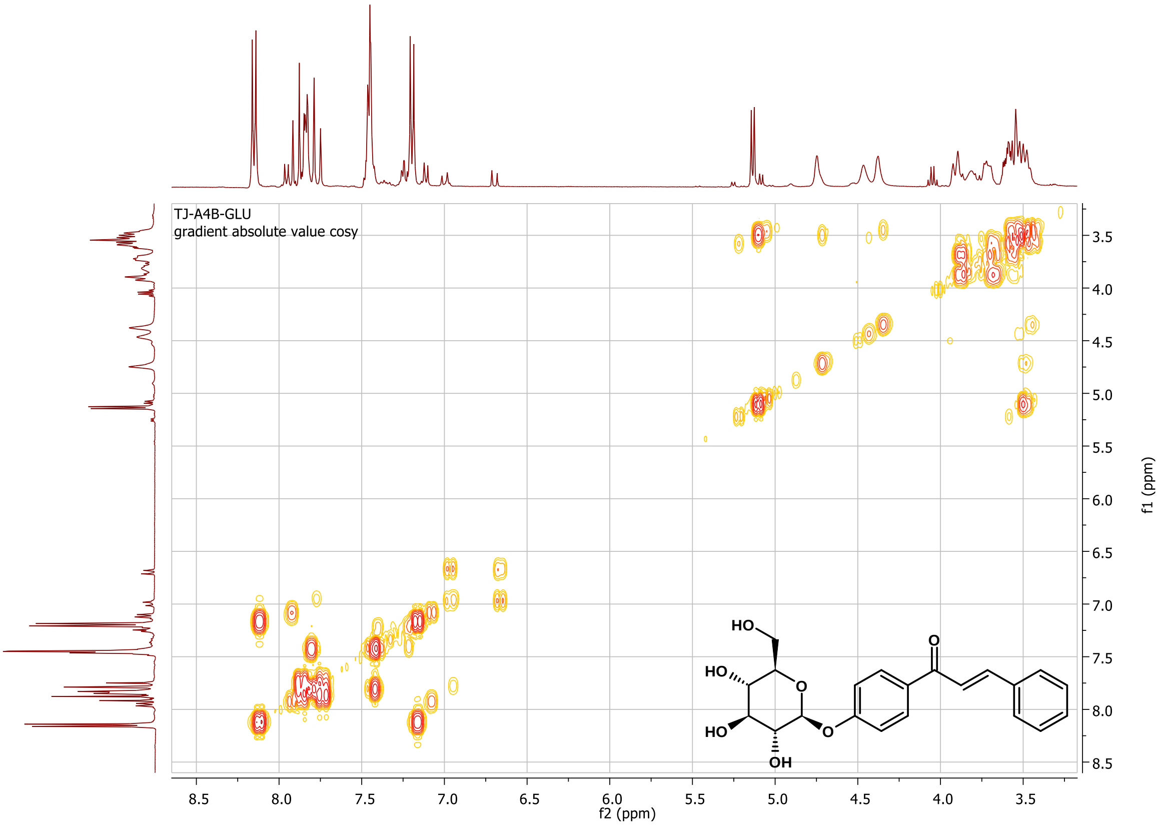


**Figure S28** COSY NMR spectrum of *trans*-4′-*O*-β-D-(glucopyranosyl)-chalcone (600MHz; Acetone-d$_6$)

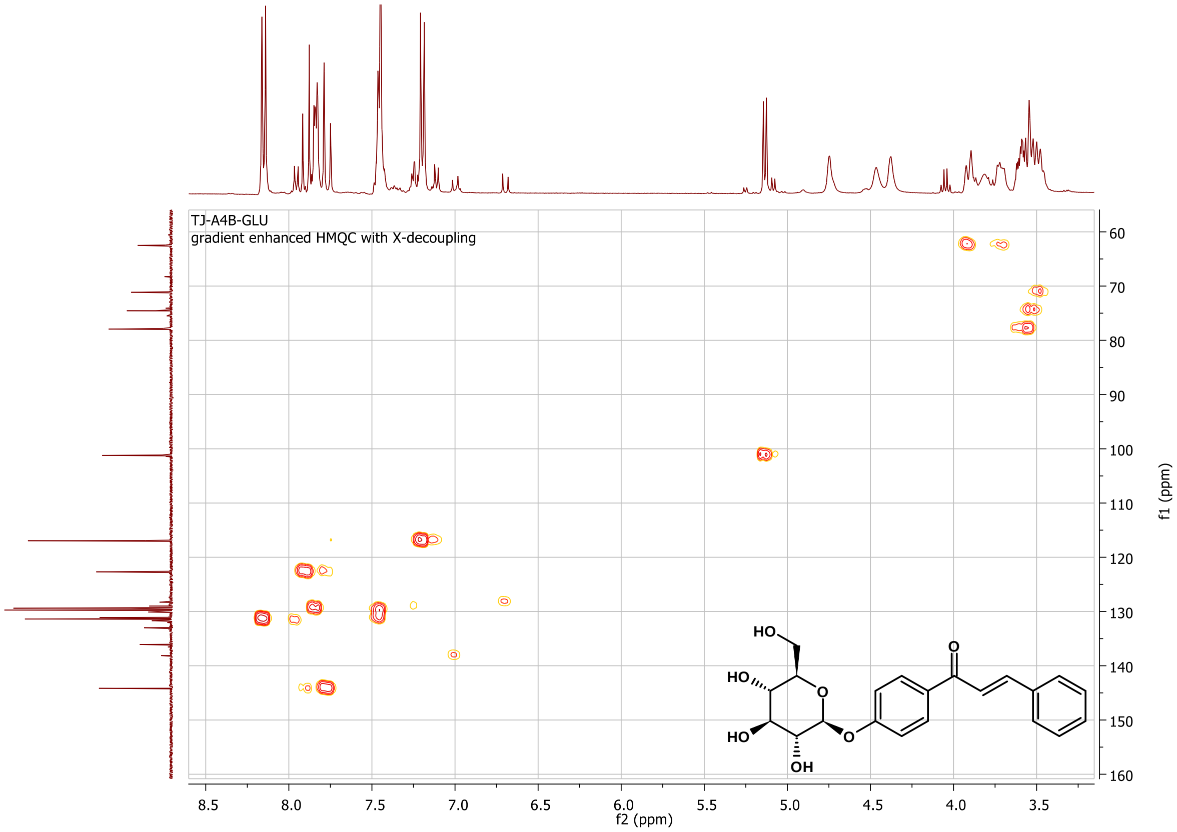


**Figure S29** HMQC NMR spectrum of *trans*-4′-*O*-β-D-(glucopyranosyl)-chalcone (600MHz; Acetone-d6)

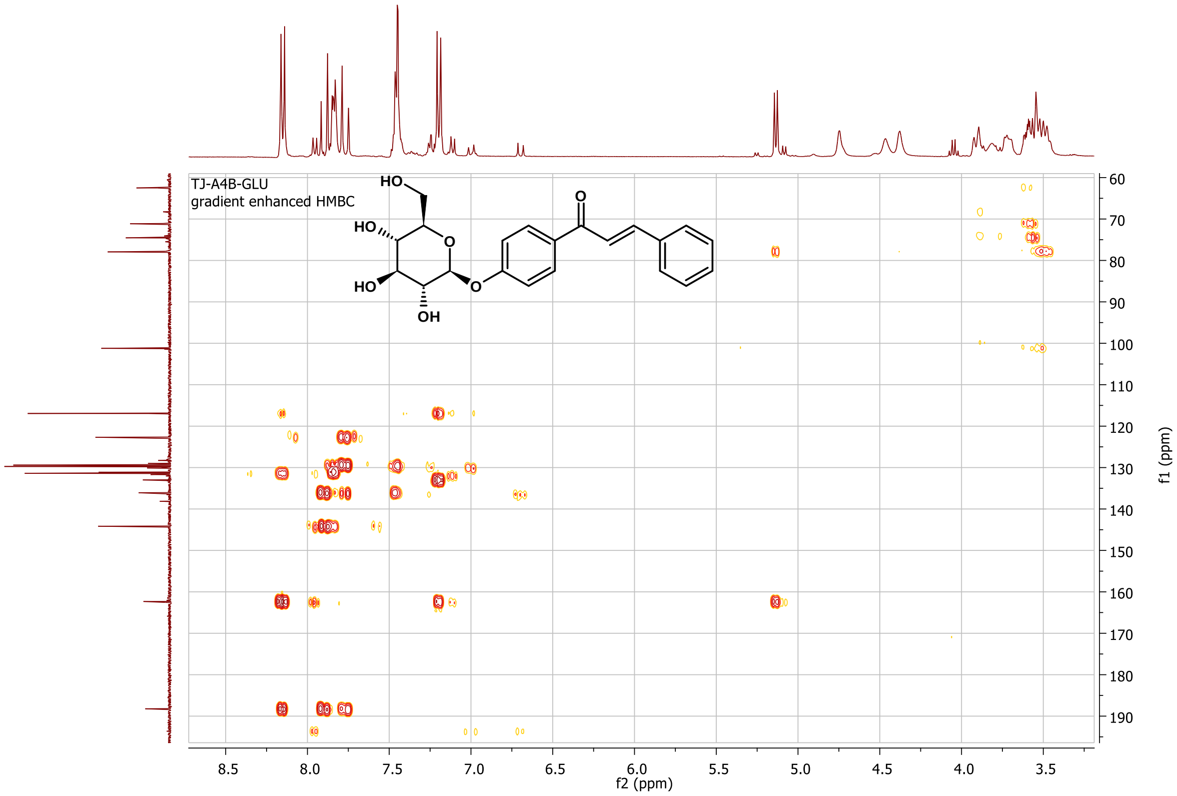


**Figure S30** HMBC NMR spectrum of *trans*-4′-*O*-β-D-(glucopyranosyl)-chalcone (600MHz; Acetone-d6)

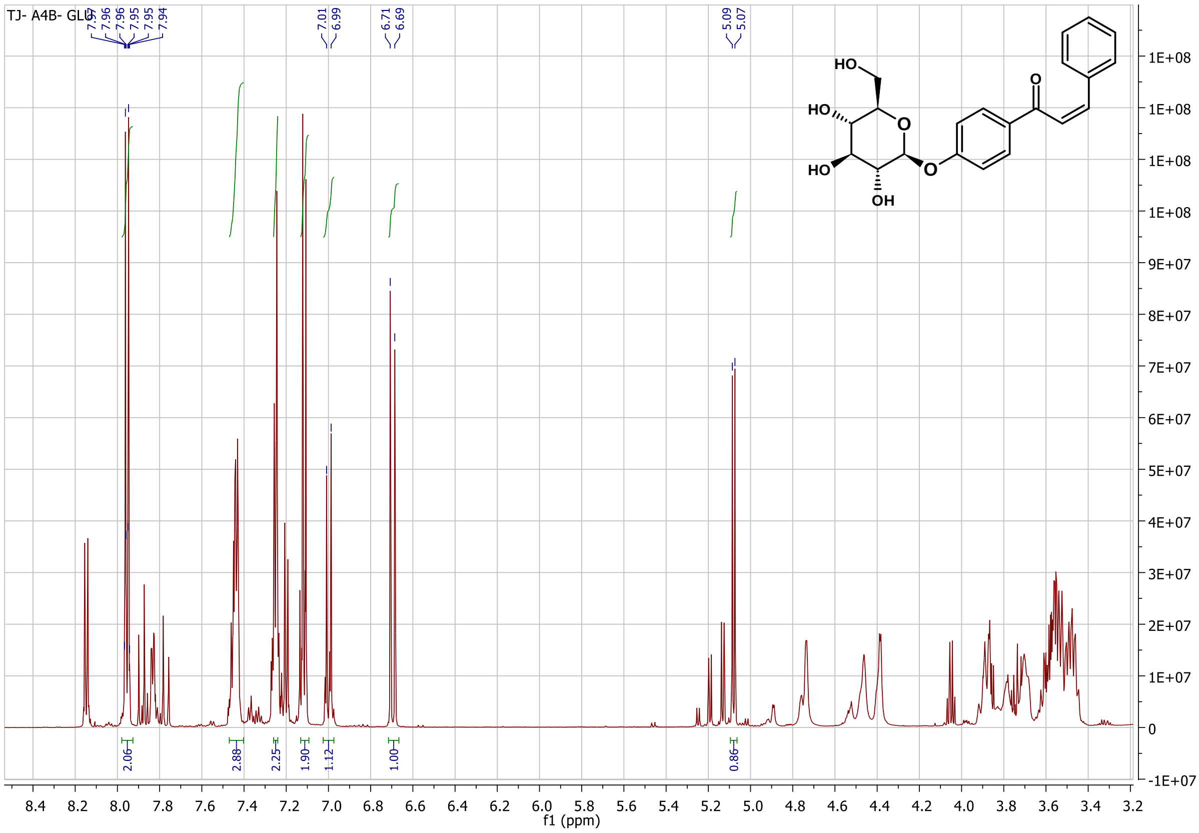


**Figure S31** $^{1}H$ NMR spectrum of *cis*-4′-*O*-β-D-(glucopyranosyl)-chalcone (600MHz; Acetone-$d_6$)

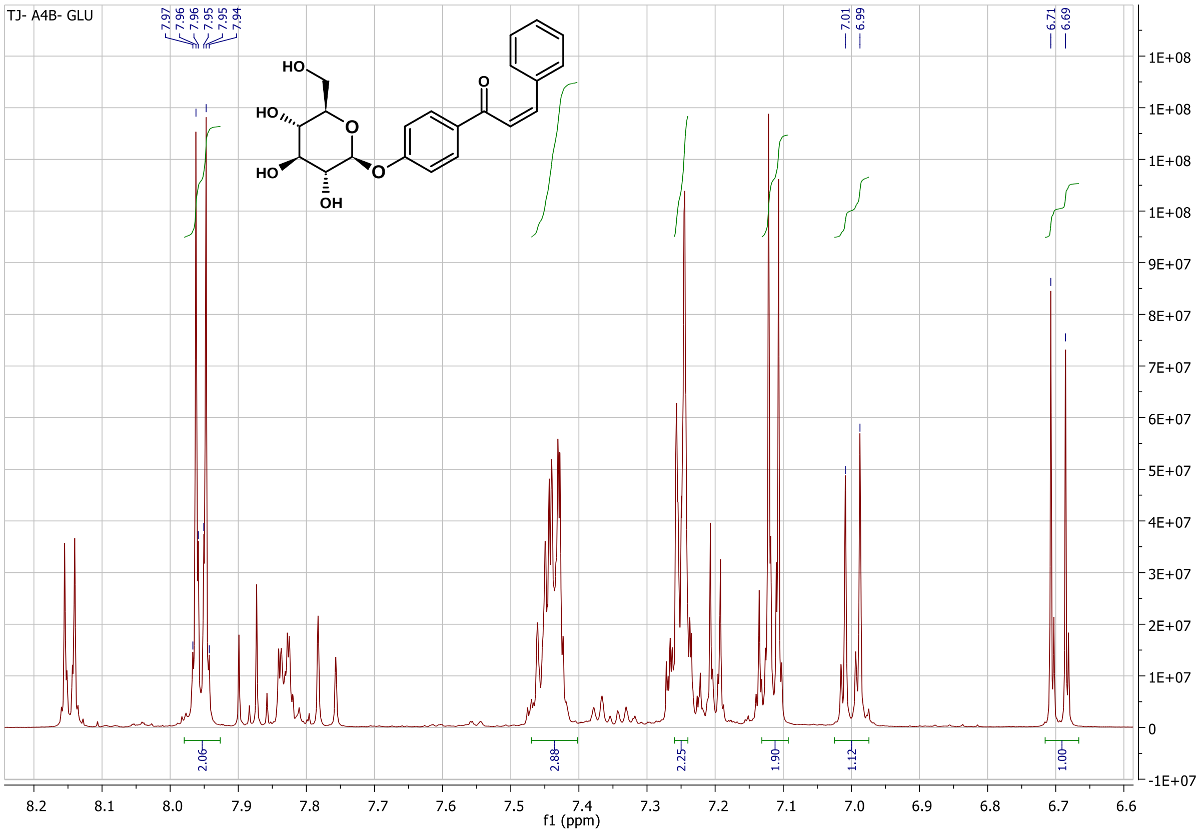


**Figure S32** Flavonoid part of $^{1}H$ NMR spectra of *cis*-4′-*O*-β-D-(glucopyranosyl)-chalcone (600MHz; Acetone-$d_6$)

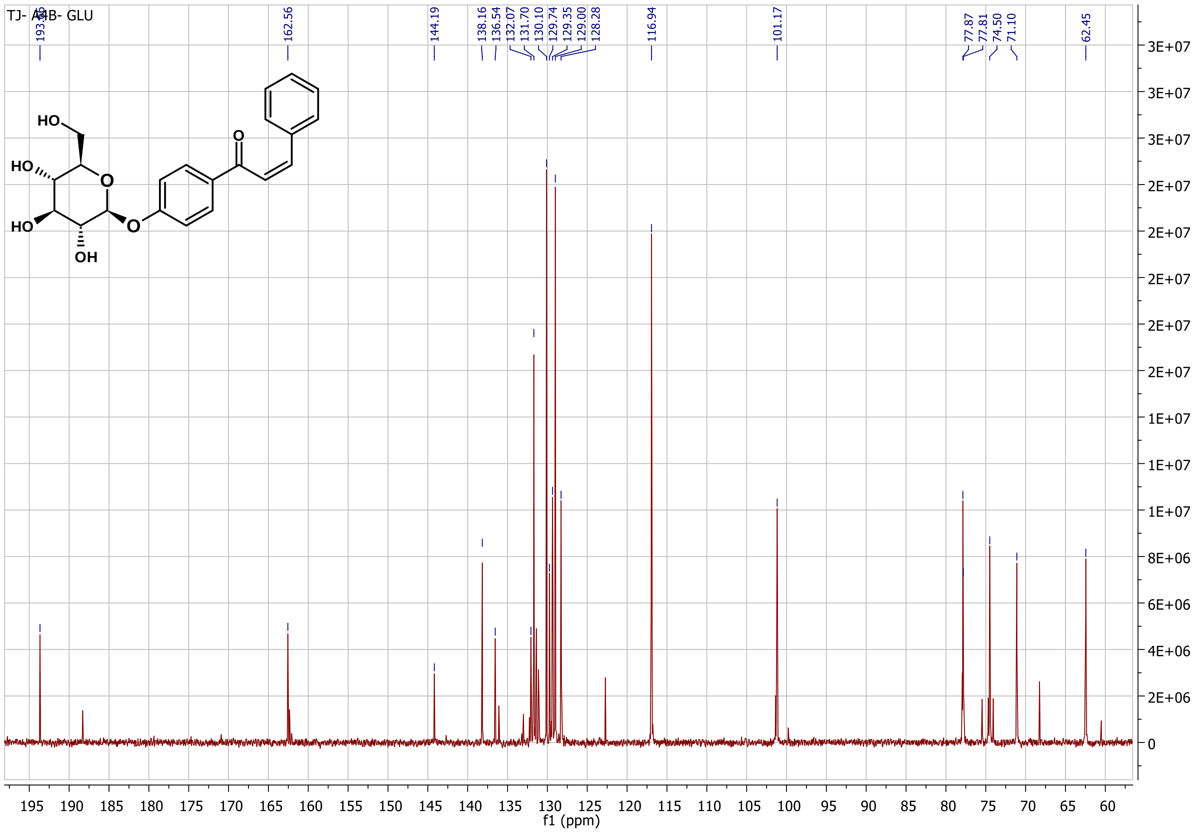


**Figure S33** $^{13}$C NMR spectra of *cis*-4′-*O*-β-D-(glucopyranosyl)-chalcone (151MHz; Acetone-d6)

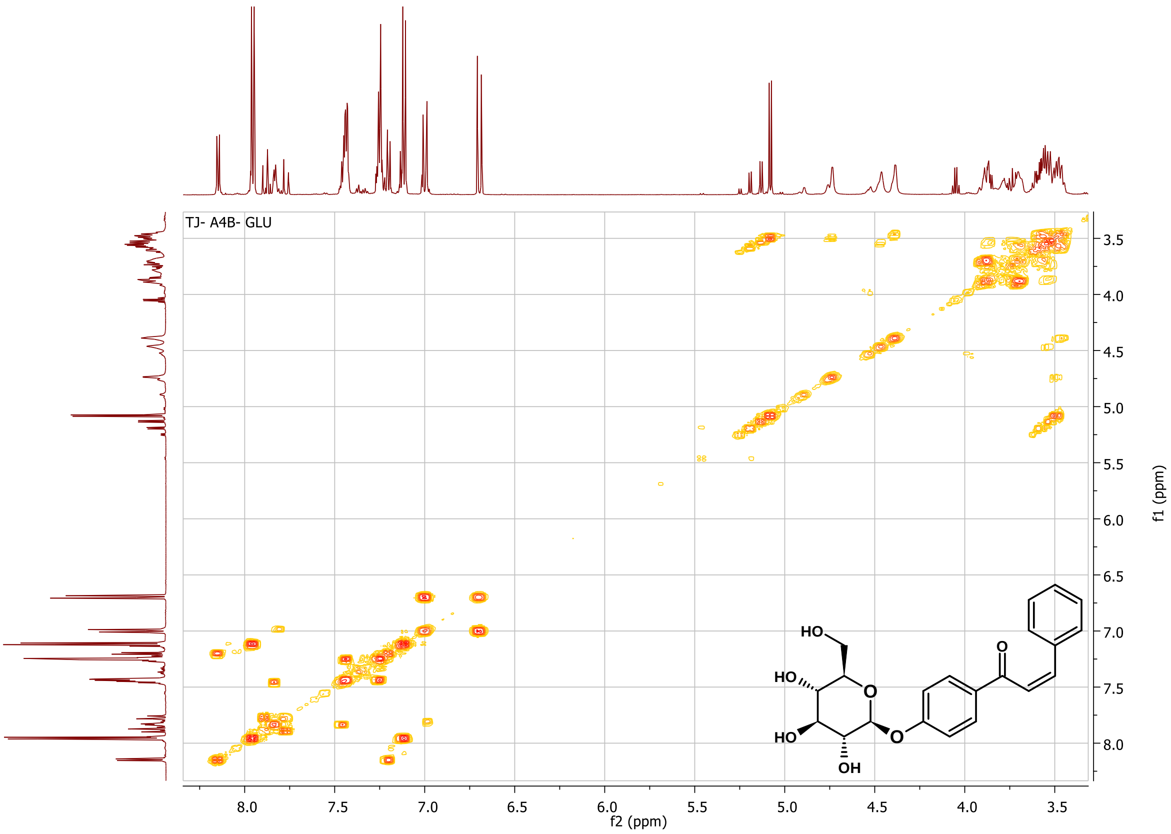


**Figure S34** COSY NMR spectra of *cis*-4′-*O*-β-D-(glucopyranosyl)-chalcone (600MHz; Acetone-d6)

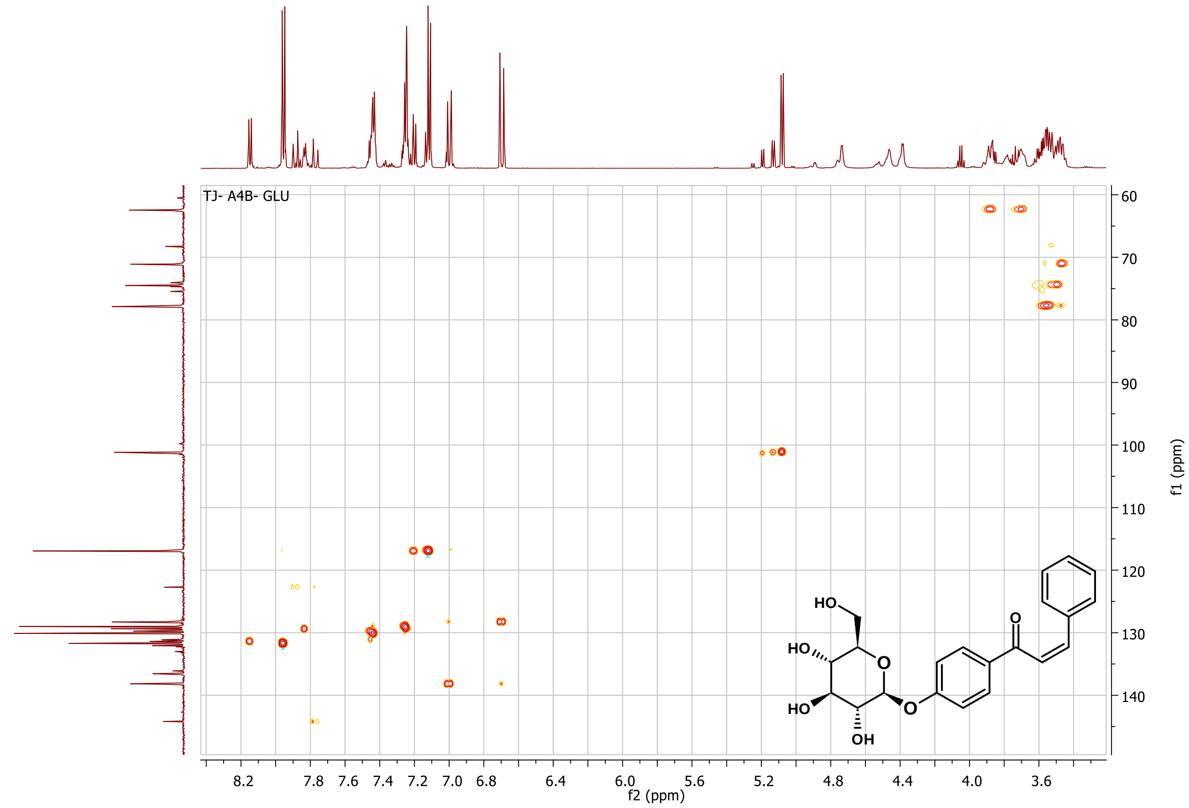


**Figure S35** HMQC NMR spectra of *cis*-4′-*O*-β-D-(glucopyranosyl)-chalcone (600MHz; Acetone-d₆)

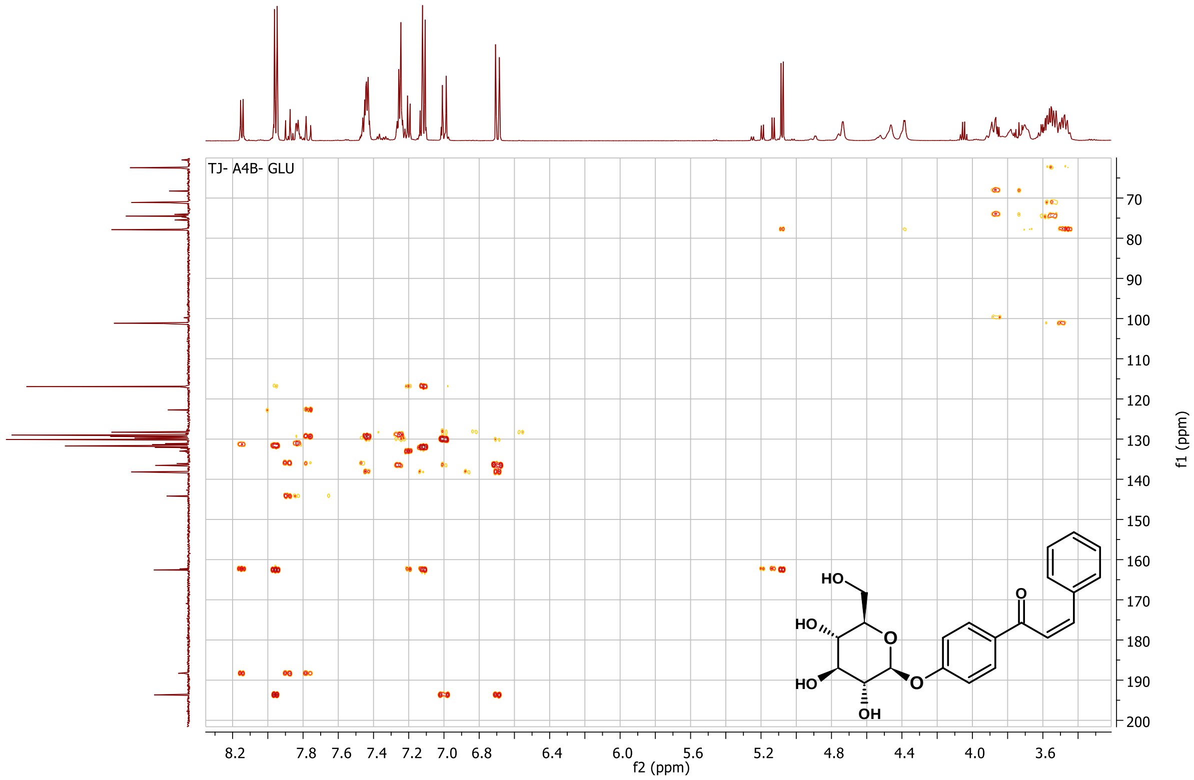


**Figure S36** HMBC NMR spectra of *cis*-4′-*O*-β-D-(glucopyranosyl)-chalcone (600MHz; Acetone-d₆)

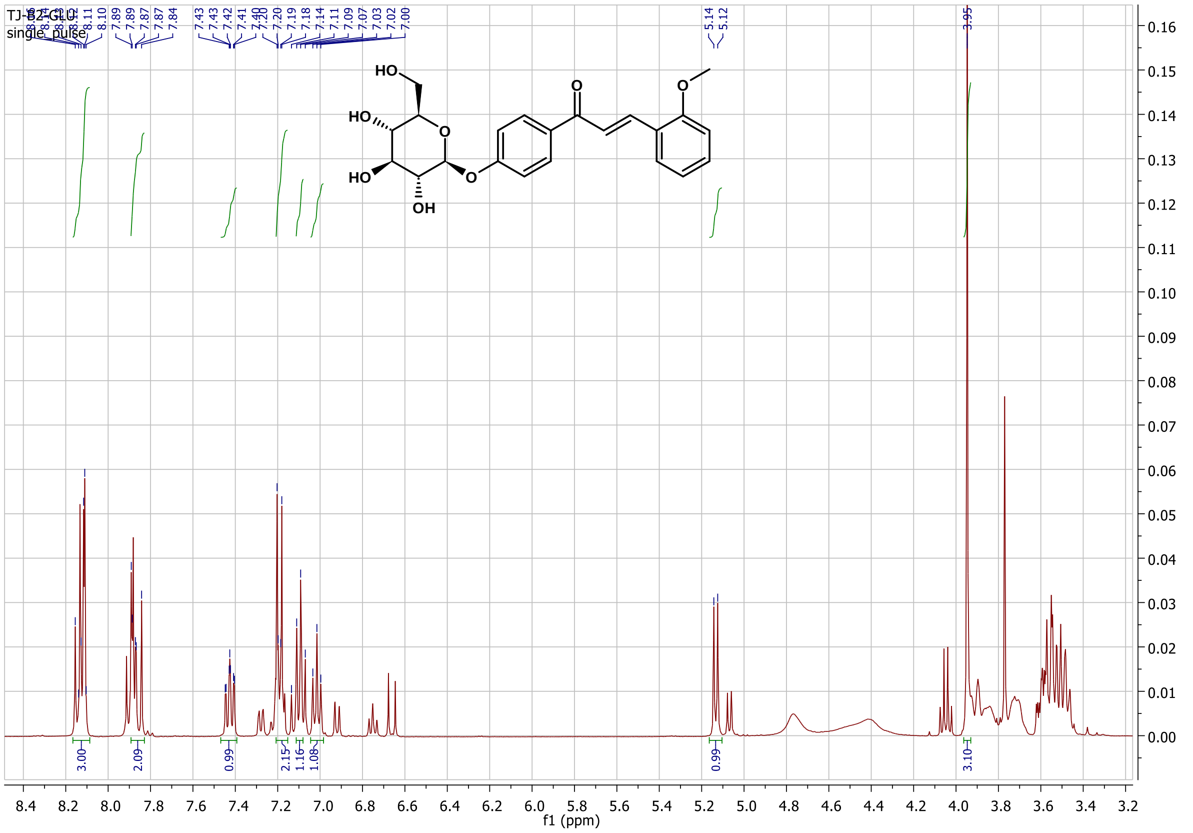

**Figure S37** $^{1}H$ NMR spectrum of *trans*-4′-*O*-β-D-(glucopyranosyl)-2-methoxychalcone (600MHz; Acetone-$d_6$)

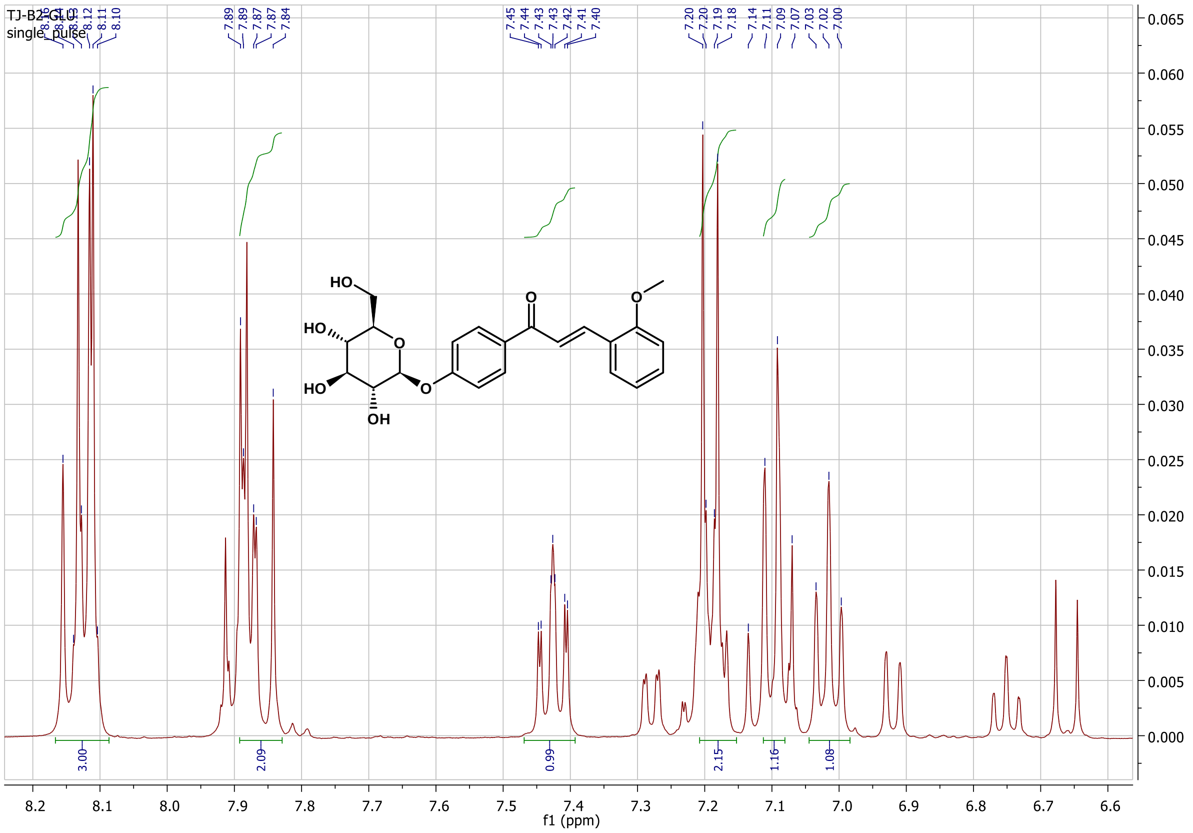

**Figure S38** Flavonoid part of $^{1}H$ NMR spectrum of *trans*-4′-*O*-β-D-(glucopyranosyl)-2-methoxychalcone (600MHz; Acetone-$d_6$)

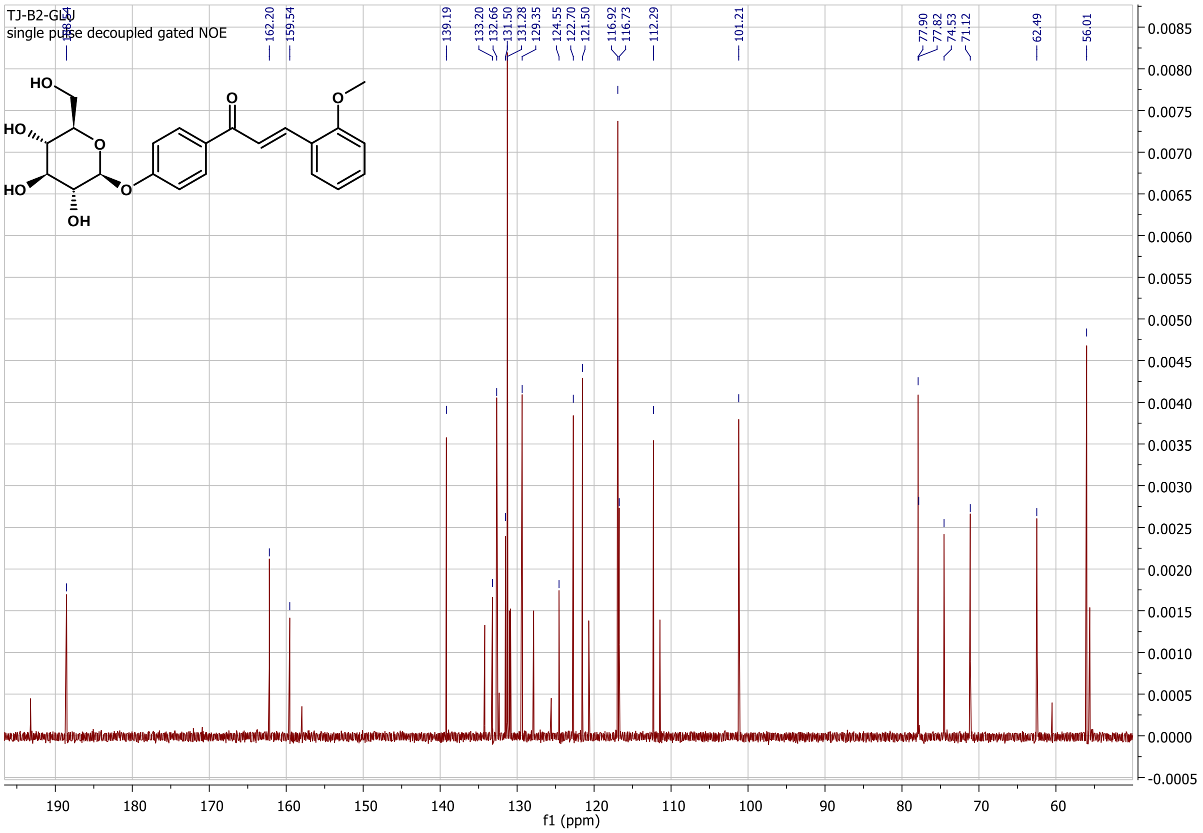


**Figure S39** $^{13}$C NMR spectrum of *trans*-4′-*O*-β-D-(glucopyranosyl)-2-methoxychalcone (151MHz; Acetone-d$_6$)

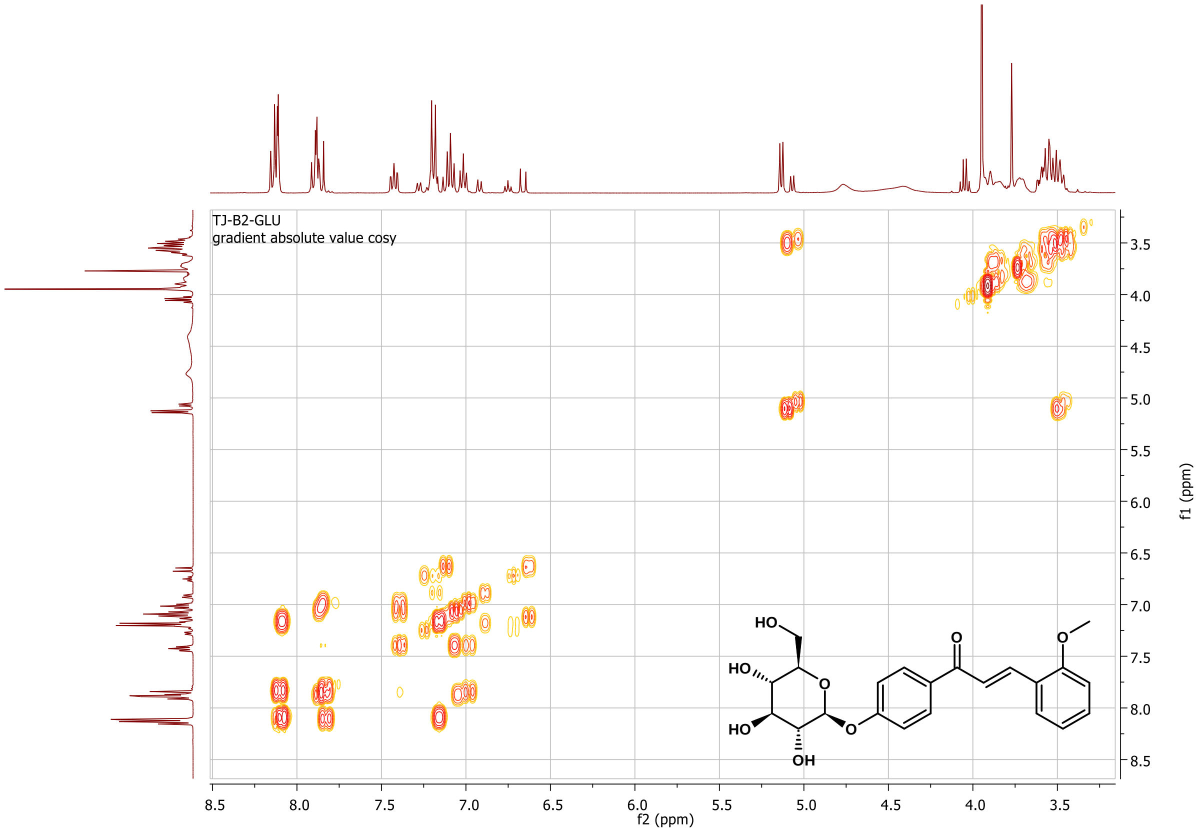


**Figure S40** COSY NMR spectrum of *trans*-4′-*O*-β-D-(glucopyranosyl)-chalcone (600MHz; Acetone-d$_6$)

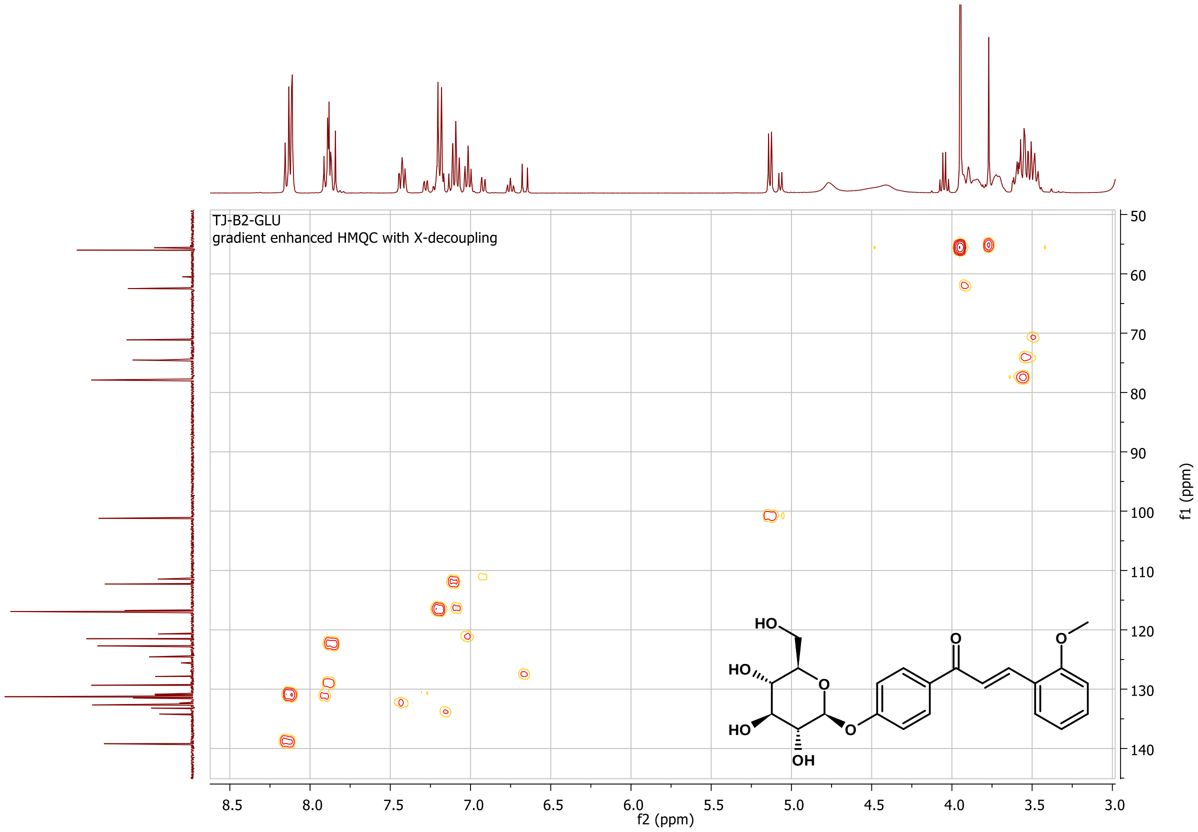


**Figure S41** HMQC NMR spectrum of *trans*-4′-*O*-β-D-(glucopyranosyl)-2-methoxychalcone (600MHz; Acetone-d₆)

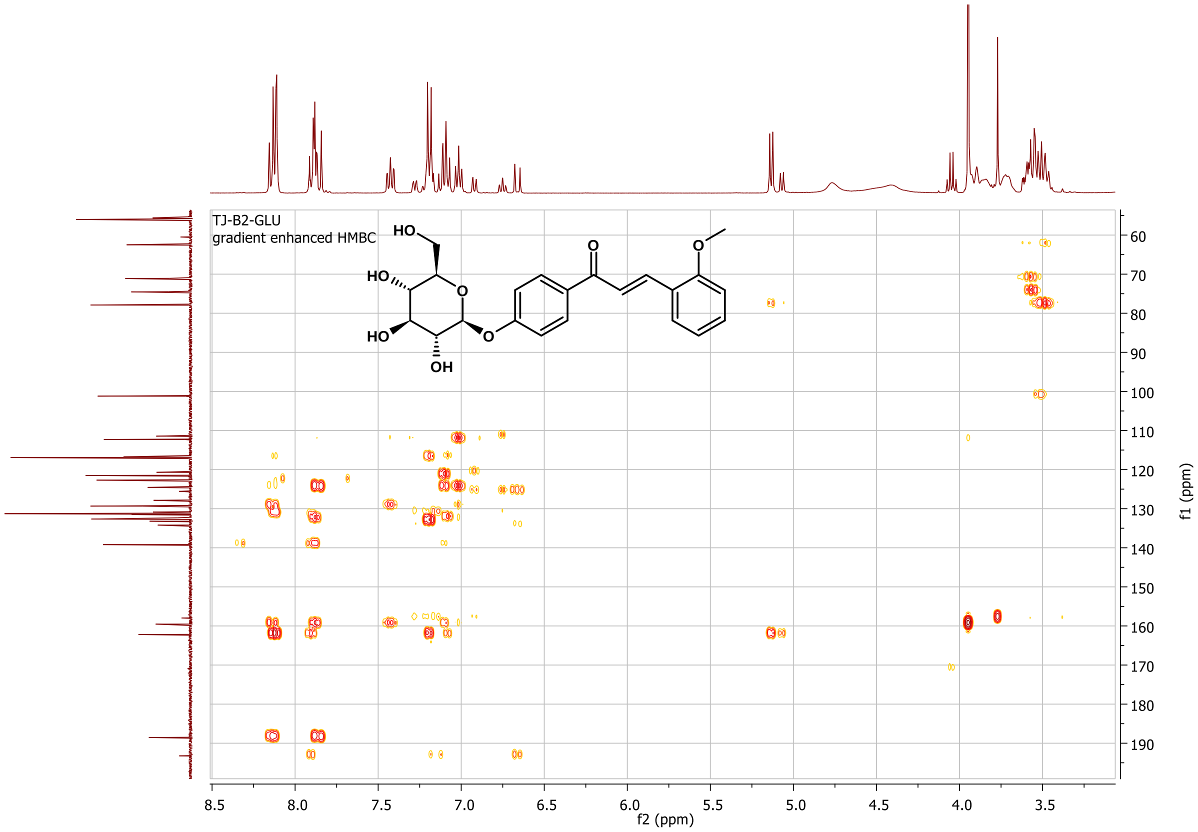


**Figure S42** HMBC NMR spectrum of *trans*-4′-*O*-β-D-(glucopyranosyl)-2-methoxychalcone (600MHz; Acetone-d₆)

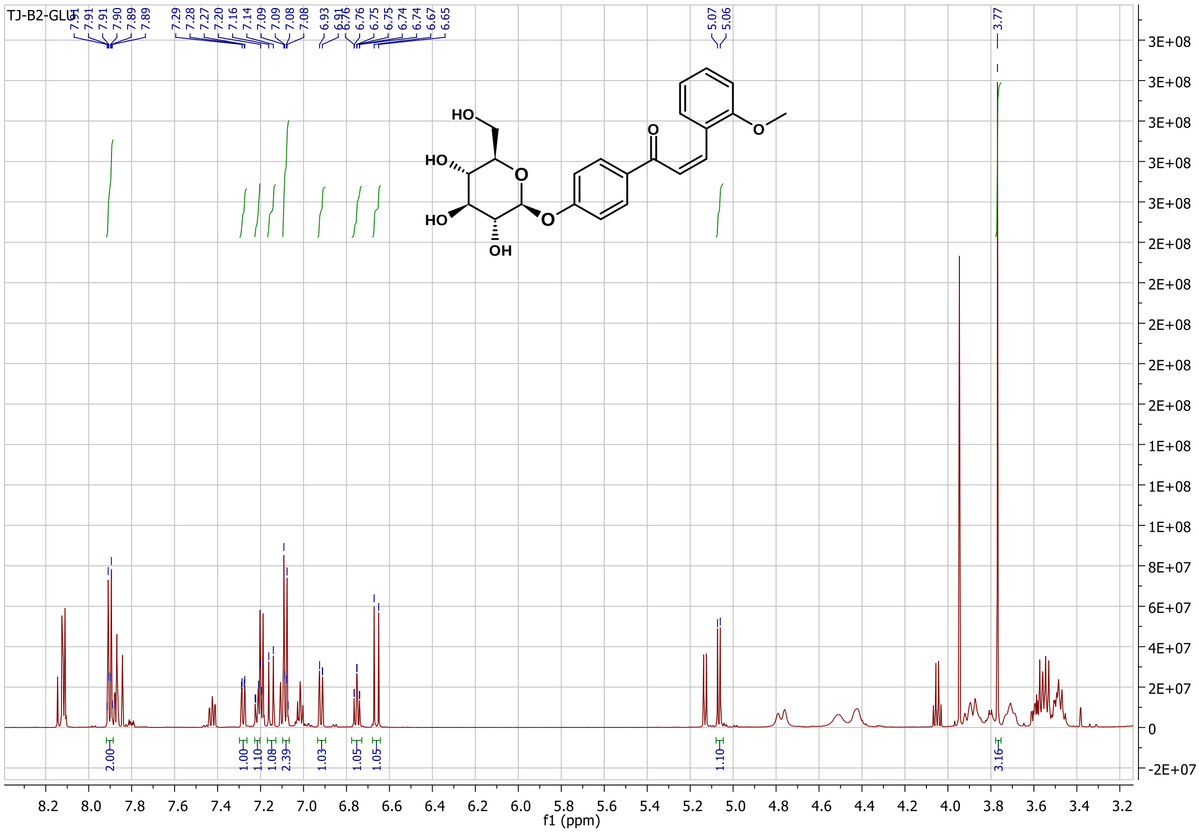


**Figure S43** $^{1}$H NMR spectrum of *cis*-4′-*O*-β-D-(glucopyranosyl)-2-methoxychalcone (600MHz; Acetone-$d_6$)

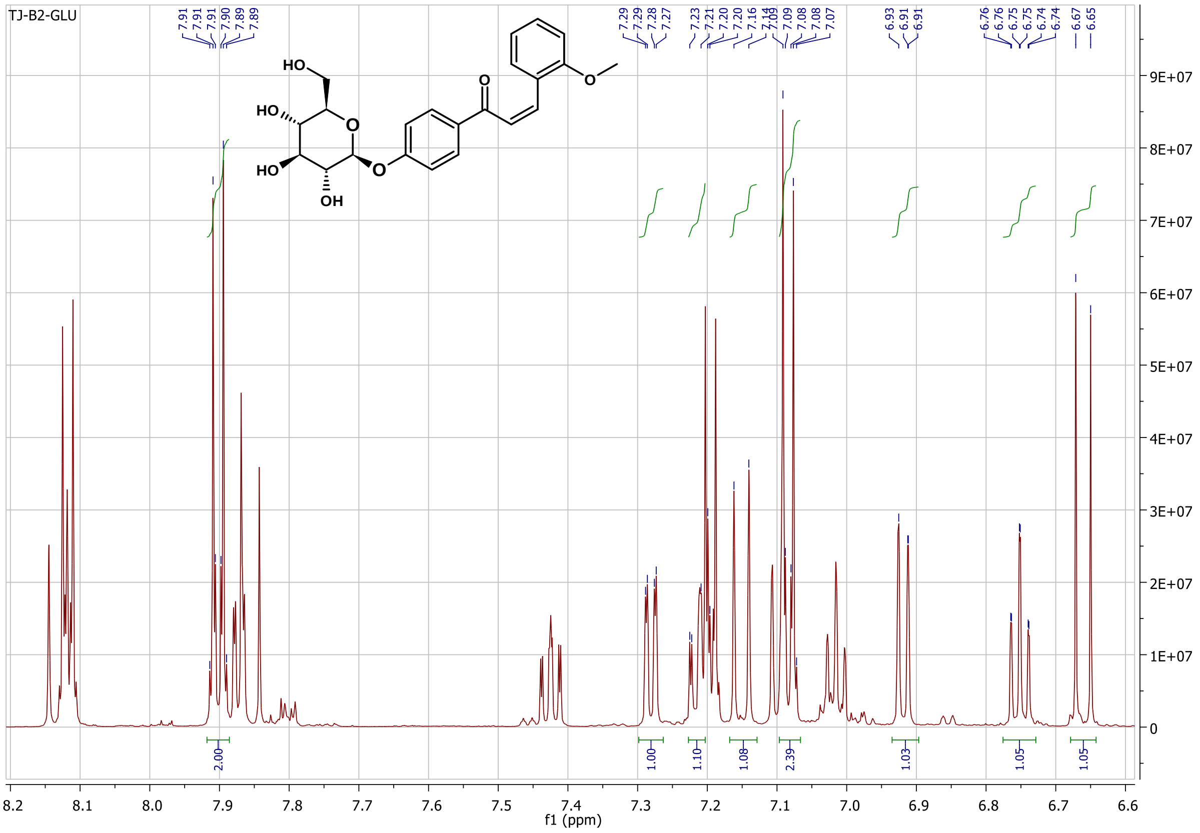


**Figure S44** Flavonoid part of $^{1}$H NMR spectrum of *cis*-4′-*O*-β-D-(glucopyranosyl)-2-methoxychalcone (600MHz; Acetone-$d_6$)

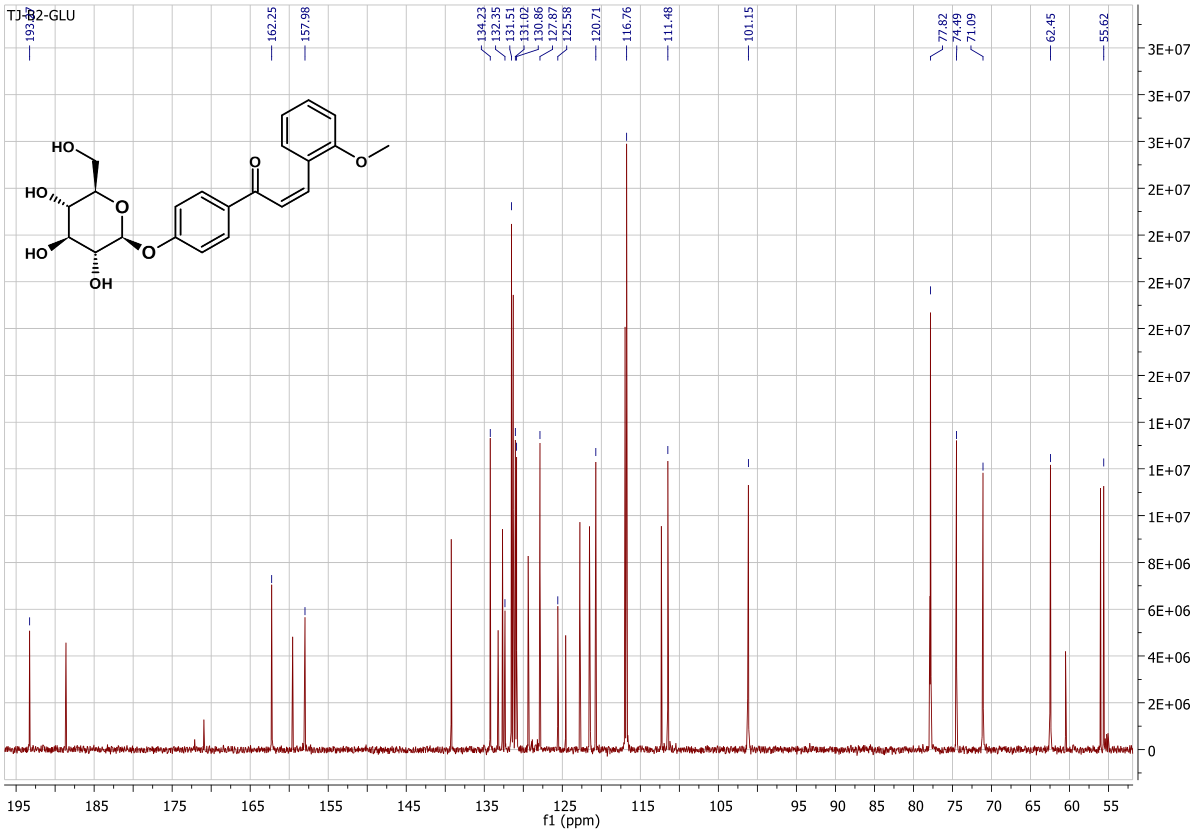

**Figure S45** $^{13}$C NMR spectrum of *cis*-4′-*O*-β-D-(glucopyranosyl)-2-methoxychalcone (151MHz; Acetone-d$_6$)

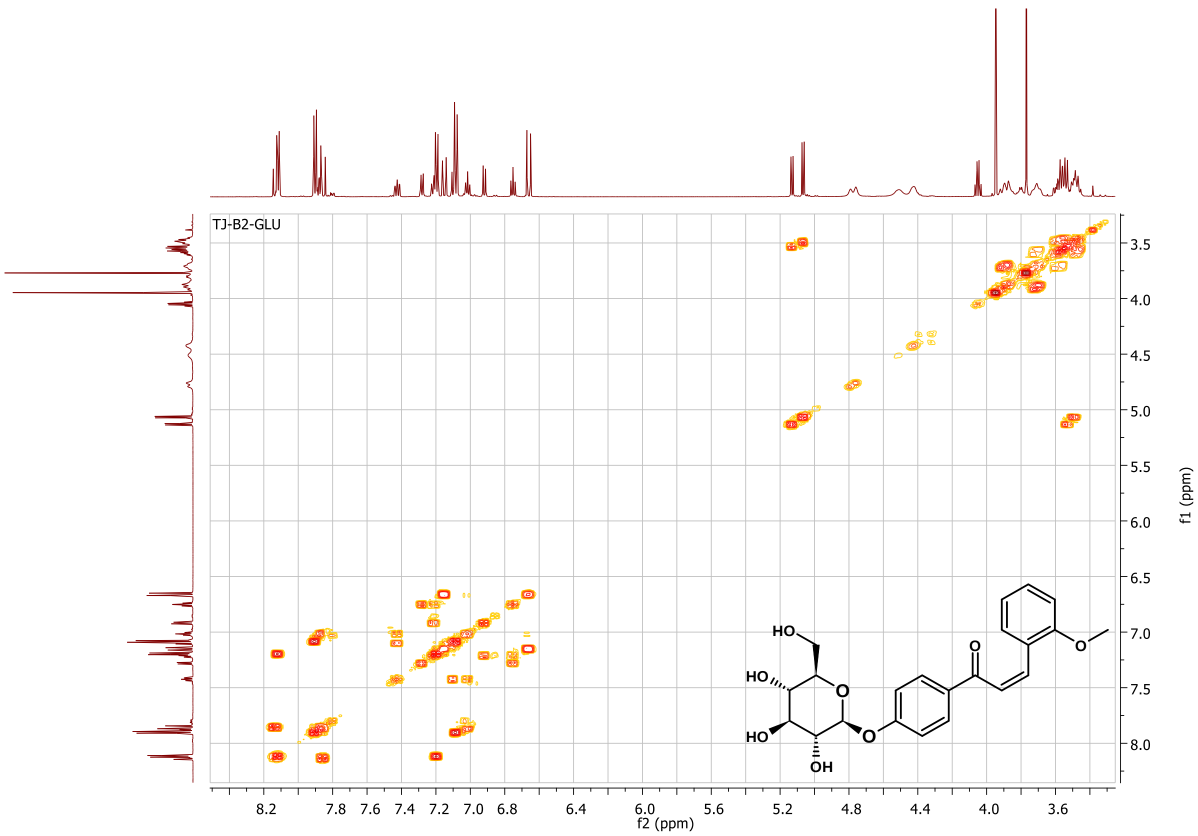

**Figure S46** COSY NMR spectrum of *cis*-4′-*O*-β-D-(glucopyranosyl)-2-methoxychalcone (600MHz; Acetone-d$_6$)

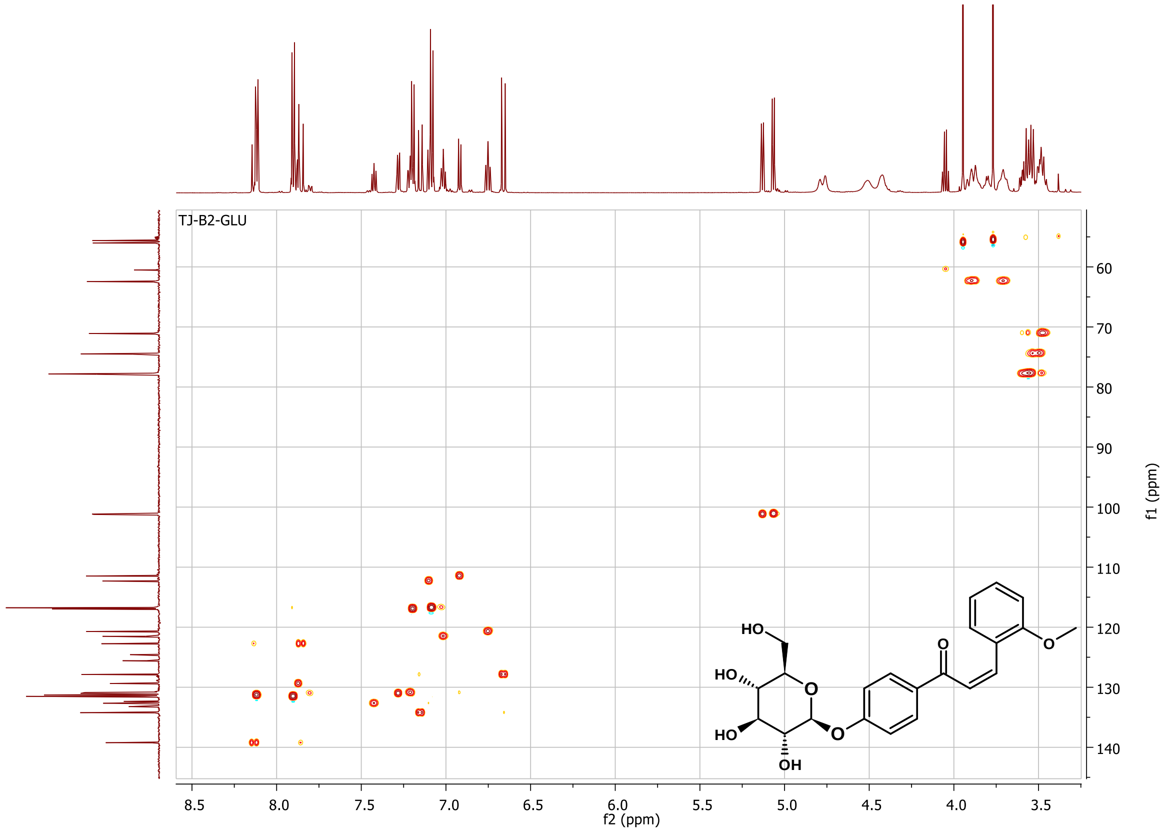


**Figure S47** HMQC NMR spectrum of *cis*-4′-*O*-β-D-(glucopyranosyl)-2-methoxychalcone (600MHz; Acetone-d$_6$)

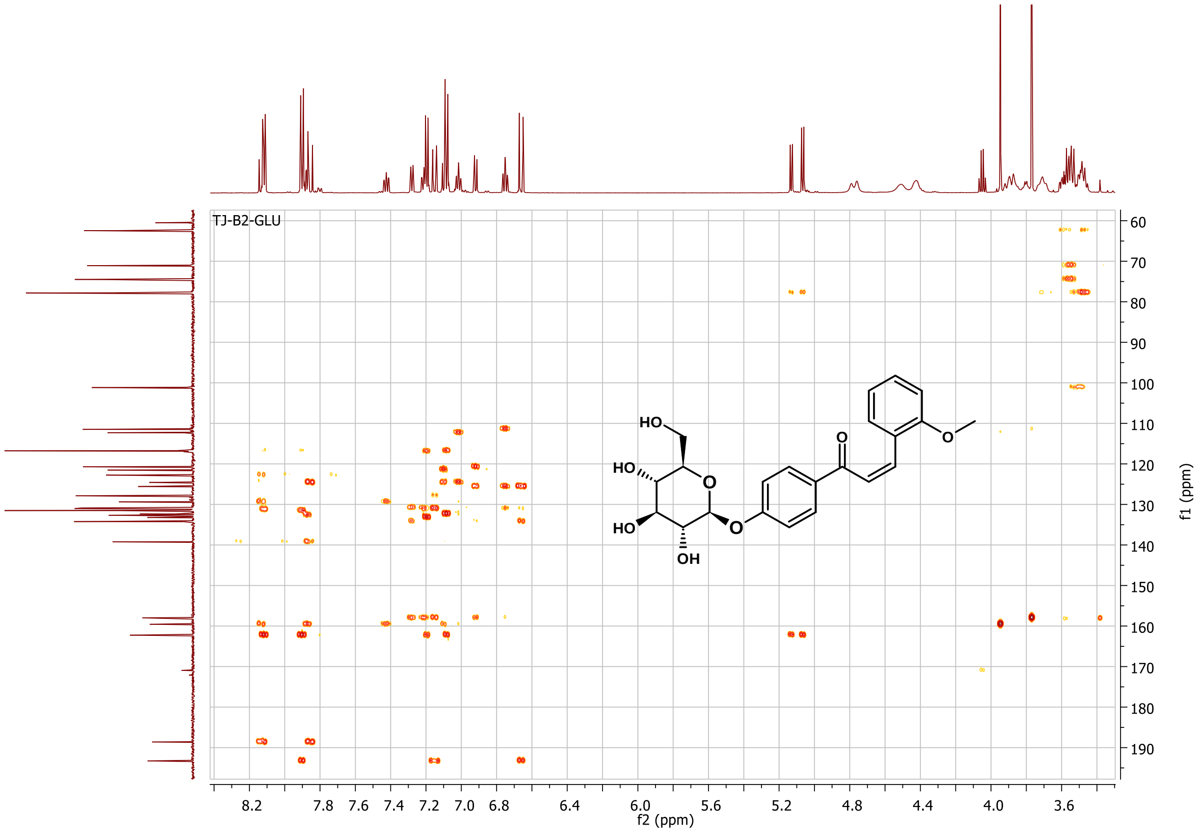


**Figure S48** HMBC NMR spectrum of *cis*-4′-*O*-β-D-(glucopyranosyl)-2-methoxychalcone (600MHz; Acetone-d$_6$)

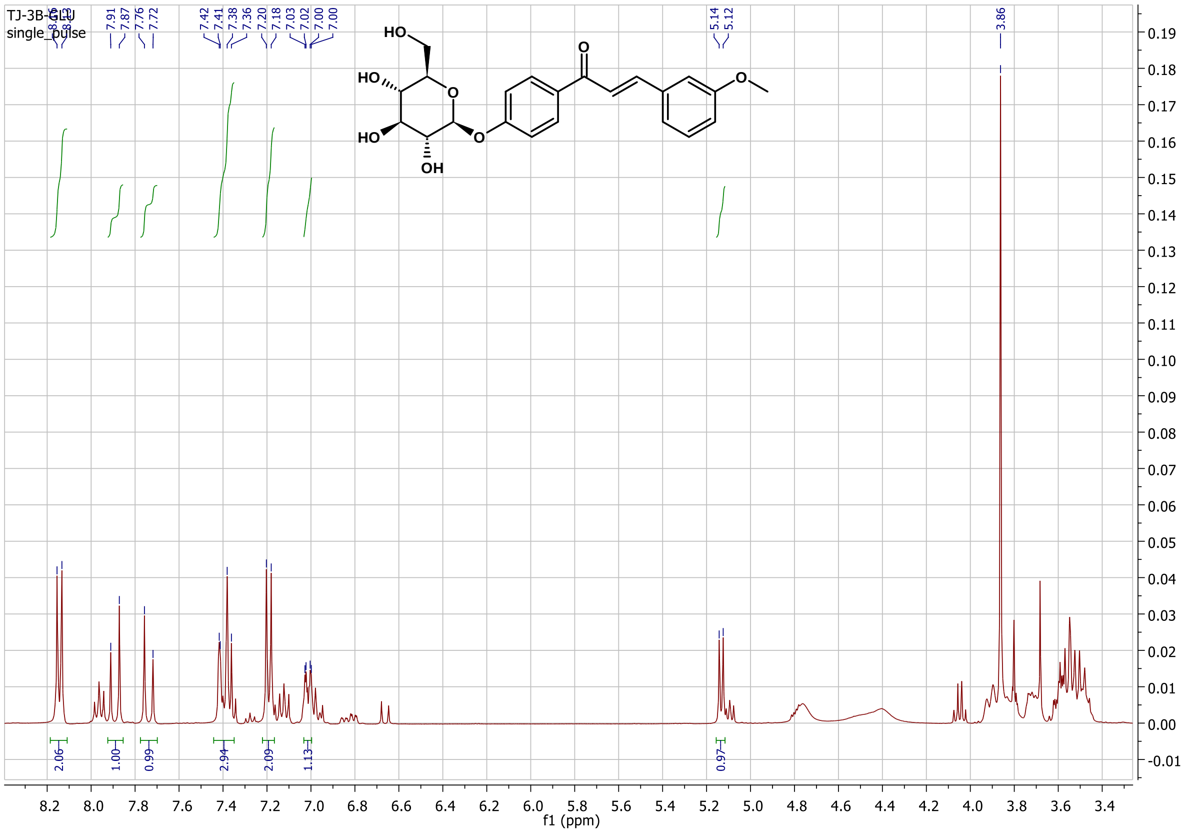

**Figure S49** $^{1}$H NMR spectrum of *trans*-4′-*O*-β-D-(glucopyranosyl)-3-methoxychalcone (600MHz; Acetone-$d_6$)

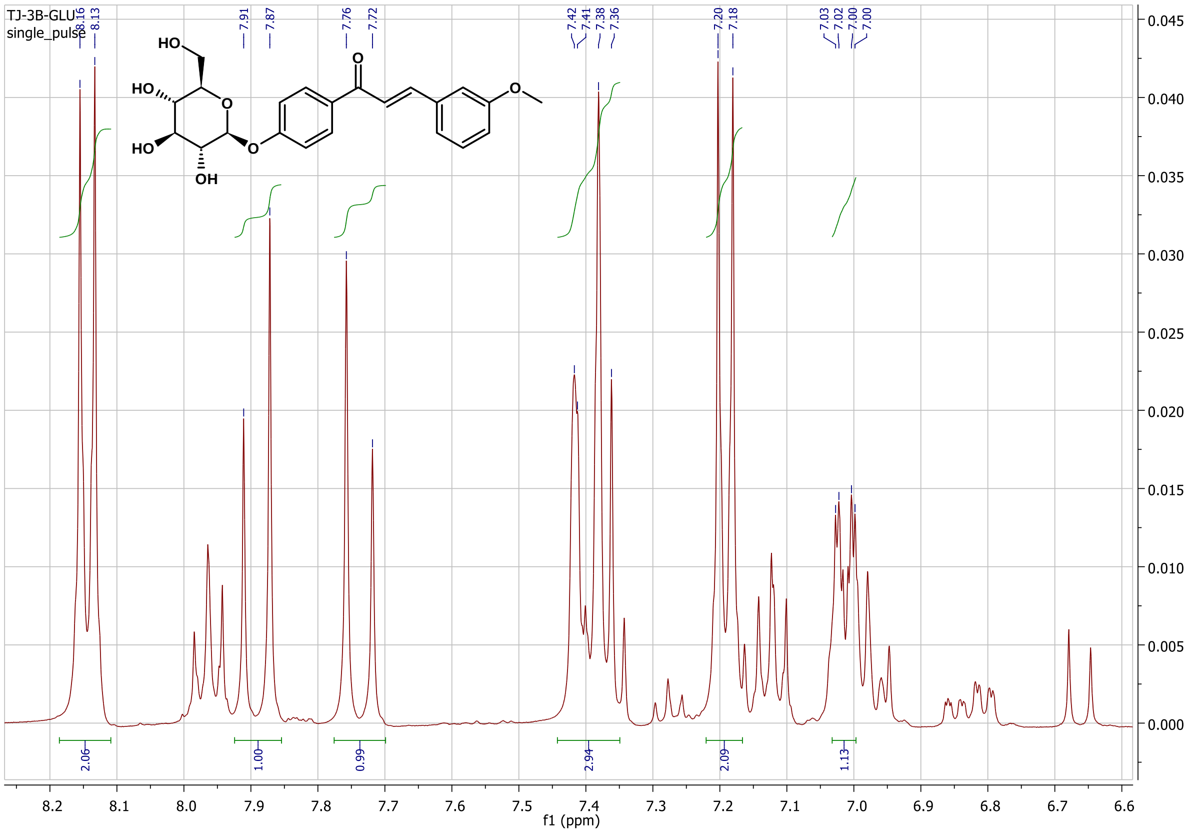

**Figure S50** Flavonoid part of $^{1}$H NMR spectrum of *trans*-4′-*O*-β-D-(glucopyranosyl)-3-methoxychalcone (600MHz; Acetone-$d_6$)

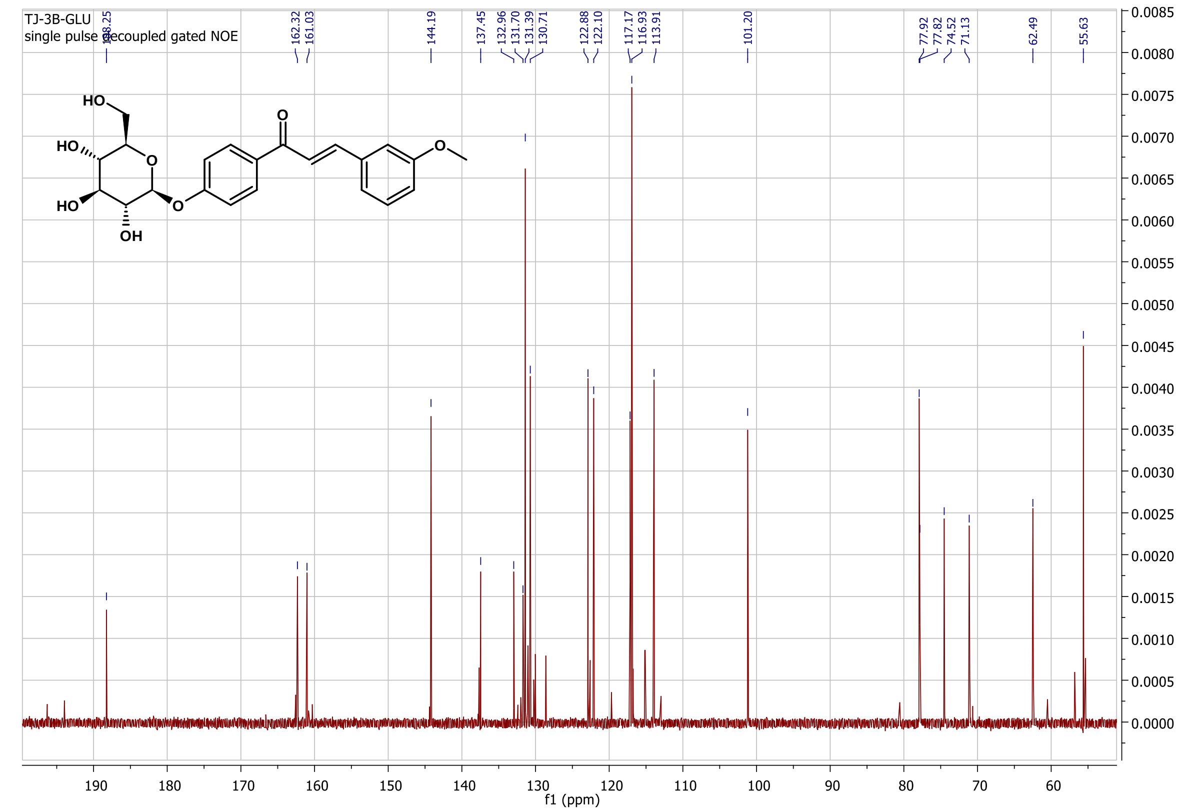


**Figure S51** $^{13}C$ NMR spectrum of *trans*-4′-*O*-β-D-(glucopyranosyl)-3-methoxychalcone (151MHz; Acetone-d$_6$)

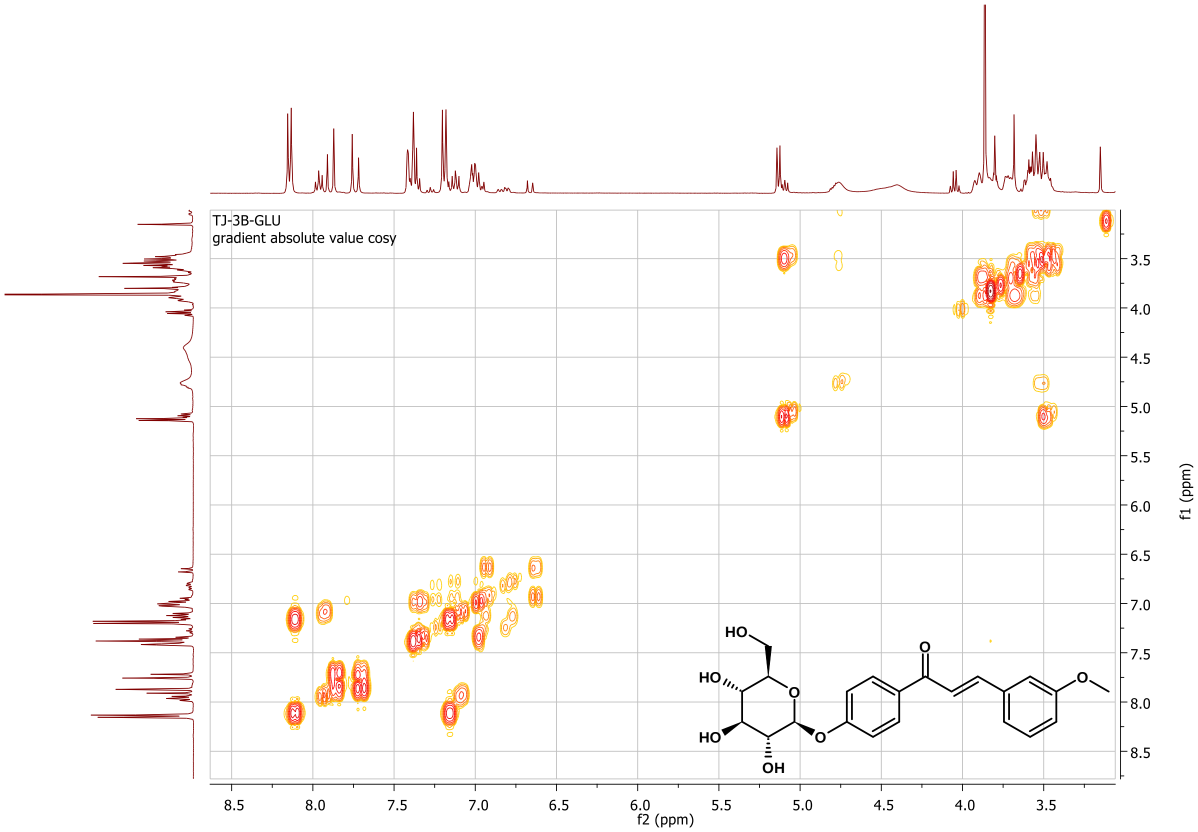


**Figure S52** COSY NMR spectrum of *trans*-4′-*O*-β-D-(glucopyranosyl)-3-methoxychalcone (600MHz; Acetone-d$_6$)

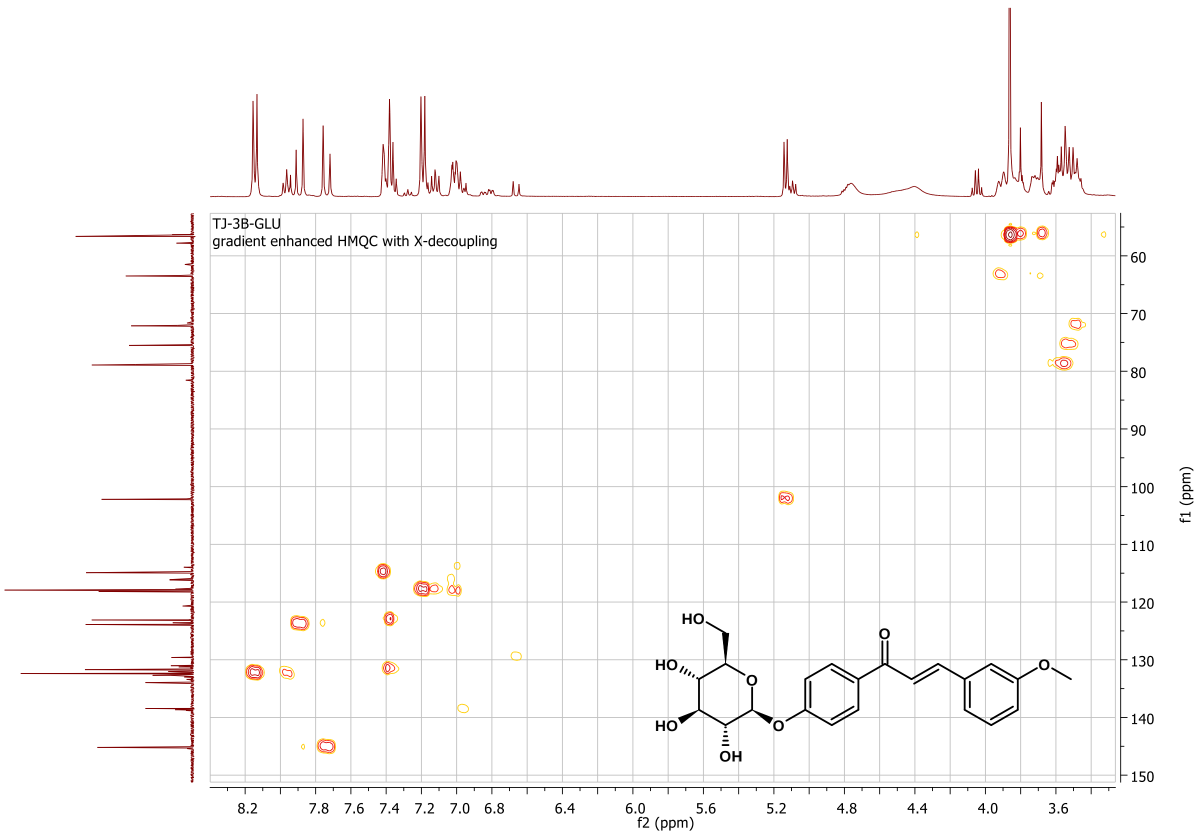


**Figure S53** HMQC NMR spectrum of *trans*-4′-*O*-β-D-(glucopyranosyl)-3-methoxychalcone (600MHz; Acetone-d6)

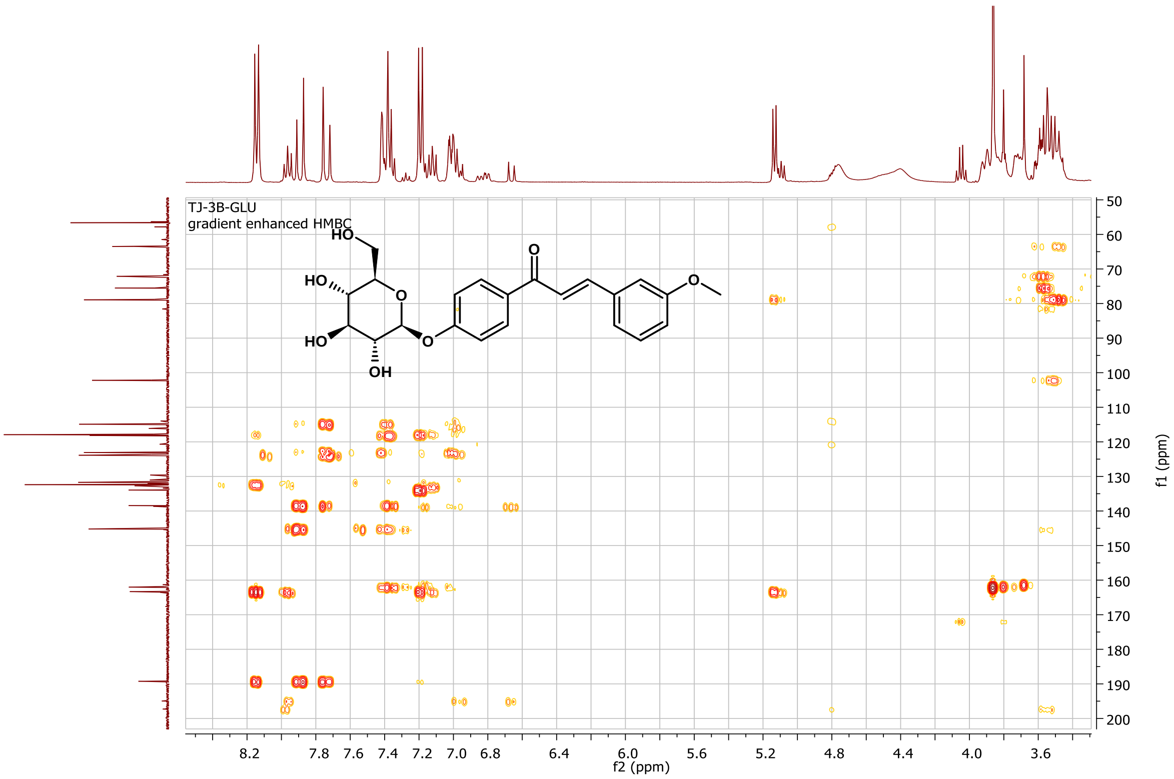


**Figure S54** HMBC NMR spectrum of *trans*-4′-*O*-β-D-(glucopyranosyl)-3-methoxychalcone (600MHz; Acetone-d6)

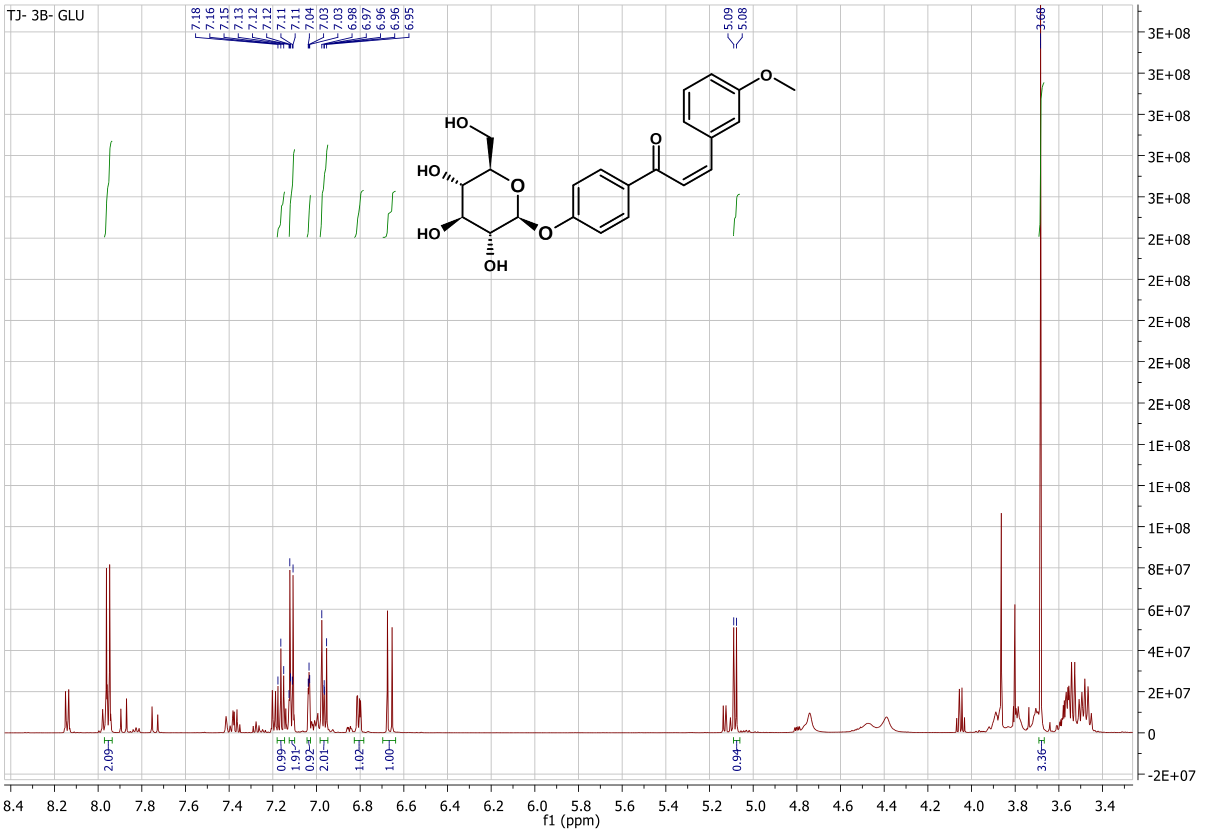


**Figure S55** ¹H NMR spectrum of *cis*-4′-*O*-β-D-(glucopyranosyl)-3-methoxychalcone (600MHz; Acetone-d₆)

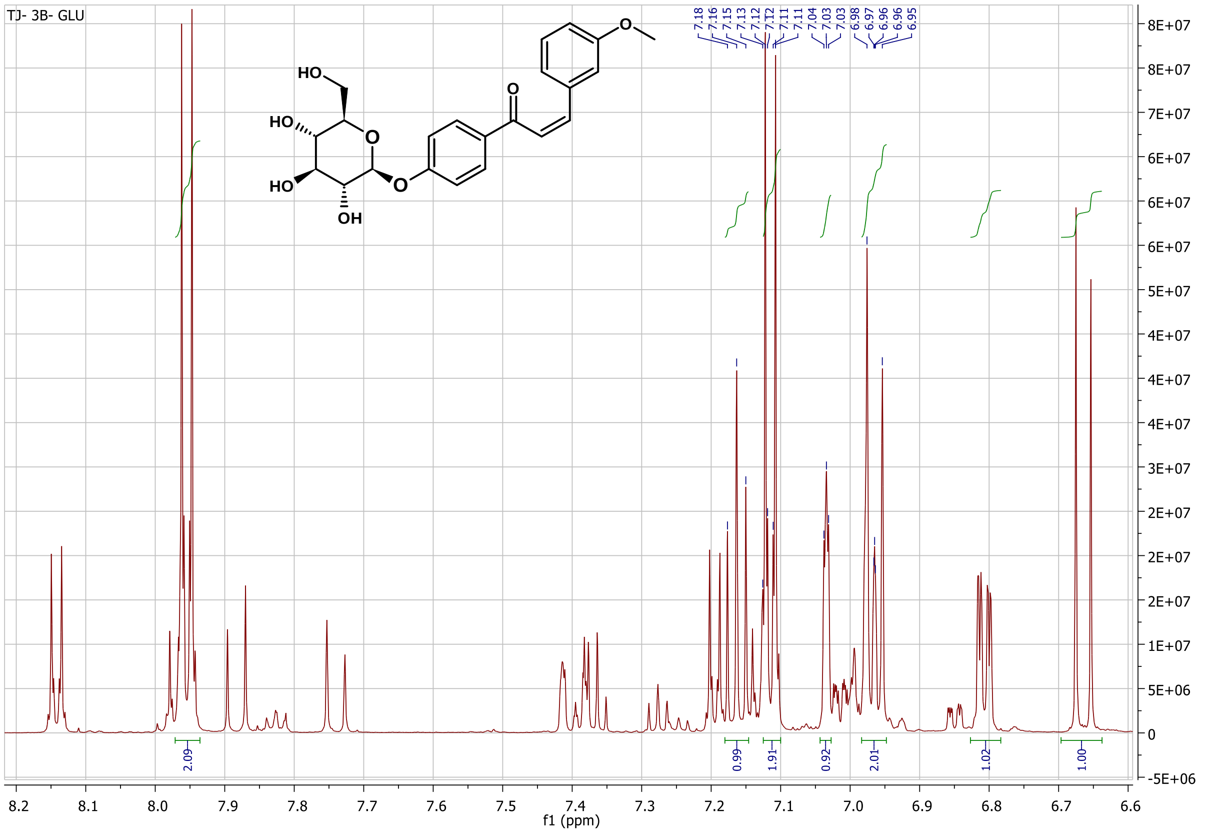


**Figure S56** Flavonoid part of ¹H NMR spectrum of *cis*-4′-*O*-β-D-(glucopyranosyl)-3-methoxychalcone (600MHz; Acetone-d₆)

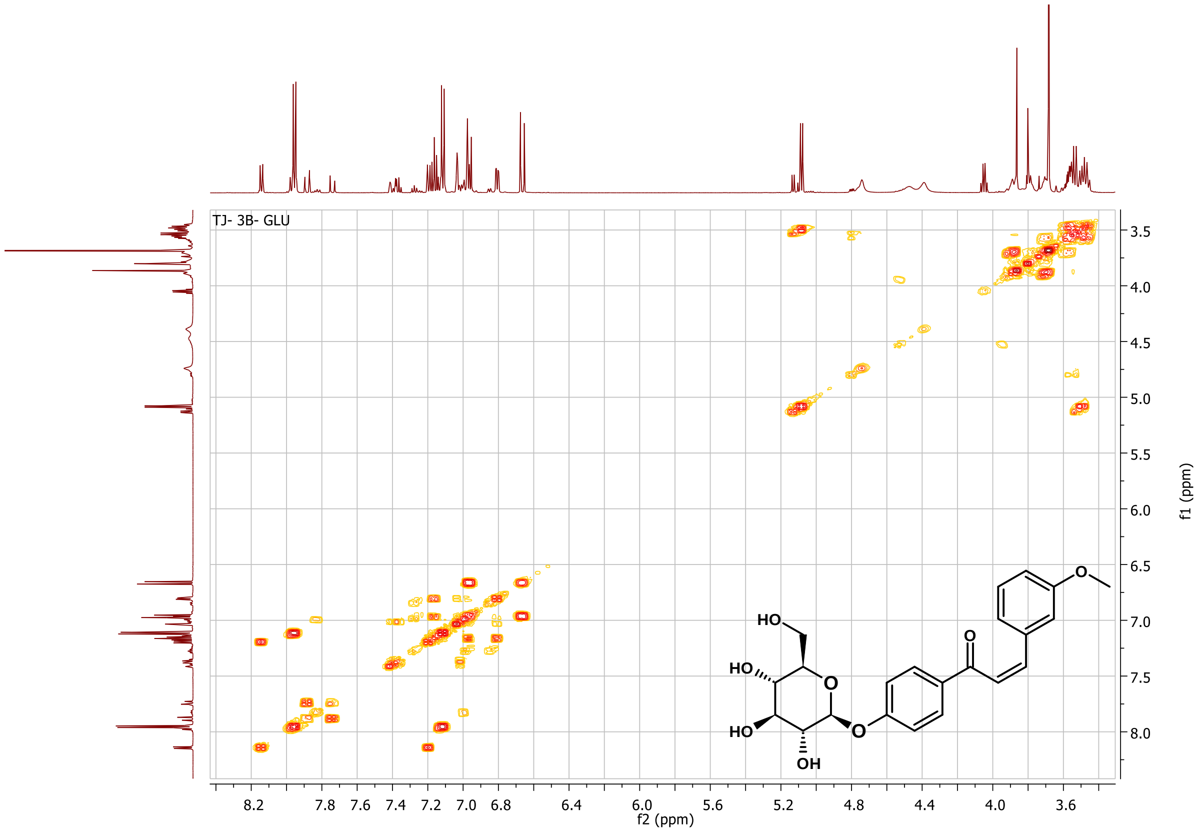


**Figure S57** COSY NMR spectrum of *cis*-4′-*O*-β-D-(glucopyranosyl)-3-methoxychalcone (600MHz; Acetone-d$_6$)

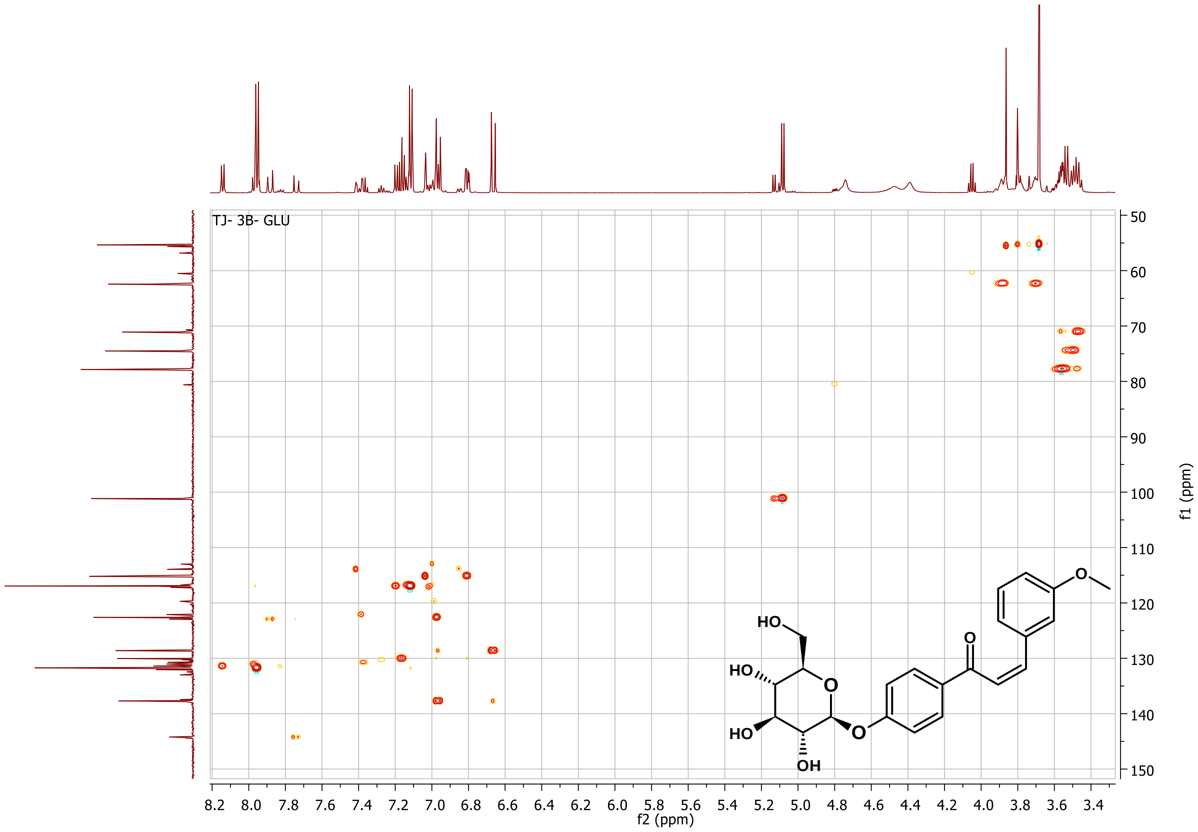


**Figure S58** HMQC NMR spectrum of *cis*-4′-*O*-β-D-(glucopyranosyl)-3-methoxychalcone (600MHz; Acetone-d$_6$)

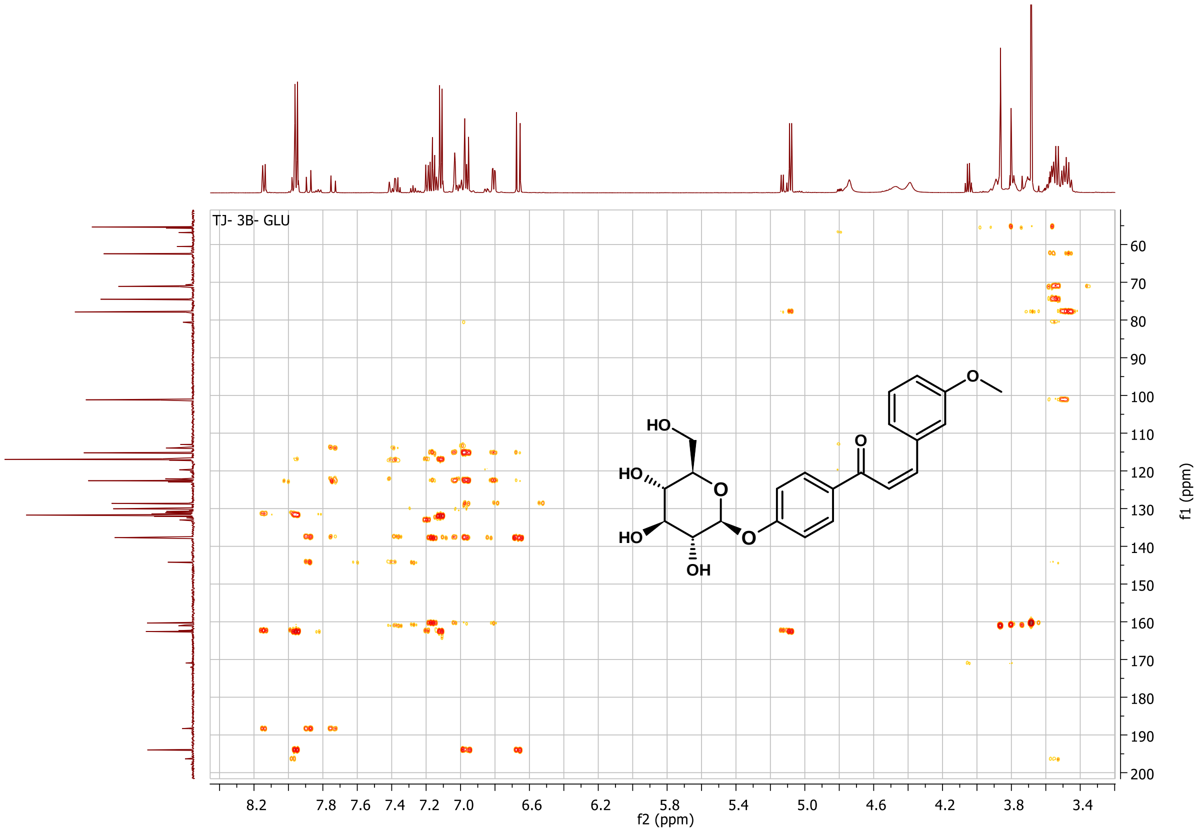


**Figure S59** HMBC NMR spectrum of *cis*-4′-*O*-β-D-(glucopyranosyl)-3-methoxychalcone (600MHz; Acetone-d6)

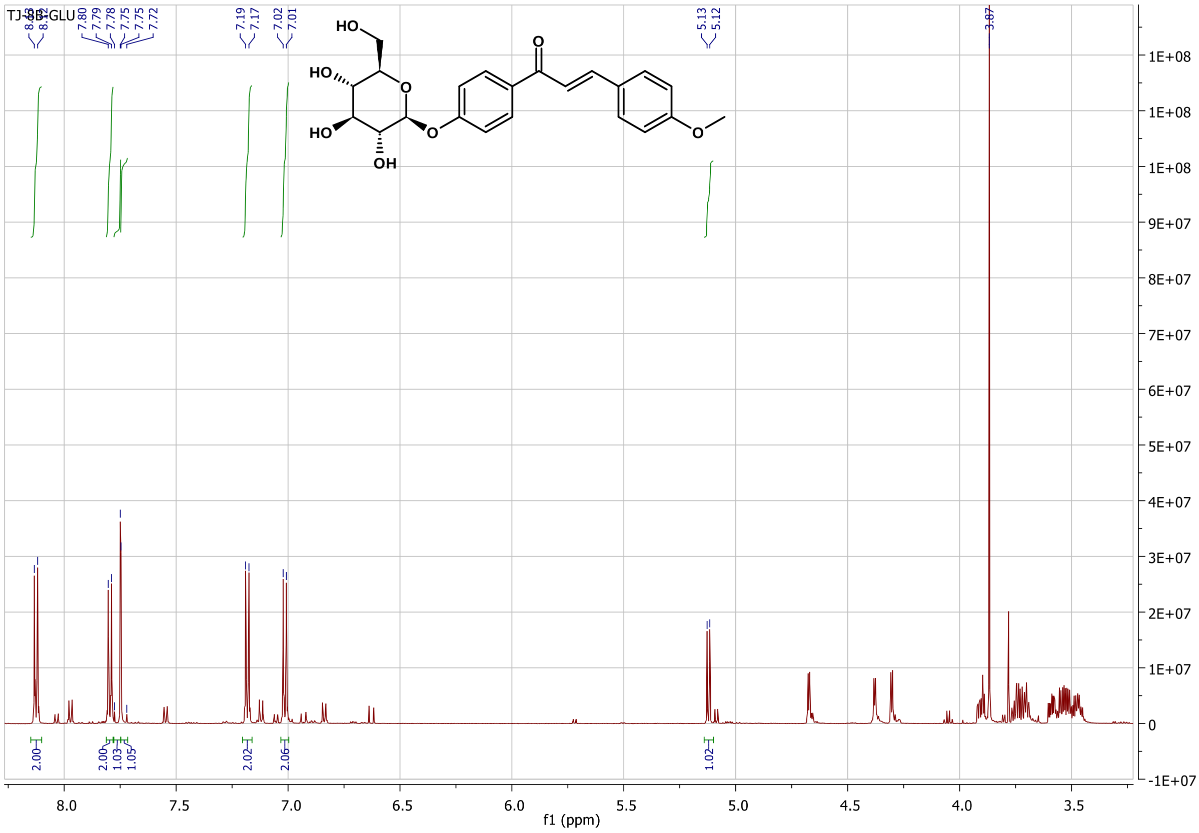


**Figure S60** $^{1}$H NMR spectrum of *trans*-4′-*O*-β-D-(glucopyranosyl)-4-methoxychalcone (600MHz; Acetone-d6)

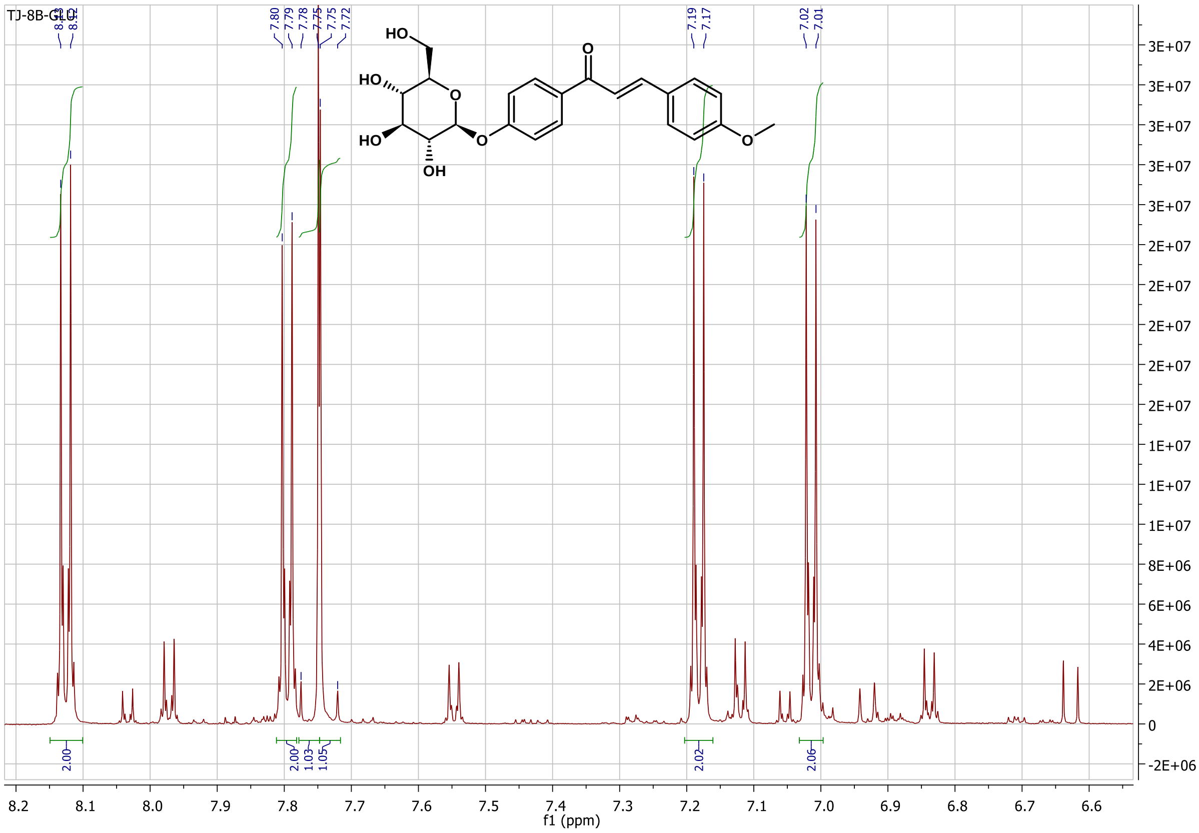

**Figure S61** Flavonoid part of ¹H NMR spectrum of *trans*-4′-*O*-β-D-(glucopyranosyl)-4-methoxychalcone (600MHz; Acetone-d₆)

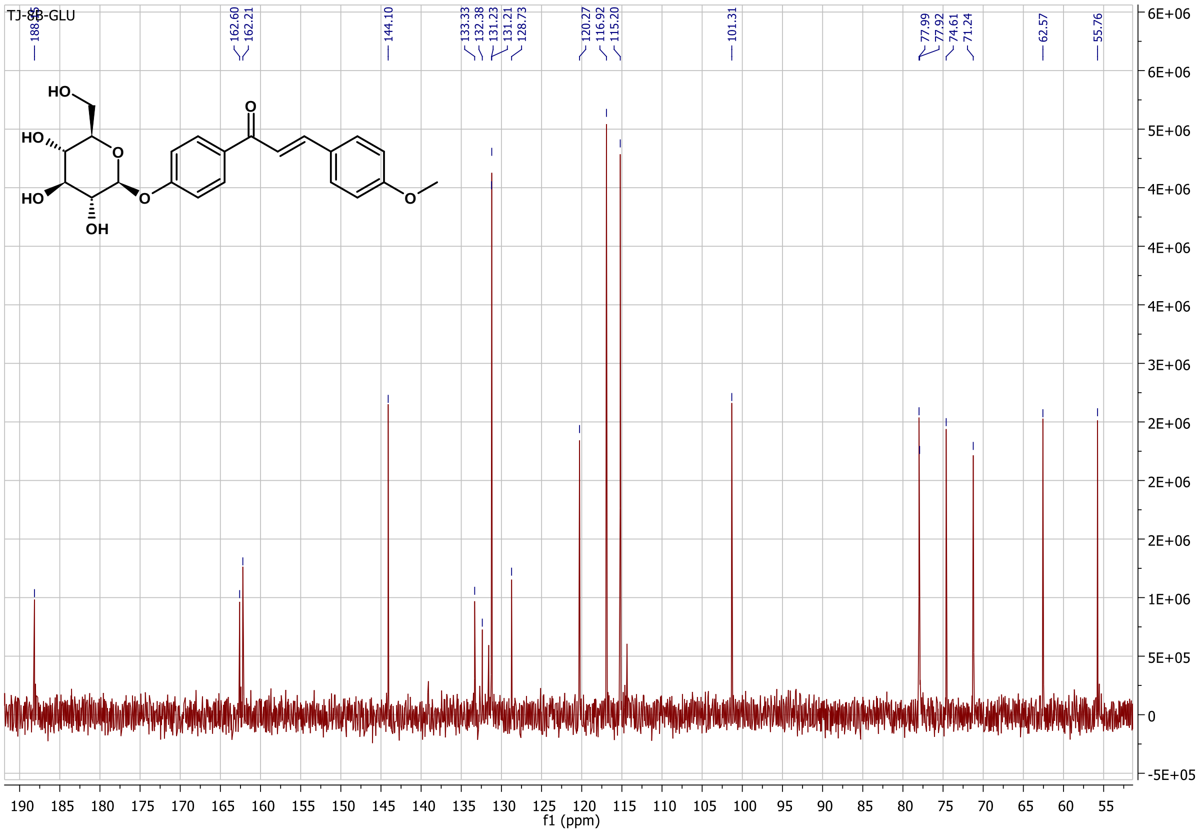

**Figure S62** ¹³C NMR spectrum of *trans*-4′-*O*-β-D-(glucopyranosyl)-4-methoxychalcone (600MHz; Acetone-d₆)

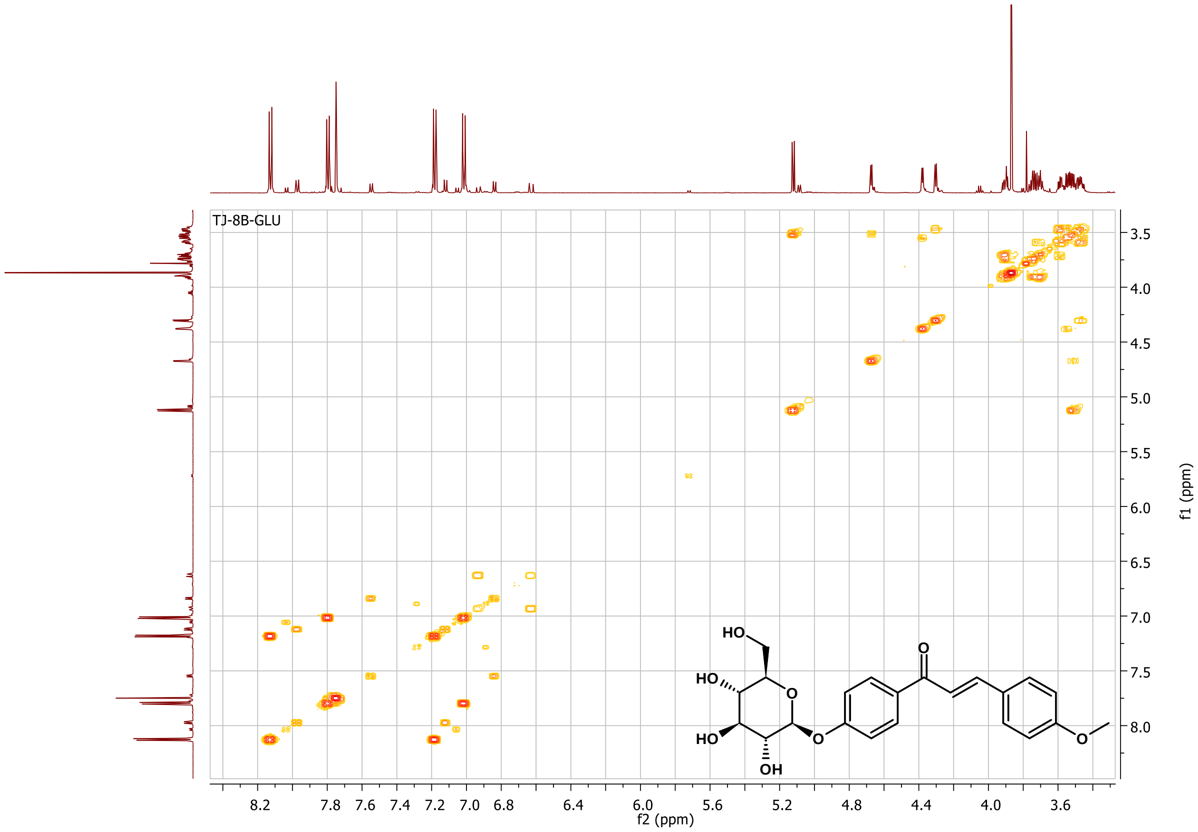


**Figure S63** COSY NMR spectrum of *trans*-4′-*O*-β-D-(glucopyranosyl)-4-methoxychalcone (600MHz; Acetone-d$_6$)

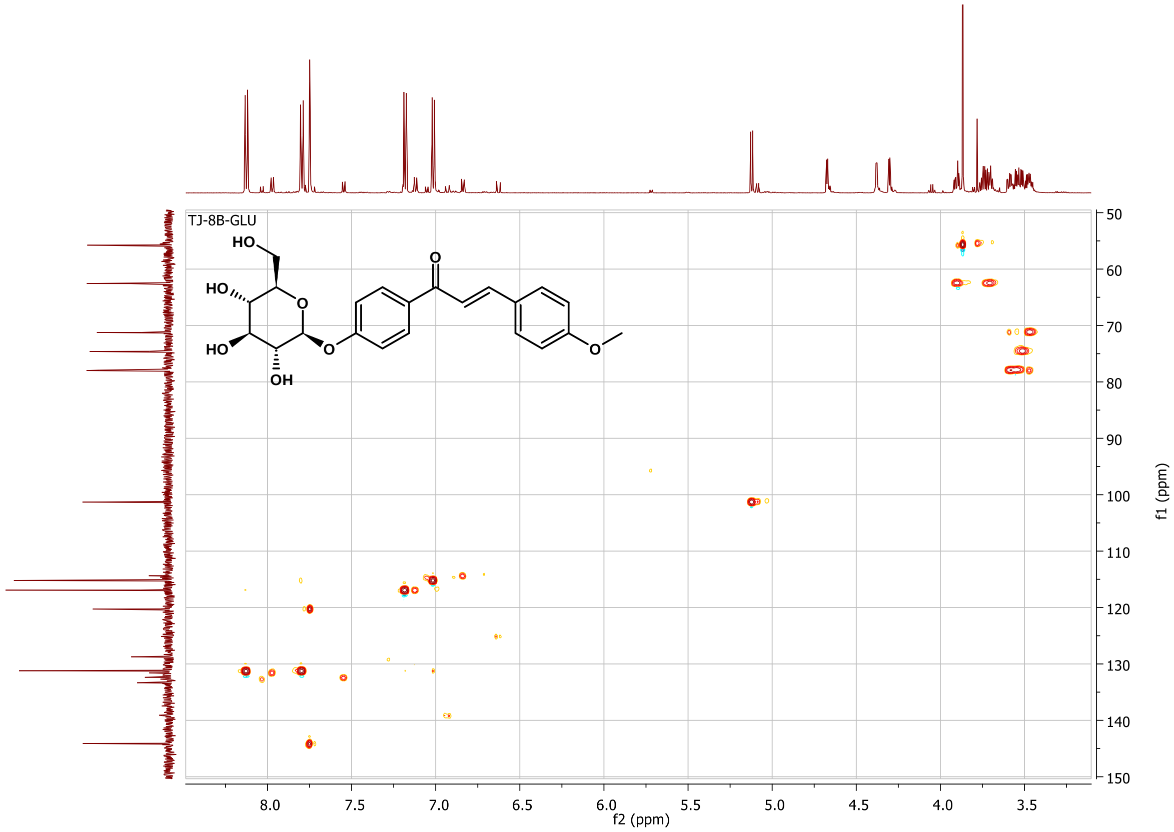


**Figure S64** HMQC NMR spectrum of *trans*-4′-*O*-β-D-(glucopyranosyl)-4-methoxychalcone (600MHz; Acetone-d$_6$)

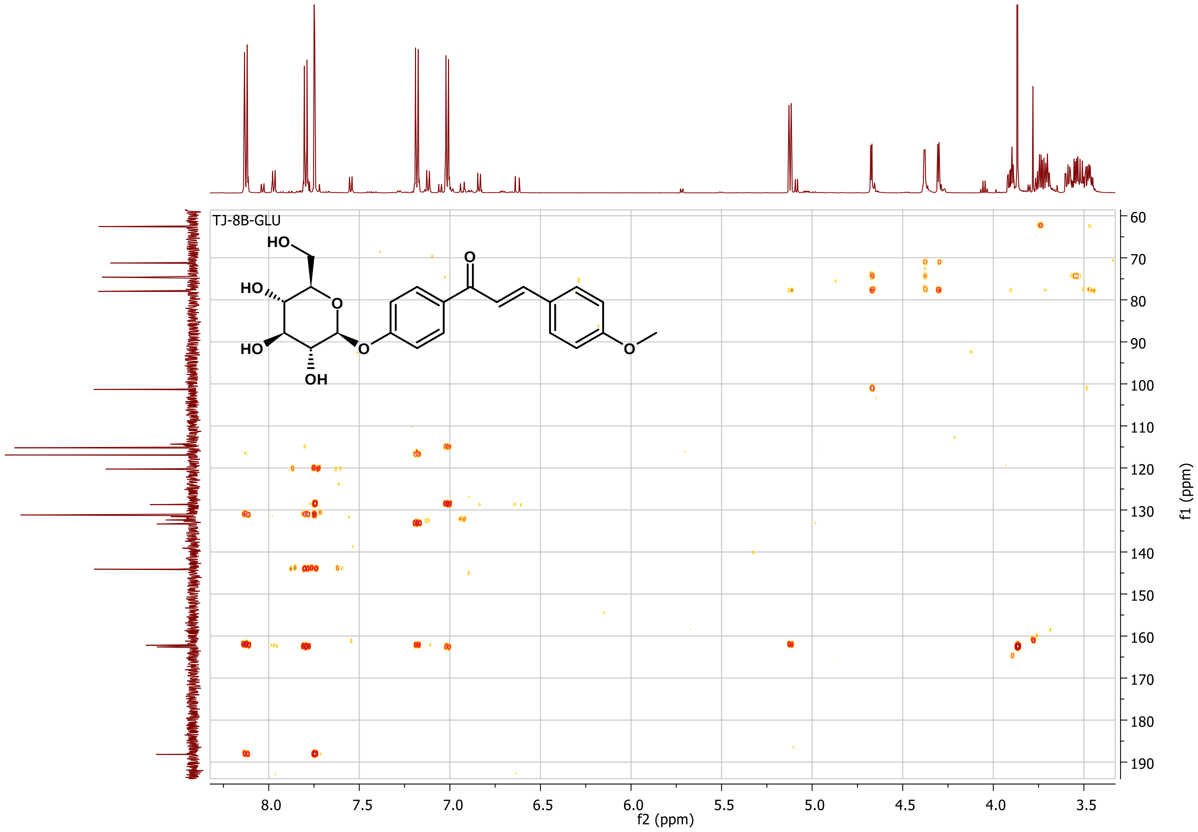

**Figure S65** HMBC NMR spectrum of *trans*-4′-*O*-β-D-(glucopyranosyl)-4-methoxychalcone (600MHz; Acetone-d6)

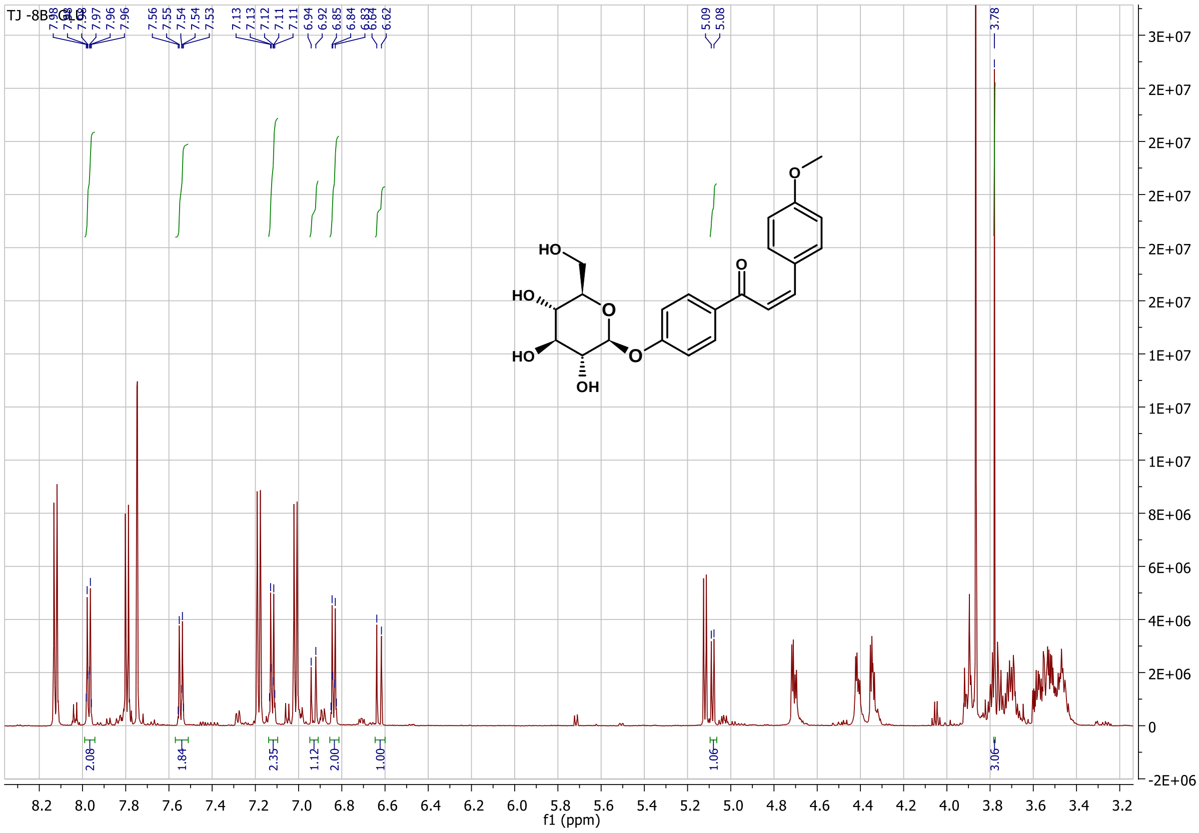

**Figure S66** $^{1}$H NMR spectrum of *cis*-4′-*O*-β-D-(glucopyranosyl)-4-methoxychalcone (600MHz; Acetone-d6)

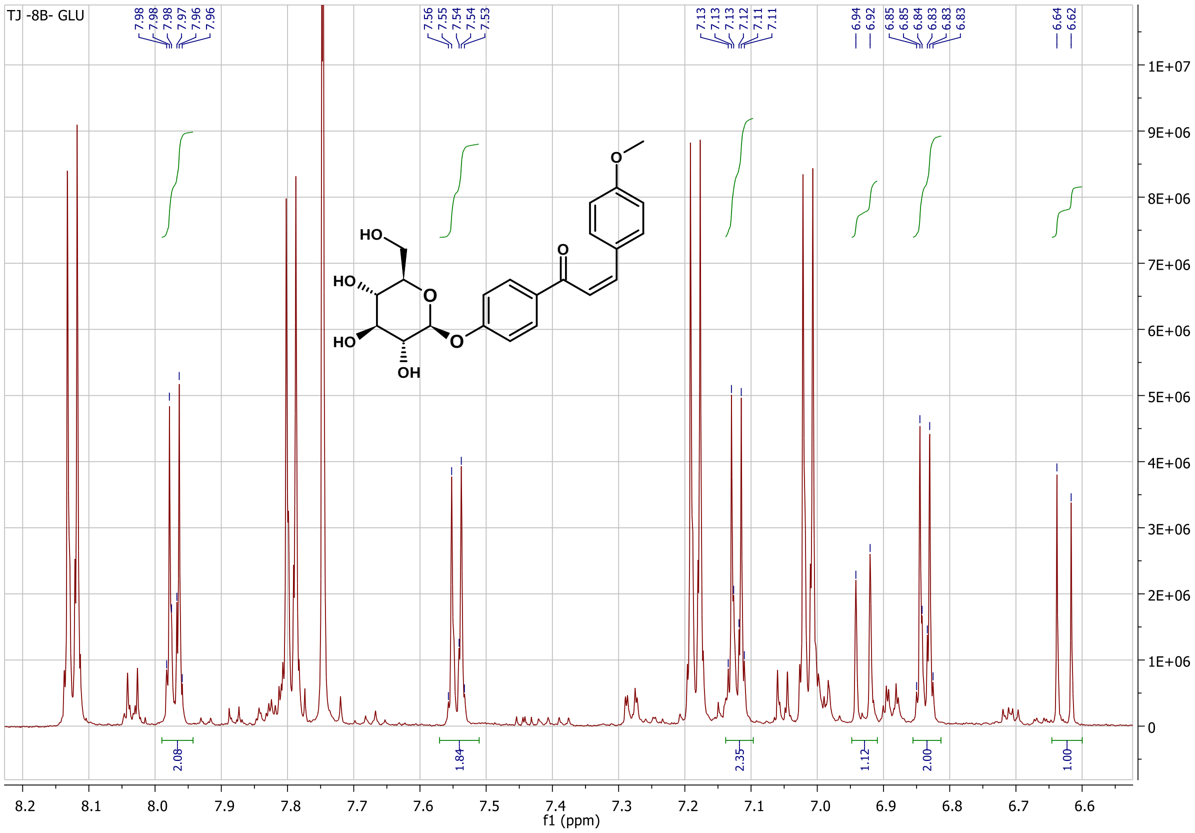

**Figure S67** Flafonoid part of $^1$H NMR spectrum of *cis*-4′-*O*-β-D-(glucopyranosyl)-4-methoxychalcone (600MHz; Acetone-d$_6$)

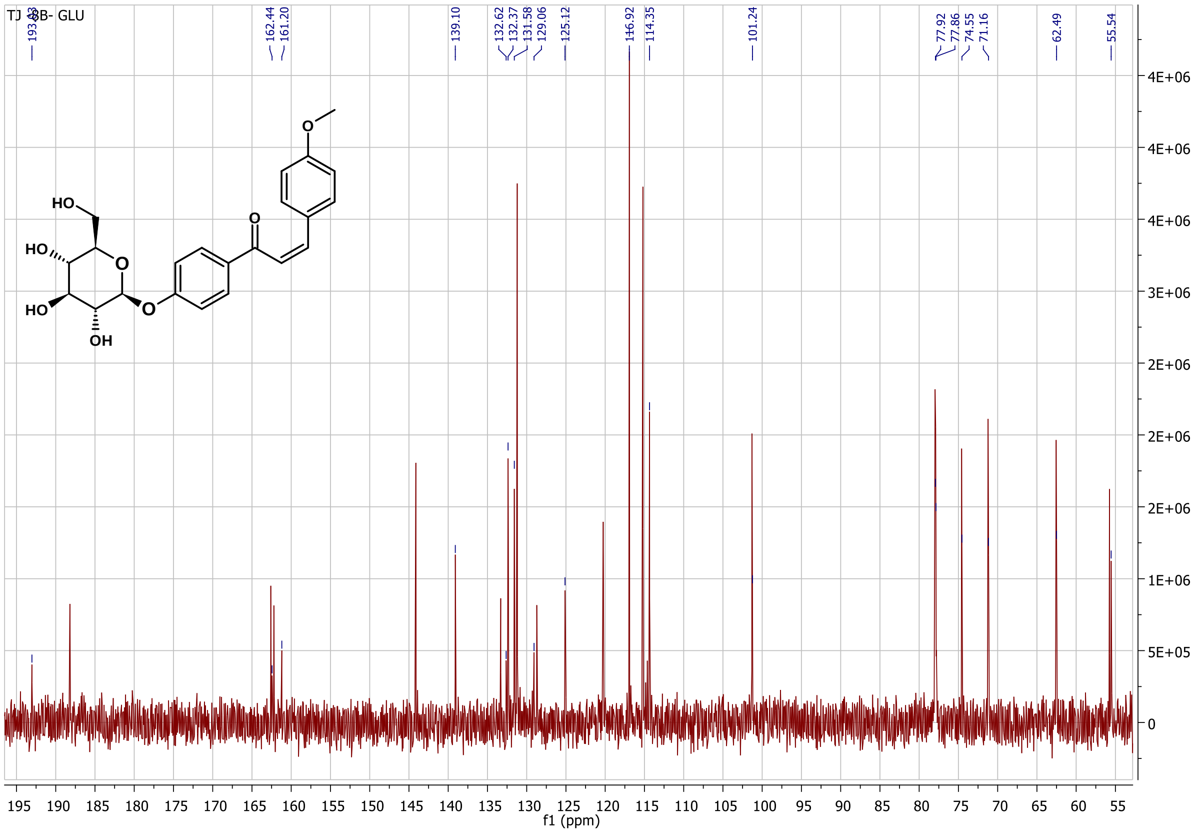

**Figure S68** $^{13}$C NMR spectrum of *cis*-4′-*O*-β-D-(glucopyranosyl)-4-methoxychalcone (151MHz; Acetone-d$_6$)

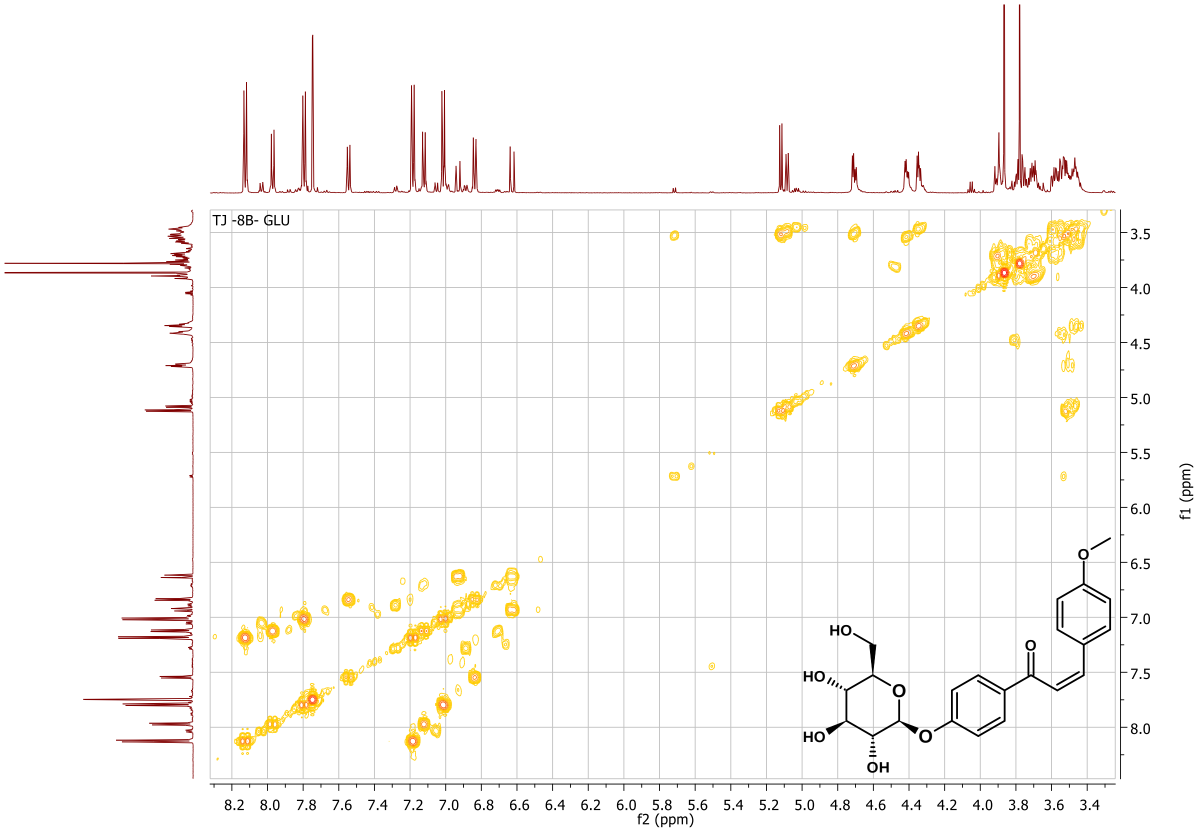


**Figure S69** COSY NMR spectrum of *cis*-4′-*O*-β-D-(glucopyranosyl)-4-methoxychalcone (600MHz; Acetone-d6)

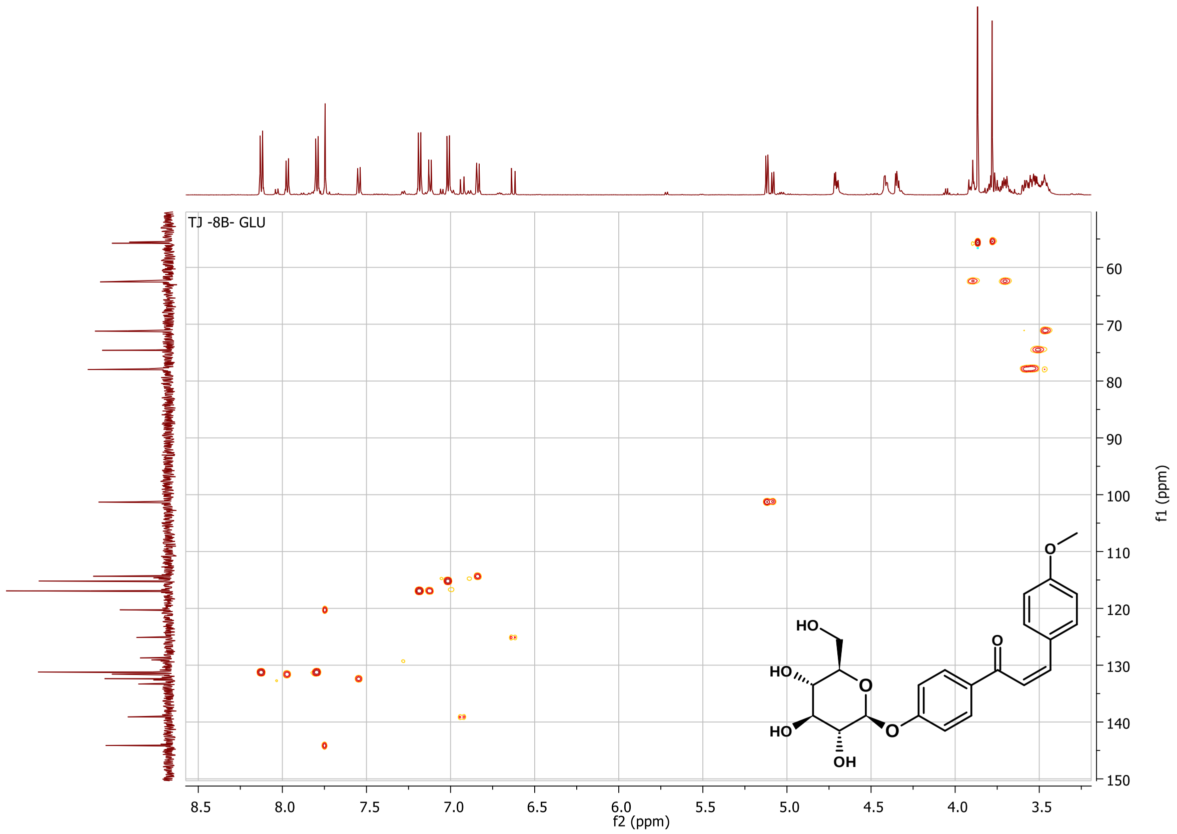


**Figure S70** HMQC NMR spectrum of *cis*-4′-*O*-β-D-(glucopyranosyl)-4-methoxychalcone (600MHz; Acetone-d6)

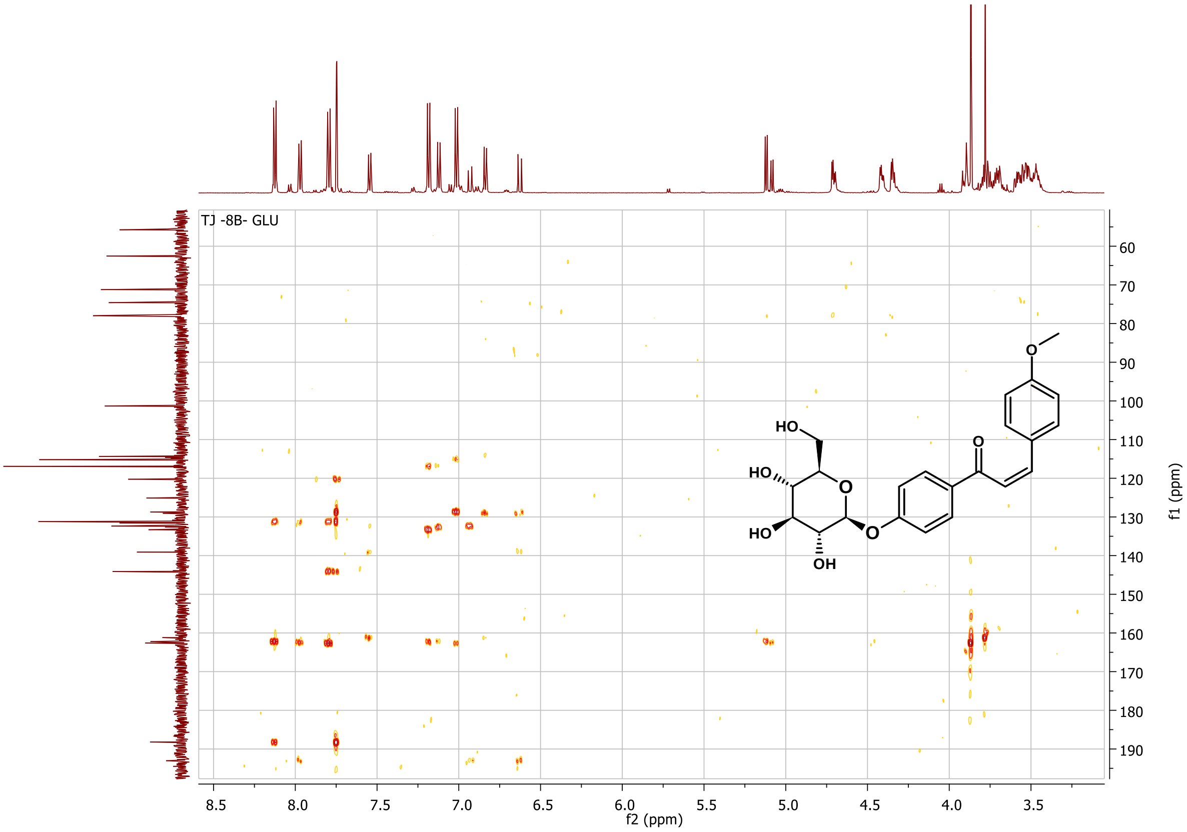


**Figure S71** HMBC NMR spectrum of *cis*-4′-*O*-β-D-(glucopyranosyl)-4-methoxychalcone (600MHz; Acetone-d6)

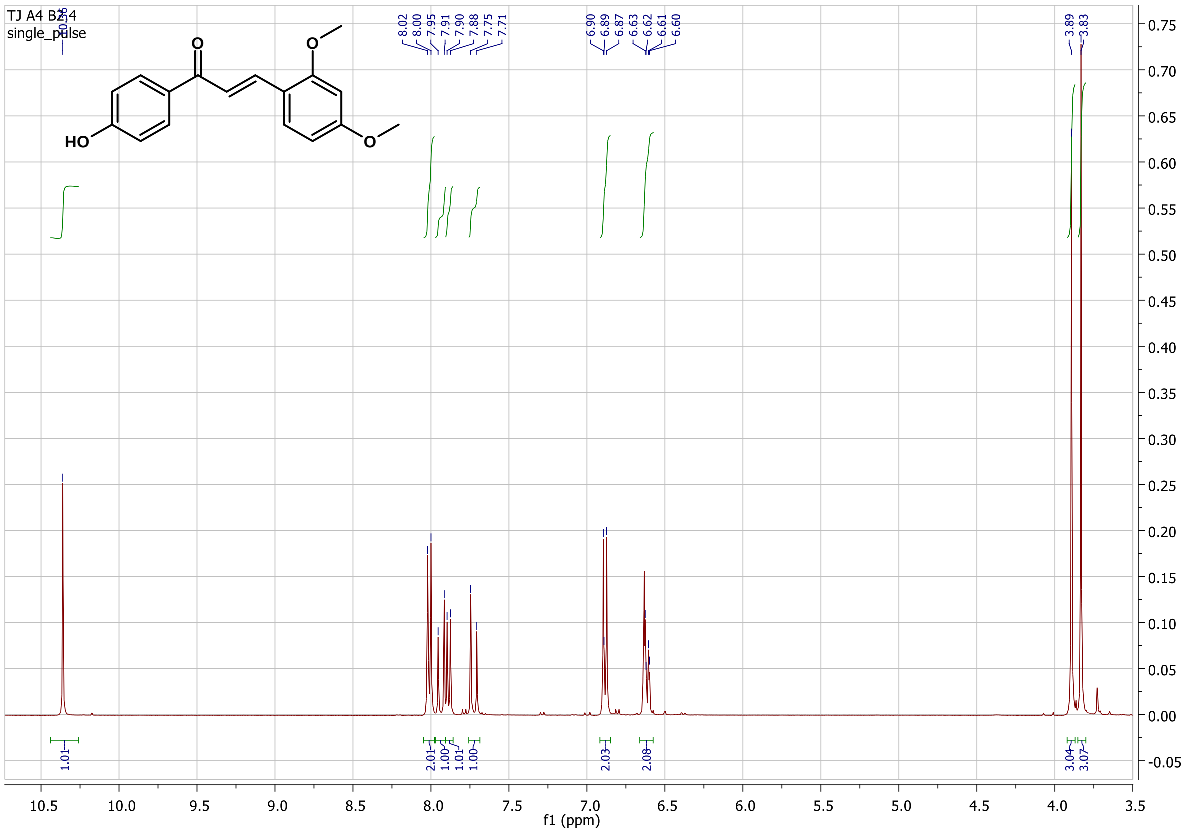


**Figure S72** $^{1}$H NMR spectrum of *trans*-4′-hydroxy-2,4-dimethoxychalcone (600MHz; DMSO-d6)

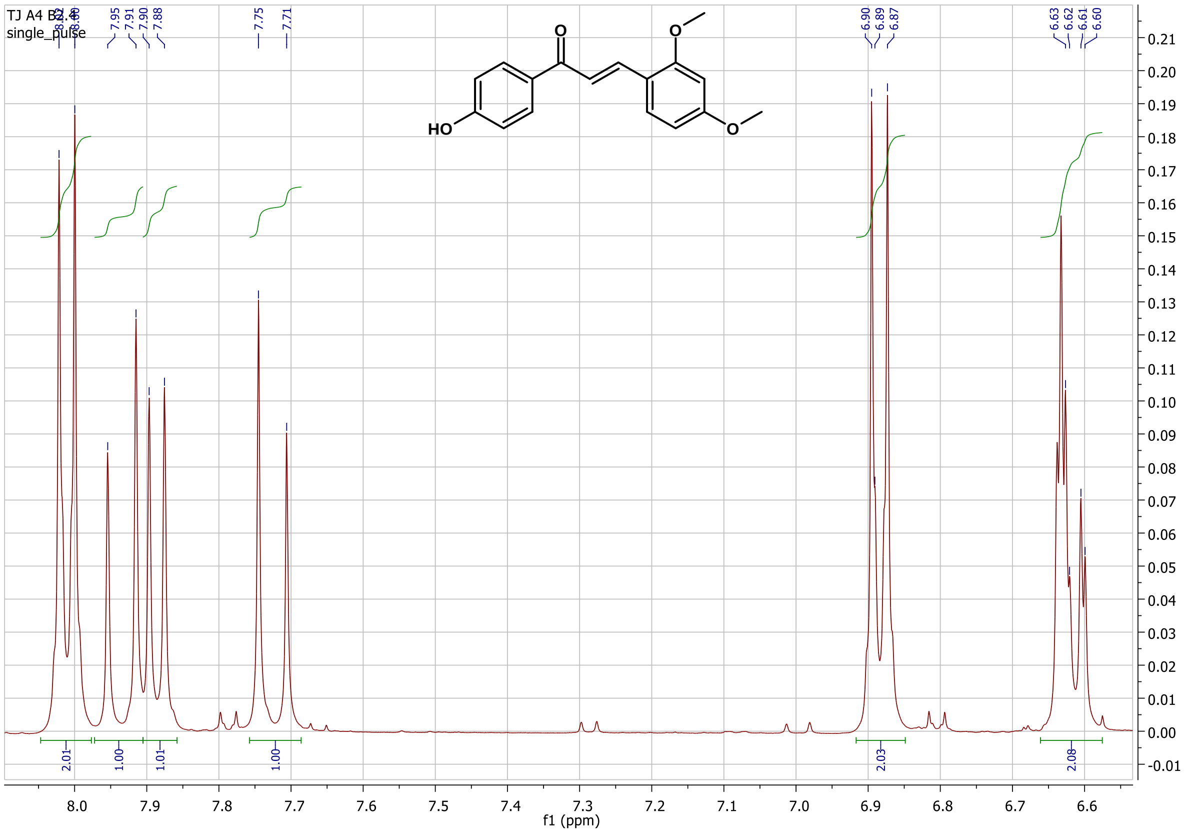


**Figure S73** Flavonoid part of spectrum of *trans*-4′-hydroxy-2,4-dimethoxychalcone (600MHz; DMSO-$d_6$)

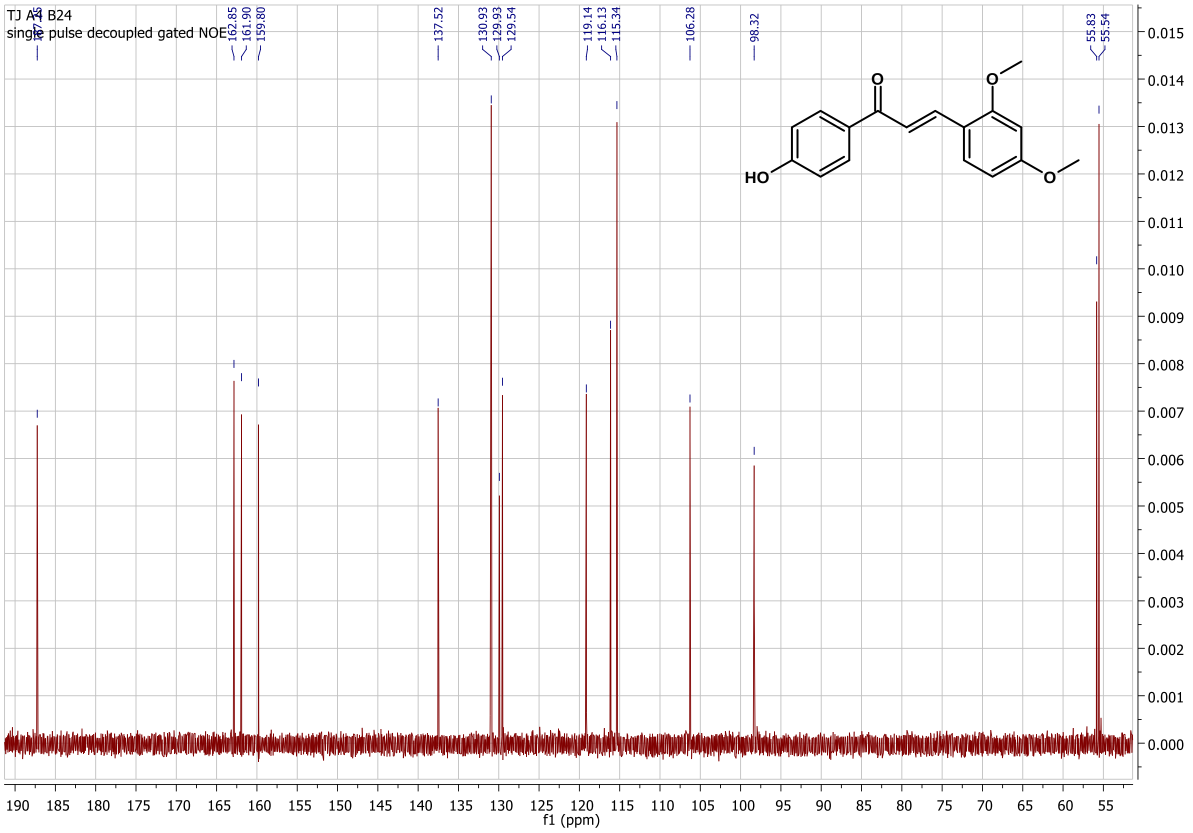


**Figure S74** $^{13}C$ NMR spectrum of *trans*-4′-hydroxy-2,4-dimethoxychalcone (151MHz; DMSO-$d_6$)

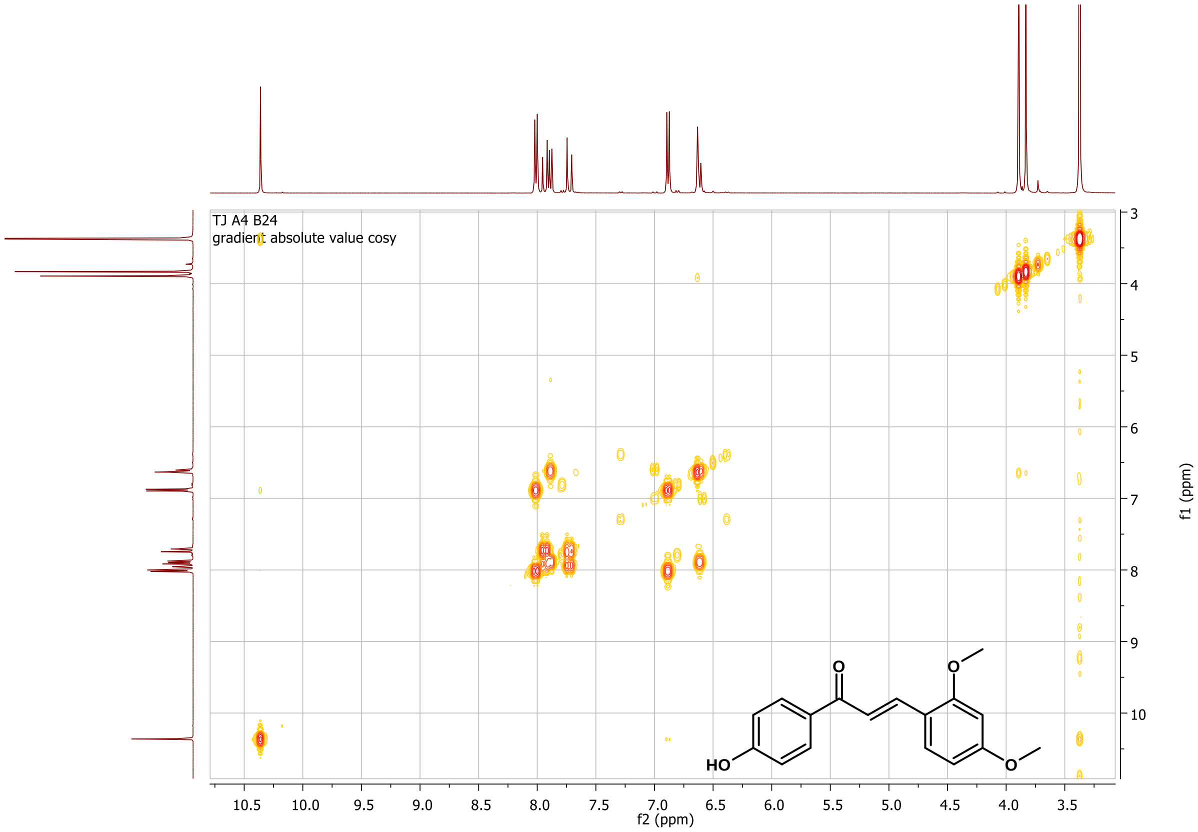


**Figure S75** COSY NMR spectrum of *trans*-4′-hydroxy-2,4-dimethoxychalcone (600MHz; DMS*O*-d6)

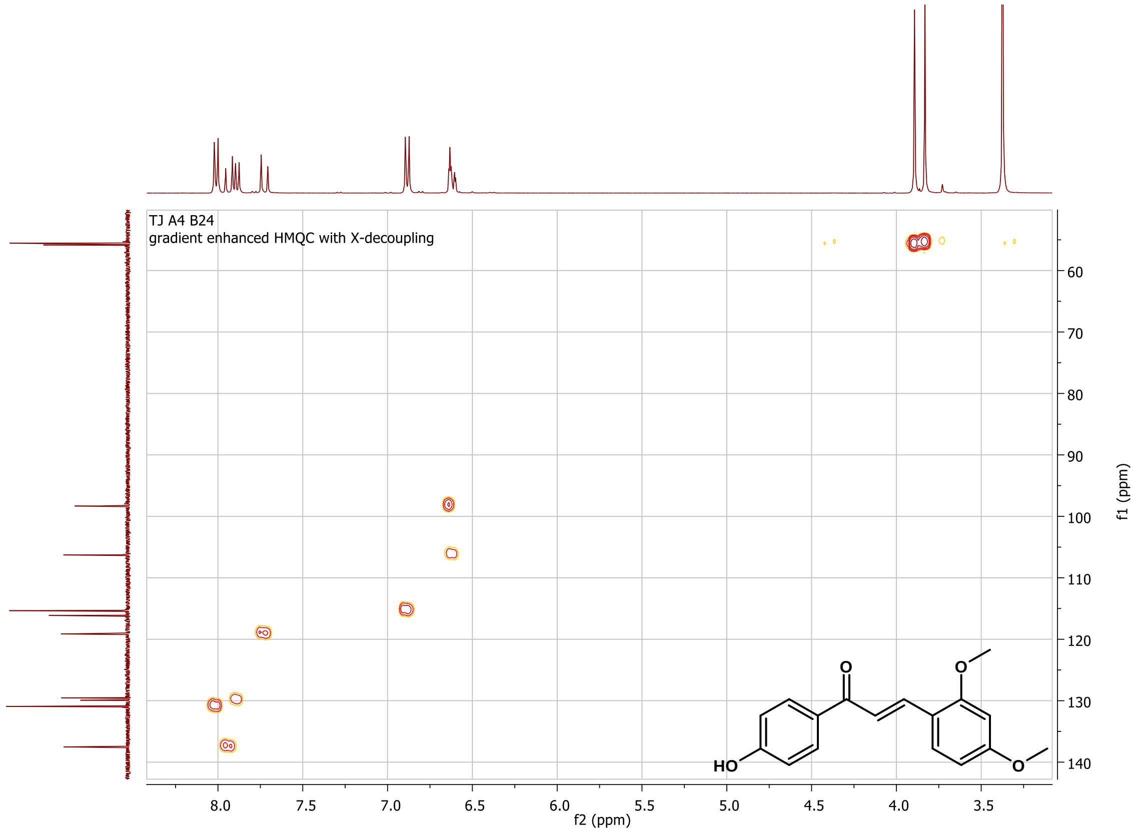


**Figure S76** HMQC NMR spectrum of *trans*-4′-hydroxy-2,4-dimethoxychalcone (600MHz; DMS*O*-d6)

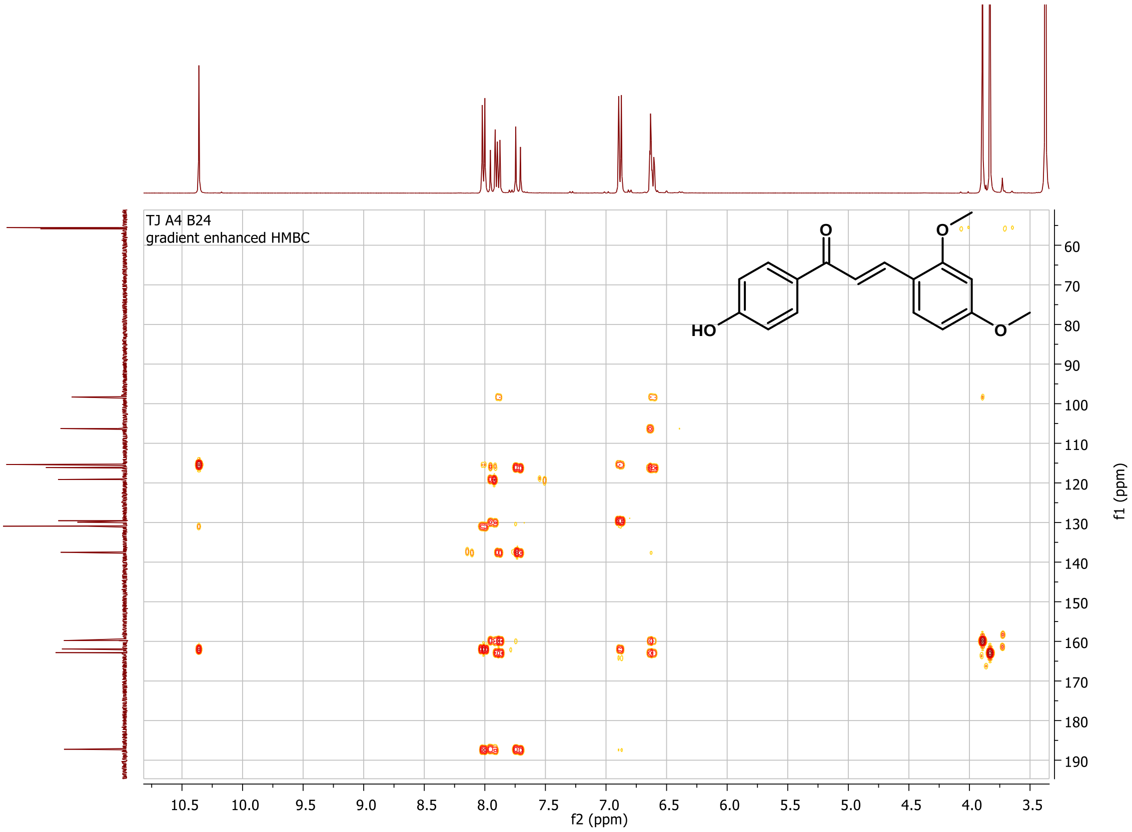


**Figure S77** HMBC NMR spectrum of *trans*-4′-hydroxy-2,4-dimethoxychalcone (600MHz; DMSO-d6)

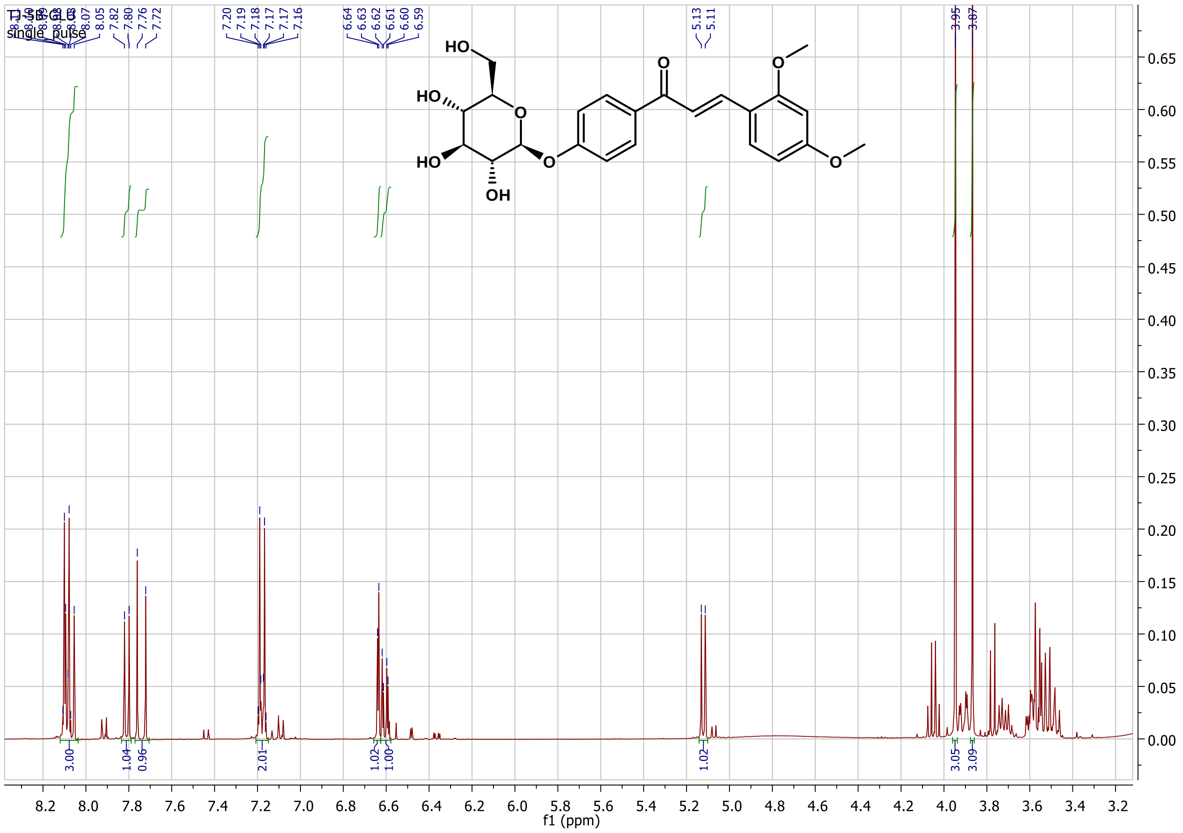


**Figure S78** $^{1}$H NMR spectrum of *trans*-4′-*O*-β-D-(glucopyranosyl)-2,4-dimethoxychalcone (600MHz, Acetone-d6)

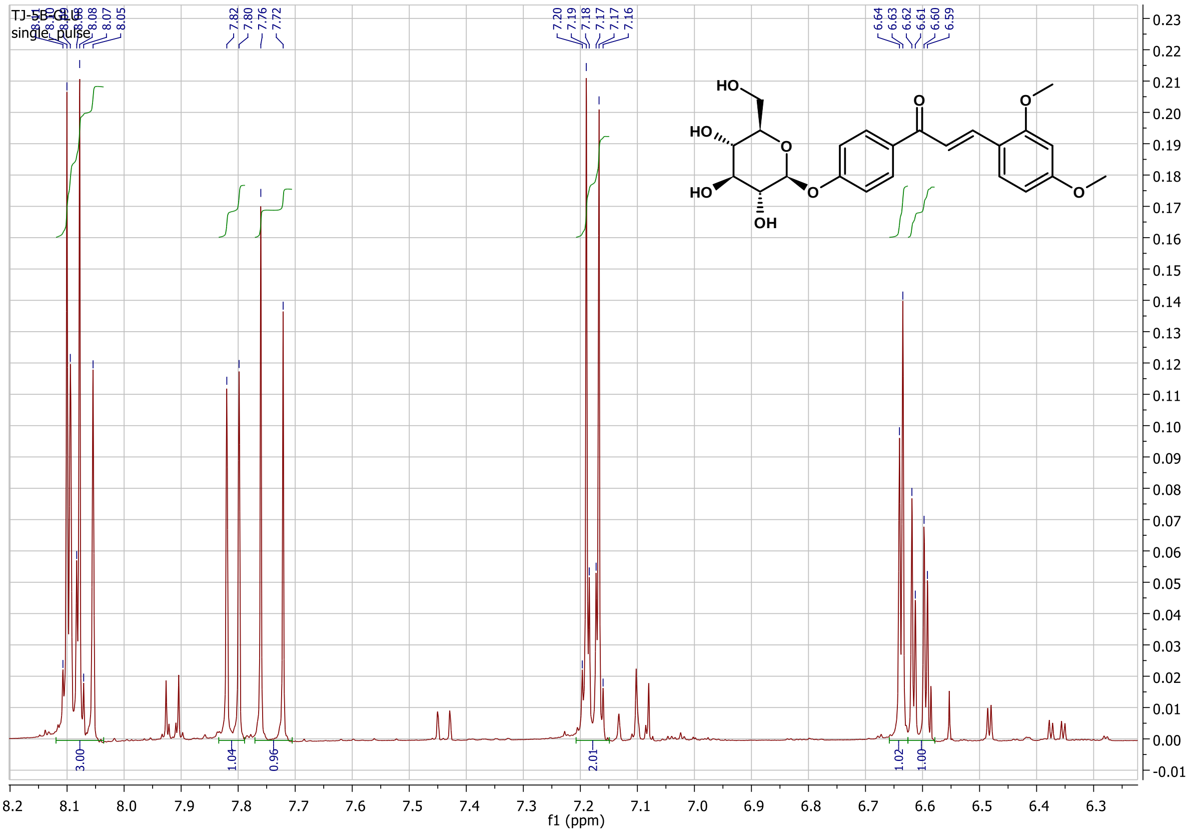

**Figure S79** Flavonoid fragment of $^{1}$H NMR spectrum of *trans*-4′-*O*-β-D-(glucopyranosyl)-2,4-dimethoxychalcone (600MHz, Acetone-d$_6$)

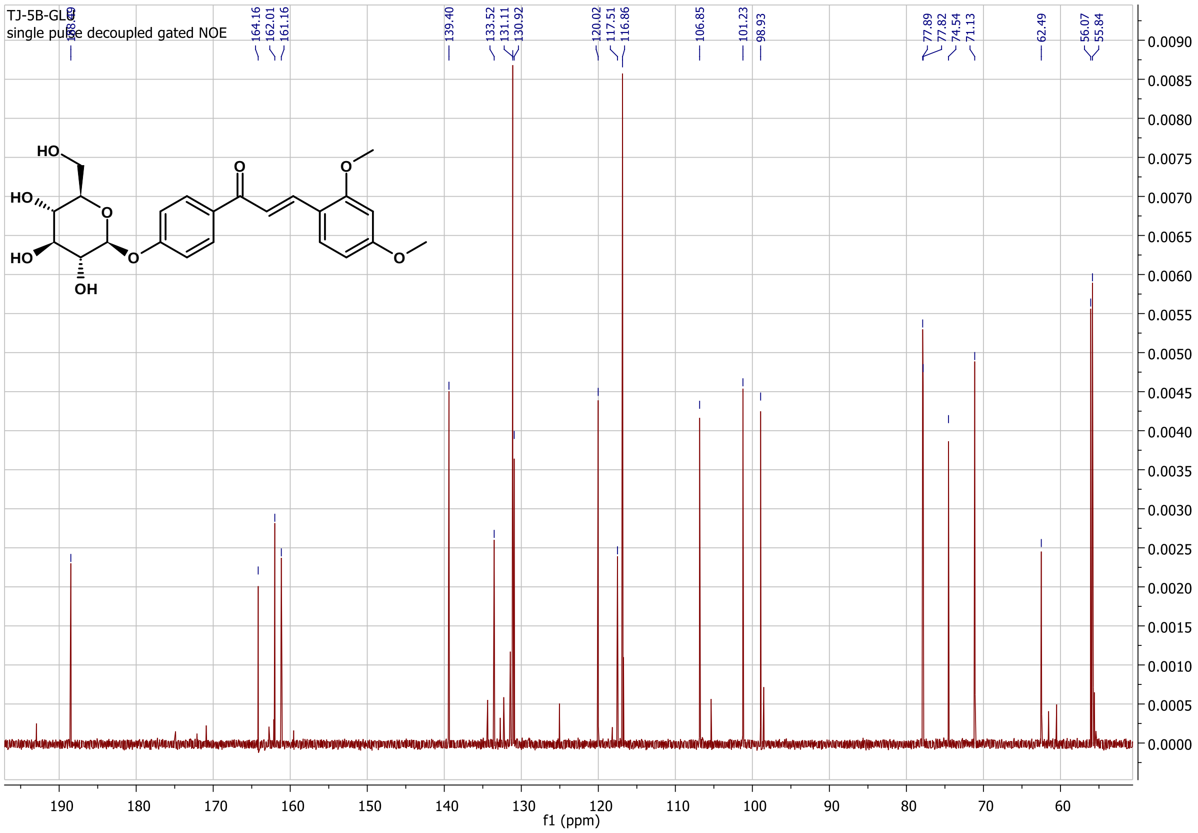

**Figure S80** $^{13}$C NMR spectrum of *trans*-4′-*O*-β-D-(glucopyranosyl)-2,4-dimethoxychalcone (151MHz, Acetone-d$_6$)

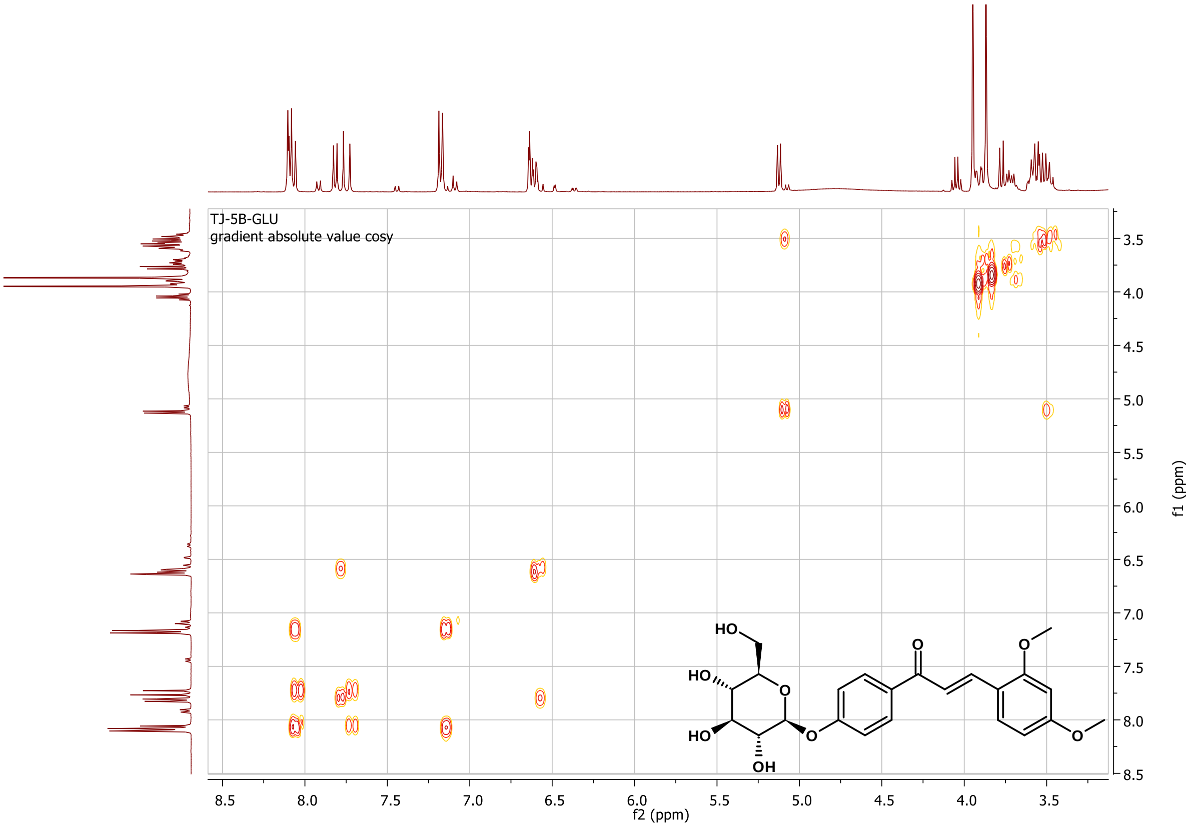


**Figure S81** COSY NMR spectrum of *trans*-4′-*O*-β-D-(glucopyranosyl)-2,4-dimethoxychalcone (600MHz, Acetone-d6)

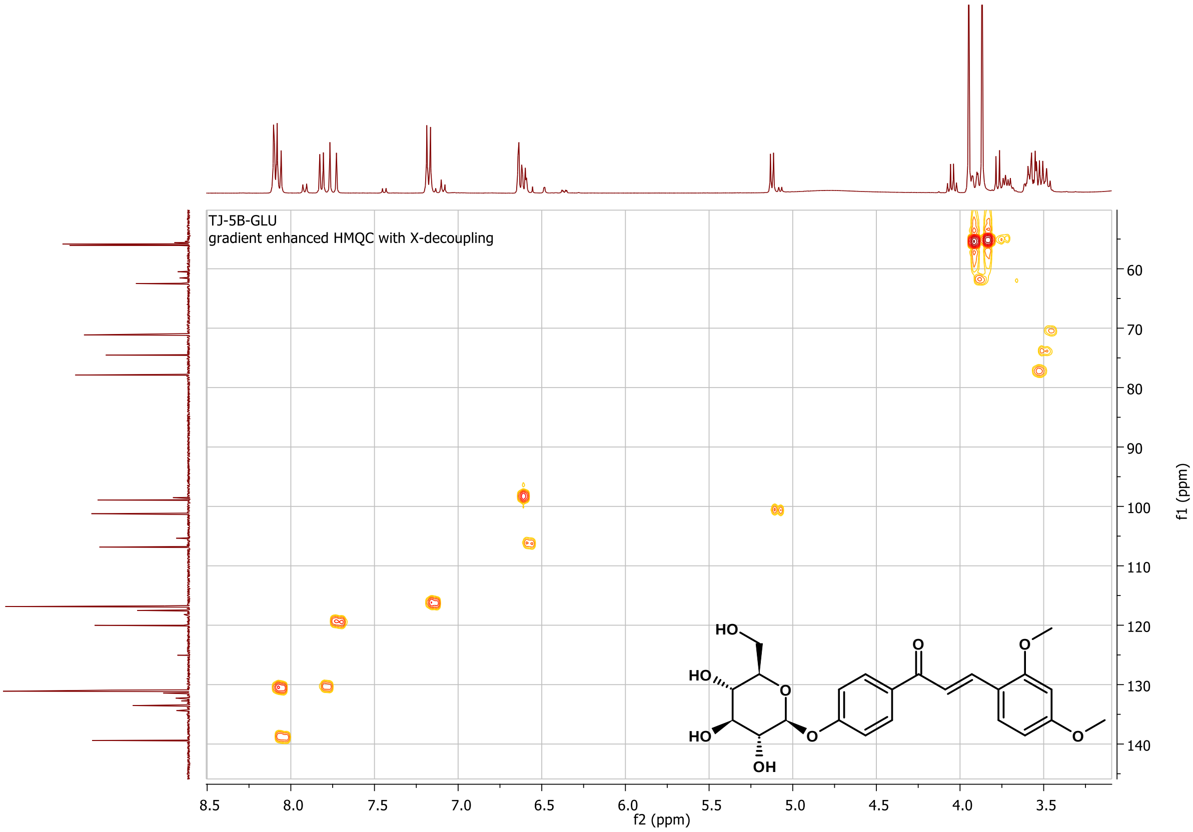


**Figure S82** HMQC NMR spectrum of *trans*-4′-*O*-β-D-(glucopyranosyl)-2,4-dimethoxychalcone (600MHz, Acetone-d6)

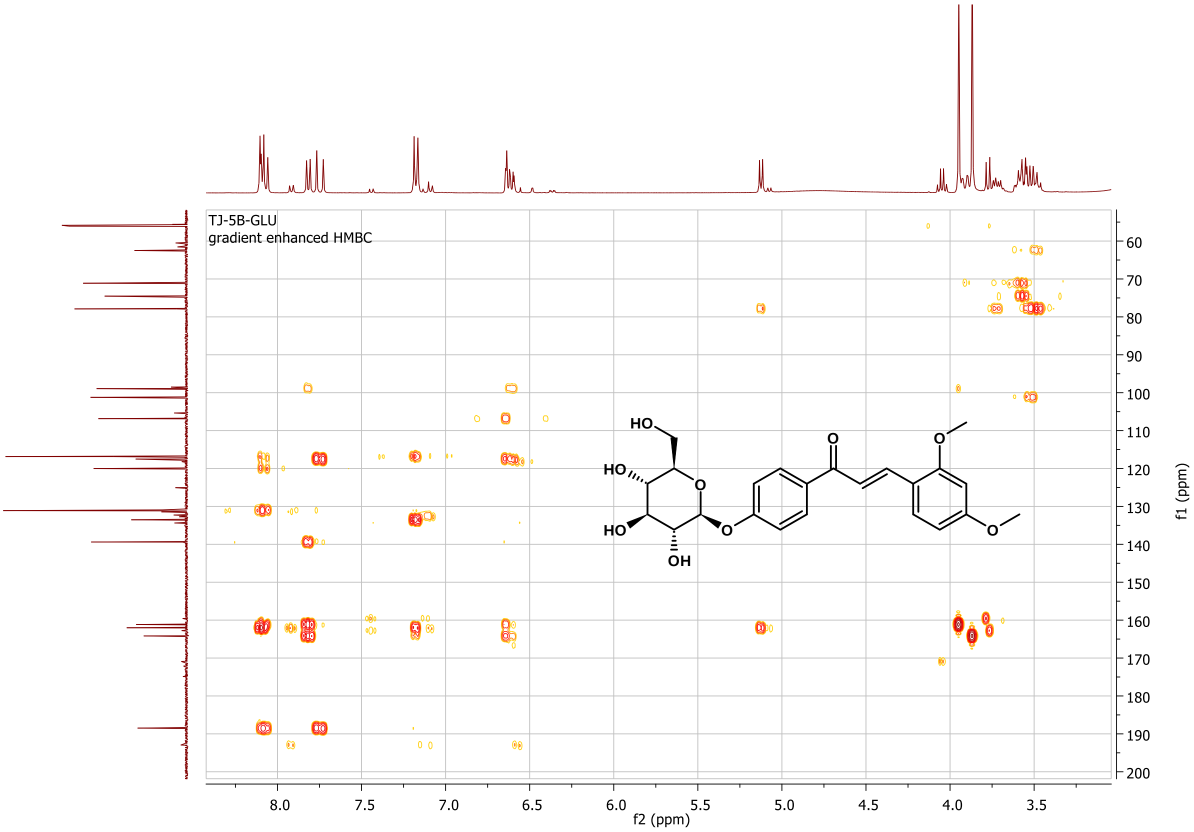


**Figure S83** HMBC NMR spectrum of *trans*-4′-*O*-β-D-(glucopyranosyl)-2,4-dimethoxychalcone (600MHz, Acetone-$d_6$)

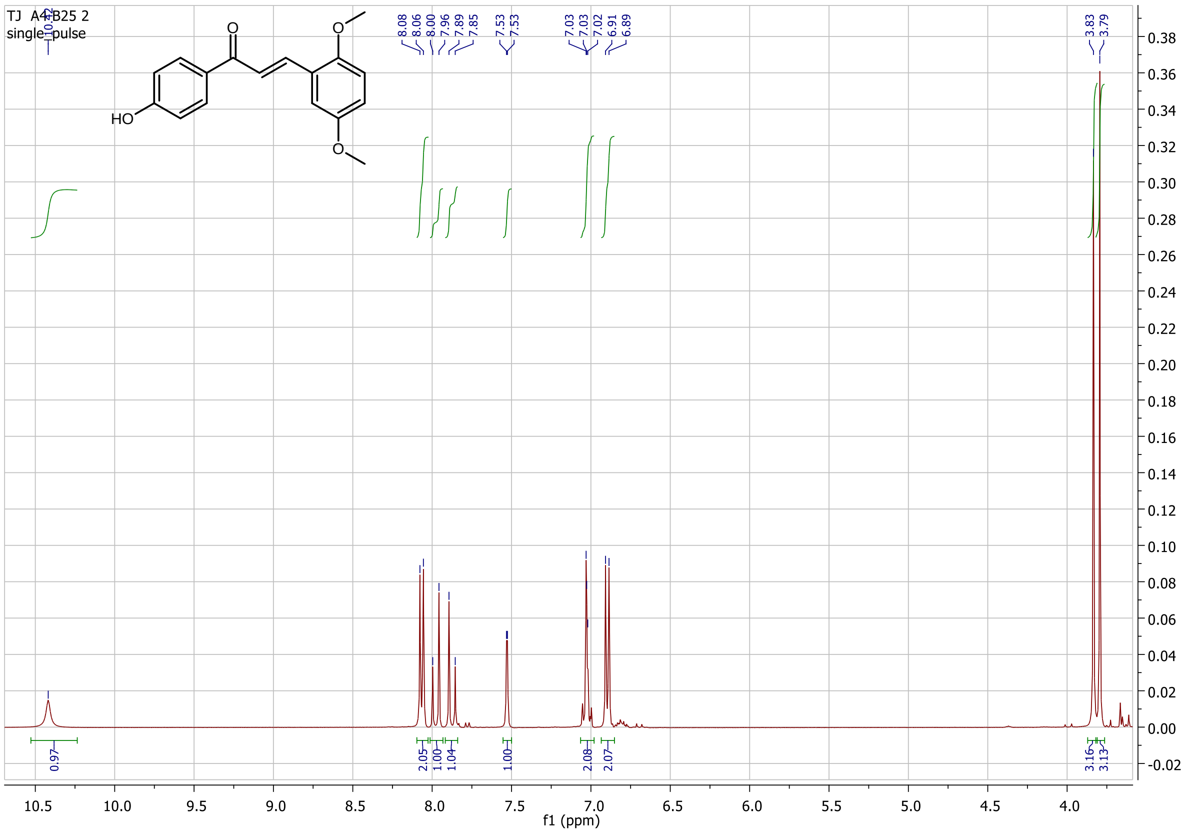


**Figure S84** $^1$H NMR spectrum of *trans*-4′-hydroxy-2,5-dimethoxychalcone (600MHz; DMS*O*-$d_6$)

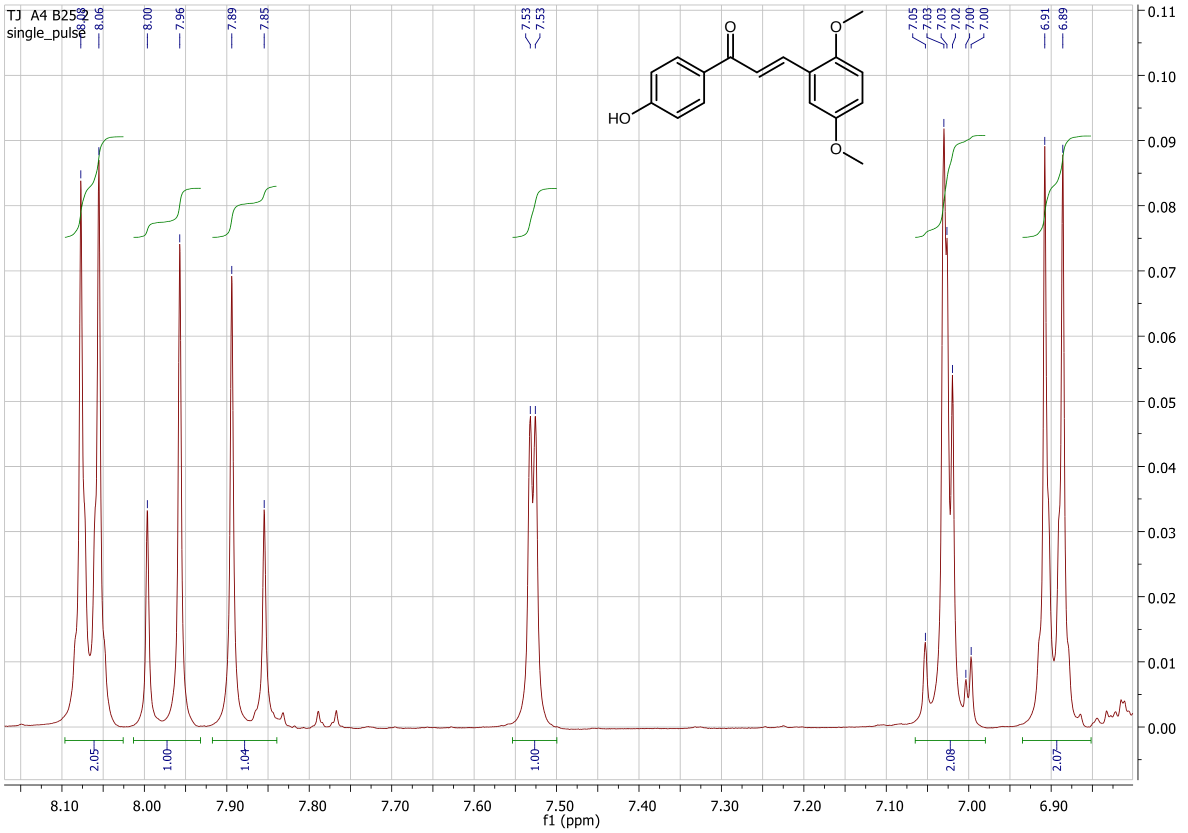


**Figure S85** Flavonoid part of $^{1}$H NMR spectrum of *trans*-4′-hydroxy-2,5-dimethoxychalcone (600MHz; DMS*O*-d₆)

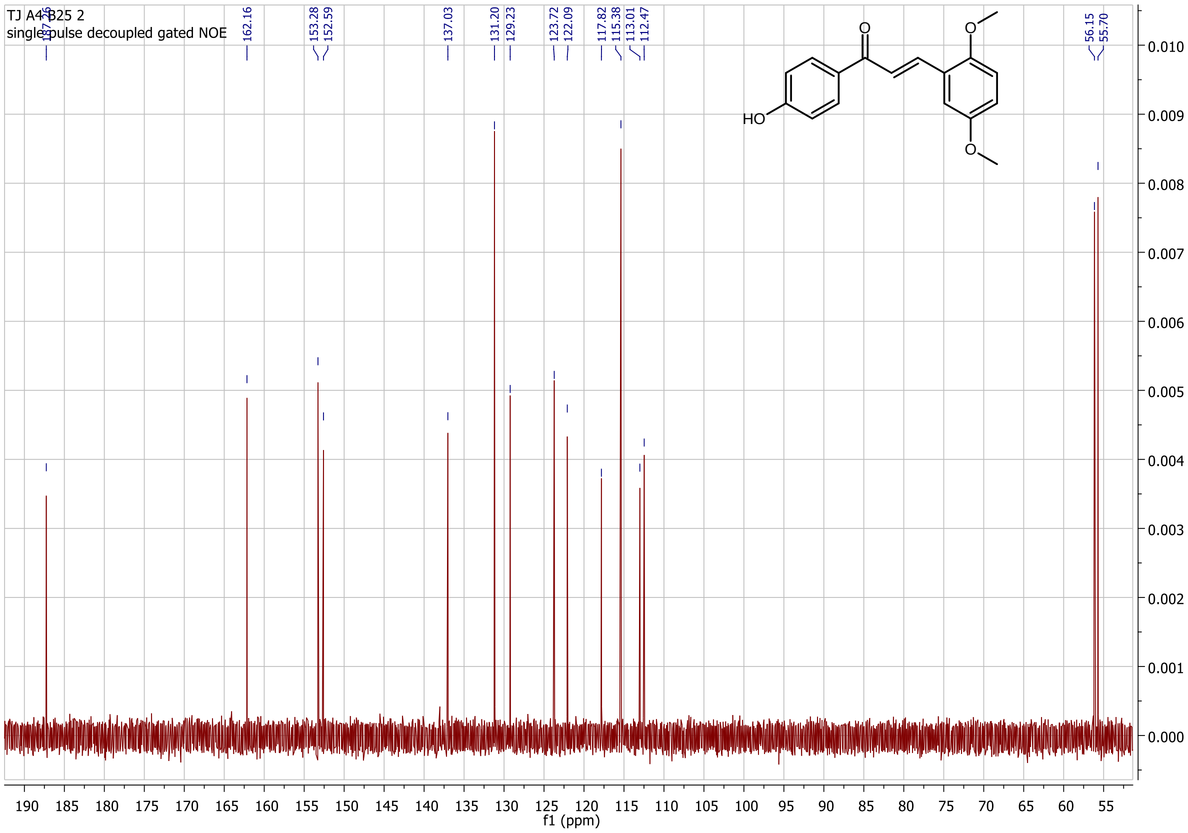


**Figure S86** $^{13}$C NMR spectrum of 4′-hydroxy-2,5-dimethoxychalcone (151MHz; DMS*O*-d₆)

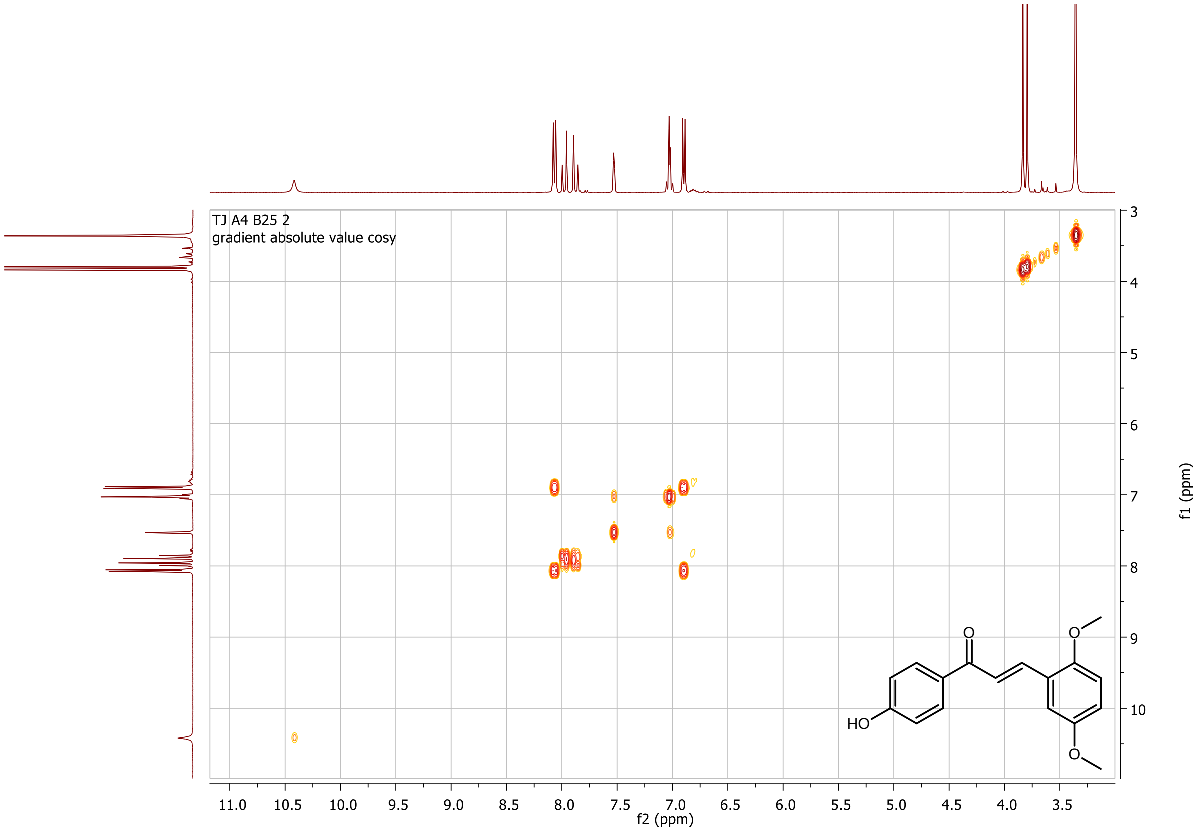


**Figure S87** COSY NMR spectrum of *trans*-4′-hydroxy-2,5-dimethoxychalcone (600MHz; DMSO-d$_6$)

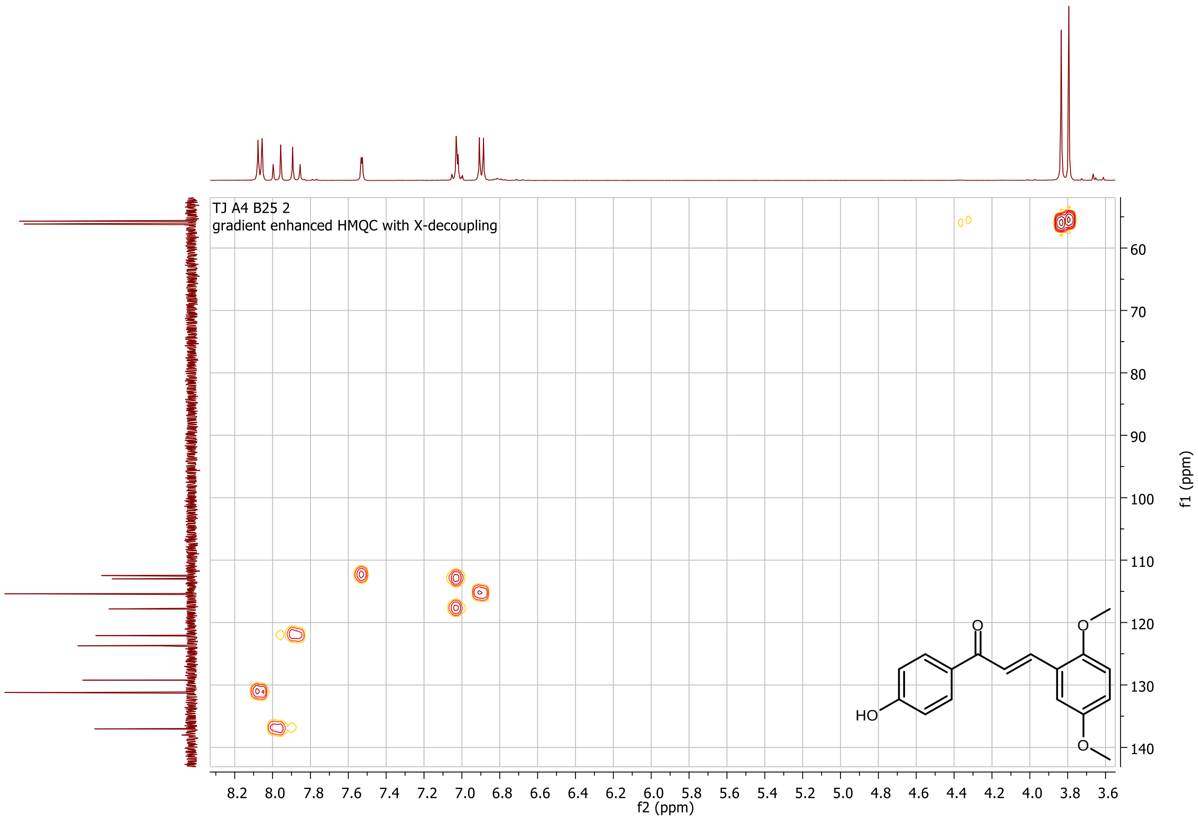


**Figure S88** HMQC NMR spectrum of *trans*-4′-hydroxy-2,5-dimethoxychalcone (600MHz; DMSO-d$_6$)

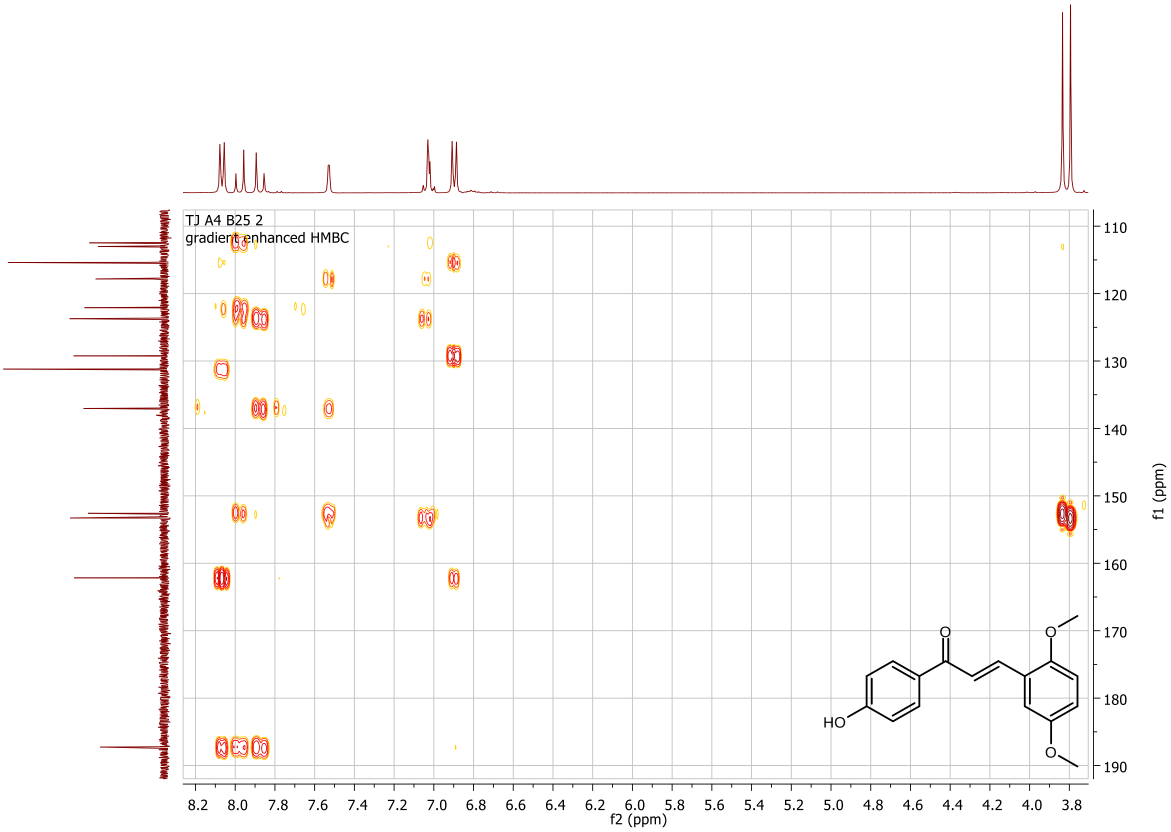

**Figure S89** HMBC NMR spectrum of *trans*-4′-hydroxy-2,5-dimethoxychalcone (600MHz; DMSO-d6)

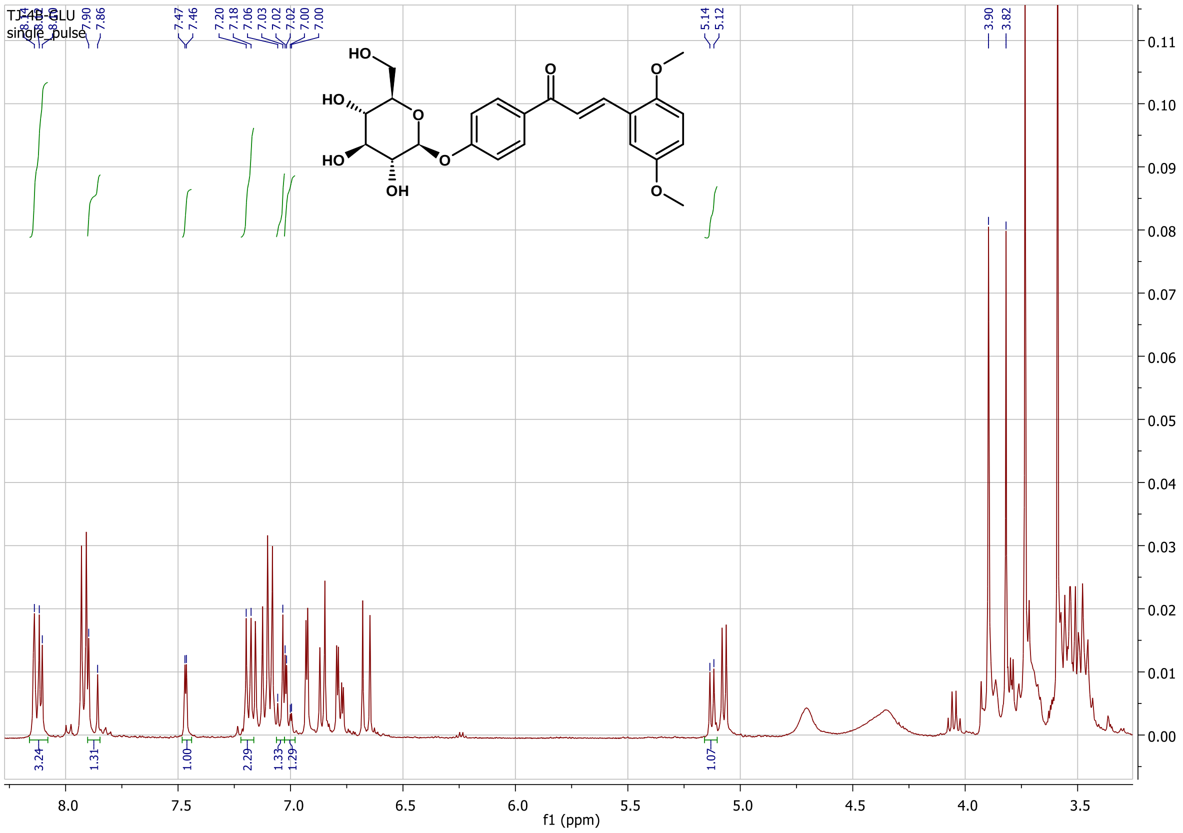

**Figure S90** ¹H NMR spectrum of *cis*-4′-*O*-β-D-(glucopyranosyl)-2,5-dimethoxychalcone (600MHz; Acetone-d6)

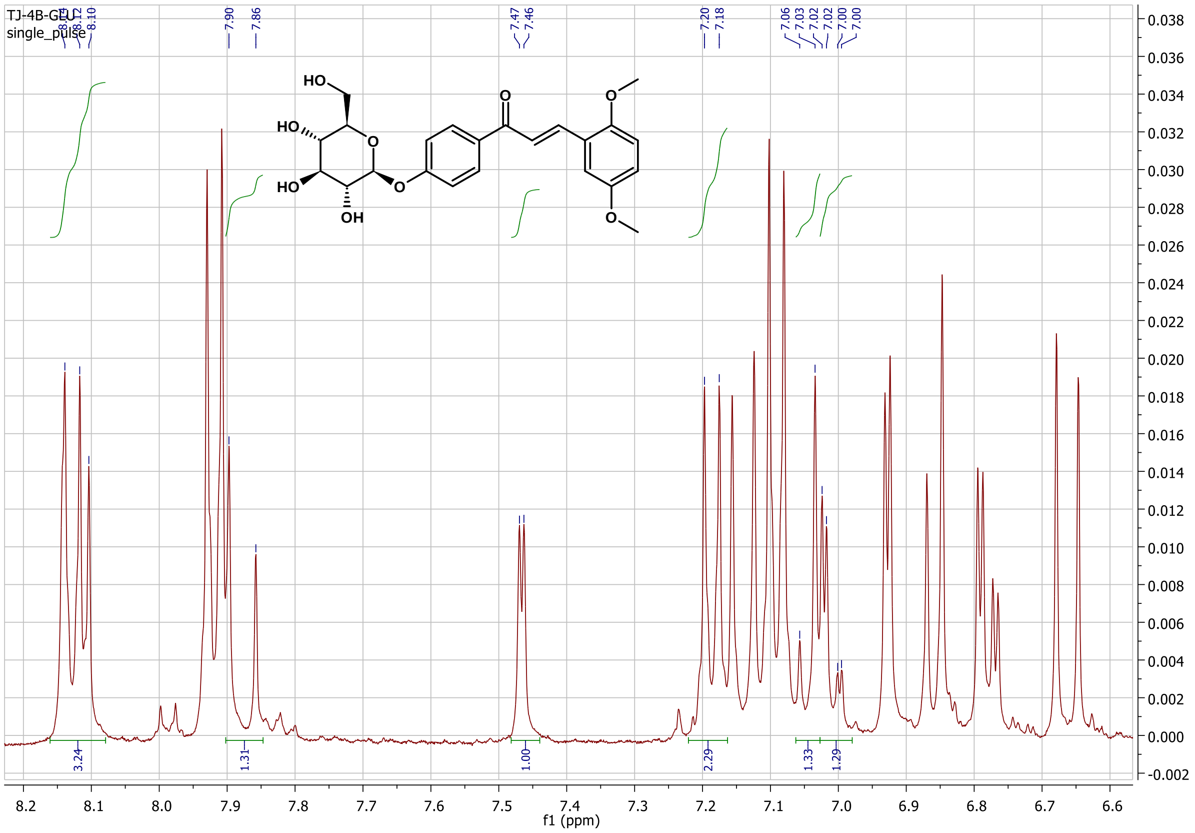


**Figure S91** Flavonoid fragment of ¹H NMR spectrum of *cis*-4′-*O*-β-D-(glucopyranosyl)-2,5-dimethoxychalcone (600MHz; Acetone-d₆)

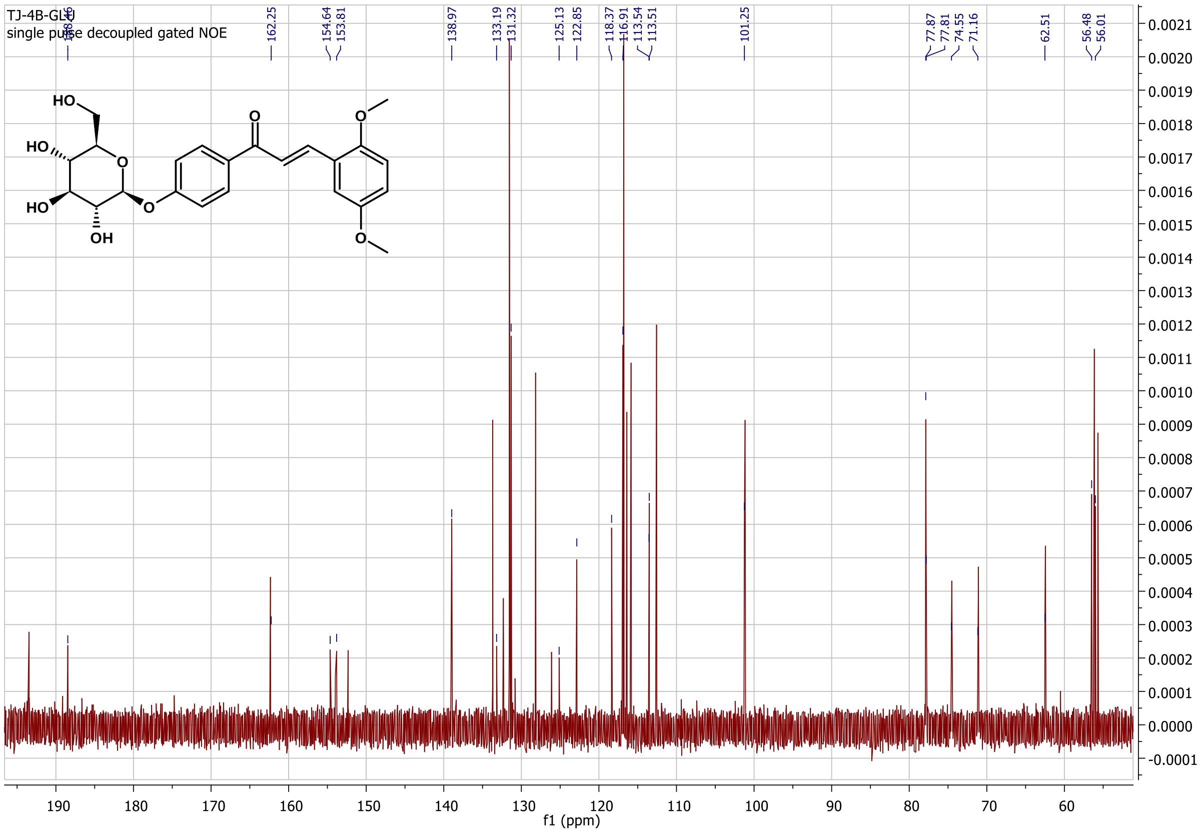


**Figure S92** ¹³C NMR spectrum of *cis*-4′-*O*-β-D-(glucopyranosyl)-2,5-dimethoxychalcone (151MHz; Acetone-d₆)

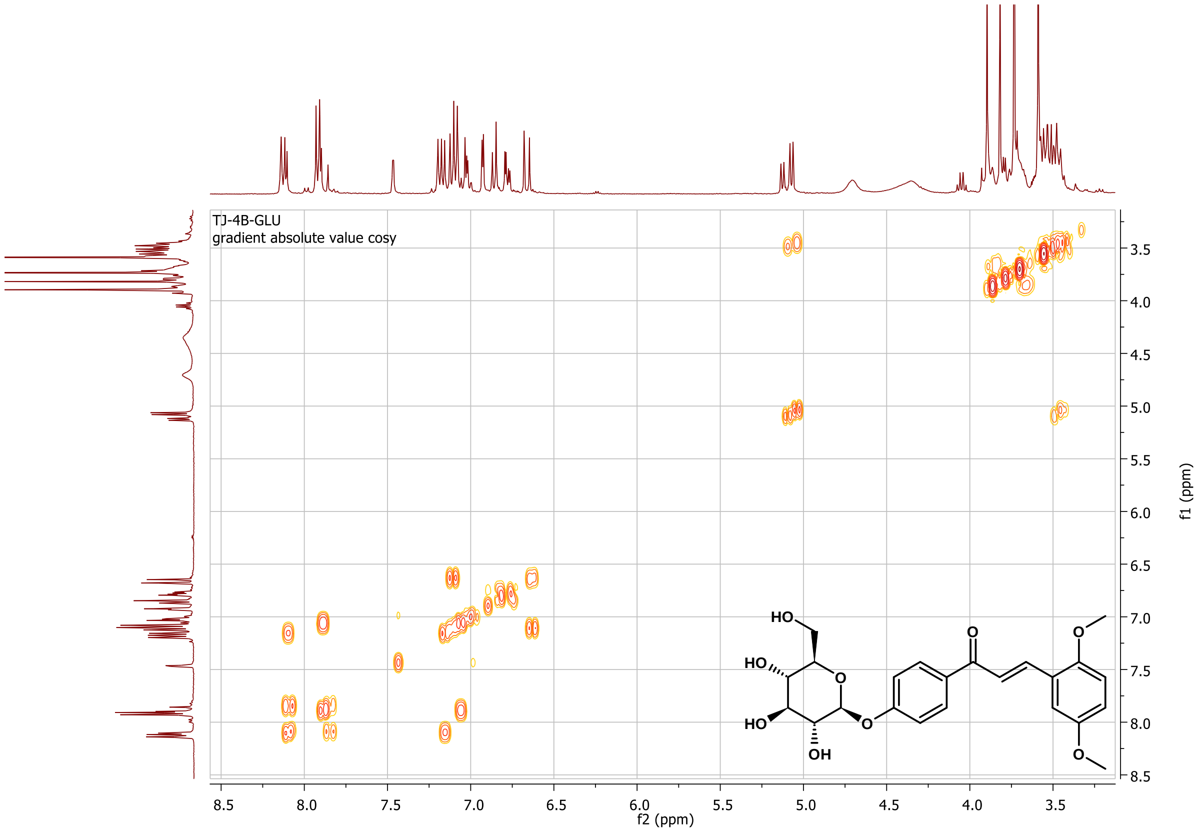


**Figure S93** COSY NMR spectrum of *cis*-4′-*O*-β-D-(glucopyranosyl)-2,5-dimethoxychalcone (600MHz; Acetone-d6)

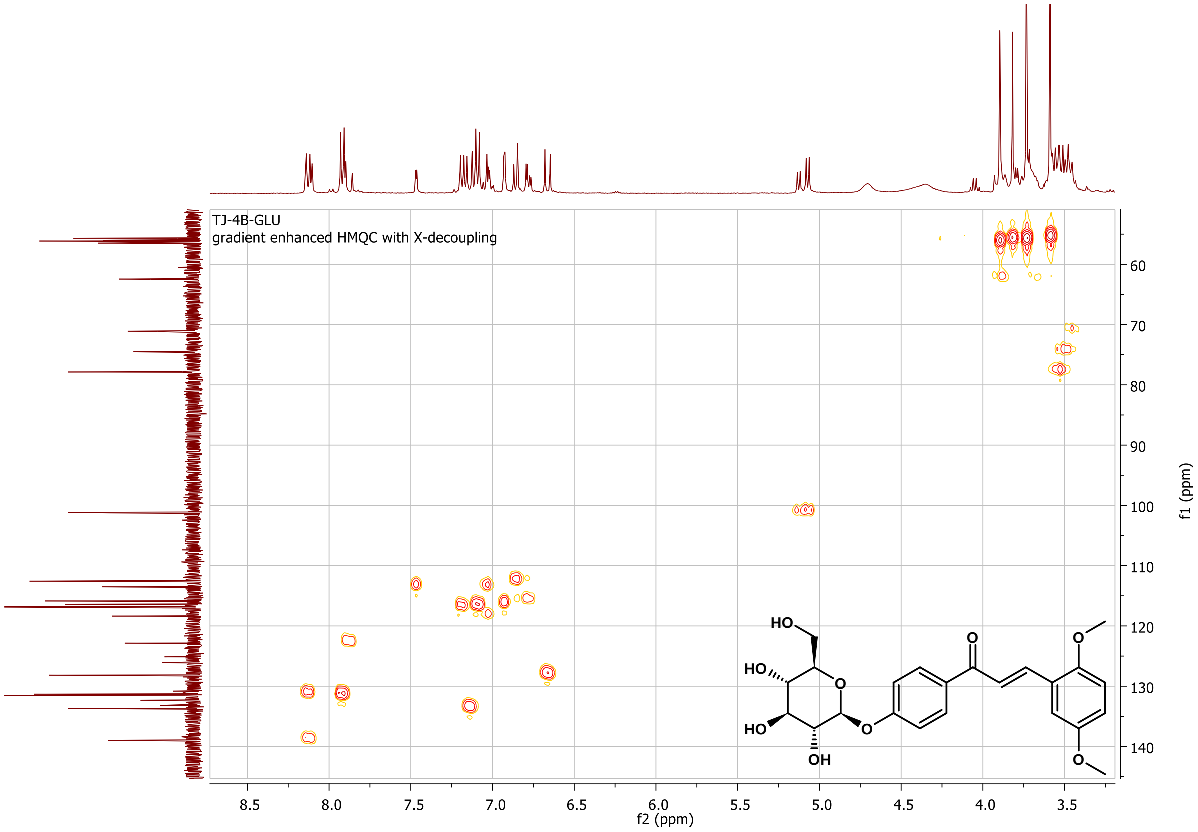


**Figure S94** HMQC NMR spectrum of *cis*-4′-*O*-β-D-(glucopyranosyl)-2,5-dimethoxychalcone (600MHz; Acetone-d6)

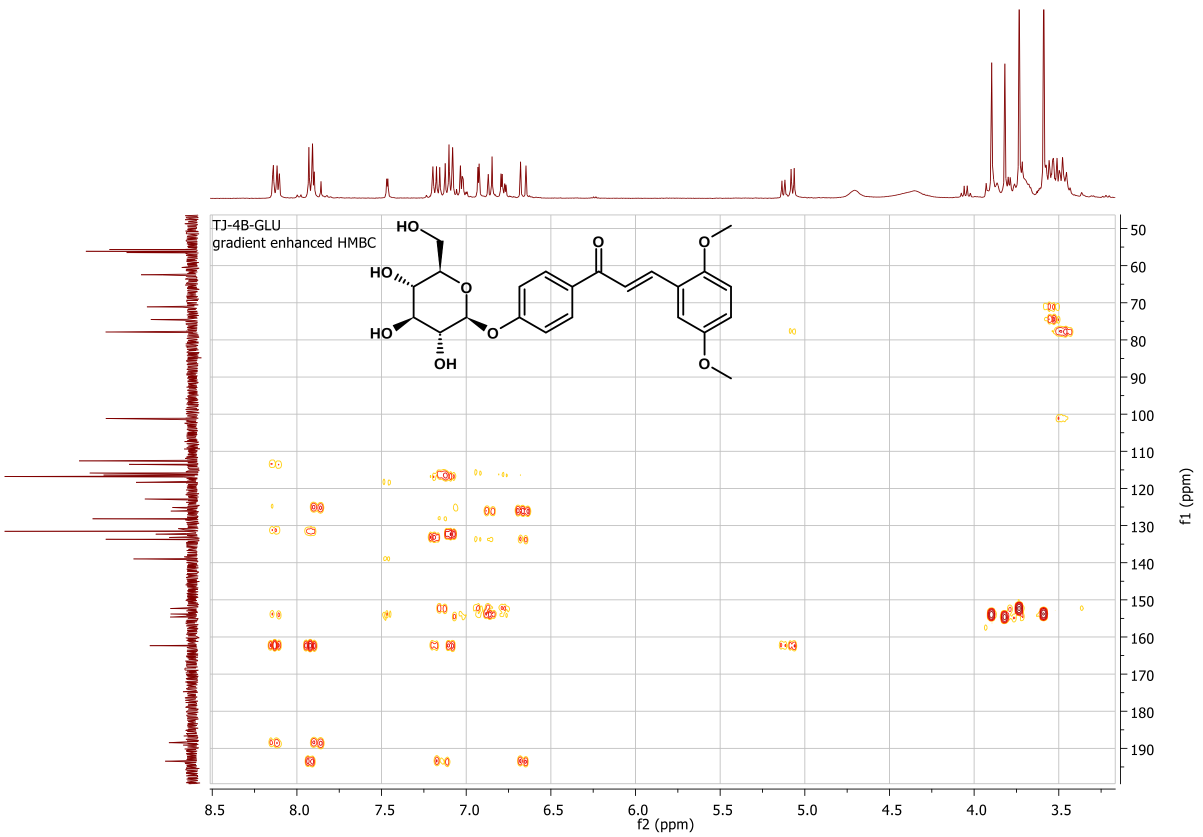


**Figure S95** HMBC NMR spectrum of *cis*-4′-*O*-β-D-(glucopyranosyl)-2,5-dimethoxychalcone (600MHz; Acetone-d$_6$)

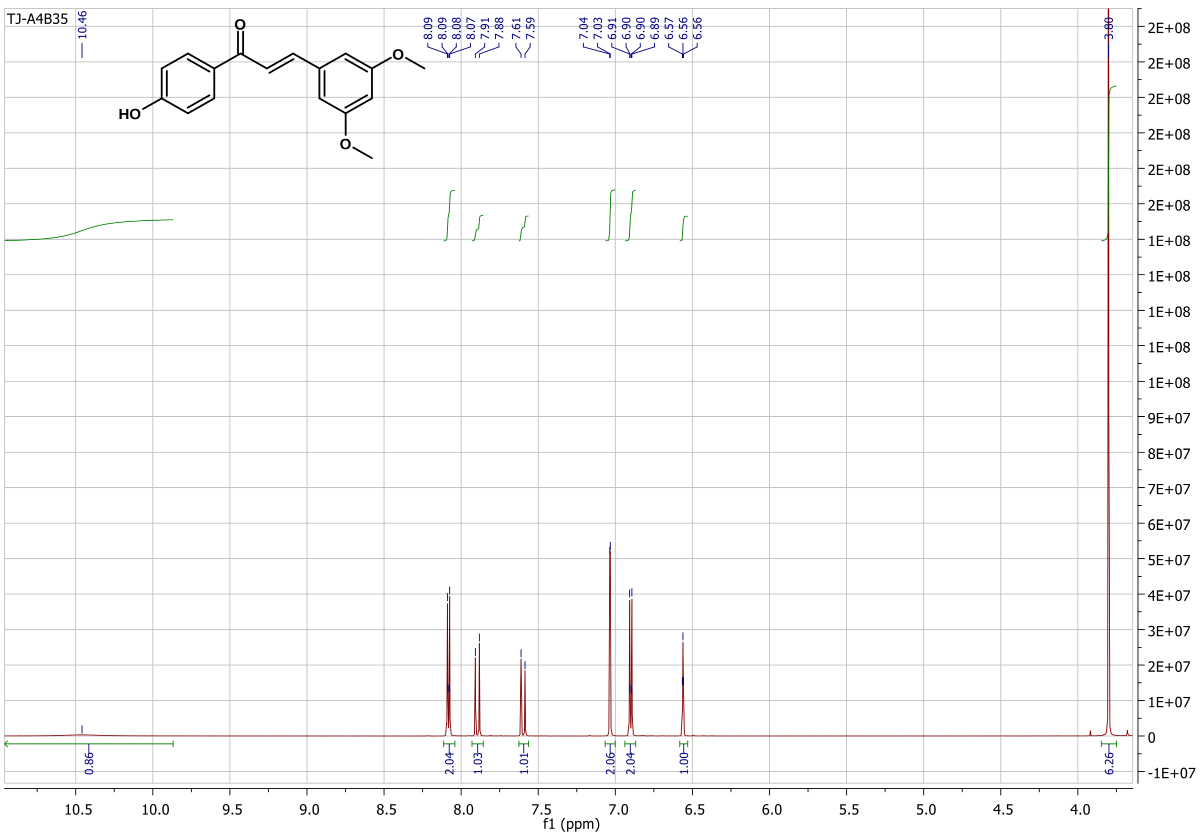


**Figure S96** $^{1}$H NMR spectrum of *trans*-4′-hydroxy-3,5-dimethoxychalcone (600MHz, DMSO-d$_6$)

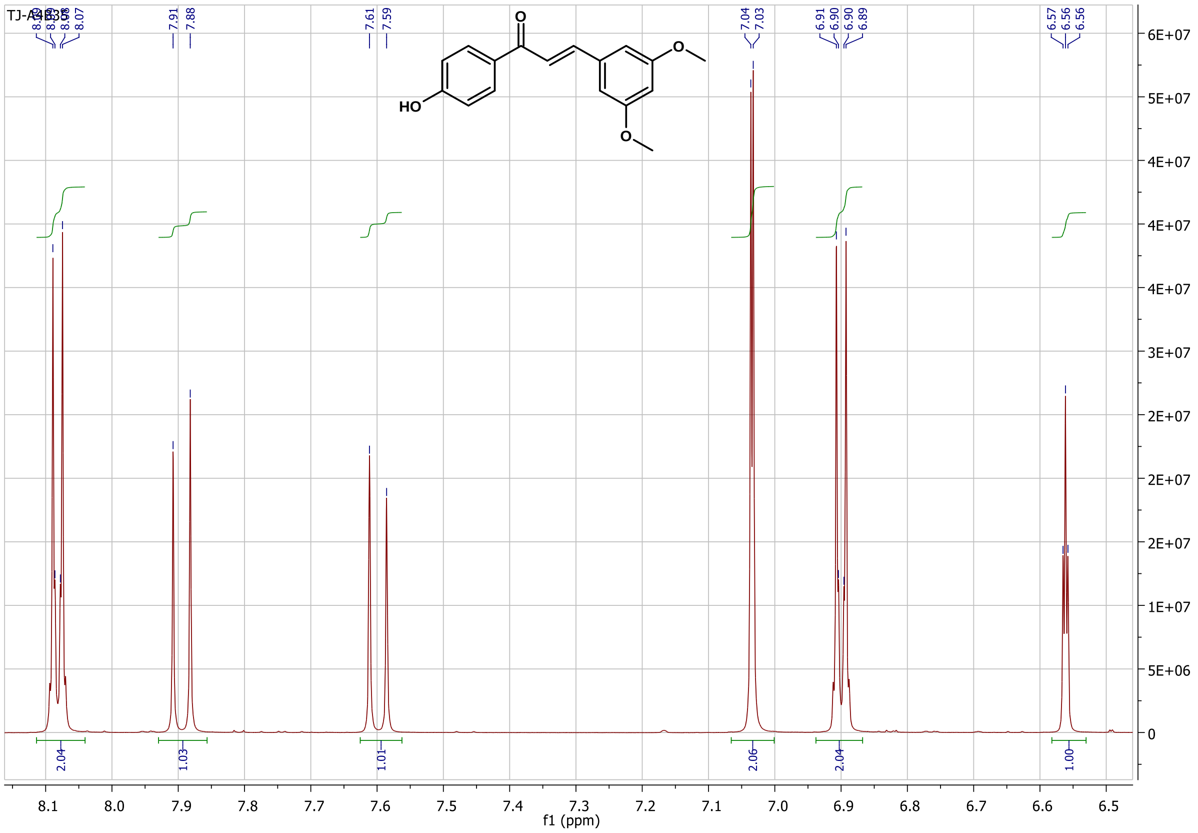


**Figure S97** Flavonoid part of spectrum of $^{1}$H NMR of *trans*-4′-hydroxy-3,5-dimethoxychalcone (600MHz, DMSO-d$_6$)

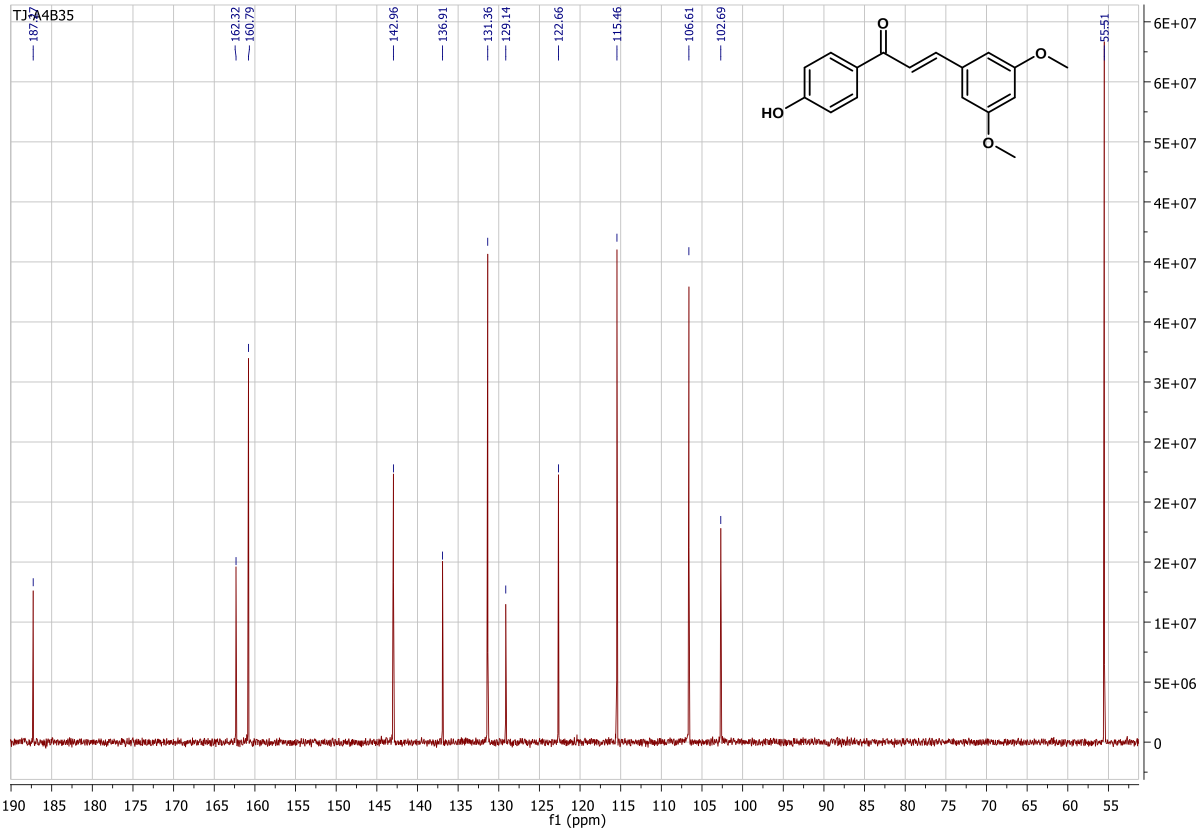


**Figure S98** $^{13}$C NMR spectrum of *trans*-4′-hydroxy-3,5-dimethoxychalcone (151MHz, DMSO-d$_6$)

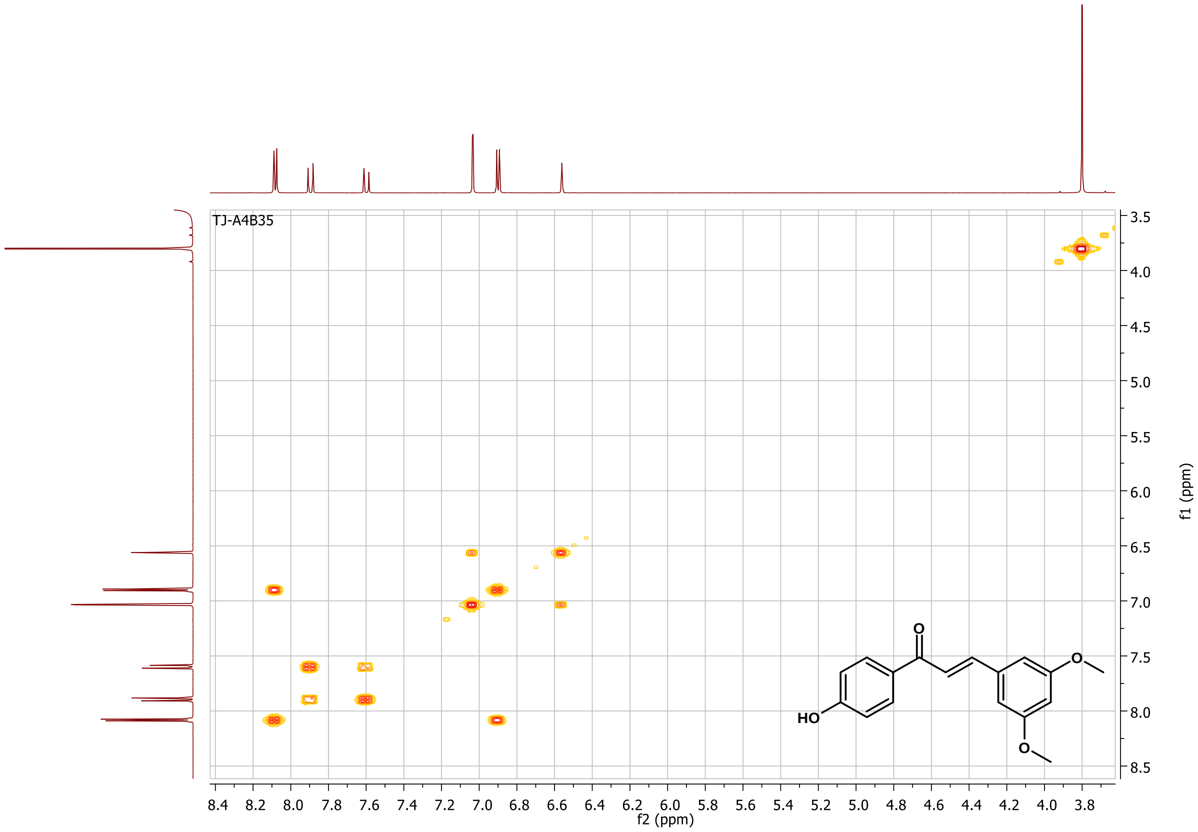


**Figure S99** COSY NMR spectrum of *trans*-4′-hydroxy-3,5-dimethoxychalcone (600MHz, DMSO-d6)

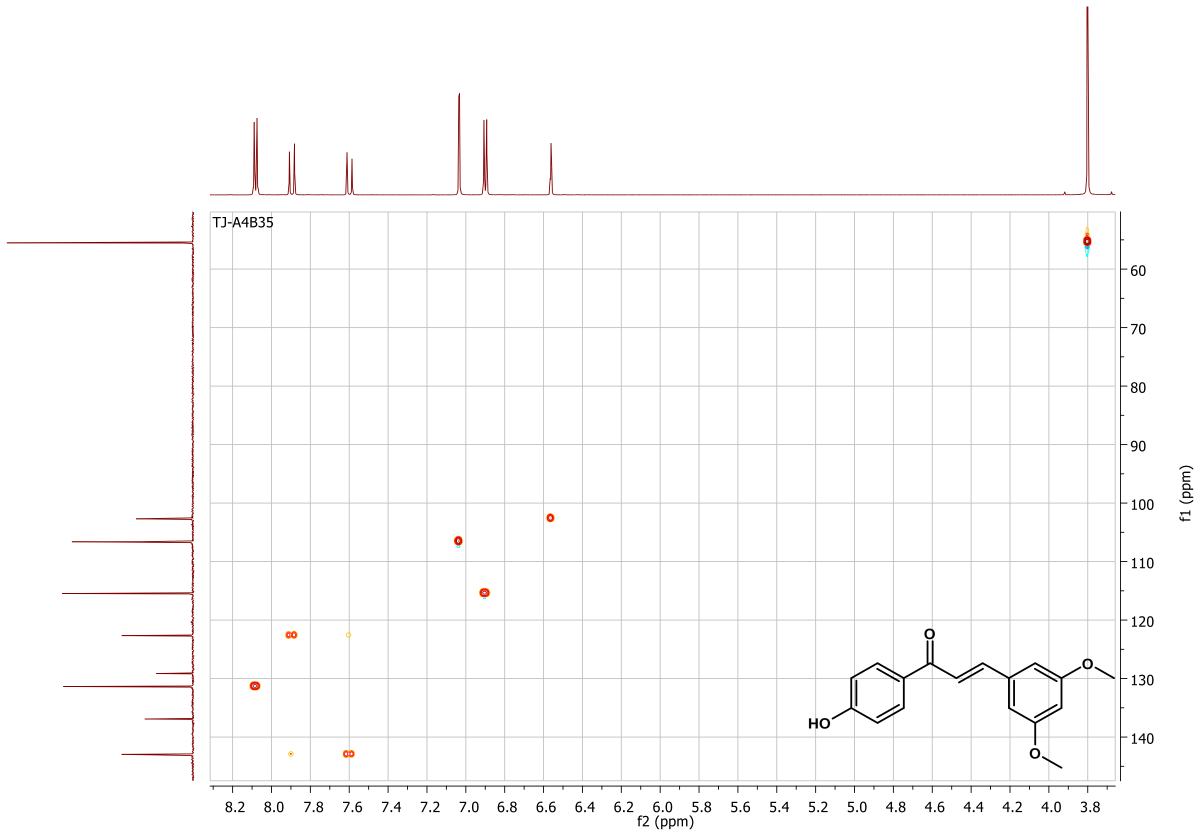


**Figure S100** HMQC NMR spectrum of *trans*-4′-hydroxy-3,5-dimethoxychalcone (600MHz, DMSO-d6)

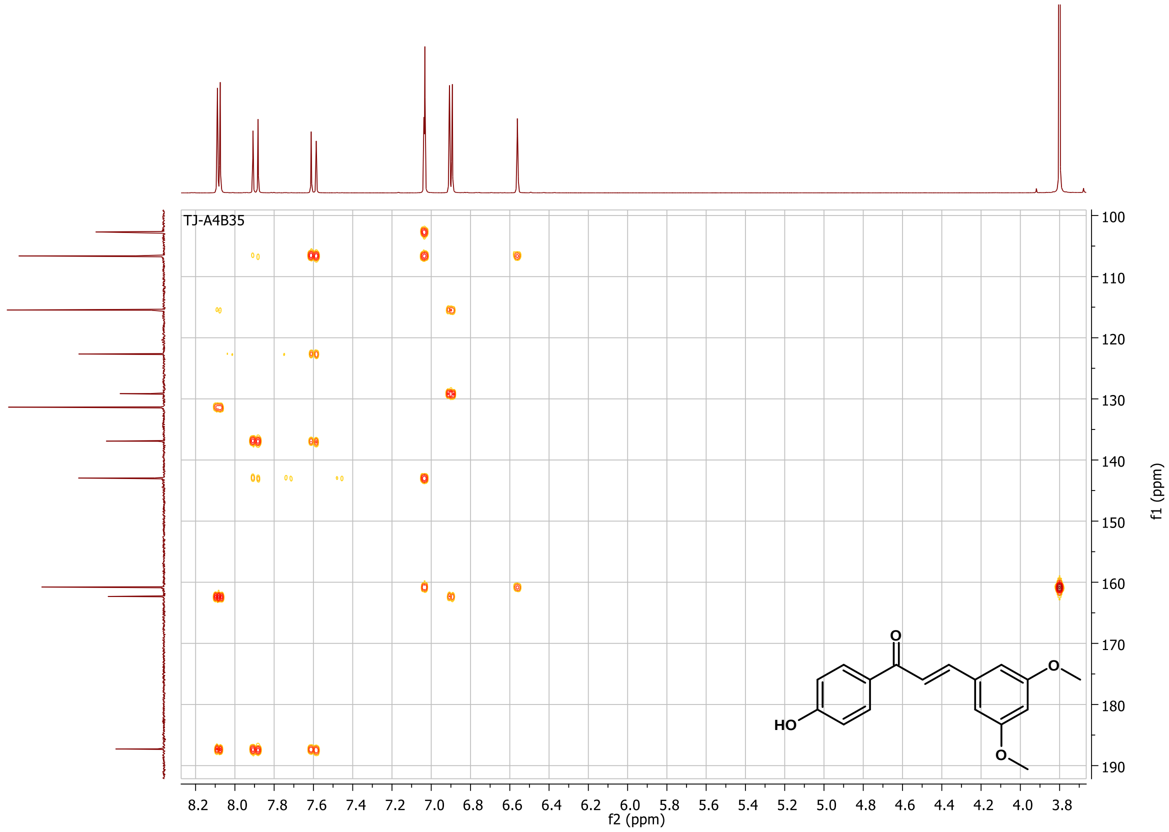

**Figure S101** HMBC NMR spectrum of *trans*-4′-hydroxy-3,5-dimethoxychalcone (600MHz, DMSO-d6)

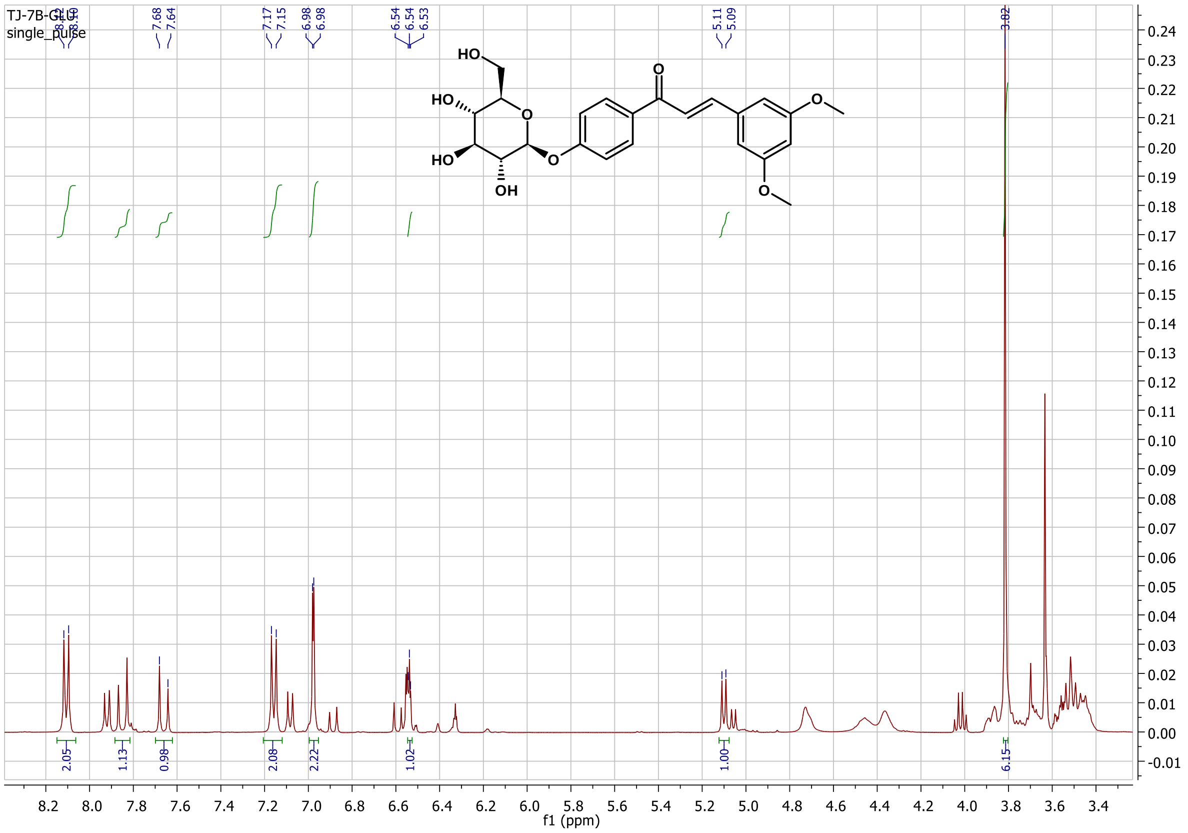

**Figure S102** $^{1}$H NMR spectrum of *trans*-4′-*O*-β-D-(glucopyranosyl)-3,5-dimethoxychalcone (600MHz, DMSO-d6)

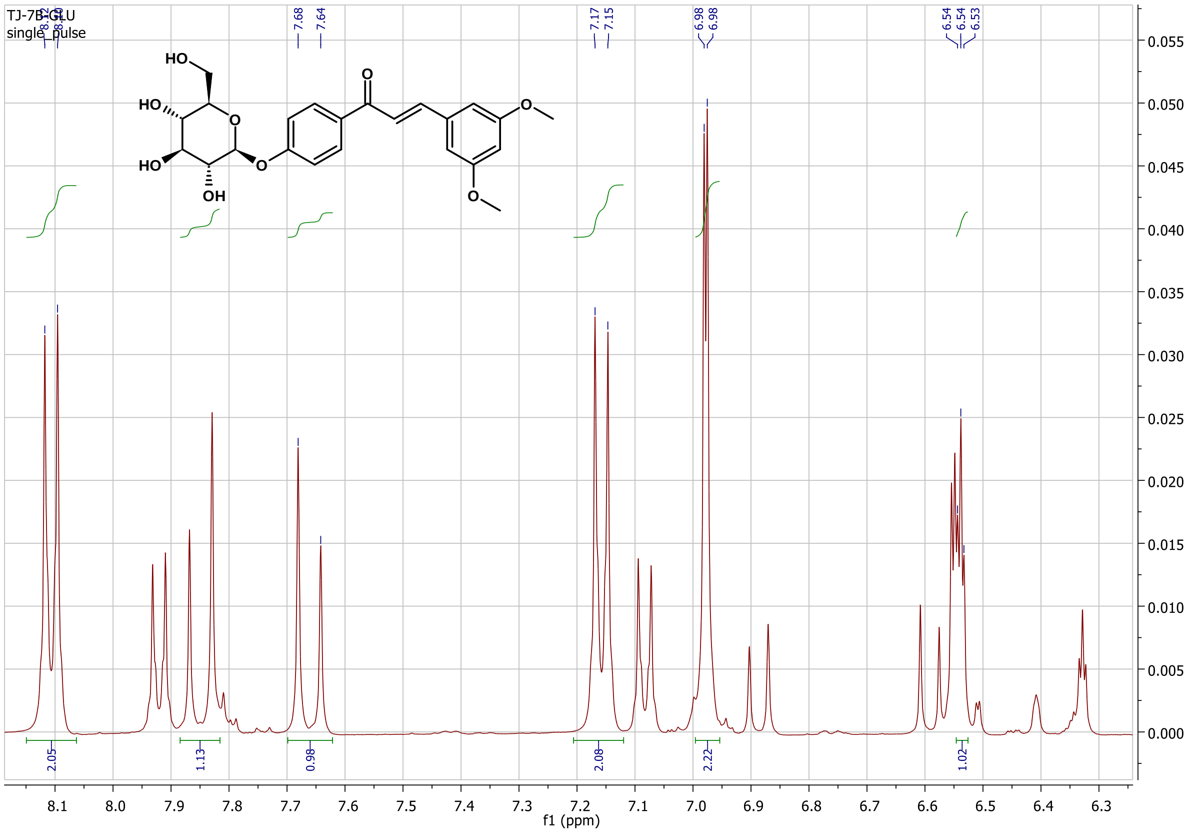

**Figure S103** Flavonoid part of ¹H NMR spectrum of *trans*-4′-*O*-β-D-(glucopyranosyl)-3,5-dimethoxychalcone (600MHz, Acetone-d$_6$)

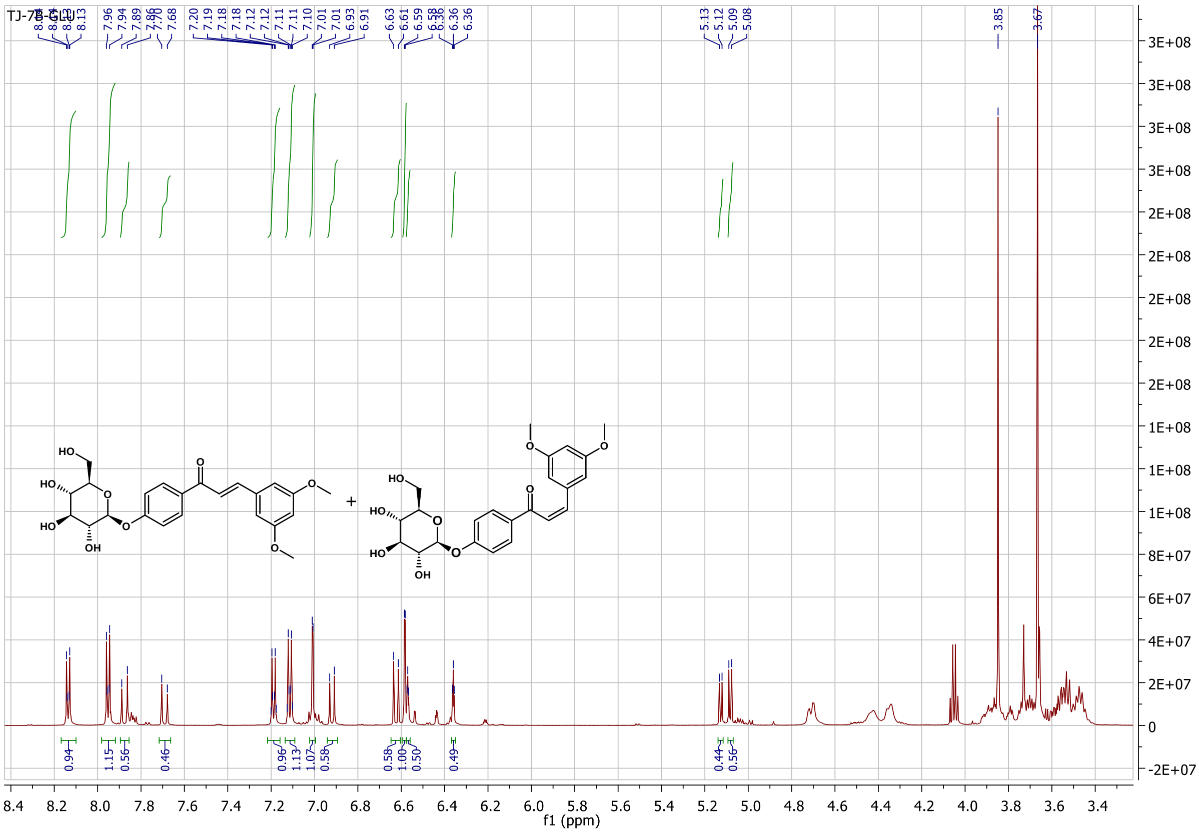

**Figure S104** ¹H NMR spectrum of mixture of *trans*- and *cis*-4′-*O*-β-D-(glucopyranosyl)-3,5-dimethoxychalcone (same probe as **Figure S102** measured second time after few days) (600MHz, Acetone-d$_6$)

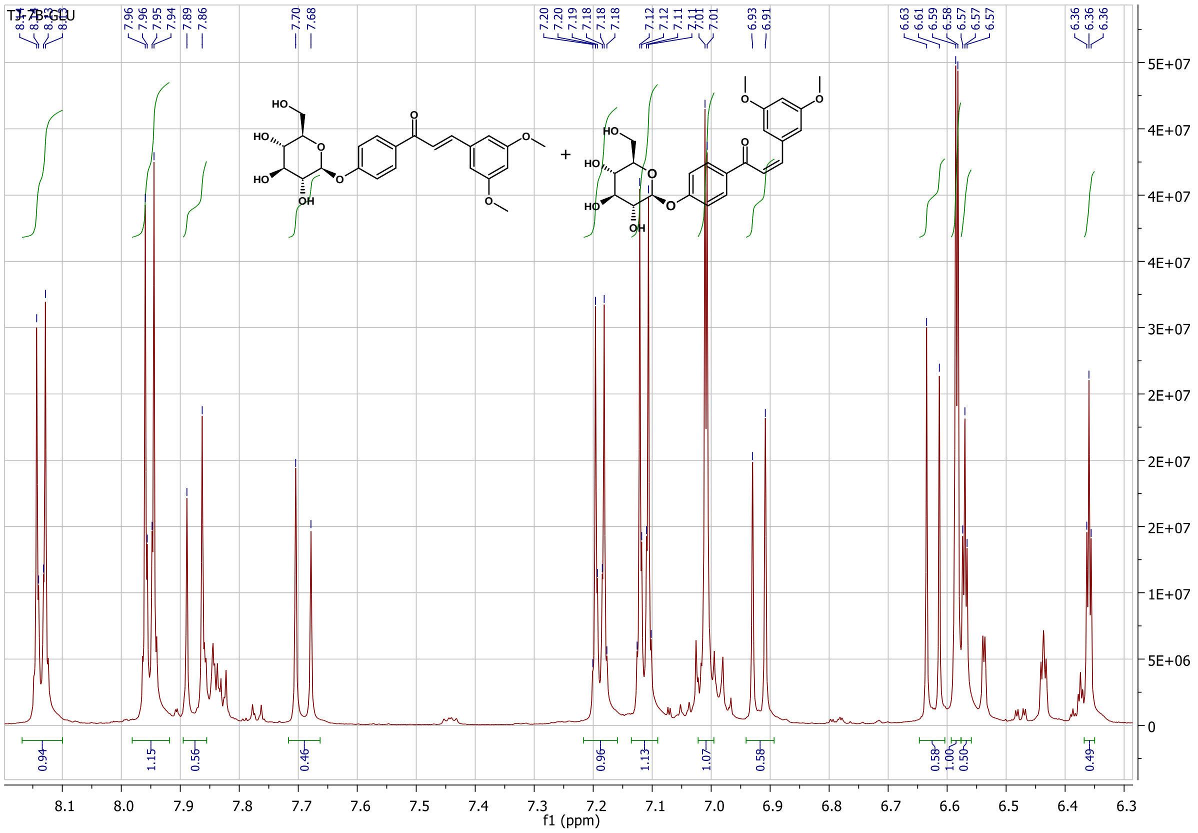

**Figure S105** Flavonoid part of $^{1}$H NMR spectrum of *trans*- and *cis*-4′-*O*-β-D-(glucopyranosyl)-3,5-dimethoxychalcone (same probe as **Figure S103** measured second time after few days) (600MHz, Acetone-d$_6$)

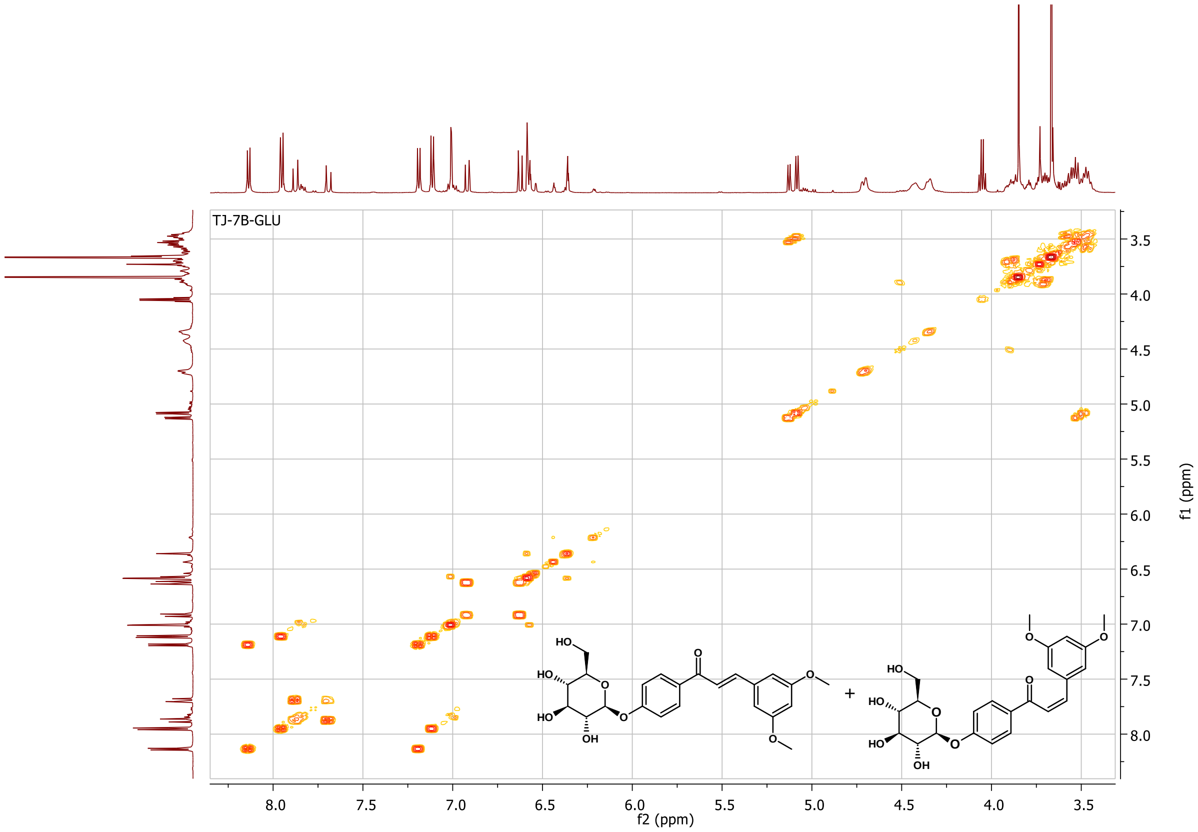

**Figure S106** COSY NMR spectrum of *trans*- and *cis*-4′-*O*-β-D-(glucopyranosyl)-3,5-dimethoxychalcone (600MHz, Acetone-d$_6$)

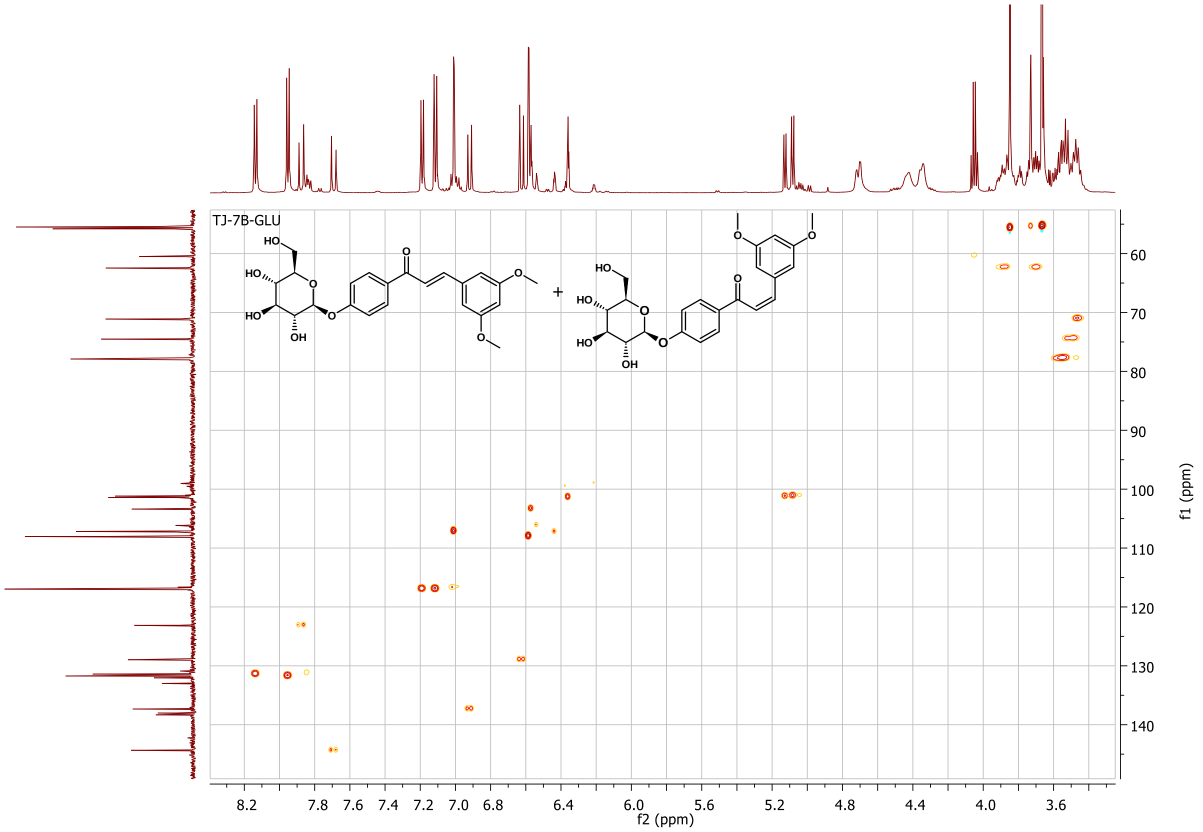


**Figure S107** HMQC NMR spectrum of *trans*- and *cis*-4′-*O*-β-D-(glucopyranosyl)-3,5-dimethoxychalcone (600MHz, Acetone-d6)

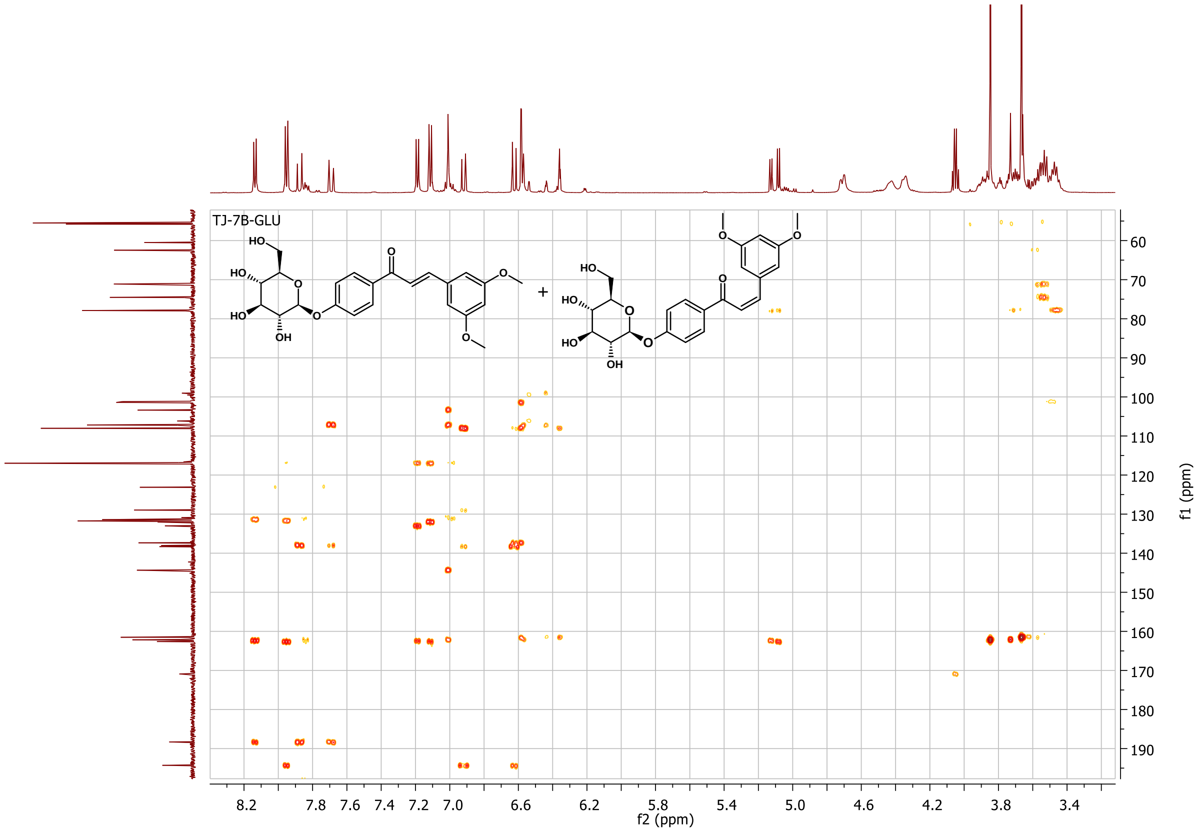


**Figure S108** HMBC NMR spectrum of *trans*- and *cis*-4′-*O*-β-D-(glucopyranosyl)-3,5-dimethoxychalcone (600MHz, Acetone-d6)

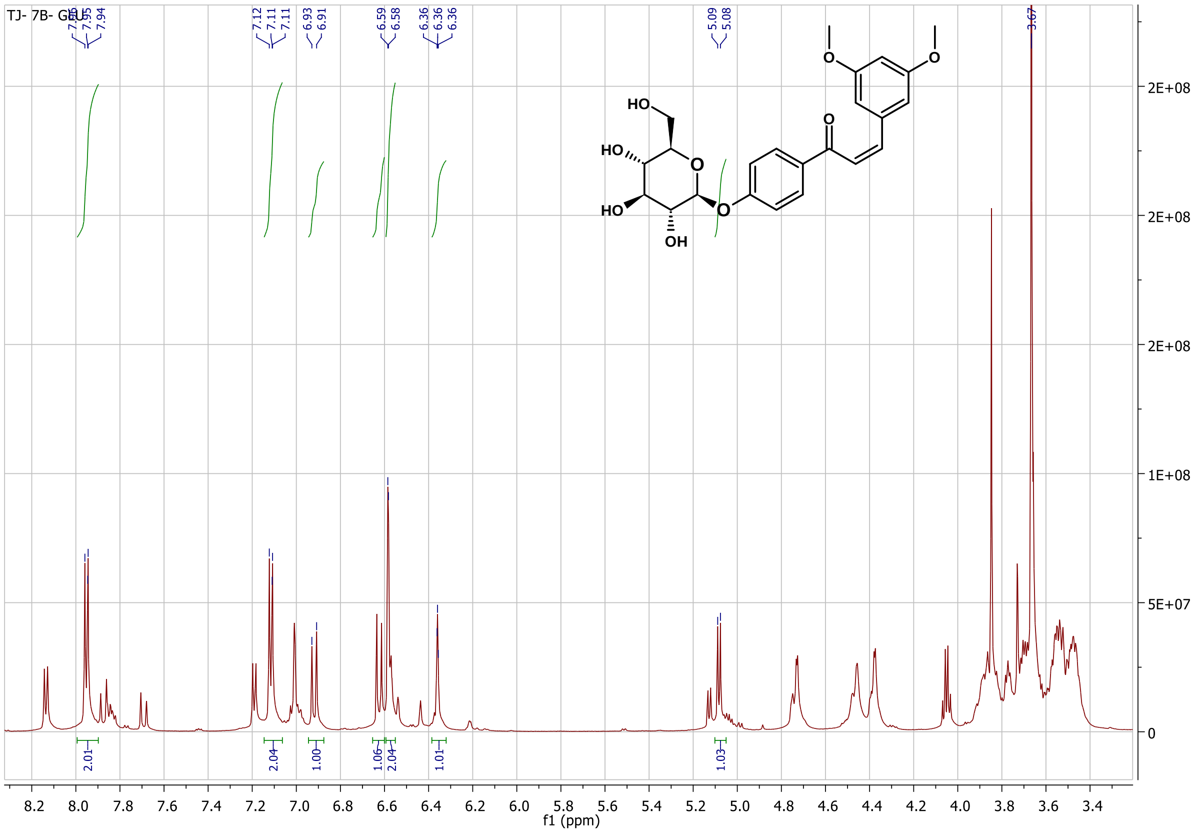


**Figure S109** $^{1}$H NMR spectrum of *cis*-4′-*O*-β-D-(glucopyranosyl)-3,5-dimethoxychalcone (600MHz, Acetone-d$_6$)

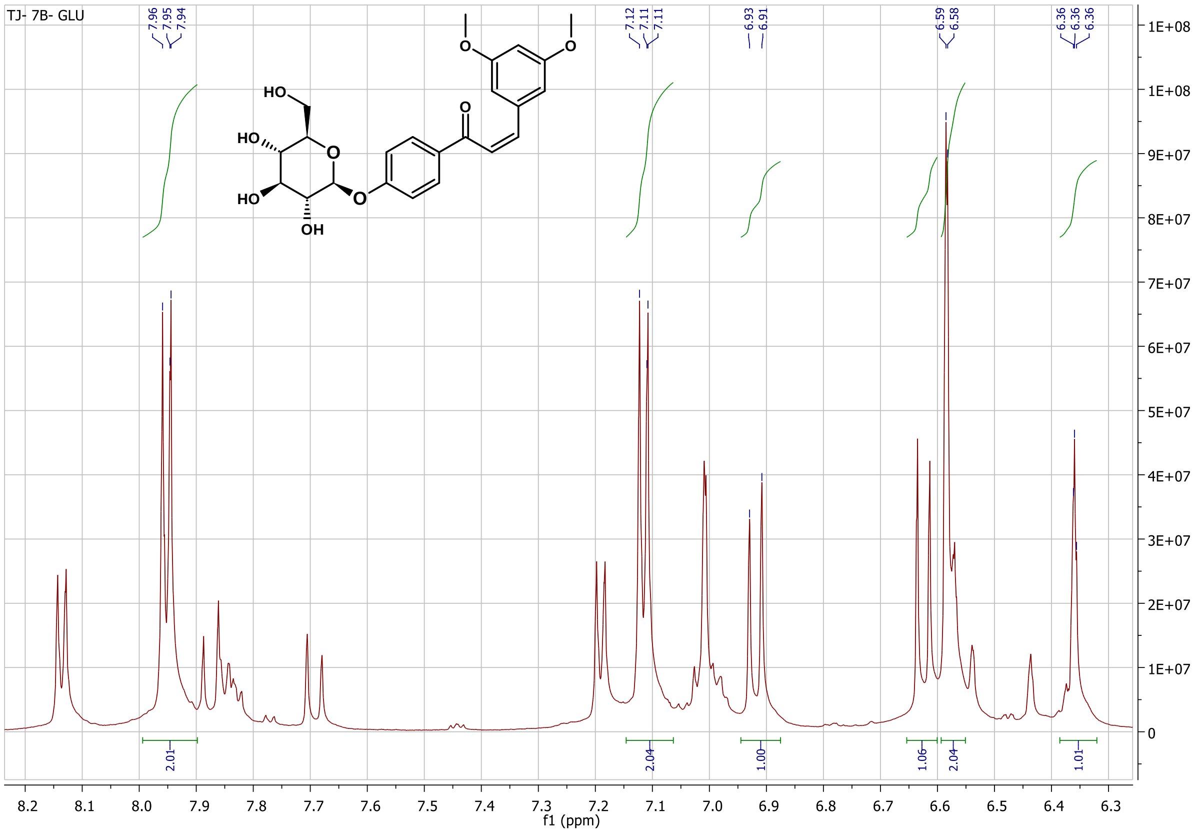


**Figure S110** Flavonoid part of spectrum of $^{1}$H NMR spectrum of *cis*-4′-*O*-β-D-(glucopyranosyl)-3,5-dimethoxychalcone (600MHz, Acetone-d$_6$)

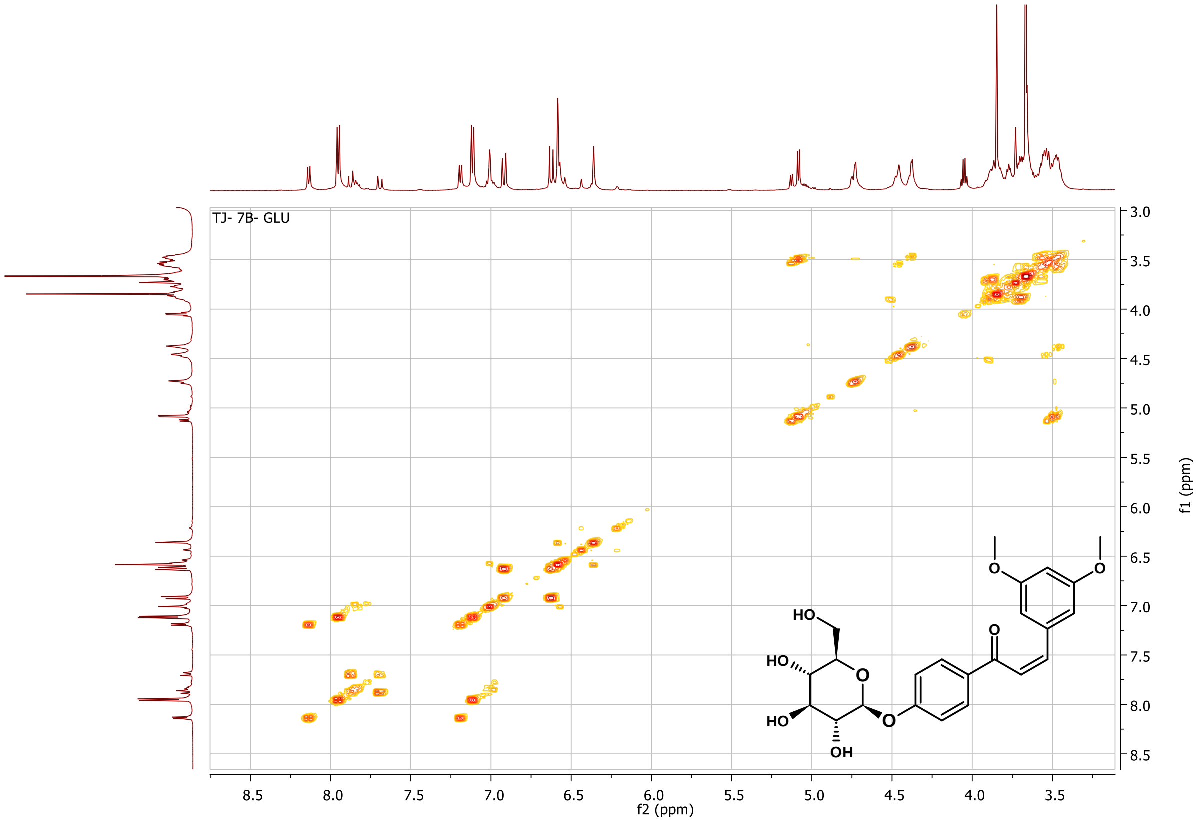


**Figure S111** COSY NMR spectrum of ¹H NMR spectrum of *cis*-4′-*O*-β-D-(glucopyranosyl)-3,5-dimethoxychalcone (600MHz, Acetone-$d_6$)

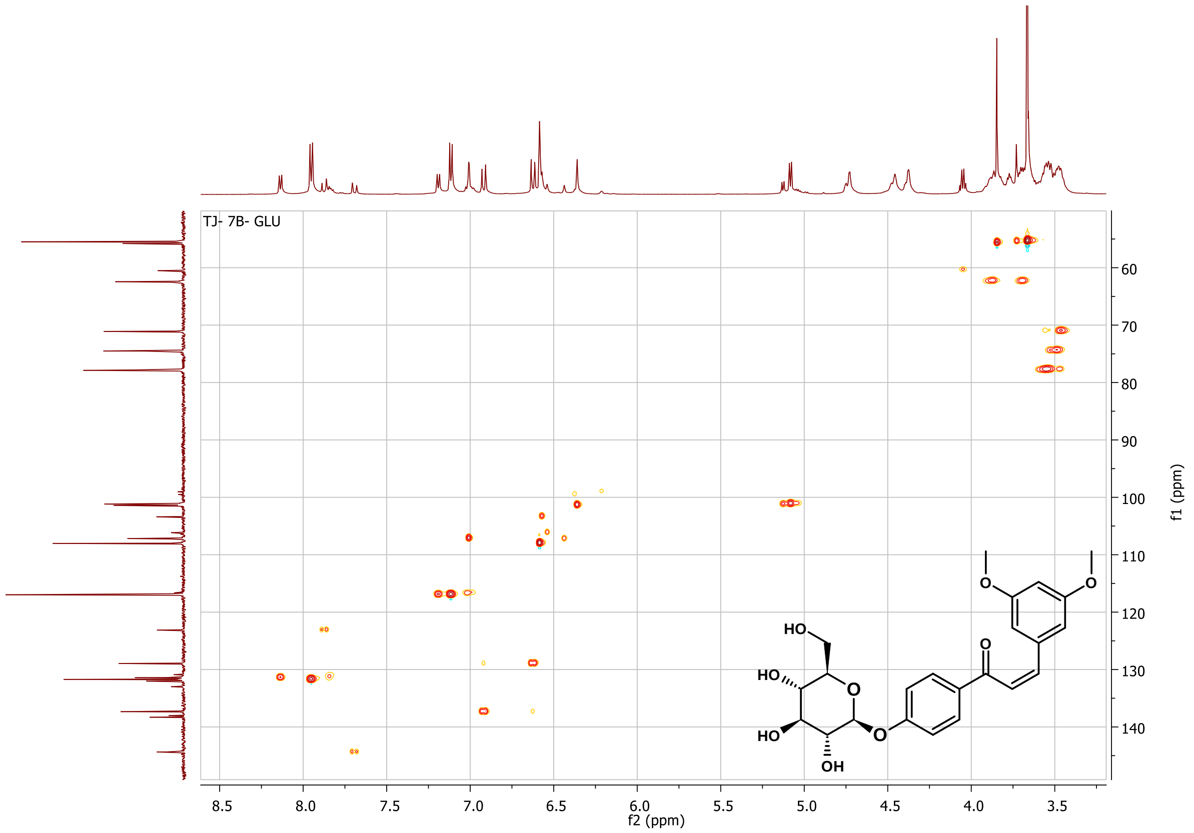


**Figure S112** HMQC NMR spectrum of *cis*-4′-*O*-β-D-(glucopyranosyl)-3,5-dimethoxychalcone (600MHz, Acetone-$d_6$)

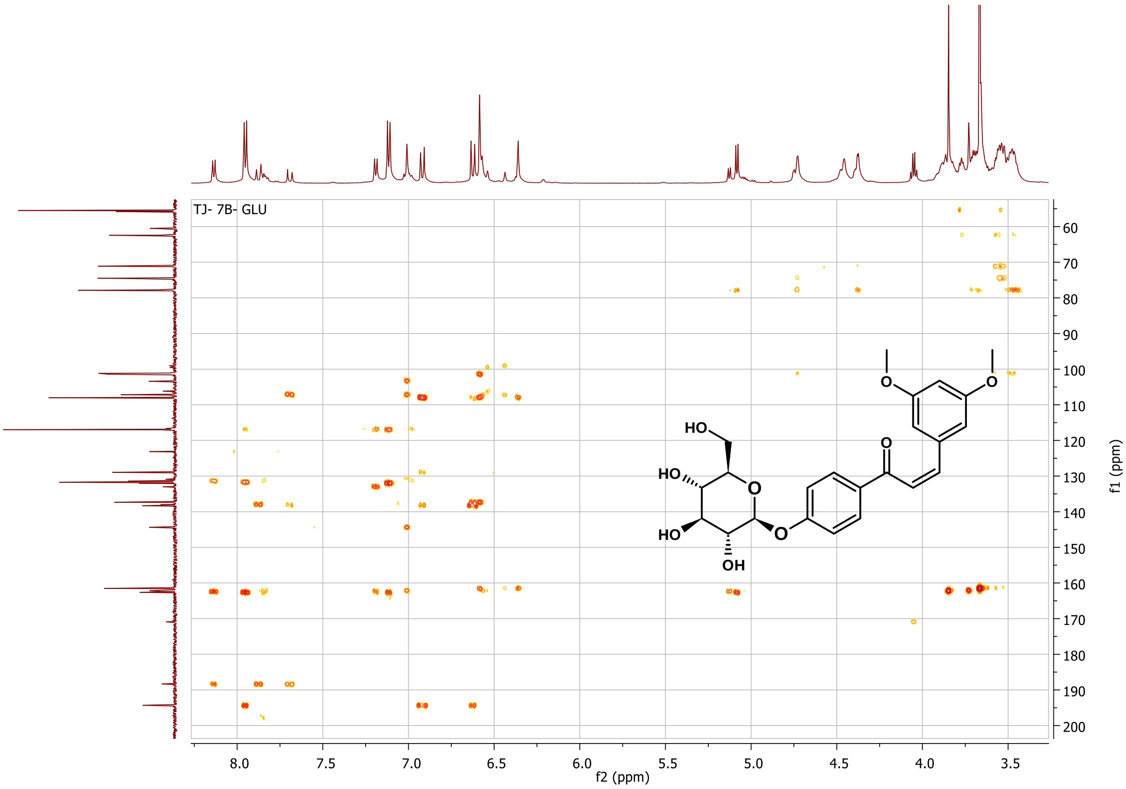


**Figure S113** HMBC NMR spectrum of *cis*-4′-*O*-β-D-(glucopyranosyl)-3,5-dimethoxychalcone (600MHz, Acetone-d6)

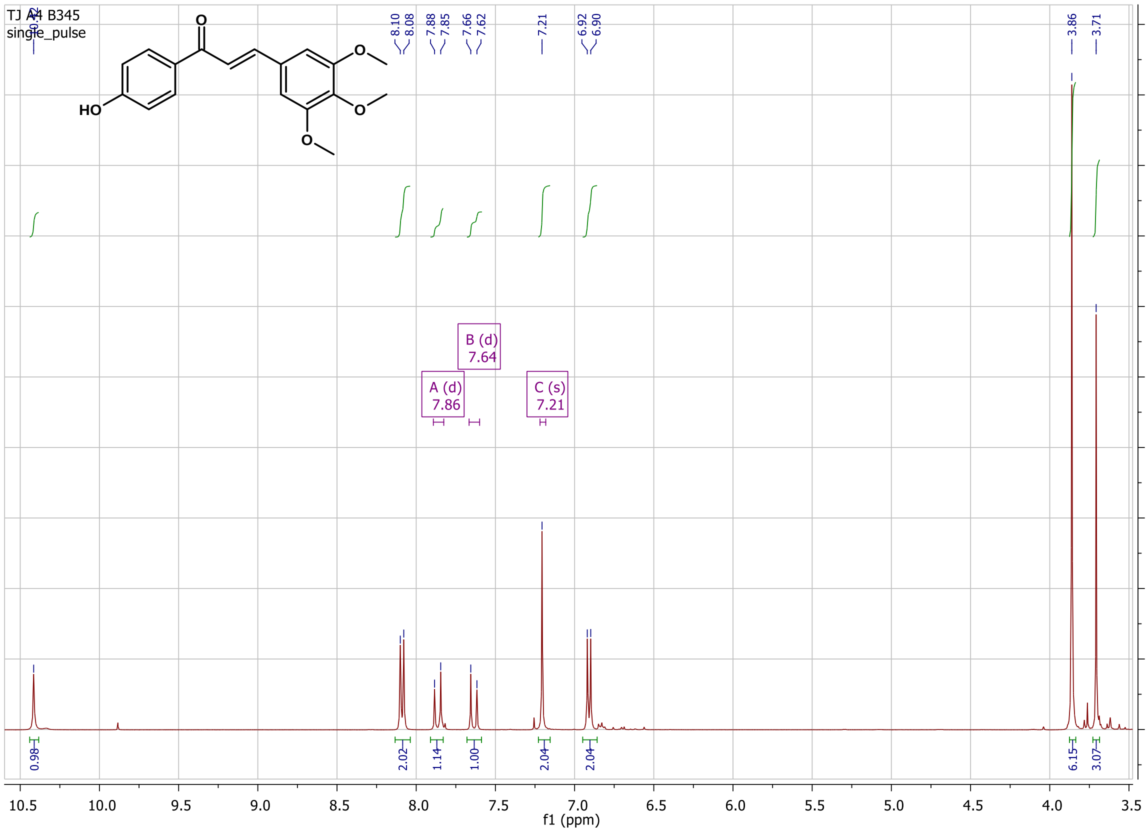


**Figure S114** ¹H NMR of *trans*-4′-hydroxy-3,4,5-trimethoxychalcone (600MHz, DMSO-d6)

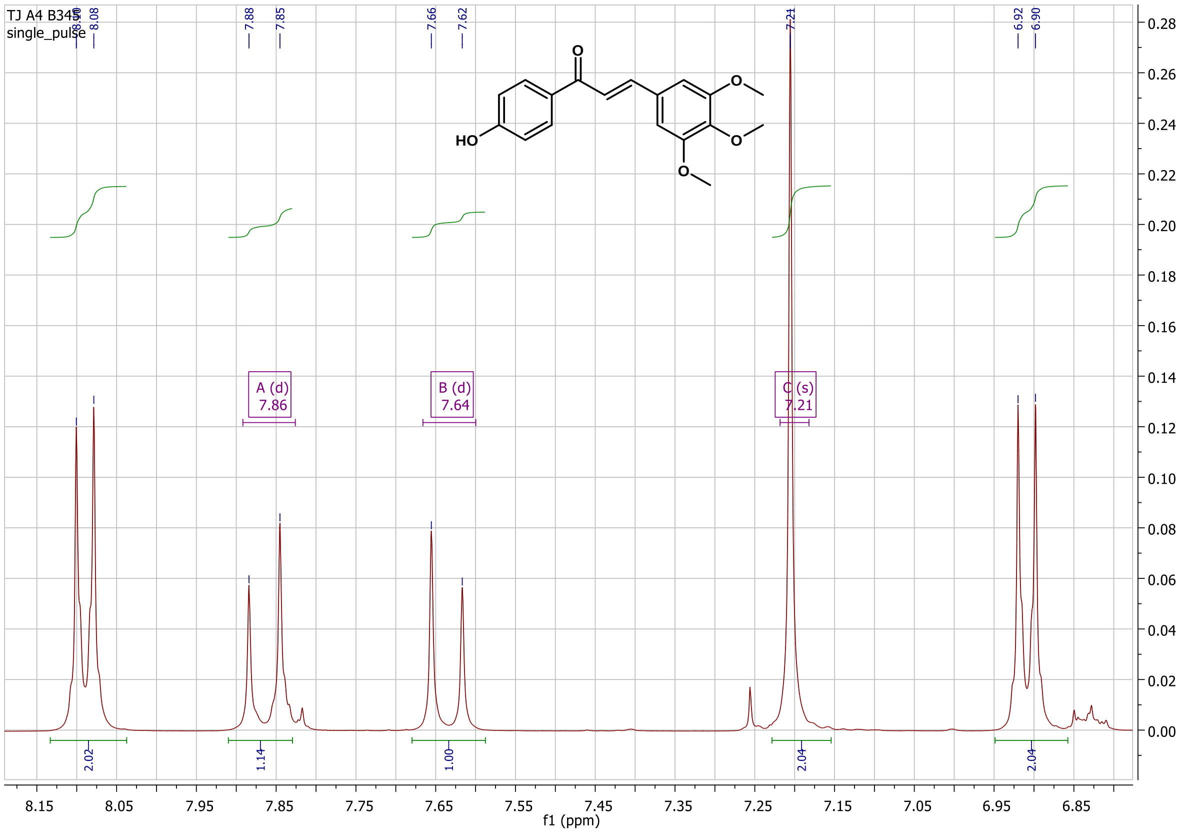

**Figure S115** Flavonoid framgment of $^{1}H$ NMR spectrum of *trans*-4′-hydroxy-3,4,5-trimethoxychalcone (600MHz, DMS*O*-d$_6$)

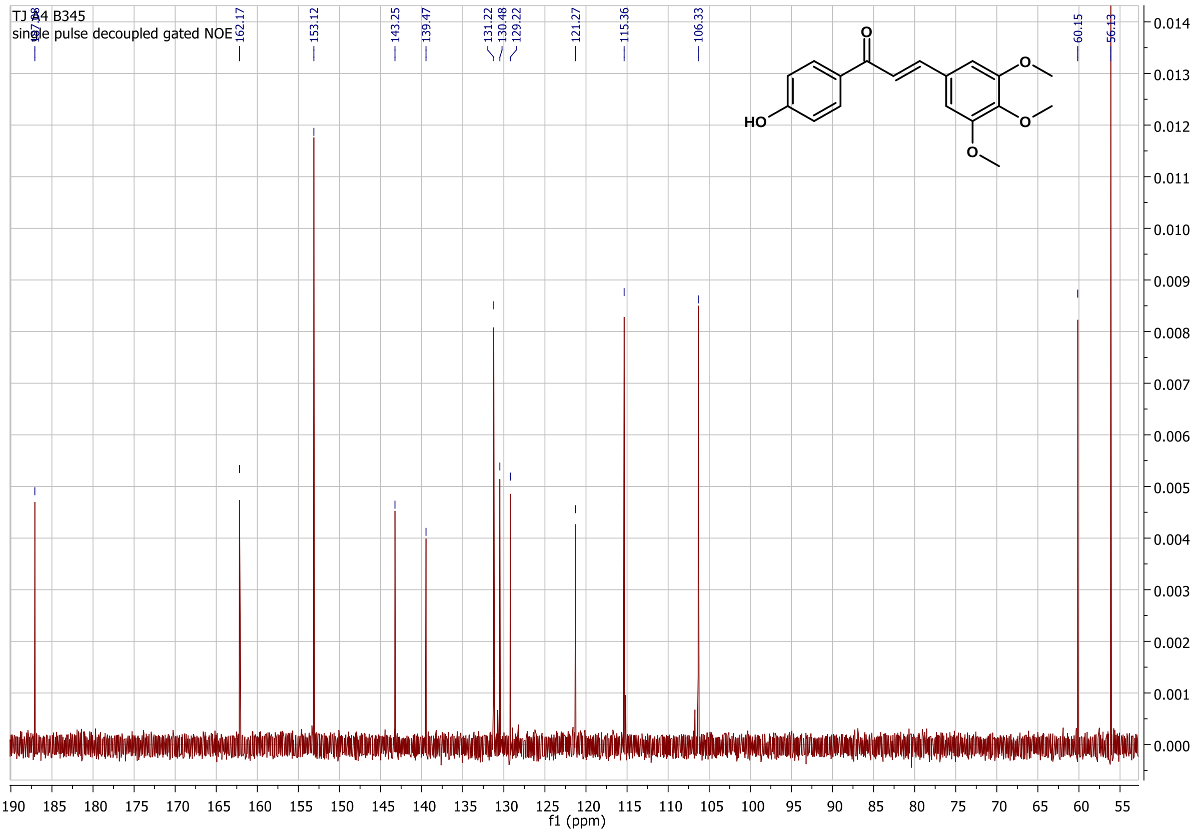

**Figure S116** $^{13}C$ NMR of *trans*-4′-hydroxy-3,4,5-trimethoxychalcone (151MHz, DMS*O*-d$_6$)

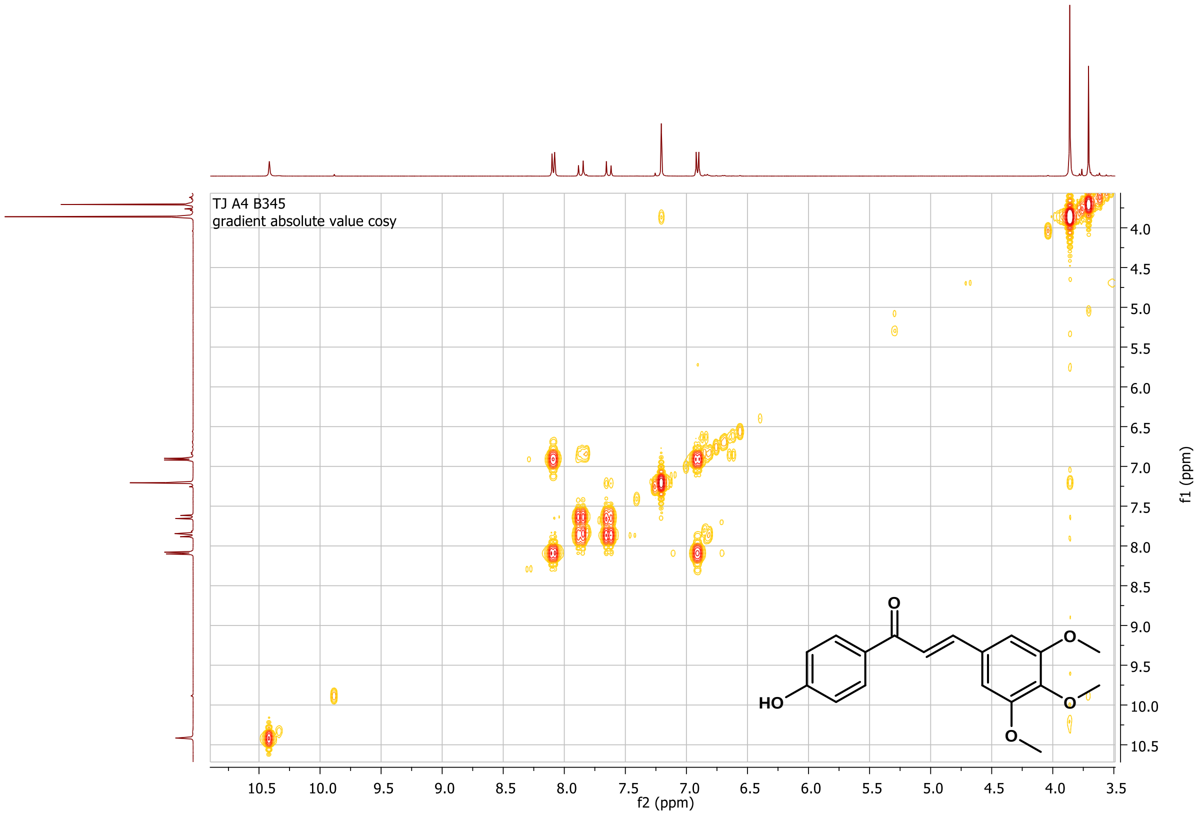


**Figure S117** COSY NMR spectrum of *trans*-4′-hydroxy-3,4,5-trimethoxychalcone (600MHz, DMSO-d$_6$)

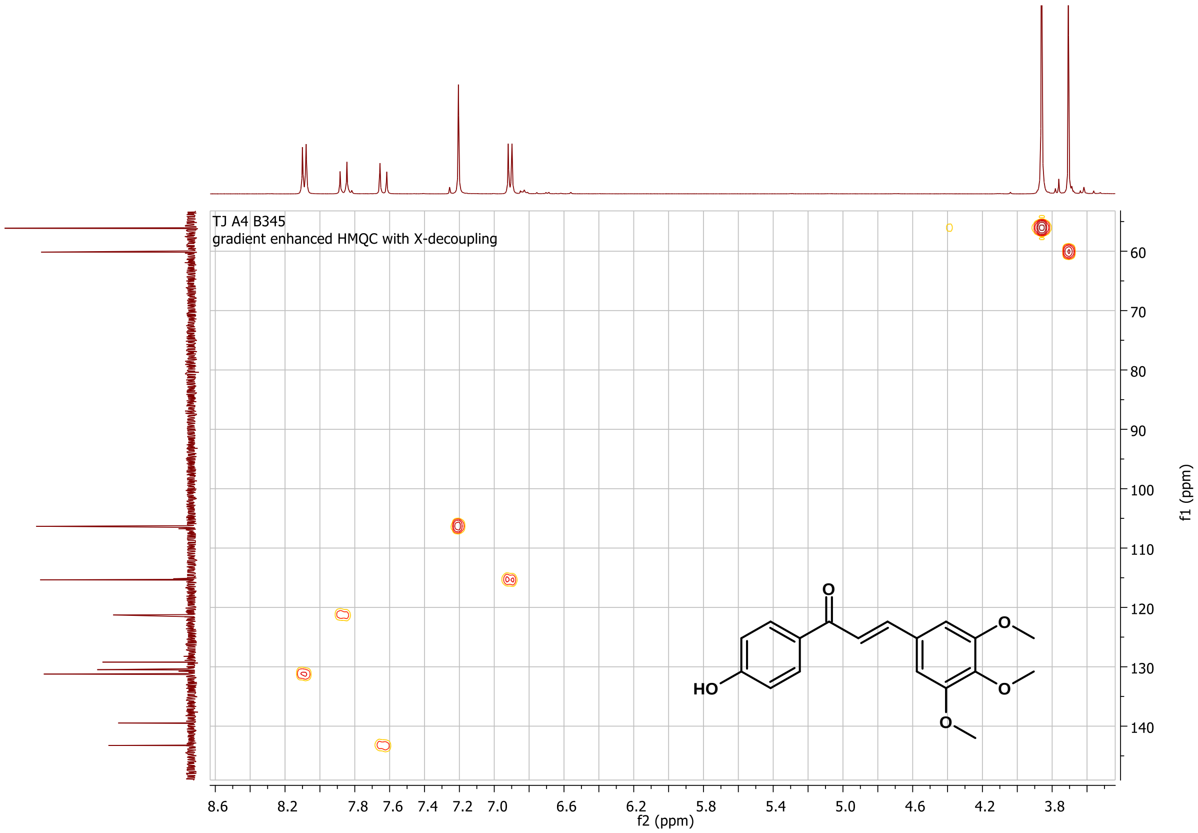


**Figure S118** HMQC NMR spectrum of *trans*-4′-hydroxy-3,4,5-trimethoxychalcone (600MHz, DMSO-d$_6$)

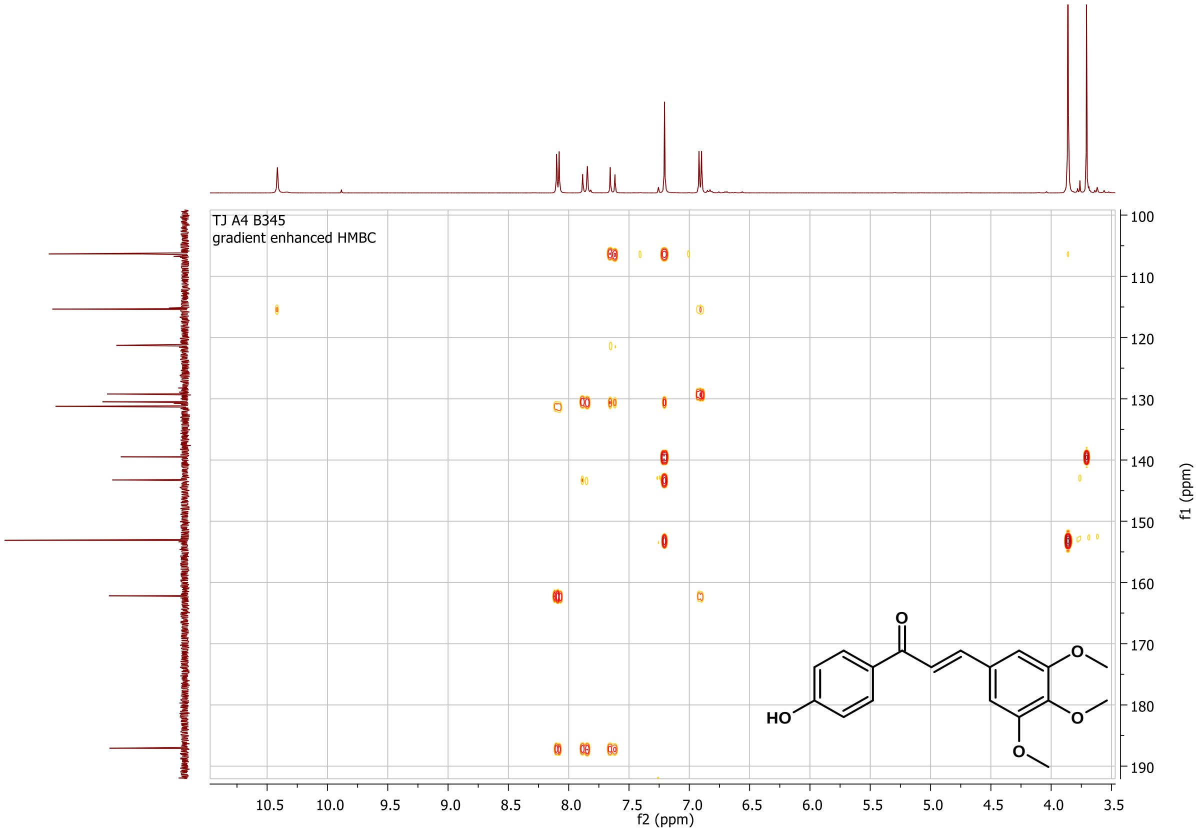


**Figure S119** HMBC NMR spectrum of *trans*-4′-hydroxy-3,4,5-trimethoxychalcone (600MHz, DMSO-d6)

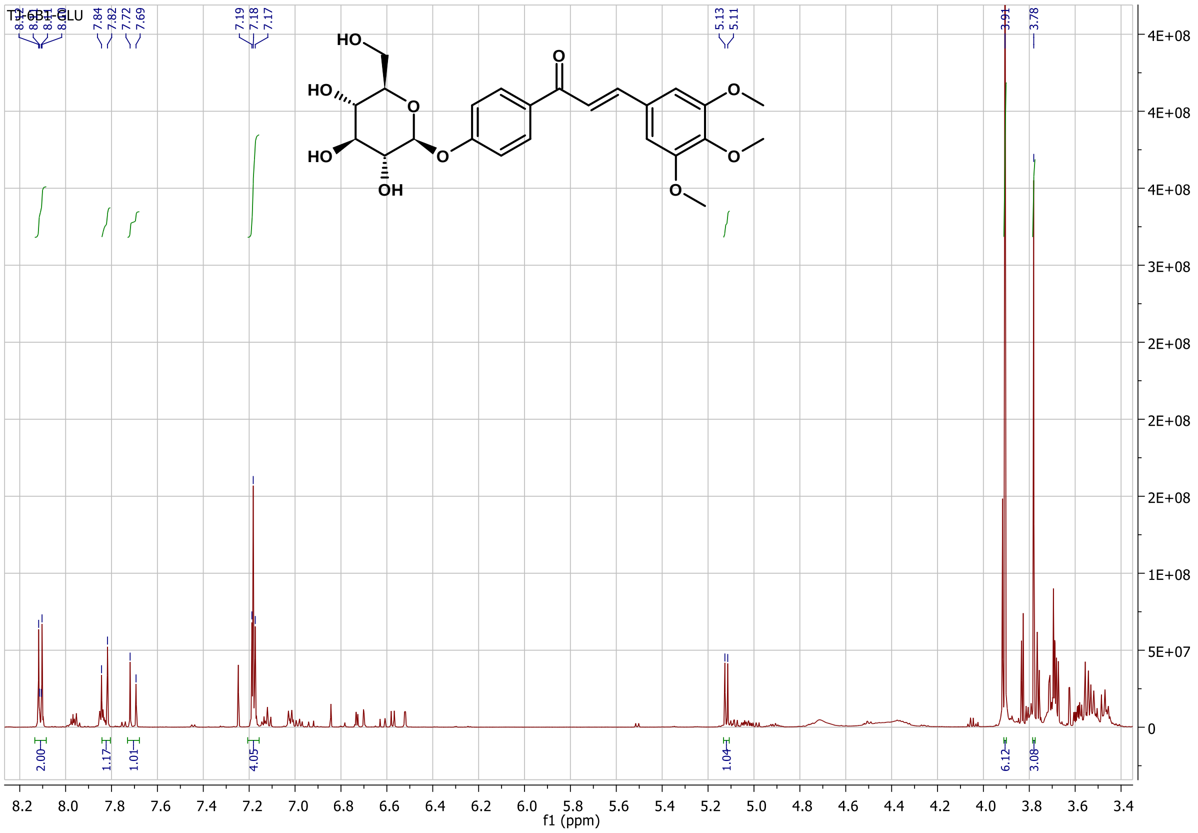


**Figure S120** $^{1}$H NMR spectrum of *trans*-4′-*O*-β-D-(glucopyranosyl)-3,4,5-trimethoxychalcone (600MHz, Acetone-d6)

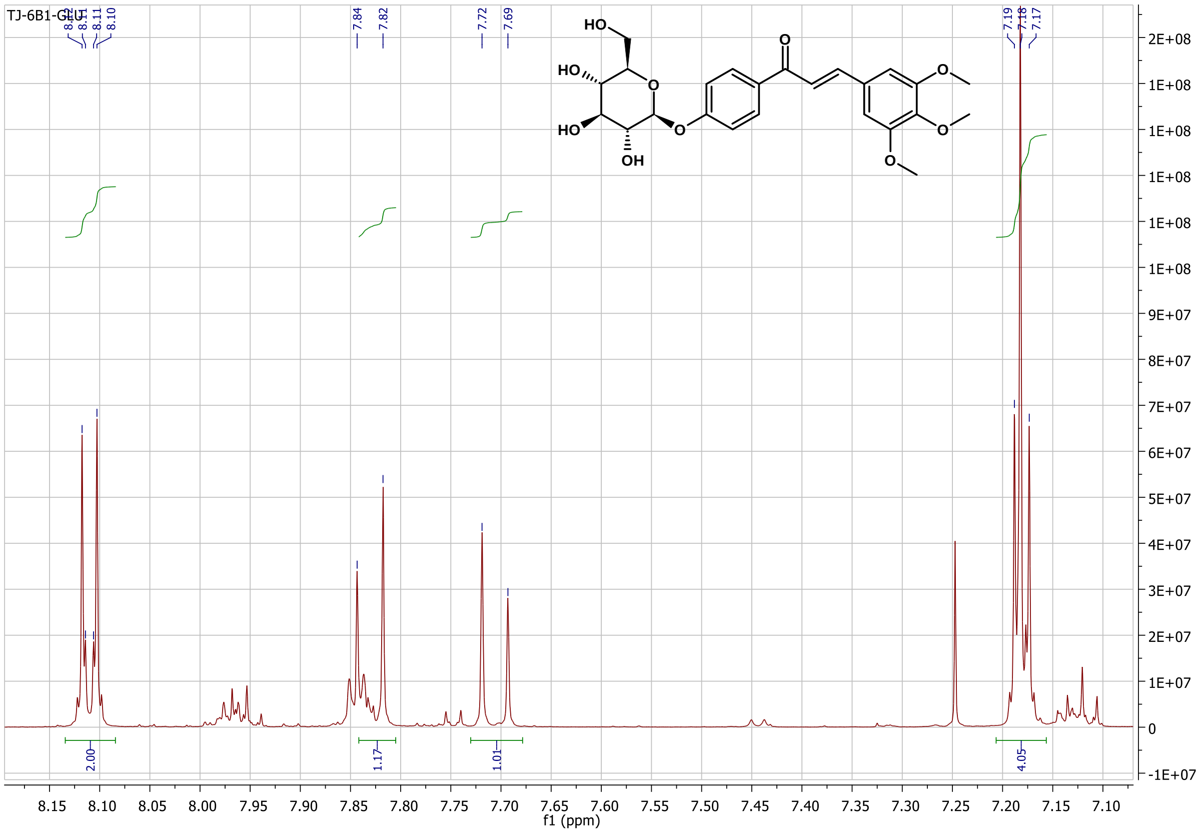

**Figure S121** Flavonoid fragment of $^{1}$H NMR spectrum of *trans*-4′-*O*-β-D-(glucopyranosyl)-3,4,5-trimethoxychalcone (600MHz, Acetone-d$_6$)

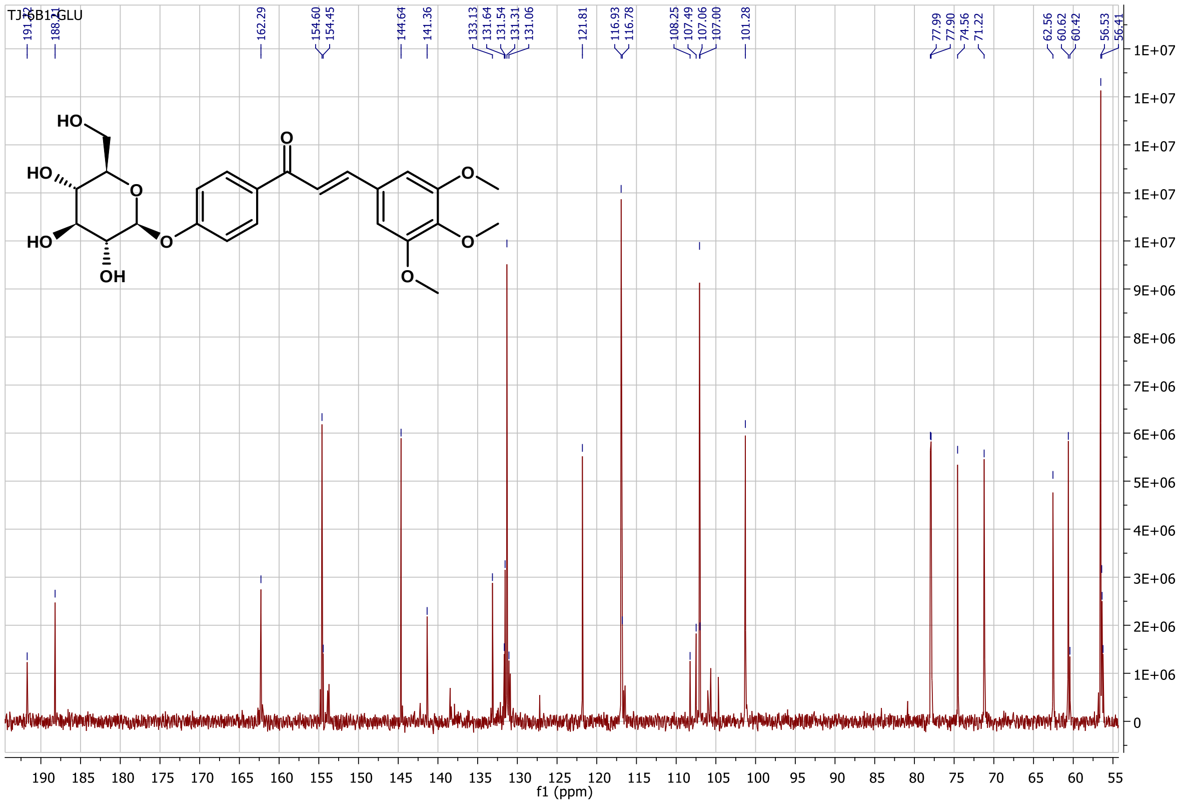

**Figure S122** $^{13}$C NMR spectrum of *trans*-4′-*O*-β-D-(glucopyranosyl)-3,4,5-trimethoxychalcone (151MHz, Acetone-d$_6$)

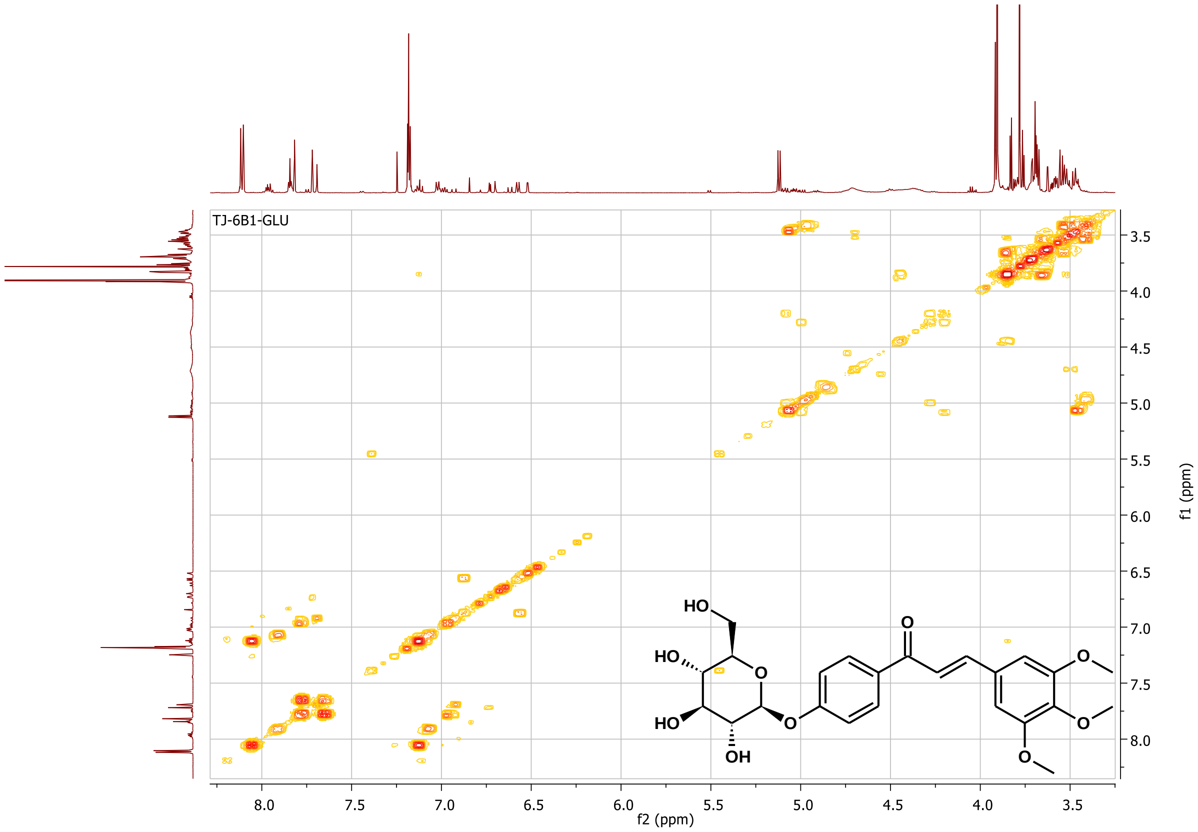


**Figure S123** COSY NMR spectrum of *trans*-4′-*O*-β-D-(glucopyranosyl)-3,4,5-trimethoxychalcone (600MHz, Acetone-d6)

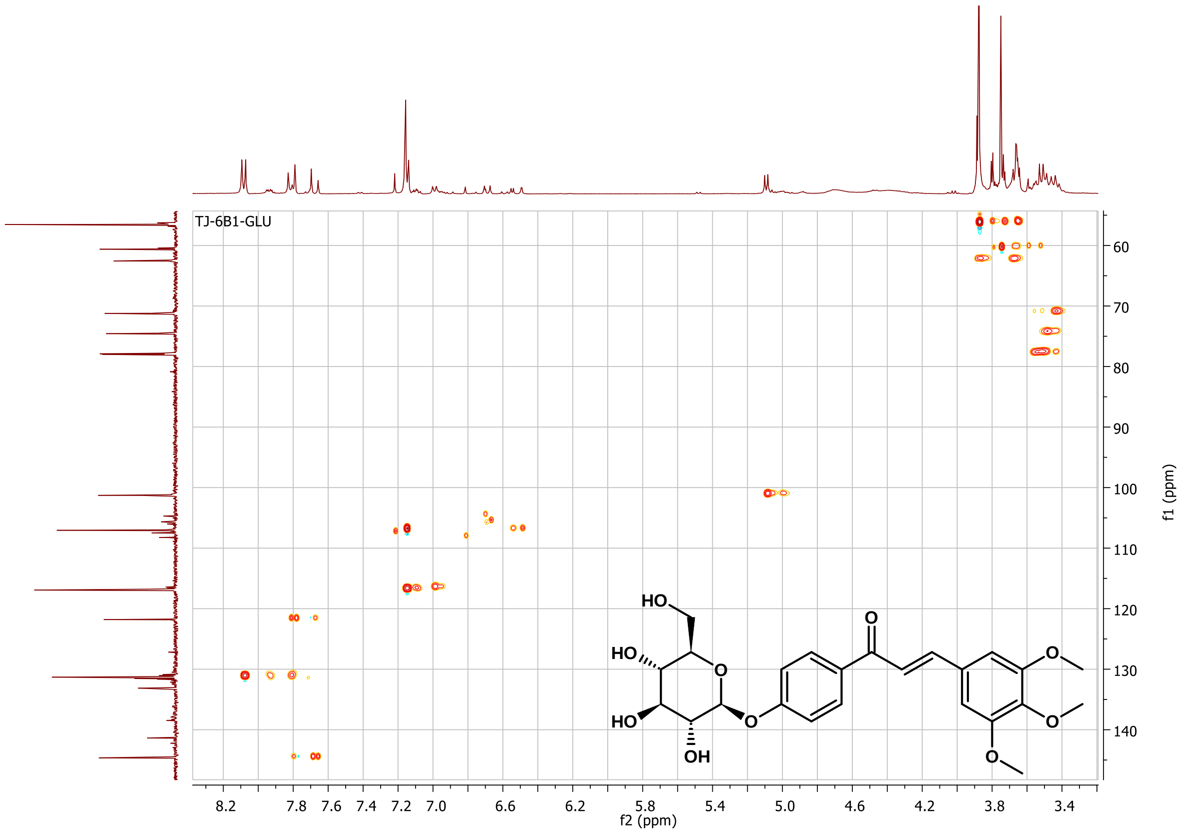


**Figure S124** HMQC NMR spectrum of *trans*-4′-*O*-β-D-(glucopyranosyl)-3,4,5-trimethoxychalcone (600MHz, Acetone-d6)

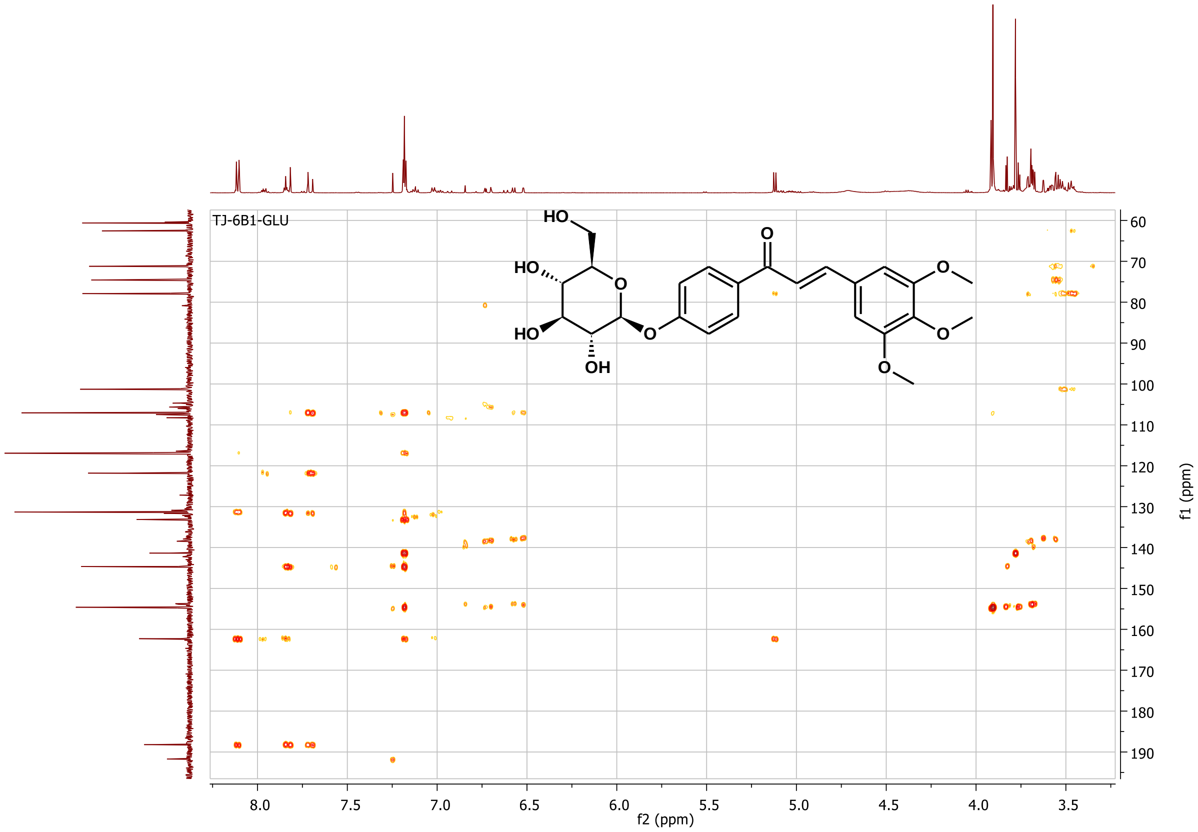


**Figure S125** HMBC NMR spectrum of *trans*-4′-*O*-β-D-(glucopyranosyl)-3,4,5-trimethoxychalcone (600MHz, Acetone-$d_6$)

**Table S1** List of recombinant enzymes used with related accession.

| GT used in the study | Source organism | NCBI accession number |
|---|---|---|
| OleD | *Streptomyces antibioticus* | OR545369 |
| YjiC | *Bacillus licheniformis* | OP381219 |
| Sbaic7OGT | *Scutellaria baicalensis* | OR545367 |
| Bet5OGT | *Cleretum bellidiforme* | OR545368 |
| UGT88F2 | *Malus x domestica* | FJ854496 |
| UGT85A2 | *Vitis vinifera* | XM_002285734.5 |
| GgCGT | *Glycyrrhiza glabra* | MH998596.1 |
| BbGT278 | *Beauveria bassiana* | OR545370 |
| GmSuSy | *Glycine max* | OP381218 |

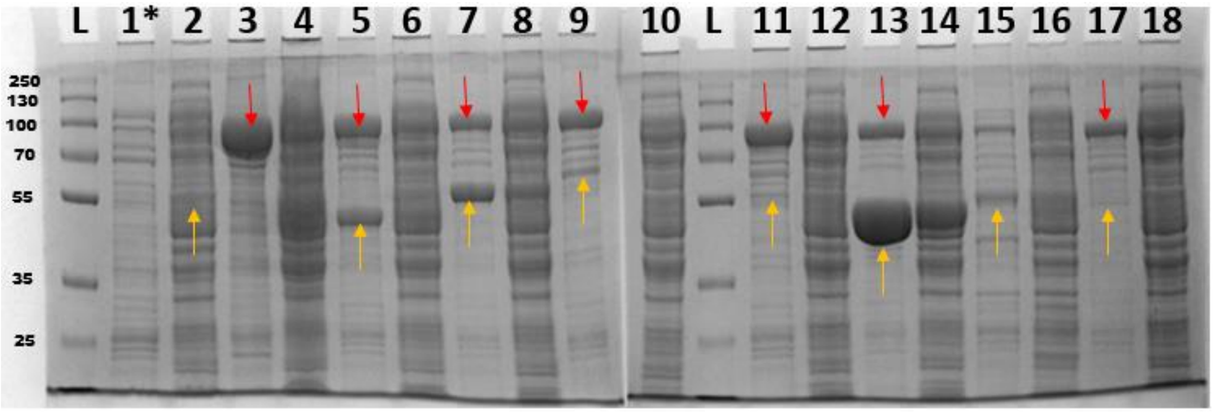


**Figure S126** SDS-PAGE analysis of crude protein lysates 20 µg protein/well (Lane: **2**, **4**, **6**, **8**, **10**, **12**, **14**, **16**, **18**) and combined fractions after Ni-NTA purification 4 µg protein/well (Lane: **1**, **3**, **5**, **7**, **9**, **11**, **13**, **15**, **17**). Lane **L** - PageRuler Plus Prestained Protein Ladder (Thermo Fisher Scientific) lane **1**-**18** E. coli BL21 (DE3) cells expressing: **1**, **2** - UGT88F2; **3**, **4** - GmSuSy; **5**, **6** - YjiC and GmSuSy; **7**, **8** - Sbaic7OGT and GmSuSy; **9**, **10** – Bet5OGT and GmSuSy; **11**, **12** – BbGT278 and GmSuSy; **13**, **14** – OleD and GmSuSy; **15**, **16** – GgCGT; **17**, **18** – UGT85A2 and GmSuSy. * First purification of UGT88F2, failed, therefore activity of the cell lysate was confirmed with phloretin as substrate and purification was repeated once again. Calculated mass for enzymes used: UGT88F2 – 53.5 kDa, GmSuSy – 93.3 kDa, YjiC – 45.7 kDa, Sbaic7OGT – 54.1 kDa. Bet5OGT – 56.3 kDa, BbGT278 – 51.3 kDa, OleD – 46.3 kDa, GgCGT – 52.9 kDa, UGT85A2 – 53.9 kDa. Red arrow points GmSuSy, Yellow arrow points GTs.

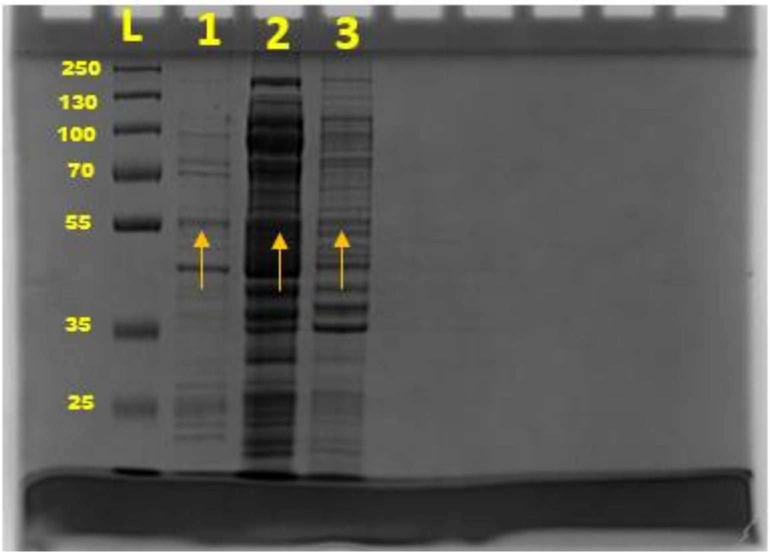


**Figure S127** SDS-PAGE analysis of repeated purification of UGT88F2. Lane **L** - PageRuler Plus Prestained Protein Ladder (Thermo Fisher Scientific Waltham, MA, USA), lane **1** - combined fractions after Ni-NTA purification (5 µg protein/well); **2** – cell pellet; **3** – crude protein lysate 20 µg (protein/well). Yellow arrow points UGT88F2.

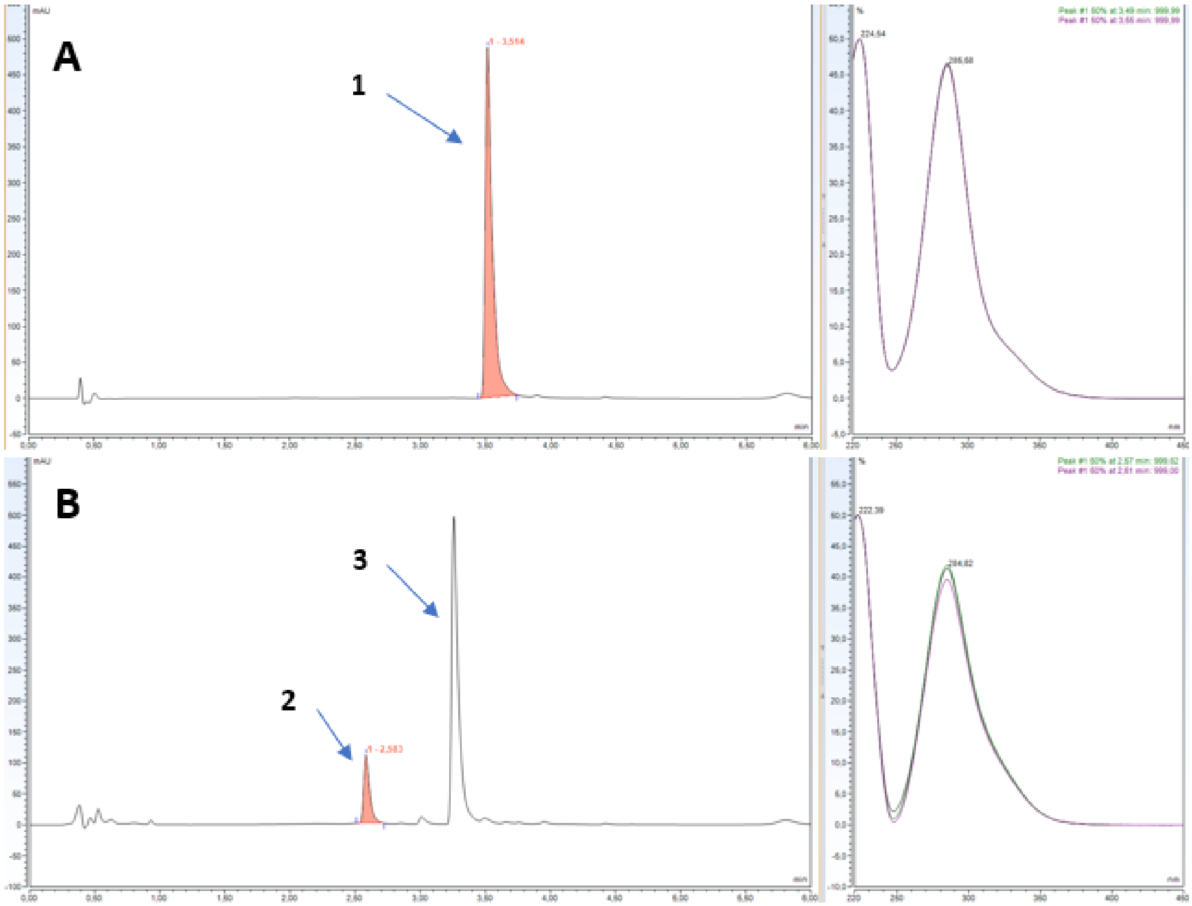


**Figure S128** UPLC chromatograms of **A** – phloretin standard, **B** – control cascade reaction of UGT88F2 crude fraction with GmSuSy performed to verify its activity. Right side are UV-Vis spectra of peaks selected in the chromatogram. **1** – phloretin; **2** – phloridzin; **3** – indole from crude cell lysate.
